# The effects of bioisostere substitution on the antimicrobial and physicochemical properties of supramolecular self-associating amphiphiles

**DOI:** 10.1039/d6md00091f

**Published:** 2026-03-23

**Authors:** Pinky K. Naicker, Mariam Yacoub, Lisa J. White, Dominick E. Balderston, Perry A. Hailey, J. Mark Sutton, Charlotte K. Hind, Jennifer R. Hiscock

**Affiliations:** a University of Kent Canterbury CT2 7NH UK J.R.Hiscock@Kent.ac.uk; b Countermeasures Development and Evaluation Preparedness, UKHSA Salisbury SP4 0JG UK Charlotte.Hind@UKHSA.gov.uk

## Abstract

The rise of antimicrobial resistance (AMR) is one of the greatest health threats facing the world today. Supramolecular self-associating amphiphiles (SSAs) have shown potential for development as novel antimicrobial agents. We now move to explore the systematic use of nonclassical bioisosteres within these systems for the first time, reporting enhanced antimicrobial efficacy against a variety of clinically relevent bacteria as a result.

## Abstract 2

The use of bioisosteres enhances the antimicrobial activity for a range of supramolecular self-associating amphiphiles.

## Graphical abstract

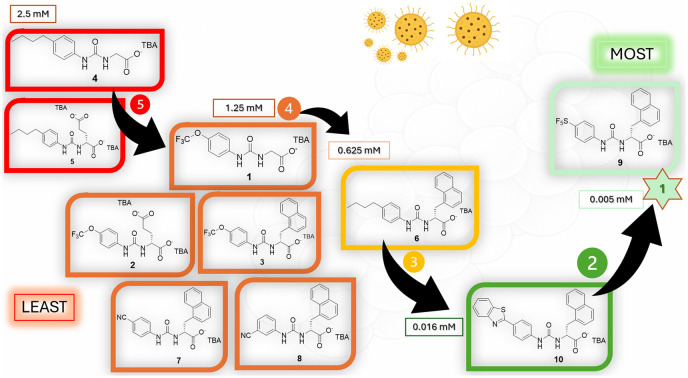

## Introduction

Antimicrobial resistance (AMR) is a critical global health threat, projected to cause ten million deaths annually by 2050, with cumulative direct and indirect deaths exceeding 200 million over the next 25 years,^1–3^ with this burden worsened by the effects of the COVID-19 pandemic.^4,5^ Addressing AMR requires coordinated strategies, including the development of novel antibiotics.^6–8^

The use of bioisosteres (classical, nonclassical, heteroatom replacements, and charged/ionic species) remains an effective design strategy within medicinal chemistry that enables systematic optimisation of molecular properties,^9–11^ including enhanced potency,^12^ desirable pharmacokinetic behaviour,^13^ reduced off-target effects,^14^ and controlled modulation of solubility, polarity, and membrane permeability.^15^ These non-classical bioisosteres include CF_3_, CN, OCF_3_, and *t*-Bu alongside the SF_5_ group, often described as a “super-trifluoromethyl (CF_3_)”.^16^ However, while substituent effects^15^ on molecular packing^17,18^ and supramolecular interactions are well studied, there are relatively few examples of true bioisosteres being explored within supramolecular antimicrobial systems. Examples include the use of SF_5_ to enhance the efficacy of 1,3,4-oxadiazole antibacterial agents against methacillin-resistant *Staphylococcus aureus* (MRSA), an effect attributed to effective target engagement and bacterial penetration.^17^ Similarly, the use of bioisosteres (CN, OCF_3_, CF_3_, CN,^19^ SF_5_) in teriflunomide derivatives,^17^ mefloquine analogues,^20^ and acylhdrazone,^21^ have improved pharmacological activity while retaining or enhancing antimicrobial efficacy.^18^

**Fig. 1:**
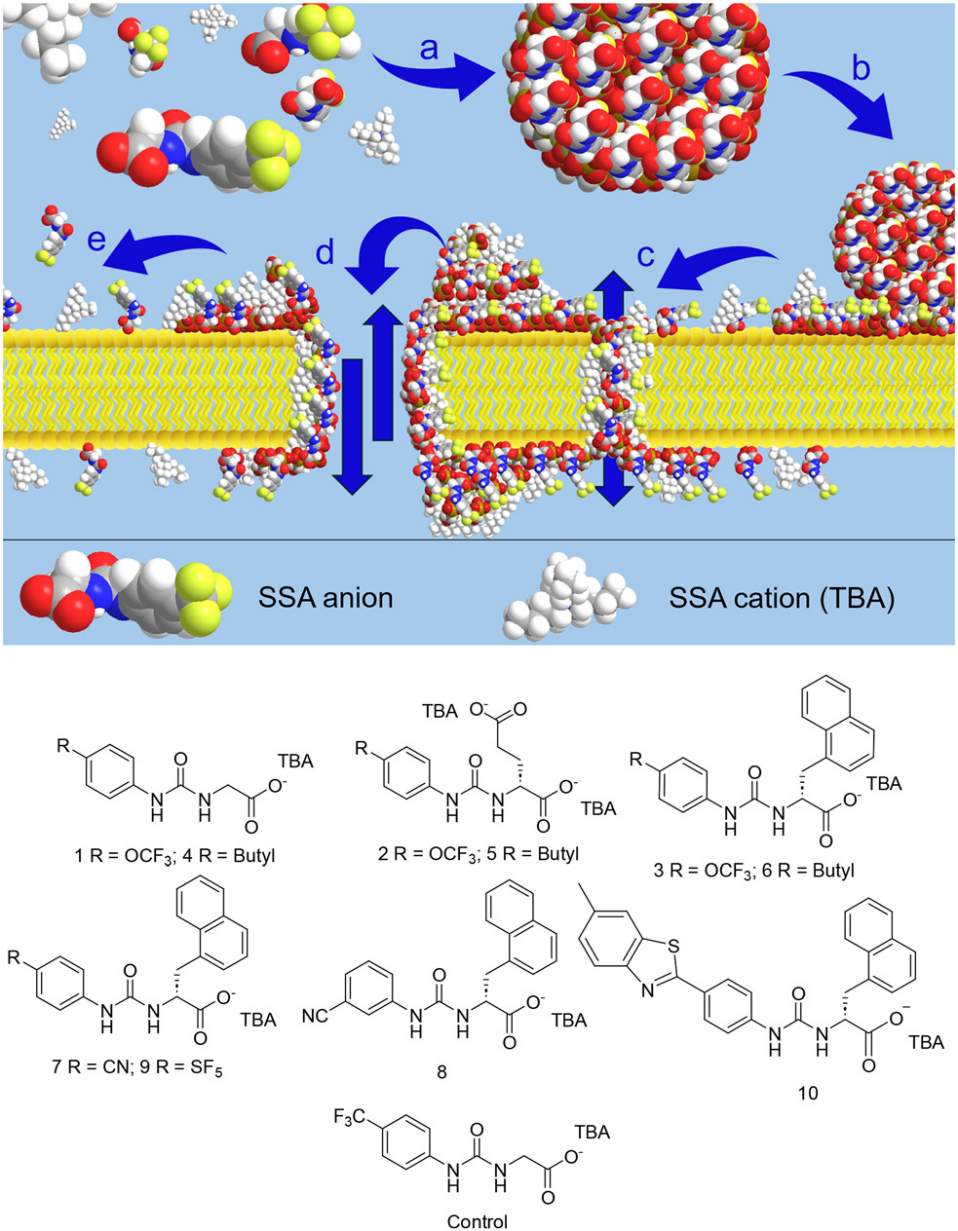
Top: A schematic illustration of the proposed interaction between SSAs and phospholipid membranes: a) in aqueous environments, SSAs spontaneously self-assemble into spherical aggregates. b) These aggregates reach the surface of the target cell membrane. Once they adhere, and reach a critical concentration the SSA molecules begin to disperse across the outer leaflet of the phospholipid bilayer. c) As favourable interactions between SSA units and phospholipid headgroups are optimised and the local SSA concentration increases, the SSAs start to self-associate within the membrane. This leads to the formation of assemblies capable of penetrating the bilayer and exhibiting channel- or pore-like behaviour. These structures remain dynamic, with SSA molecules continuing to diffuse through the phospholipid bilayer in a process that effectively lowers the concentration of SSA within/on the bilayer. d) Further increases in local SSA concentration cause more pronounced disruption of the phospholipid bilayer, enhancing membrane permeability. e) When SSA levels on either leaflet decrease below a critical level, the transmembrane/membrane disruption events can no longer be supported, allowing the phospholipid bilayer to return to its intact, functional state.^23^ Bottom: The chemical structures of novel SSAs 1–10, plus a control SSA, from which the structures of 1–10 are derived. TBA = tetrabutylammonium.

Supramolecular self-associating amphiphiles (SSAs) are a class of amphiphilic salt and related compounds that have shown antimicrobial activity towards a variety of clinically relevant planktonic and biofilm bound Gram-positive and Gram-negative bacteria.^22–24^ They also act as antibiotic efficacy enhancers^25^ and have shown a drug-like profile following *in vitro* and *in vivo* DMPK assessment.^23,24^ We hypothesise, as summarised in Fig. 1 (top), that the SSA mode of action includes the formation of self-associated spherical aggregates, with a hydrodynamic diameter (*d*_H_) ∼100–550 nm. Upon interaction with a target cell surface, these SSA aggregates morph into a coating, reaching a critical concentration that enables cellular entry, through a variety of mechanisms, including those which exhibit ion channel/pore characteristics under patch clamp analysis.^23^ Therefore, although SSAs appear simple, the inherent ability to self-associate with both themselves and target cells within an environment that results in desired biological activities, adds complexity that may not be resolved using traditional analysis pathways. With this complexity in mind we now move to enhance the efficacy and broad spectrum antimicrobial effects of SSAs through the use of bioisosteres (Fig. 1 – bottom).

## Results and discussion

SSAs 1–8 were synthesised through the reaction of the appropriate amine and isocyanate, while SSAs 9 and 10 were produced through the reaction of two different primary amines with triphosgene. We have previously published the control SSA (Fig. 1)^26^ which contains a CF_3_ functionality, used in the field of synthetic ion transporters to increase phospholipid membrane solubility, while acting as an electron withdrawing functionality, that is able to enhance the hydrogen bond donating properties of urea (or analogous) groups present within the same conjugated system. Building on the antimicrobial and physicochemical data that we have already gathered for SSAs, we now explore the effect of incorporating nonclassical bioisosteres to the CF_3_ group into the structures of 1–10. These groups include the OCF_3_ (1–3), butyl (4–6), CN (7 and 8) and SF_5_ (9), alongside the less traditional benzothiazole (10) functionality. The OCF_3_, butyl, CN, SF_5_ and benzothiazole functionalities were chosen as bioisosteres for the CF_3_ group to closely simulate aspects of electronic, steric and lipophilic behaviour. The OCF_3_ functionality closely simulates the electronic and lipophilic properties of the CF_3_ group, offering comparable electron withdrawing and highly hydrophobic properties, while maintaining a similar steric profile.^27^ the butyl functionalise acts as a hydrophobic isostere, rather than an electronic isostere, investigating the effects of maintaining hydrophobic properties, while changing electronic ones.^28^ The CN group, is strongly electron-withdrawing so offers similar electronic properties, but is smaller than the CF_3_ functionality – reducing steric bulk.^29^ As previously mentioned the SF_5_ group, known as the “super CF_3_” combines a very strong electron withdrawing properties, with increased lipophilicity and exceptional metabolic stability.^30^ Finally, the benzothiazole functionality offers a bulky, hydrophobic, electron-poor heteroaromatic system that can mimic CF_3_'s role as a steric/lipophilic anchor while introducing additional π-stacking or heteroatom-mediated interactions.^29^

We also build on previously published results, showing that the introduction of a dicarboxylate functionality can selectively enhance antimicrobial activity against target microorganisms,^24^ and the introduction of a naphthalene group to stabilise transmembrane membrane channel/pore-like structures, through extended pi–pi stacking interactions.^23^

**Fig. 2:**
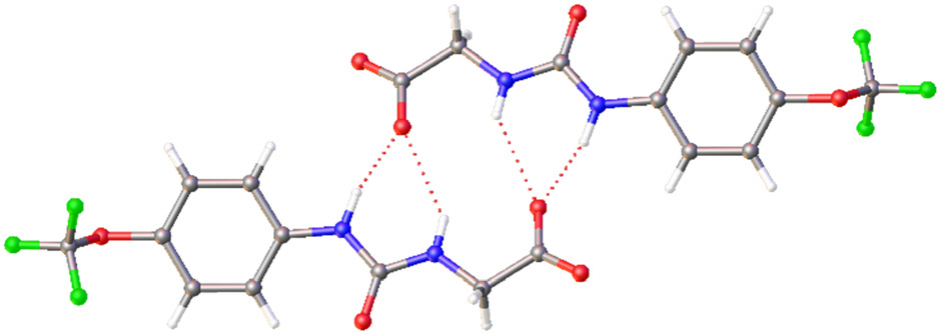
Single crystal X-ray structure obtained for 1. The TBA counter cation and H_2_O solvent molecule(s) have been omitted for clarity. Grey = carbon, blue = nitrogen, red = oxygen, white = hydrogen, green = fluorine, red dashed lines = hydrogen bonds.^31–33^

The single crystal X-ray diffraction structure obtained for 1 showed that the substitution of the CF_3_ functionality for the OCF_3_ group maintained the most prevalent hydrogen bonding mode observed for SSAs to date, in which the anionic component of the SSA forms a dimer, stabilised through the formation of four hydrogen bonds (Fig. 2).

Moving into the solution state, the physicochemical properties of these SSAs systems were characterised in line with our previously published standardised protocols.^22–26^ The self-associative properties of 1–10 were first explored in DMSO(−*d*_6_), a polar organic solvent which is known to simplify SSA self-associative events.^26^ As shown in Table 1, quantitative (q) ^1^H NMR experiments, confirmed all SSAs with the exception of 7 form higher-order aggregates with solid-like properties at 112 mM, with the OCF_3_ functionality (1–3) and increased hydrophilicity (2) leading to a greater proportion of the SSA to be incorporated into these higher-order aggregates when compared to the butyl appended compounds (4–6). The presence of a naphthalene ring (3, 6–10) was also found to increase the proportion of compound incorporated into higher-order species exhibiting solid-like properties, presumably through the presence of intermolecular pi–pi interactions, with the exception of 6 and 7. Interestingly moving the CN group from the *para* (7) to the *meta* (8) position, dramatically increased the SSA aggregation properties. However, dynamic light scattering (DLS) measurements support the hypothesis that the higher order structures evidenced in qNMR spectroscopy studies to be small in size, with the correlation function providing evidence for a lack of stability.

**Table 1:** Overview of the results obtained from qNMR spectroscopy, where the proportion of an SSA to adopt solid-like properties and therefore become NMR silent (error = ± 10%) is confirmed through comparative integration against an internal standard (DCM 1%), and DLS studies confirm the hydrodynamic diameter (*d*_H_) through the reporting of intensity distribution peak maxima, performed in DMSO-*d*_6_ 1% DCM and DMSO solutions respectively at 112 mM and 298 K.^22–24,26^ Cont. = control, Fig. 1

SSA	qNMR (%)		*d*_H_ (nm)
	Anion	Cation	
Cont.^26^	0	0	b
1	15	14	<1, 3, 48
2	72^a^	78^a^	<1, 38
3	47	47	<1, 100
4	5	5	<1, 7^c^
5	25^a^	24^a^	b
6	9	6	<1, 67
7	0	0	b
8	46	43	1
9	50	48	2
10	29	28	<1

a Slow exchange means these data should be treated with caution.

b No evidence of stable higher order self-associated structures.

c Wide range of structures *d*_H_ = 10–100 nm.

Moving from an organic polar solvent, to a H_2_O (or D_2_O)/5% EtOH solution (Table 2), where the EtOH is present to act as an internal standard in qNMR spectroscopy experiments, alongside aiding SSA solubility, we see that the substitution of the CF_3_ (control) for a butyl chain (6) or SF_5_ (9) functionality, in addition to a naphthalene group, increasing hydrophobicity and supporting intermolecular pi–pi stacking interactions, increases the proportion of SSA to be incorporated into higher-order aggregates. In addition, and in comparison to the control SSA, DLS and zeta potential data support the formation of higher-order aggregates with enhanced stability, with this increased stability linked to the presence of both the dicarboxylate or naphthalene functionalities, specifically the SF_5_ (9) and benzothiazole (10) containing examples. Further to this the presence of the naphthalene group was found to drive down the critical aggregation concentration (CAC), defined as the concentration at which should any additional SSA be added to a H_2_O/5% EtOH solution, the SSA will be incorporated into higher-order aggregated species. The greatest decrease in CAC was noted upon the addition of the OCF_3_ (3) or SF_5_ (9) and naphthalene functionalities. However, as detailed within the SI, alterations to the SSA structure results in an array of subtle, and sometimes non-traditional differences related to surfactant behaviour.

**Table 2:** Overview of the results obtained from: qNMR spectroscopy (error = ± 10%), where the proportion of an SSA to adopt solid-like properties and therefore become NMR silent is confirmed through comparative integration against an internal standard (EtOH 5%); CAC (obtained by tensiometry, ST = surface tension); zeta potential; and DLS studies confirm the stability and hydrodynamic diameter (*d*_H_) through the reporting of intensity distribution peak maxima. All experiments were performed in D_2_O or H_2_O/5% EtOH solutions (as appropriate) at 5.56 mM and 298 K, unless stipulated otherwise.^22–24,26^ Cont. = control, Fig. 1

SSA	qNMR (%)		CAC (mM)	ST at CAC (mN m^−1^)	*d*_H_ (nm)	Zeta potential (mV)
	Anion	Cation				
Cont.^26^	68	a	11.2	39	220	−37
1	54	56	4.50	43	188^b^	−48
2	53	50	c	c	409^d^	−64
3	56	56	0.52	48	422	−66
4	25	20	18.5	35	3, 222	−47
5	67	66	12.5	35	125	−71
6	88	e	1.45	38	116	−71
7	14	14	2.00	47	186	−82
8	29	28	4.35	44	225	−72
9	74	76	1.10	42	364^b^	−92
10	f	f	g	g	155^f^	−94^f^

a Data not collected at the time of original publication.

b Some evidence of higher order structures *d*_H_ >1000 nm.

c CAC above the limit of solubility, surfactant properties observed.

d Correlation function raises concerns relating to sample stability.

e Signal overlap prevented the reporting of accurate data.

f Studies conducted at 1.0 mM due to SSA solubility, Accurate integration of SSA anion and cation signals could not be obtained due to low signal intensity and signal overlap.

g No surfactant properties observed to the limit of SSA solubility.

Table 3 summarises the results of simple low level computational simulations, using Spartan '24 to estimate the *E*_max_ and *E*_min_ electrostatic surface potential values of the SSA anion,^34,35^ which correspond to the hydrogen bond donor and acceptor functionalities respectively. When these data are combined with consensus (c) log *P* values obtained from SwissADME,^36^ these data suggest that the potential for stronger intermolecular hydrogen bond formation, combined with an enhanced clog *P* contribute to the increased stability of the self-associated higher-order aggregates produced by 1–10 in comparison to the control. It should be noted that these simple computational models do not account for any intermolecular pi–pi stacking interactions that may also occur, facilitated by the naphthalene and benzothiazole groups, or the presence of double the amount of TBA counter cation in the case of 2 and 4. Attempts to quantify the strength of lower-order SSA anion dimerization events in a H_2_O/5% EtOH solution failed for all but 10, as the intrinsic fluorescent properties of this SSA enabled the experiment to be conducted at much lower concentrations. Here the results of UV-vis (1–9) or fluorescence emission (10) spectroscopy dilution study data were fitted to both the EK and Co-EK self-associative binding isotherm models using Bindfit v0.5.^37^ These binding isotherm models assume single component, one-dimensional polymerisation events – to include single component dimerisation, with the EK binding isotherm assuming the association constant for all self-association events within the polymerisation process to be the same, and the Co-EK model assuming the first self-association event to differ in strength from all subsequent self-associative events. In this instance only the data obtained for 10 was considered a good fit to either of these binding isotherm models, in this case the EK dimerisation binding isotherm model. The fit of these data to the EK dimerisation binding isotherm model was supported by the data summarised in Table 2, which confirms these fluorescence emission dilution study data to be collected at a concentration far below the CAC. The data obtained from the dilution of 1–9 proved to complex to fit to either the EK or Co-EK binding isotherm models.

**Table 3:** Overview of the results obtained from low-level *in silico* modelling of the SSA anion. Electrostatic surface potential maxima (*E*_max_) and minima (*E*_min_) values were obtained following energy minimisation using Spartan '24.^34,35^ clog *P* values were obtained using SwissADME.^36^*K*_dim_ values were obtained through the fitting of fluorescence emission (<0.001 mM) UV-vis spectroscopy (<0.001 M) dilution study data, obtained from an H_2_O/5% EtOH solution at 298 K, to both the EK and CoEK self-associative binding isotherm models using Bindfit v0.5.^37^ Cont. = control, Fig. 1

SSA	Surface potential (kJ mol^−1^)		clog *P*	*K*_dim_ (M^−1^)	± error (%)
	*E*_max_	*E*_min_			
Cont.	−51	−722	4.02	a	a
1	−48	−735	1.21	b	b
2	−235	−1029	1.02	a	a
3	−48	−715	3.70	c	c
4	−64	−757	1.65	b	b
5	−163	−1038	1.48	b	b
6	−63	−734	4.23	c	c
7	−41	−715	2.60	d	d
8	−41	−715	2.60	d	d
9	−20	−699	4.16	c	c
10	−29	−718	4.02	3570^d^	0.59^d^

a Higher order aggregation events hypothesised to exist, data could not be fitted to either the EK or CoEK self-associative isotherm.

b no evidence of molecular association observed.

c Evidence of low order and high order self-association, data could not be fitted to either the EK or CoEK self-associative isotherm with confidence.

d Obtained from the fitting of fluorescence emission data, excitation = 331 nm.

With an understanding of the physicochemical self-associative properties of 1–10 achieved, we next moved to elucidate the antimicrobial efficacy (minimum inhibitory concentration – MIC, the minimum amount of an SSA required to inhibit visible bacterial growth) of these 10 novel SSAs against a panel of clinically relevant, drug resistant and susceptible Gram-positive (*S. aureus*) and Gram-negative (*Klebsiella pneumoniae*, *Acinetobacter baumannii*, *Pseudomonas aeruginosa*, *Escherichia coli*) bacteria. Within the antimicrobial efficacy studies, the SSAs are supplied, pre-aggregated within a H_2_O/5% EtOH solution, and giving the stability of those aggregates produced (Table 2) and previous data acquired, we are confident that the aggregated form of the SSAs is maintained post initial introduction into the microbial culture, before any interaction with a target cell is made.^22^ As summarised in Table 4, when compared to the control SSA, those SSAs that exhibited far greater stability in H_2_O/5% EtOH solution (Table 2) were also found to exhibit a far greater antimicrobial efficacy towards both *S. aureus* strains (MIC: 9 ≈ 10 < 6). However, there was no discernible activity noted against any Gram-negative bacteria. Encouragingly, when comparing the antimicrobial activity of 9 and 10 to the known antibiotic ciprofloxacin, the activity of these SSAs was found to be competitive when considering the MIC results generated against the *S. aureus* strain NCTC 13616.

**Table 4:** Overview of MIC (mM, *n* = 3) results obtained for 1–10 against *S. aureus* (ATCC 9144, NCTC 13616), *K. pneumoniae* (M6), *A. baumannii* (ATCC 17978), *P. aeruginosa* (PAO1, NCTC 13437) and *E. coli* (NCTC 12923) strains. Cont. = control, Fig. 1. CIP = ciprofloxacin. PMB = polymyxin B

SSA	ATCC 9144	NCTC 13616	M6	ATCC 17978	PAO1	NCTC 13437	NCTC 12923
Cont.^23^	1.390	>5	N/A	N/A	N/A	N/A	N/A
1	1.250	2.500	>5^a^	>5^a^	>5^a^	>5^a^	>5^a^
2	1.250	1.250	>5^a^	>5^a^	>5^a^	>5^a^	>5^a^
3	1.250	0.625	>5^a^	>5^a^	>5^a^	>5^a^	>5^a^
4	2.500	>5	>5^a^	>5^a^	>5^a^	>5^a^	>5^a^
5	2.500	5.000	>5^a^	>5^a^	>5^a^	>5^a^	>5^a^
6	0.625	0.625	>5^a^	>5^a^	>5^a^	>5^a^	>5^a^
7	1.250	>5	>5^a^	>5^a^	>5^a^	>5^a^	>5^a^
8	1.250	5.000	>5^a^	>5^a^	>5^a^	>5^a^	>5^a^
9	0.005	0.005	>5^a^	>5^a^	>5^a^	>5^a^	>5^a^
10	0.016	0.030	>0.5^b^	>0.5^b^	>0.5^b^	>0.5^b^	>0.5^b^
CIP^c^	1	128	0.06	0.5	0.5	64	0.008
PMB^c^	N/A	N/A	1	1	2	4	0.06

a Due to a lack of activity, only two biological repeats were undertaken.

b Solubility prevented testing at higher concentrations.

c Concentrations provided in μg mL^−1^.

Due to the hypothesised mode of action of these SSAs being associated with membrane association/permeation/disruption events, we theorised that the limit to the activity of 1–10 may be the ability of the SSAs to breach the outer of the two Gram-negative phospholipid membranes. To test this hypothesis we determined the antimicrobial efficacy of these same 10 SSAs in the presence of a sub-inhibitory dose of polymyxin b nonapeptide (PMBN), an outer membrane permeabilising agent, Table 5.^38^ Here the presence of PMBN was shown to activate the antimicrobial efficacy of 1–10 against the Gram-negative bacteria, with the naphthalene based SSAs, substituted with an SF_5_ (9) > butyl chain (6) > OCF_3_ (3) group showing the greatest broad spectrum antimicrobial activity.

**Table 5:** Overview of MIC (mM, *n* = 3) results obtained for 1–10 in the presence of PMBN (15 μg mL^−1^) against *K. pneumoniae* (M6), *A. baumannii* (ATCC 17978), *P. aeruginosa* (PAO1, NCTC 13437) and *E. coli* (NCTC 12923) strains

SSA	M6	ATCC 17978	PAO1	NCTC 13437	NCTC 12923
1	>5	5.000	5.000	>5	5.000
2	>5	5.000	5.000	>5	2.500
3	0.625	>5	0.040	0.625	0.313
4	>5	>5	2.500	5.000	>5
5	>5	>5	1.250	2.500	2.500
6	0.300	0.300	0.080	0.300	0.300
7	>5	>5	5.000	>5	>5
8	>5	>5	2.500	>5	>5
9	0.02–0.3	0.010	0.005	>5	0.020
10	>0.5^a^	>0.5^a^	0.125	>0.5^a^	0.5^a^
PMBN^b^	>30	>30	≥30	>30	>30

a Solubility prevented testing at higher concentrations.

b Concentrations provided in μg mL^−1^.

## Conclusions

As exemplified by the differences in the trends presented within the datasets collected for SSAs 1–10 from DLS, zeta potential and surface tension (used to calculate CAC) measurements, it is clear that minor substitutions to the SSA anion scaffold can be used to alter the surfactant, size and stability of any resultant higher-order self-associated aggregate. However, we have also shown that the incorporation of common bioisosteres, especially the SF_5_ functionality, into the structure of SSAs can not only be used to modulate physicochemical properties, although at present the structure activity relationships remain unclear, but also to increase the activity of this class of supramolecular antimicrobials against a panel of clinically relevant Gram-positive and Gram-negative bacteria (where PMBN is present). What becomes clear from this work, is the next challenge for SSAs will be to learn how to engineer these complex systems to retain antimicrobial efficacy against Gram negative pathogenic bacteria in the absence of PMBN.

## Author contributions

P. K. N., L. J. W.: investigation; validation; writing – original draft, review and editing. M. Y., D. E. B., P. A. H.: Investigation; validation. J. M. S., C. K. H.: supervision; validation; writing – review & editing; funding acquisition. J. R. H.: conceptualization; funding acquisition; project administration; supervision; writing – original draft, review & editing.

## Conflicts of interest

There are no conflicts to declare.

## Supplementary Material

MD-017-D6MD00091F-s001

MD-017-D6MD00091F-s002

## Data Availability

Supporting data are provided in the supplementary information (SI). Supplementary information: this includes experimental details, DLS data, zeta potential data, tensiometry data, mass spectrometry data, NMR spectroscopy data, crystallography data, UV-vis and fluorescence spectroscopy data, molecular characterisation data, and biological data. See DOI: https://doi.org/10.1039/d6md00091f. CCDC 2435071 contains the supplementary crystallographic data for this paper.^39^
