# Supplementary material for: The effects of bioisostere substitution on the antimicrobial and physicochemical properties of supramolecular self-associating amphiphiles
Source: RSC Med Chem. 2026 Mar 23;17(4):2151–6. doi: 10.1039/d6md00091f (PMC13007787; doi:10.1039/d6md00091f)
Supplement: MD-017-D6MD00091F-s001 [file MD-017-D6MD00091F-s001.pdf]

Supplementary Information (SI) for RSC Medicinal Chemistry.
This journal is © The Royal Society of Chemistry 2026

Supplementary Materials

Table of Contents

Section S1: Chemical structures 2
Section S2: General methods 3
Section S3: Chemical synthesis 5
Section S4: Single Crystal XRD data 9
Section S5: NMR Spectroscopy Data 10
Section S5.1: ^{1}H NMR Spectroscopy Data 10
Section S5.2: $^{13}C\{^{1}H\}$ NMR spectroscopy data 16
Section S5.3: ^{1}H Quantitative NMR (qNMR) Spectroscopy Data 21
Section S5.3.1: ^{1}H qNMR spectra in DMSO-d_6/1% DCM 21
Section S5.3.2: ^{1}H qNMR spectroscopy in D_2O/5 % EtOH 26
Section S6: Dynamic Light Scattering Studies 32
Section S6.1: Data in D_2O/5 % EtOH 32
Section S6.2: DLS data in DMSO 43
Section S7: Stability and Surface Tension Data 52
Section S7.1: Zeta potential data 52
Section 7.2: Surface tension and determination of critical aggregation concentration (CAC) 58
Section S8: Low level in-silico modelling 64
Section S8.1: Electrostatic potential maps 64
Section S8.2: Modelling *in vitro* ADME properties 70
Section S8.3: Modelling data references 71
Section S9: UV-Vis and fluorescence dilution study data 72
Section S9.1 UV-Vis dilution data 72
Section S9.2: Fluorescence data 77
Section S9.3: Bindfit data 78
Section S9.3.1: EK and CoEK binding isotherm models 78
Section S10: Mass Spectrometry data 93

Section S1: Chemical structures

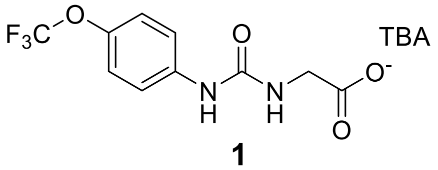

1

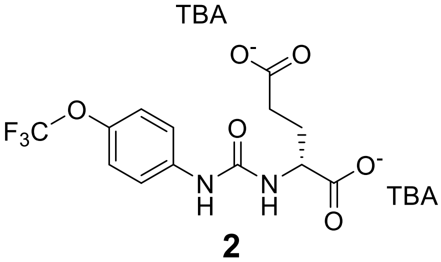

2

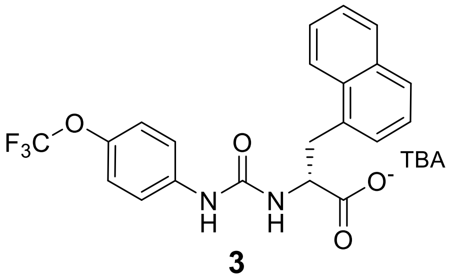

3

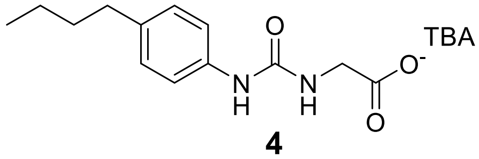

4

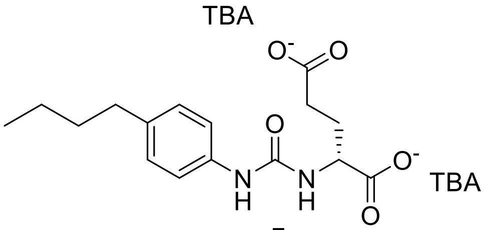

5

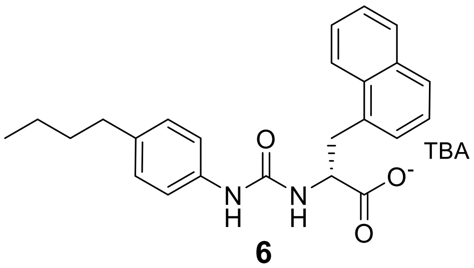

6

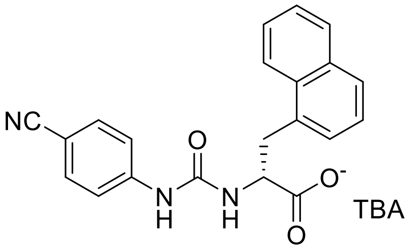

7

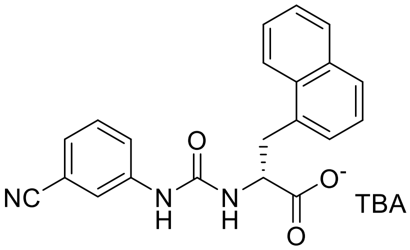

8

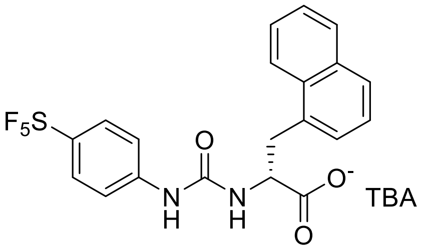

9

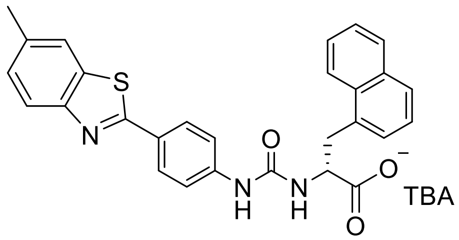

10

Figure S1 - Chemical Structures of **1-10**. TBA = Tetrabuytlammonium.

Section S2: General methods

General remarks: A positive pressure of nitrogen and oven dried glassware were used for all reactions. All solvents and starting materials were purchased from known chemical suppliers or available stores and used without any further purification unless specifically stipulated. The NMR spectra were obtained using a Bruker AV2 400 MHz or AVNEO 400 MHz spectrometer. The data was processed using TopSpin 4.1.4. software. NMR chemical shift values are reported in parts per million (ppm) and calibrated to the centre of the residual solvent peak set (s = singlet, br = broad, d = doublet, t = triplet, q = quartet, m = multiplet). Tensiometry measurements were undertaken using the Biolin Scientific Theta Attension optical tensiometer. The data was processed using Biolin OneAttension software. A Hamilton (309) syringe was used for these measurements. The melting point for each SSA was measured using Stuart SMP10 melting point apparatus. High resolution mass spectrometry was performed using a Waters Acquity H-Class Quaternary Solvent Manager (QSM) and Flow Through Needle (FTN), configured to allow direct injection into a Waters V-ion.

Quantitative ^{1}H NMR (qNMR) spectroscopy studies: Proton qNMR spectroscopy studies of all compounds were conducted in an aqueous D_2O/5 % EtOH and an organic DMSO-d_6/1 % DCM solvent system. Ethanol and DCM served as internal standards for the aqueous and organic systems respectively. Comparative integration of diagnostic signals was used to assess the relative extent of aggregation and signal 'loss' in each system. A target concentration of 5.56 mM in the aqueous and 112 mM in the organic system were applied (unless changed due to solubility issues). A weighing tolerance of ± 0.05 mg/mL was deemed acceptable. qNMR spectra were acquired on a 400 MHz spectrometer at 298 K with a relaxation time (d_1) of 60 seconds. Spectra were recorded with 16 scans and a spectral width of 12 ppm.

Fourier transform infrared spectroscopy: Infrared spectra were obtained using a Shimadzu ATR-FTIR spectrometer. Samples were analysed neat using the attenuated total reflectance (ATR) method without further preparation. Absorption maxima are reported as ν_{max} (cm^{-1}) using IRsolution software.

DLS studies: All vials used for preparing the samples were clean and dry. All solvents used were filtered to remove any particulates that may interfere with the results obtained. Samples of differing concentrations were obtained through serial dilution of a concentrated solution. All samples underwent an annealing process, in which they were heated to approximately 313 K before being allowed to cool to room temperature, allowing each sample to reach a thermodynamic minimum. The studies were carried out using Anton Paar Litesizer™ 500 and processed using Kalliope™ Professional software. A series of 10 consecutive runs were recorded and averaged at 298 K.

Zeta potential (ZP) studies: All vials used for preparing the samples were clean and dry. All solvents used were filtered to remove any particulates that may interfere with the results obtained. All samples underwent an annealing process in which the various solutions were heated to approximately 313 K before being allowed to cool to room temperature, allowing each sample to reach a thermodynamic minimum. The ZP studies were carried out using Anton Paar Litesizer™ 500 and processed using Kalliope™ Professional software. The final zeta potential value given is an average of the number of the ten consecutive experiments conducted at 298 K.

Tensiometry studies: All samples were prepared in an H_2O/5 % EtOH solution. All samples underwent an annealing process in which the compound containing solutions were heated to approximately 313 K before being allowed to cool to room temperature, allowing each sample to reach a thermodynamic minimum. All samples were prepared through serial dilution of the most concentrated sample. Three surface tension measurements were obtained for each sample at a given concentration, using the pendant drop method. The average of the three values were plotted to calculate the critical aggregation concentration (CAC).

Low level in-silico modelling: Computational calculations to identify primary hydrogen bond donating and accepting sites were conducted in line with studies reported by Hunter using Spartan '24.[1] Calculations were performed using semi-empirical PM6 methods, after energy minimisation calculations, to identify E_{max} and E_{min} values. PM6 was used over AM1 in line with research conducted by Stewart.[2]

Biological studies: Antimicrobial susceptibility testing for SSAs **1-10** was performed following EUCAST guidelines for microbroth minimum inhibitory concentrations (MIC), with adaptations specific to the physicochemical properties of the SSAs. MICs were conducted in tryptic soy broth (TSB) using polystyrene microtiter plates. Stock solutions of the SSA compounds were prepared in (5% v/v) DMSO to facilitate solubilisation; mixtures were gently annealed and allowed to equilibrate to room temperature prior to use. Serial two-fold dilution series were generated in the same solvent system before inoculation with bacterial suspensions prepared in concentrated TSB at a final concentration of 5 x 10^5 CFU/mL. When included, the outer membrane permeabiliser Polymyxin-b-nonapeptide (PMBN) was incorporated into assays to a final concentration of 15 µg/mL.

Fluorescence and UV-Vis studies: All vials used for preparing the samples were clean and dry. All solvents used were filtered to remove any particulates that may interfere with the results obtained. Samples of differing concentrations were obtained through serial dilution of a concentrated solution. All samples underwent an annealing process, in which they were heated to approximately 313 K before being allowed to cool to room temperature, allowing each sample to reach a thermodynamic minimum. Clear and black bottom 96-well plates were prepared by serially diluting solution (20 dilutions) across the plate, the appropriate SSA solution (100 µL, 1.00 mM) was added to each well to give a total well volume of 200 µL. Data were acquired in endpoint mode. All experiments were repeated in triplicate to ensure experimental reproducibility. UV-Vis absorbance and fluorescence excitation plus emission measurements were conducted and analysed using a Clariostar plate reader and MARS data analysis software. All data was fitted using EK binding isotherm model using Bindfit v0.5.

Mass spectrometry: Ammonium Acetate (10.00 mM, Supleco Lichropur, Lot AM 1890034413) was prepared in H_2O/5 % EtOH solution (Fischer Chemicals, Lot 2361141): methanol (Sigma-Aldrich, Lot STBK7695). The Quaternary Solvent Manager (QSM) was configured to deliver 0.02 mL/min using direct injection. The Flow Through Needle (FTN) delivered 10 uL per injection and samples were analysed in triplicate. The parameters for the mass spectrometry are as follows: Source Temperature = 121 °C; Desolvation Temperature = 350 °C; Cone Gas = 49 L/h; Desolvation Gas = 597 L/h; Capillary = 2.54 kV; Mode = negative mode, High Definition MSE; Scan Time = 1.000 secs.

Mass Spectrometry Standards: Lock mass spray consisted of a Leucine Enkephalin (ex. Waters, Lot W27072310) (100 pg/µL) and was prepared according to Waters procedure but adjusted to 100 pg/µL; Mass spec calibration performed using Major Mix (ex. Waters, Lot W07022424) and prepared according to Waters procedure 715005131. Rev A. Sample Preparation: Approximately 1 mg of each SSA was dissolved in 1 mL of methanol (Sigma-Aldrich, Lot STBK7695). This solution was further diluted as 10 µL in 1000 µL H_2O/5 % EtOH solution (Fischer 8 Chemicals, Lot 2361141): methanol (Sigma-Aldrich, Lot STBK7695). Samples directly injected into a flow of 10 mM ammonium acetate in H_2O/5 % methanol at 0.2 mL/min.

Section S3: Chemical synthesis

Compound **1:** Glycine methyl ester hydrochloride (0.69 g, 5.50 mmol), 4-trifluoromethoxy benzene isocyanate (0.76 mL, 5.00 mmol) and anhydrous triethylamine (0.83 mL, 6.03 mmol) were stirred overnight at room temperature in dichloromethane (30 mL) to yield a white solid. The resultant precipitate (0.82 g, 2.81 mmol) was removed by filtration, and redissolved in methanol (6.00 mL). Water (4.00 mL) and an aqueous solution of NaOH (3.08 mL, 2.00 M) were then added and left to stir for 3 hours at room temperature. HCl (2.00 M) was then added dropwise until pH = 5.00. The resultant precipitate (2.09 g, 7.51 mmol) was then filtered, washed with ethyl acetate (30 mL) and water (30 mL), re-dissolved in methanol (5.00 mL) and sonicated for 30 minutes with one equivalence of tetrabutylammonium hydroxide in methanol (7.51 mL, 1.00 M). The solution was then taken to dryness under reduced pressure to give a white solid. This white solid was then recrystallised in ethyl acetate (30 mL) and washed with water (30 mL) to give the pure product as a white solid (2.89 g, 5.56 mmol, 74 %). Melting point: 347 K; ^{1}H NMR (400 MHz, 298 K, DMSO-d_6): δ: 0.91 (t, *J* = 7.2 Hz, 12H), 1.23-1.35 (m, 8H), 1.49-1.60 (m, 8H), 3.15 (t, *J* = 8.0 Hz, 8H), 3.43 (d, *J* = 2.3* Hz, 2H), 6.98 (s, 1H), 7.16 (d, *J* = 8.5 Hz, 2H), 7.64 (d, *J* = 8.8 Hz, 2H), 10.37 (s, 1H); ^{13}C {^{1}H} NMR (100 MHz, 298 K, DMSO-d_6): δ: 13.9 (CH_3), 19.7 (CH_2), 23.5 (CH_2), 45.0 (CH_2), 49.0 (CH_2), 57.9 (s, ArC), 121.8 (q, *J* = 19.6 Hz, CF_3), 141.6 (ArC), 141.7 (d, ArCH), 155.6 (C=O), 171.7 (C=O); IR (film): ν_{max} (cm^{-1}) = 3331 (NH stretch) 1695, 1487, 1257, 1153, 877; HRMS for the carboxylate-urea ion **1** ($C_{10}H_8F_3N_2O_4$) (ESI$^-$): m/z measured = 277.0428 [M]$^-$, predicted = 277.0442 [M]$^-$. **J*-coupling value is difficult to calculate due to overlap with water peak.

Compound **2:** D-Glutamic acid dimethyl ester hydrochloride (1.16 g, 5.50 mmol), 4-trifluoromethoxy benzene isocyanate (0.76 mL, 5.00 mmol) and anhydrous triethylamine (0.83 mL, 6.03 mmol) were stirred overnight at room temperature in dichloromethane (30 mL) to yield a pinkish-grey waxy solid. The resultant precipitate (1.54 g, 4.07 mmol) was removed by filtration, and redissolved in methanol (6.00 mL). Water (4.00 mL) and an aqueous solution of NaOH (4.46 mL, 2.00 M) were then added and left to stir for 3 hours at room temperature. HCl (2.00 M) was then added dropwise until pH = 5.00. The resultant precipitate (0.80 g, 2.28 mmol) was then filtered, washed with ethyl acetate (30 mL) and water (30 mL), re-dissolved in methanol (5.00 mL) and sonicated for 30 minutes with two equivalences of tetrabutylammonium hydroxide in methanol (4.56 mL, 1.00 M). The solution was then taken to dryness under reduced pressure to give a brownish-white sticky solid (1.37 g, 1.66 mmol, 60 %). Melting point: 350 K; ^{1}H NMR (400 MHz, 343 K, DMSO-d_6): δ: 0.95 (br s, 12H), 1.34 (br s, 8H), 1.61 (br s, 8H), 1.77 (s, 1H), 1.99 (s, 1H), 2.15 (br s, 1H), 3.20 (br s, 8H), 3.76 (s, 1H), 6.28 (s, 1H), 7.05 (s, 2H), 7.76 (s, 2H), 10.98 (s, 1H); ^{13}C {^{1}H} NMR (100 MHz, 343 K, DMSO-d_6): δ: 13.7 (CH_3), 19.7 (CH_2), 23.7 (CH_2), 35.6 (CH_2), 49.0 (CH), 55.8 (CH_2), 58.5 (CH_2), 119.5 (q, *J* = 104 Hz, CF_3), 142.0 (ArCH), 142.2 (ArC), 156.1 (ArC), 174.2 (C=O), 176.9 (C=O); IR (film): ν_{max} (cm^{-1}) = 3334 (NH stretch), 2962, 1697, 1253, 1047, 844; HRMS for the carboxylate-urea ion **2** ($C_{13}H_{11}F_3N_2O_6$) (ESI$^-$): m/z measured = 348.0596 [M]$^-$, predicted = 348.0580 [M]$^-$.

Compound **3**: (R)-3-(1-Naphthyl)-D-alanine (1.18 g, 5.50 mmol) was dissolved in methanol (5.00 mL) and sonicated for 10 minutes with one equivalence of tetrabutylammonium hydroxide in methanol (5.50 mL, 1.00 M) producing a naphthyl TBA salt which was dried under pressure overnight. This brown-oil was then added to a solution of 4-trifluoromethoxy benzene isocyanate (0.76 mL, 5.00 mmol) and anhydrous triethylamine (0.83 mL, 6.03 mmol), then stirred overnight at room temperature in dichloromethane (30 mL) to yield an orange-brown solid. The resultant mixture was taken to dryness under reduced pressure. The crude product was purified using silica flash chromatography (100 % ethyl acetate followed by 100 % methanol). The resulting brownish-gold glassy product was dried under reduced pressure overnight (3.00 g, 4.30 mmol, 86 %). Melting point:

347 K; ^{1}H NMR (400 MHz, 343 K, DMSO-d_6): δ: 0.91 (t, J = 7.2 Hz , 12H), 1.23-1.34 (m, 8H), 1.48-1.59 (m, 8H), 3.13 (t, J = 8.4 Hz, 8H), 3.34-3.41 (m, 2H), 4.31 (s, 1H), 7.10-8.02 (m, 11H), 8.42 (d, J = 8.0 Hz, 1H), 10.24 (s, 1H); ^{13}C {^{1}H} NMR (100 MHz, 343 K, DMSO-d_6): δ: 13.5 (CH_3), 19.2 (CH_2), 23.1 (CH_2), 56.0 (CH), 57.5 (CH_2), 118.3 (q, J = 70 Hz, CF_3), 124.4 (ArCH), 125.2 (ArCH), 125.3 (ArCH), 125.5 (ArCH), 127.5 (ArC), 128.3 (ArC), 132.3 (ArC), 133.3 (ArC), 135.8 (ArC), 140.9 (ArC), 141.3 (d, J = 1.8 Hz, ArCH), 155.2 (C=O), 173.9 (C=O); IR (film): ν_{max} (cm^{-1}) = 3330 (NH-stretch), 2962, 1685, 1193, 1016, 777; HRMS for the carboxylate-urea ion ($C_{21}H_{16}N_3O_3$) (ESI$^-$): m/z measured = 417.1065 [M]$^-$, predicted = 417.1068 [M]$^-$.

Compound **4**: Glycine methyl ester hydrochloride (0.99 g, 13.10 mmol) was dissolved in methanol (10.00 mL) and tetrabutylammonium hydroxide in methanol (14.00 mL, 1.00 M) added. The sample was dried for 24 hours under reduced pressure to give a clear oil (4.12 g, 13.05 mmol). The tetrabutylammonium salt was then dissolved in ethyl acetate (30.00 mL) and an inert atmosphere was then applied to it. This brown-oil was then added to a solution of 4-butylphenylisocyonate (2.12 mL, 12.00 mmol) in dichloromethane (30.00 mL) dropwise and the mixture was then heated to 60 °C. After 48 hours the solvent was removed under reduced pressure to give a crude product as a white solid (6.41 g, 13.48 mmol). Purification by silica gel chromatography (100 % ethyl acetate followed by 100 % methanol) afforded the pure product as a white precipitate (1.86 g, 3.91 mmol, 28 %). Melting point 396 K; ^{1}H NMR (400 MHz, 298 K, DMSO-d_6): δ: 0.89 (t, J = 7.4 Hz, 3H), 0.94 (t, J = 7.4 Hz, 12H), 1.25-1.38 (m, 10H), 1.45-1.64 (m, 10H), 2.46 (t, J = 7.7 Hz, 2H), 3.17 (t, J = 8.5 Hz, 8H), 3.26 (d, J = 3.7 Hz, 2H), 6.27 (d, J = 2.9 Hz, 1H), 6.98 (d, J = 8.4 Hz, 2H), 7.32 (d, J = 8.4 Hz, 2H), 9.18 (s, 1H); ^{13}C{^{1}H} NMR (100 MHz, 298 K, DMSO-d_6): δ: 13.5 (CH_3), 13.9 (CH_3), 19.2 (CH_2), 21.8 (CH_2), 23.1 (CH_2), 33.5 (CH_2), 34.2 (CH_2), 45.1 (CH_2), 57.5 (CH_2), 117.2 (ArCH), 128.2 (ArCH), 133.7 (ArC), 139.3 (ArC), 155.2 (C=O), 170.3 (C=O); IR (film): ν_{max} (cm^{-1}) = 3438, 3331 (NH stretch), 2958, 2928, 2873, 1691, 1599, 1545, 1415, 1488; HRMS for the carboxylate-urea ion **4** ($C_{13}H_{17}N_2O_3$) (ESI$^-$): m/z measured = 249.1229 [M]$^-$, predicted = 249.1244 [M]$^-$.

Compound **5**: *D*-Glutamic acid (1.93 g, 13.10 mmol) was dissolved in methanol (10.00 mL) and tetrabutylammonium hydroxide in methanol (28.00 mL, 1.00 M) added. The solvent was removed under reduced pressure and further dried for 24 hours under reduced pressure to give a yellow oil (8.10 g, 12.86 mmol). The tetrabutylammonium salt was then dissolved in anhydrous dichloromethane (30.00 mL). Once stirring, 4-butylphenylisocyonate (2.12 mL, 12.00 mmol) was added dropwise, and the mixture taken to 60 °C temperature. After 48 hours the solvent was removed under reduced pressure. The crude product was then dissolved in chloroform (30.00 mL) and then flooded with hexane to induce crystallisation of the partially pure product as a sticky yellow oil. Purification by column chromatography on silica gel using a gradient of hexane/ethyl acetate (10–40% hexane) afforded the pure compound as a sticky oil of a deep orange colour (0.95 g, 1.18 mmol, 10 %). Melting point - 340 K; ^{1}H NMR (400 MHz, 298 K, CD_3OD): δ: 0.89 (t, J = 7.3 Hz, 3H), 0.95 (*t*, J = 7.4 Hz, 24H), 1.27-1.41 (m, 18H), 1.48-1.66 (m, 20H), 1.88-2.16 (m, 3H), 2.47 (d, J = 7.68 Hz, 1H), 3.16-3.22 (m, 18H), 5.96 (s, 1H), 6.94 (d, J = 8.3 Hz, 2H), 7.50 (d, J = 7.2 Hz, 2H), 10.35 (s, 1H); ^{13}C{^{1}H} NMR (100 MHz, 298 K, DMSO-d_6): δ: 4.8 (CH_3), 11.2 (CH_3), 13.8 (CH_3), 15.4 (CH_3), 20.1 (CH_3), 20.8 (CH_3), 21.0 (CH_3), 21.2 (CH_3), 22.7 (CH_2), 25.6 (CH_2), 26.0 (CH_2), 26.5 (CH_2), 47.1 (CH_2), 49.9 (CH), 110.7 (ArCH), 120.0 (ArCH), 128.0 (ArCH), 129.6 (ArCH), 148.2 (C), 170.0 (C=O), 172.6 (C=O); IR (film): ν_{max} (cm^{-1}) = 3312 (NH stretch), 2959, 2928, 2872, 1707, 1684, 1600, 1549, 1390, 1230; HRMS for the carboxylate-urea ion of **5** ($C_{16}H_{20}N_2O_5$) (ESI$^-$): m/z measured = 319.1297 [M]$^-$, predicted = 319.1289 [M]$^-$.

Compound **6**: (R)-3-(1-Naphthyl)-D-alanine (0.90 g, 4.20 mmol) was dissolved in methanol (10.00 mL) and tetrabutylammonium hydroxide in methanol (5.00 mL, 1.00 M) added. The solvent was removed under reduced pressure. The sample was further dried for 24 hours under high vacuum. The

tetrabutylammonium salt was then dissolved in ethyl acetate (30.00 mL) and an inert atmosphere applied prior to adding 4-butylphenylisocyonate dropwise to the mixture. The mixture was then heated to 60 °C. After 48 hours the solvent was removed under reduced pressure and the sample was further dried for 24 hours under high vacuum giving a pale-yellow sticky precipitate as a pure product (2.20 g, 2.69 mmol, 87 %). Melting point: 351 K; ^{1}H NMR (400 MHz, 333 K, DMSO-*d$_6$*): δ: 0.86-0.98 (m, 15H), 1.23-1.37 (m, 10H), 1.46-1.63 (m, 10H), 2.43-2.49 (m, 2H), 3.17 (t, *J* = 8.6 Hz, 8H), 3.25 - 3.32 (m, 1H), 3.47-3.54 (m, 1H), 4.07 (s, 1H), 6.97 (d, *J* = 8.4 Hz), 7.23 - 7.46 (m, 4H), 7.68 (d, *J* = 7.72 Hz, 2H), 7.82 - 7.86 (m, 1H), 7.78-7.92 (m, 1H), 8.38 (t, *J* = 3.8 Hz, 1H), 8.91 (*s*, 1H); ^{13}C{^{1}H} NMR (100 MHz, 298 K, DMSO-*d$_6$*): δ: 13.5 (CH_3), 13.9 (CH_3), 19.3 (CH_2), 21.7 (CH_2), 23.1 (CH_2), 33.5 (CH_2), 34.2 (CH_2), 37.0 (CH_2), 56.5 (CH), 57.5 (CH), 117.4 (ArCH), 124.8 (ArCH), 125.1 (ArCH), 125.3 (ArCH), 125.9 (ArCH), 127.5 (ArCH), 128.1 (ArCH), 132.7 (ArC), 133.30 (ArC), 133.6 (ArC), 136.6 (ArC), 139.3 (ArC), 155.4 (C=O), 172.9 (C=O); IR (film): ν_{max} (cm^{-1}) = 3298 (NH stretch), 3042, 2957, 2929, 2872, 1686, 1603, 1541, 1379, 1233; HRMS for carboxylate ion of **6** ($C_{24}H_{25}N_2O_3$) (ESI$^-$): m/z measured = 388.1798 [M]$^-$, predicted = 388.1781 [M]$^-$.

Compound **7:** (R)-3-(1-Naphthyl)-D-alanine (1.18 g, 5.50 mmol) was dissolved in methanol (5.00 mL) and sonicated for 10 minutes with one equivalent of tetrabutylammonium hydroxide in methanol (5.50 mL, 1.00 M) producing a naphthyl-tetrabutylammonium salt which was then dried under reduced pressure overnight. This brownish-gold oil was then added to a solution of 4-cyanophenyl isocyanate (0.721 g, 5.00 mmol) and anhydrous triethylamine (0.83 mL, 6.03 mmol), then stirred overnight at room temperature in dichloromethane to yield an orange-brown solid. The resultant precipitate was taken to dryness under reduced pressure. The crude product was purified using silica flash chromatography (ethyl acetate followed by methanol. The resulting golden glassy product was dried under reduced pressure overnight (2.57 g, 4.28 mmol, 78 %). Melting Point: 350 K; ^{1}H NMR (400 MHz, 298 K, DMSO-*d$_6$*): δ: 0.91 (t, *J* = 7.2 Hz, 12H), 1.23-1.33 (m, 8H), 1.48-1.58 (m, 8H), 3.13 (t, *J* = 8.1 Hz, 8H), 3.41 (d, *J* = 4.8 Hz, 2H), 4.28 (s, 1H), 7.29-7.89 (m, 11H), 8.45 (d, *J* = 7.9 Hz, 1H), 10.96 (s, 1H); ^{13}C{^{1}H} NMR (100 MHz, 298 K, DMSO-*d6*): δ: 13.5 (CH_3), 19.2 (CH_2), 23.0 (CH_2), 56.4 (CH), 57.5 (CH_2), 101.0 (ArCH), 117.3 (ArCH), 119.8 (CH), 124.4 (ArCH), 125.33 (*t, J* = 16.2 Hz, CH_2), 126.2 (ArCH), 127.4 (ArC), 128.3 (ArC), 132.4 (ArC), 132.8 (ArC), 133.3 (ArC), 135.8 (ArC), 146.2 (CN), 154.8 (C=O), 173.7 (C=O); IR (film): ν_{max} (cm^{-1}) = 3284 (NH stretch), 2214, 1593, 1171, 842; HRMS for the carboxylate-urea ion **7** ($C_{21}H_{16}N_3O_3$) (ESI$^-$): m/z measured = 358.1186 [M]$^-$, predicted = 358.1187 [M]$^-$.

Compound **8**: (R)-3-(1-Naphthyl)-D-alanine (1.18 g, 5.50 mmol) was dissolved in methanol (5.00 mL) and sonicated for 10 minutes with one equivalence of tetrabutylammonium hydroxide (5.50 mL, 5.50 mmol), producing a naphthyl-tetrabutylammonium salt which was dried under reduced pressure overnight. This brownish-gold salt-oil was then added to a solution of 5-cyanophenyl isocyanate (0.72 g, 5.00 mmol) and anhydrous triethylamine (0.83 mL, 6.03 mmol), then stirred overnight at room temperature in dichloromethane to yield a dark orange-brown solid. The resultant precipitate was taken to dryness under reduced pressure. The crude product was purified using silica flash chromatography (ethyl acetate followed by methanol. The resulting brown glassy product was dried under reduced pressure overnight (2.15 g, 3.58 mmol, 65 %). Melting Point: 350 K; ^{1}H NMR (400 MHz, 298 K, DMSO-*d$_6$*): δ: 0.91 (t, *J* = 7.6 Hz, 12H), 1.23-1.34 (m, 8H), 1.49-1.59 (m, 8H), 3.09-3.17 (m, 8H), 3.41 (t, *J* = 7.6 Hz, 2H), 4.31 (s, 1H), 7.13-8.13 (m, 11H), 8.46 (d, *J* = 8.2 Hz, 1H), 10.80 (s, 1H); ^{13}C{^{1}H} NMR (100 MHz, 298 K, DMSO-*d6*): δ: 13.5 (CH_3), 19.2 (CH_2), 23.0 (CH_2), 56.4 (CH), 57.5 (CH_2), 111.1 (ArC), 119.2 (ArC), 119.6 (CH), 122.0 (ArCH), 123.3 (ArCH), 124.4 (ArCH), 125.30 (t, *J* = 19.8 Hz, CH_2), 126.2 (ArCH), 127.4 (ArCH), 128.3 (ArCH), 129.6 (ArC), 132.3 (ArC), 133.3 (ArC),135.8 (ArC), 142.6 (CN), 155.1 (C=O), 174.0 (C=O); IR (film): ν_{max} (cm^{-1}) = 3275 (NH stretch), 2224, 1585, 1232, 777; HRMS for the carboxylate-urea ion **8** ($C_{21}H_{16}N_3O_3$) (ESI$^-$): m/z measured = 358.1187 [M]$^-$, predicted = 358.1197 [M]$^-$.

Compound **9**: (R)-3-(1-Naphthyl)-D-alanine (1.07 g, 5.00 mmol) was dissolved in methanol (5.00 mL) and sonicated for 10 minutes with one equivalent of tetrabutylammonium hydroxide in methanol (5.50 mL, 1.00 M) producing a naphthyl tetrabutylammonium salt which was dried under reduced pressure overnight. In a separate round bottom, triphosgene (0.445 g, 1.5 mmol) was added to a stirring solution of 4-aminophenylsulphur pentafluoride (1.10 g, 5.00 mM), anhydrous triethylamine (1.25 mL, 12.32 mmol) and dichloromethane (30 mL), and the mixture heated at reflux for 4 hours. The naphthyl tetrabutylammonium salt was then added to the solution and anhydrous triethylamine (0.83 mL, 6.03 mmol) was topped up, then stirred overnight at room temperature to yield a cream white solid. The resultant precipitate was taken to dryness under reduced pressure. The crude product was purified using silica flash chromatography(ethyl acetate followed by methanol). The resulting cream white product was dried under vacuum overnight (1.75 g, 2.49 mmol, 50 %). Melting Point: 355 K; ^{1}H NMR (400 MHz, 298 K, DMSO-d_6): δ: 0.91 (*t*, *J* = 7.3 Hz, 12H), 1.23-1.34 (m, 8H), 1.48-1.58 (m, 8H), 3.13 (t, *J* = 7.9 Hz, 8H), 3.35 (s, 2H), 4.19 (s, 1H), 7.29-7.89 (m, 11H), 8.44 (d, *J* = 7.9 Hz, 1H), 10.94 (s, 1H); ^{13}C{^{1}H} NMR (100 MHz, 298 K, DMSO-d_6): δ: 13.4 (CH_3), 19.2 (CH_2), 23.0 (CH_2), 56.5 (CH), 57.5 (CH_2), 116.3 (CH), 124.6-126.2 (m, C-SF_5), 127.4 (ArCH), 128.2 (ArCH), 132.5 (ArCH), 133.3 (ArCH), 136.1 (ArCH), 144.3-144.6 (m, CH_2), 145.2 (C=O), 154.8 (C=O); IR (film): ν_{max} (cm^{-1}) = 3290 (NH stretch), 2960, 1485, 1097, 833, HRMS for the carboxylate-urea ion **9** ($C_{20}H_{16}F_5N_2O_3S$) (ESI$^-$): m/z measured = 459.0801 [M]$^-$, predicted = 459.0807 [M]$^-$.

Compound **10**: (R)-3-(1-Naphthyl)-D-alanine (0.90 g, 4.20 mmol) and tetrabutylammonium hydroxide in methanol (4.20 mL, 1.00 M) was dissolved in methanol (10.00 mL), heated, sonicated, and dried under reduced pressure. 4-(6-Methyl-1,3-benzothiazol-2-yl)phenylamine (1.92 g, 8.00 mmol) was dissolved in ethyl acetate (30.00 mL) and together with triethylamine (1.00 mL, 7.20 mmol) added to the mixture. Triphosgene (1.18 g, 4.00 mmol) was added to the solution and refluxed for 4 hours. (R)-2-Amino-3-(naphthalen-2-yl)propanoate tetrabutylammonium salt was added to the reaction with acetonitrile (10.00 mL) and reacted overnight. The resulting crude yielded a sticky brown precipitate, the solvent was decanted and taken to dryness. Redissolving in dichloromethane (30.00 mL) and extracting with deionised water (30.00 mL x2), the crude formed a precipitate. The precipitate was resuspended in methanol and refiltered under reduced pressure. Hydantoin hydrolysis was performed with methanol: isopropyl alcohol (5.00 mL: 5.00 mL) and heating to 50° C. Sodium hydroxide (4.00 mL, 4.00 mmol) was added and reacted for 16 hours at room temperature. Subsequently, a pH = 1.00 was attained with hydrochloric acid (2.00 M) before flooding with deionised water and filtering under reduced pressure. The pure product was a light brown solid (1.40 g, 1.9 mmol, 24 %). Melting point: > 473 K; ^{1}H NMR (400 MHz, 298 K, DMSO-d_6): δ 0.89 (t, *J* = 7.4 Hz, 12H), 1.23-1.32 (m, 8H), 1.47-1.55 (m, 8H), 2.45 (s, 3H), 3.10 (t, *J* = 8.4 Hz, 8H), 3.45-3.55 (m, 2H), 4.24-4.27 (q, *J* = 7.6 Hz, 1H), 7.30-7.53 (m, 6H), 7.72-7.88 (m, 8H), 8.50 (d, *J* = 8.1 Hz, 1H), 10.72 (s, 1H); ^{13}C{^{1}H} NMR (100 MHz, 298 K, DMSO-d_6) : δ: 13.5 (CH_3), 19.2 (CH_2), 21.0 (CH_3), 23.0 (CH_2), 37.1 (CH_2), 56.53 (CH), 57.5 (CH_2), 117.3 (ArCH), 121.6 (ArCH), 121.8 (ArCH), 124.5 (ArC), 124.7 (ArCH), 125.1 (ArCH), 125.3 (ArCH), 126.0 (ArCH), 127.4 (ArCH), 127.6 (ArCH), 127.8 (ArCH), 128.2 (ArCH), 132.6 (ArC), 133.3 (ArC), 134.5 (ArC), 136.3* (ArC), 144.9 (ArC), 151.9 (ArC), 155.0 (CO), 166.5 (ArC) 173.1 & 173.2* (CO); IR (film): ν_{max} (cm^{-1}) = 3298 (NH Stretch), 3041, 2958, 2872, 1695, 1595, 1479, 1452, 1379, 1319, 1225, 1175; HRMS for the carboxylate-urea ion **10** ($C_{28}H_{22}N_3O_3S$)(ESI$^-$): m/z measured = 480.1414 [M]$^-$, predicted = 480.1419 [M]$^-$. *Conformational isomerism observed in this molecule.

Section S4: Single Crystal XRD data

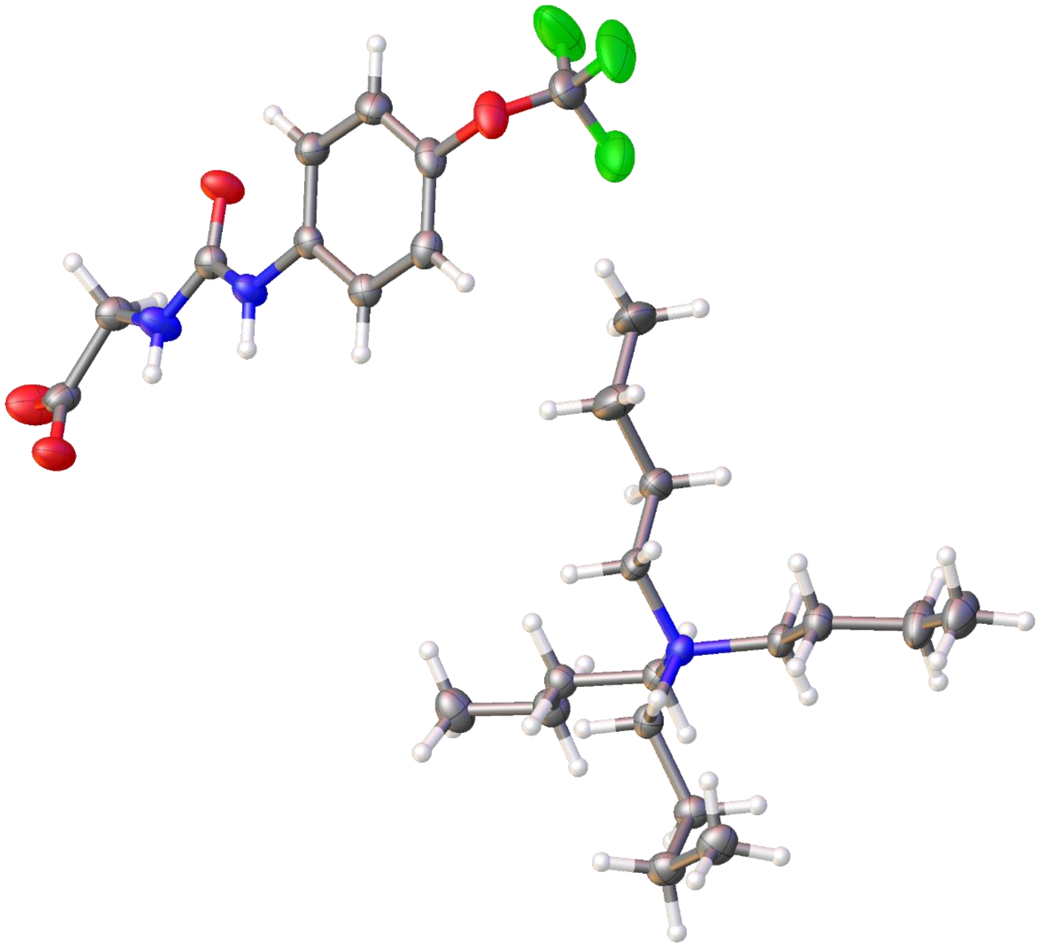

Figure S2 - Single crystal X-ray structure of **1**: red = oxygen; green = fluorine; blue = nitrogen; white = hydrogen; grey = carbon. CCDC 2435071, C26H46F3N3O5 (M = 537.66): triclinic, space group P -1, a = 8.7764(5) Å, b = 9.3855(5) Å, c = 19.5804(10) Å, α = 95.882(4)°, β = 96.538(4)°, γ = 114.489(5)°, V = 1438.10(14) Å3, Z = 2, T = 150(1) K, CuK\α = 1.5418 Å, Dcalc = 1.242 g/cm3, 10001 reflections measured (9.22 ≤ 2Θ ≤ 144.00), 5478 unique (Rint = 0.0435, Rsigma = 0.0550) which were used in all calculations. The final R1 was 0.0479 (I > 2σ(I)) and wR2 was 0.1294 (all data). Interior angle of dimerization = 180.0(17)°.

Table S1 - Hydrogen bond distances and angles observed for **1**, calculated from the single crystal X-ray structure shown in Figure S2.

Hydrogen bond donor	Hydrogen atom	Hydrogen bond acceptor	Hydrogen bond length (D•••A) (Å)	Hydrogen bond angle (D-H•••A) (°)
N1	H1	O2	3.057(2)	139.9(19)
N2	H2	O2	2.729(2)	163.7(19)
O5	H5A	O3	2.7711(19)	169.89(11)
O5	H5B	O1	2.766(2)	170.91(12)

Section S5: NMR Spectroscopy Data

Section S5.1: ^{1}H NMR Spectroscopy Data

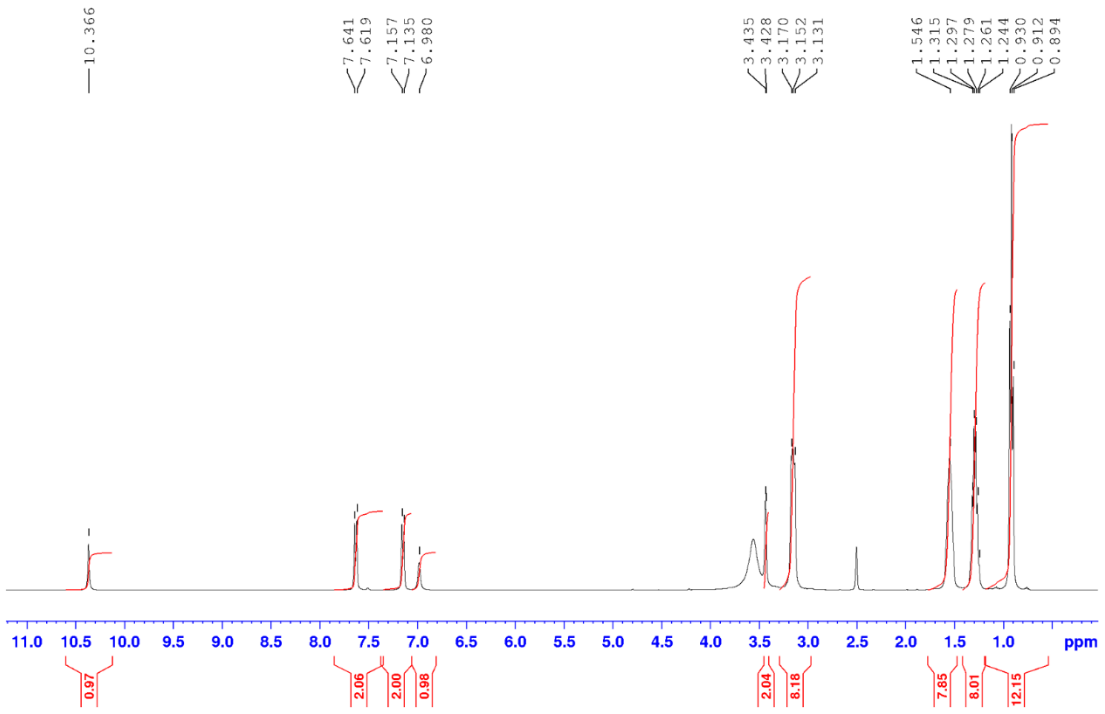

Figure S3 - ^{1}H NMR spectrum of **1** in DMSO-d_6 at 298 K.

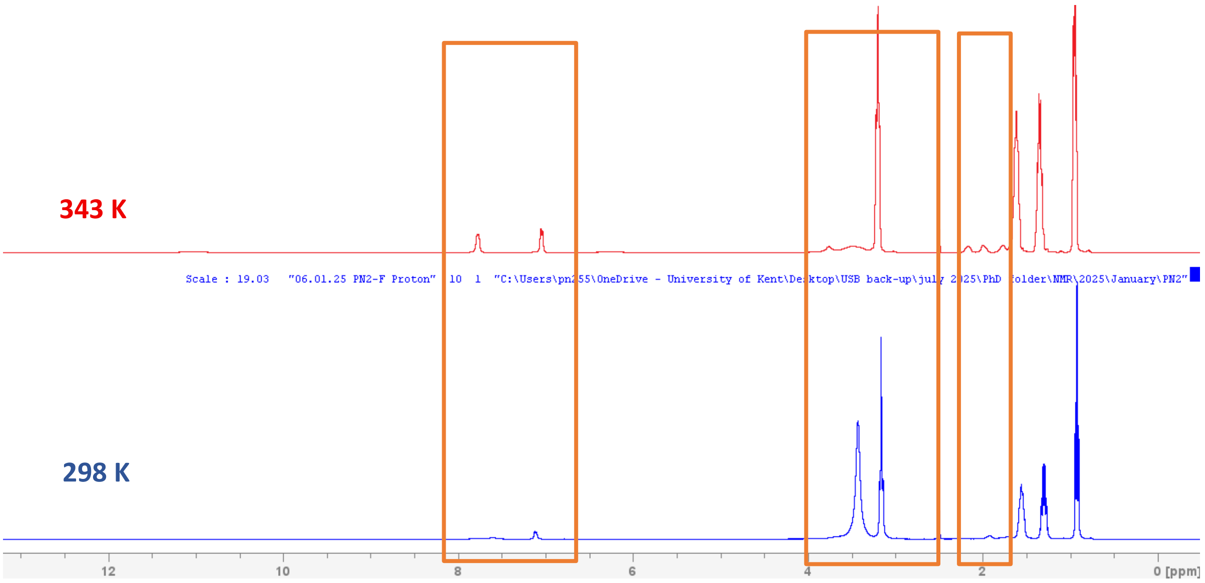

Figure S4 - Variable temperature ^{1}H NMR of **2** in DMSO-d_6, experiments at 298 K, 343 K. Exchangeable NH's (7.00 ppm - 9.00 ppm) as well as the CH_2's up-field (1.65 ppm - 2.35 ppm) become clearly observable at 343 K both highlighted in red boxes. The characterisation spectra can be seen below at a high temperature of 343 K (Figure S5).

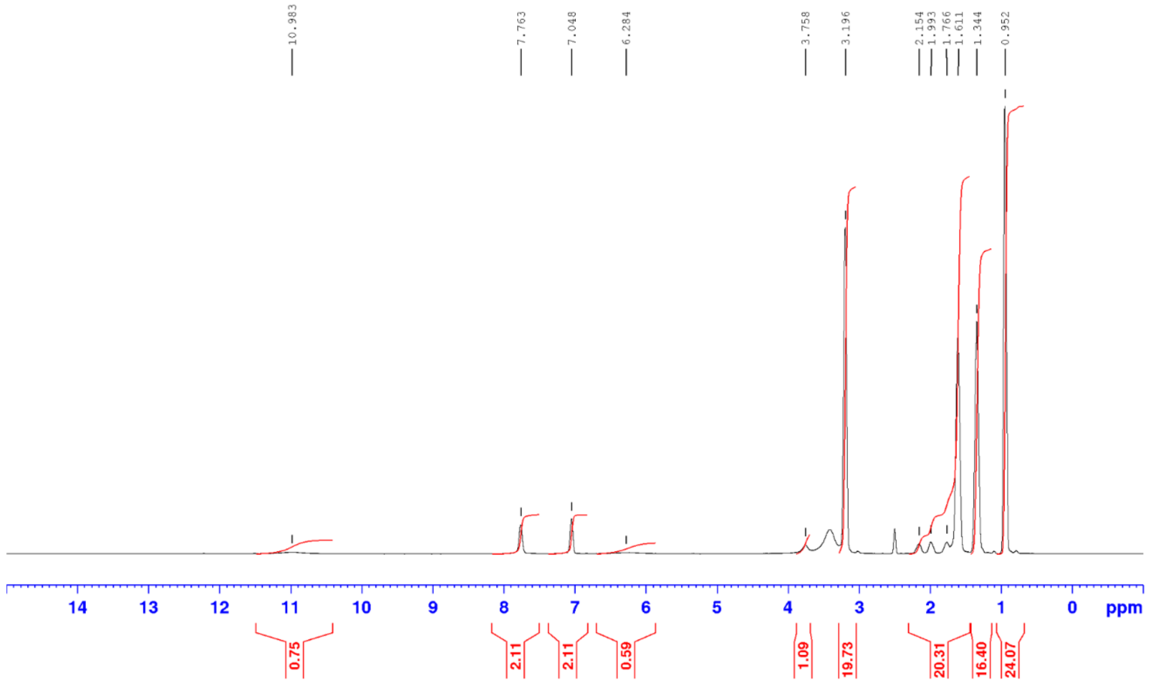

Figure S5 - ^{1}H NMR spectrum of **2** in DMSO-d_6 at 343 K.

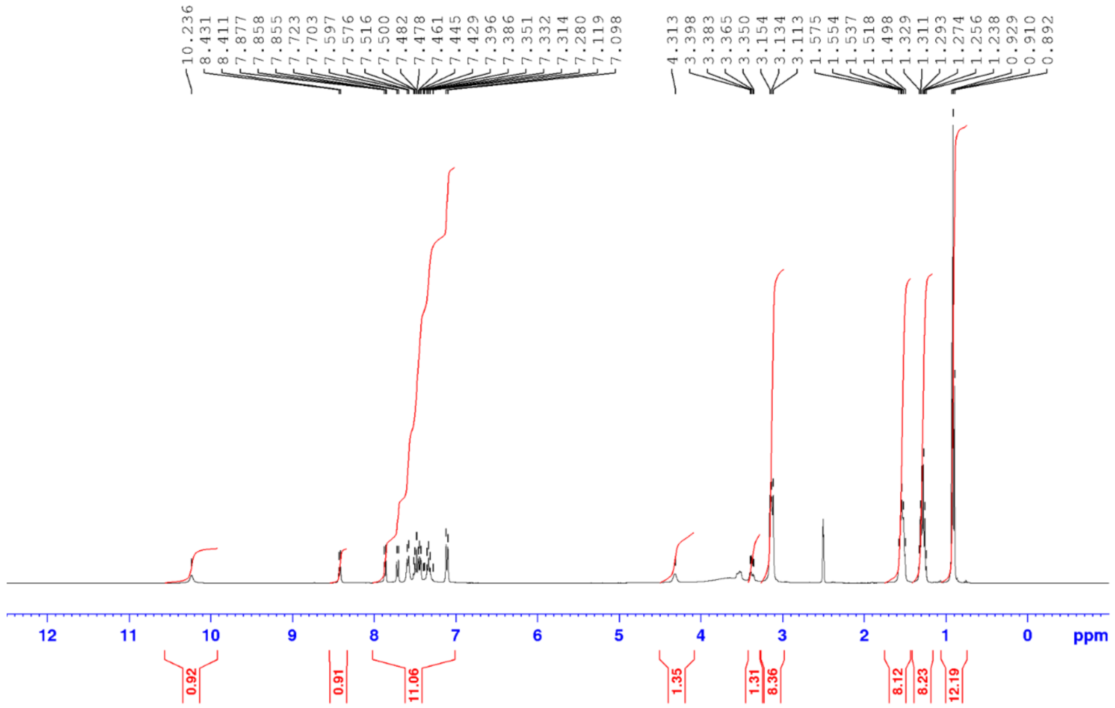

Figure S6 - ^{1}H NMR spectrum of **3** in DMSO-d_6 conducted at 298 K.

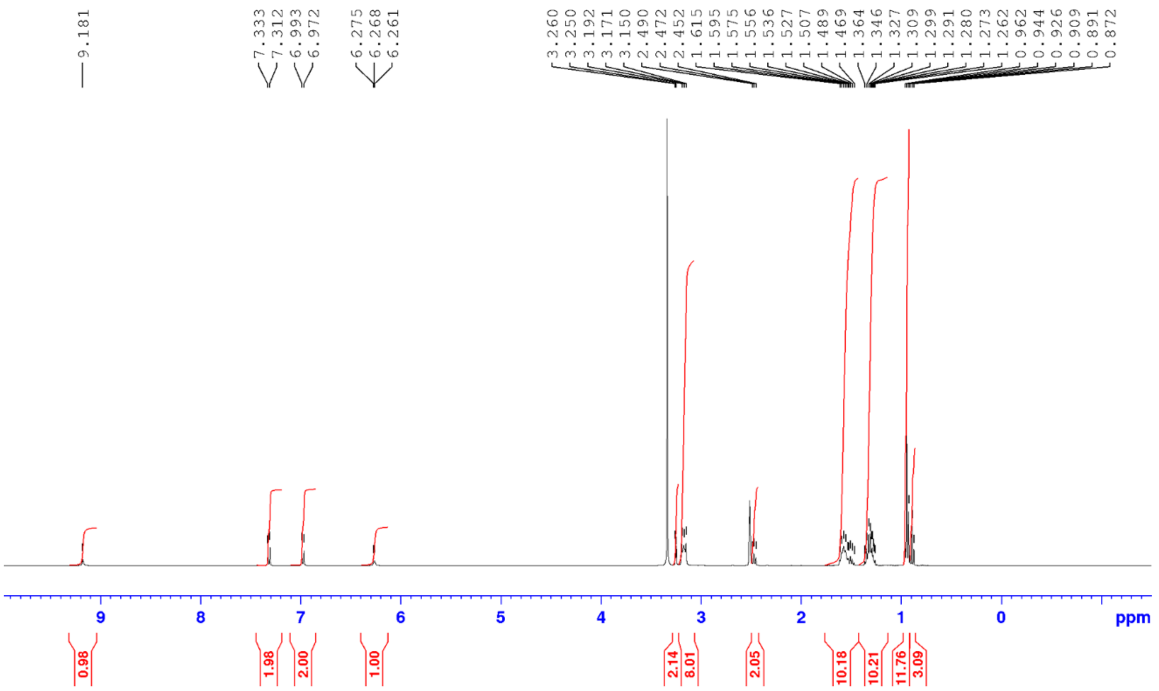

Figure S7 - ^{1}H NMR spectrum of **4** in DMSO-d_6 at 298 K.

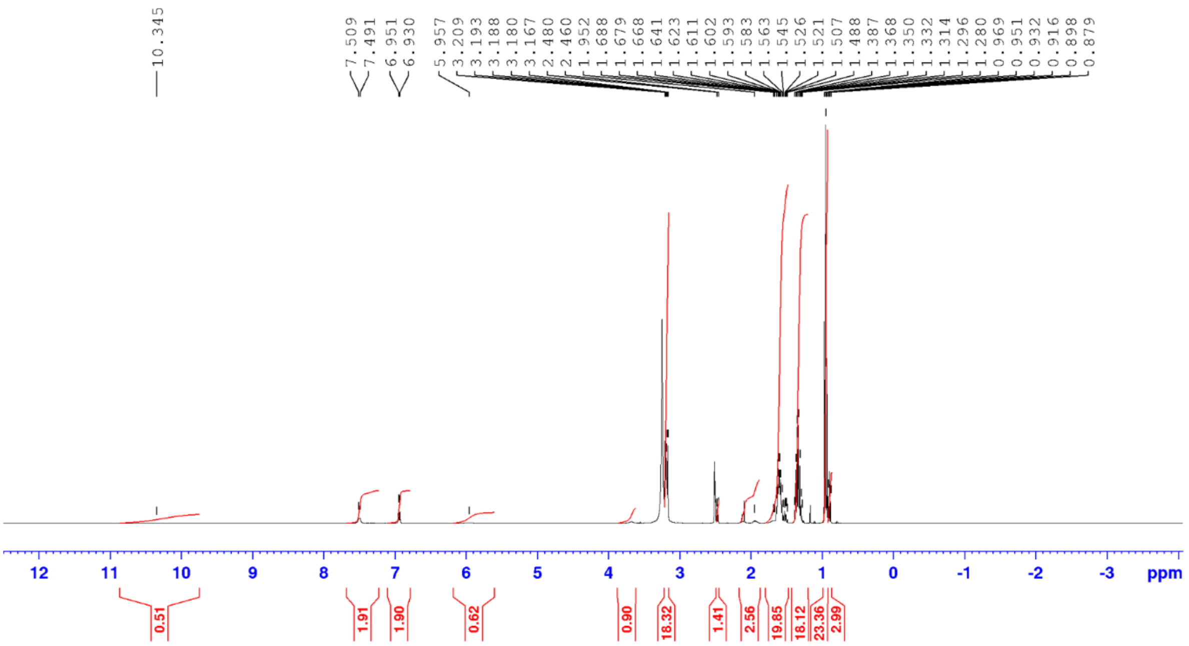

Figure S8 - ^{1}H NMR spectrum of **5** in $CDCl_3$ at 298 K. The broad peaks observed in this spectra are a result of slow exchange, and the VT spectra at 353 K confirm this in Figure S9 below.

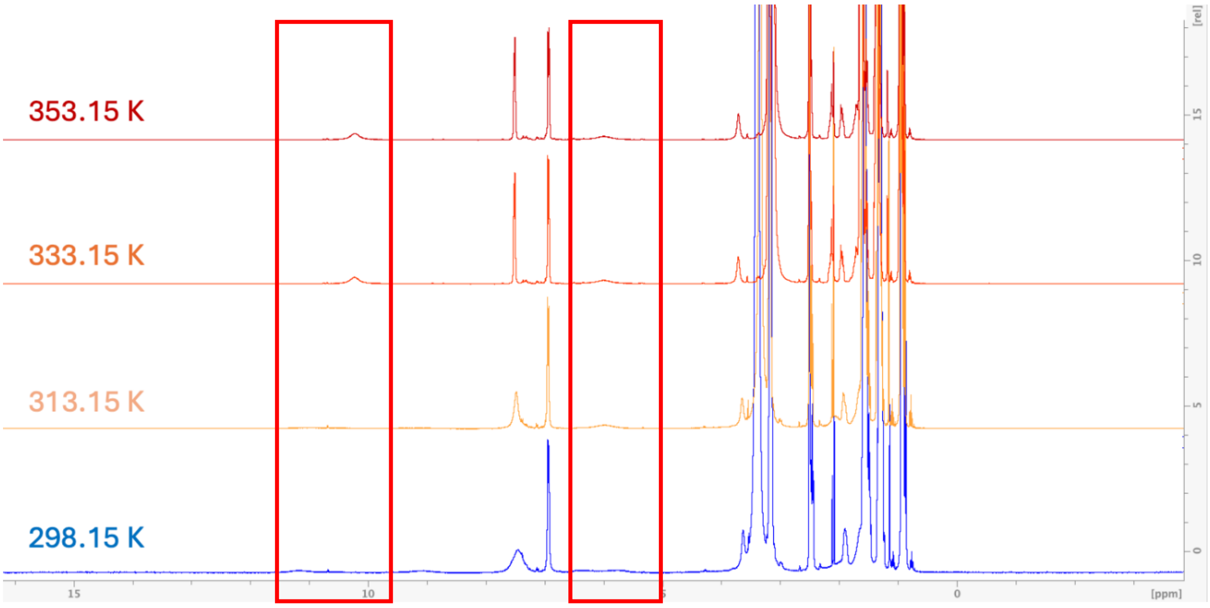

Figure S9 - Variable temperature ^{1}H NMR of **5** in DMSO-d_6, experiments at 298 K, 313 K, 333.15 K and 353.15 K. Exchangeable NH's become clearly observable at 333.15 K (highlighted in red boxes).

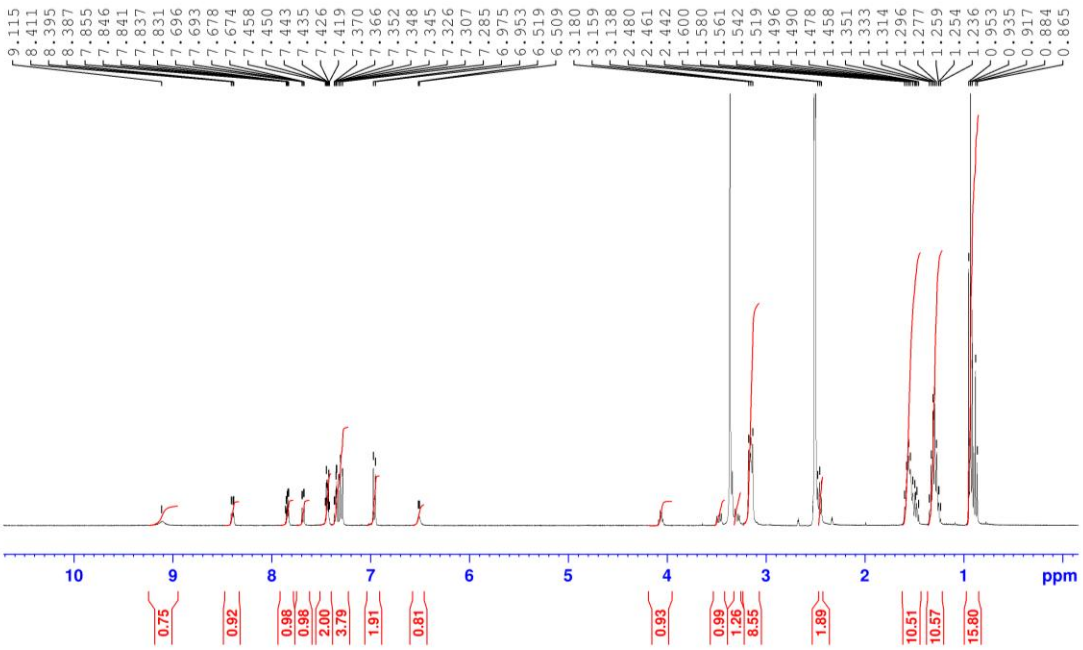

Figure S10 - ^{1}H NMR spectrum of **6** in DMSO-d_6 at 298 K.

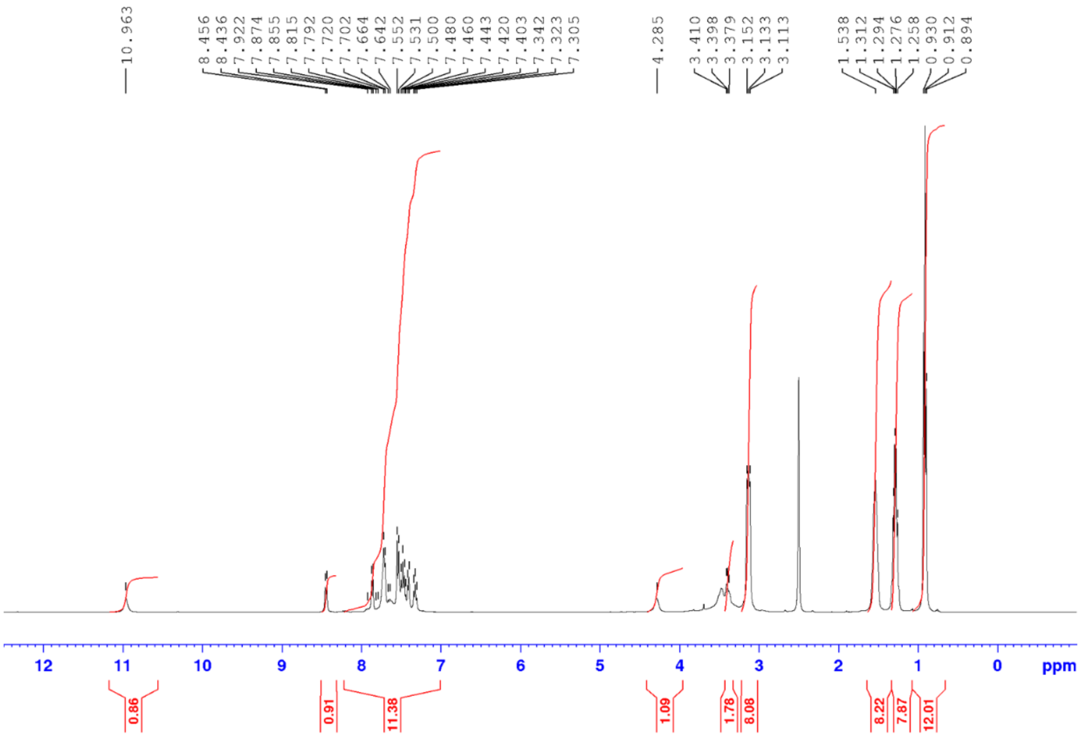

Figure S11 - ^{1}H NMR spectrum of **7** in DMSO-d_6 at 298 K.

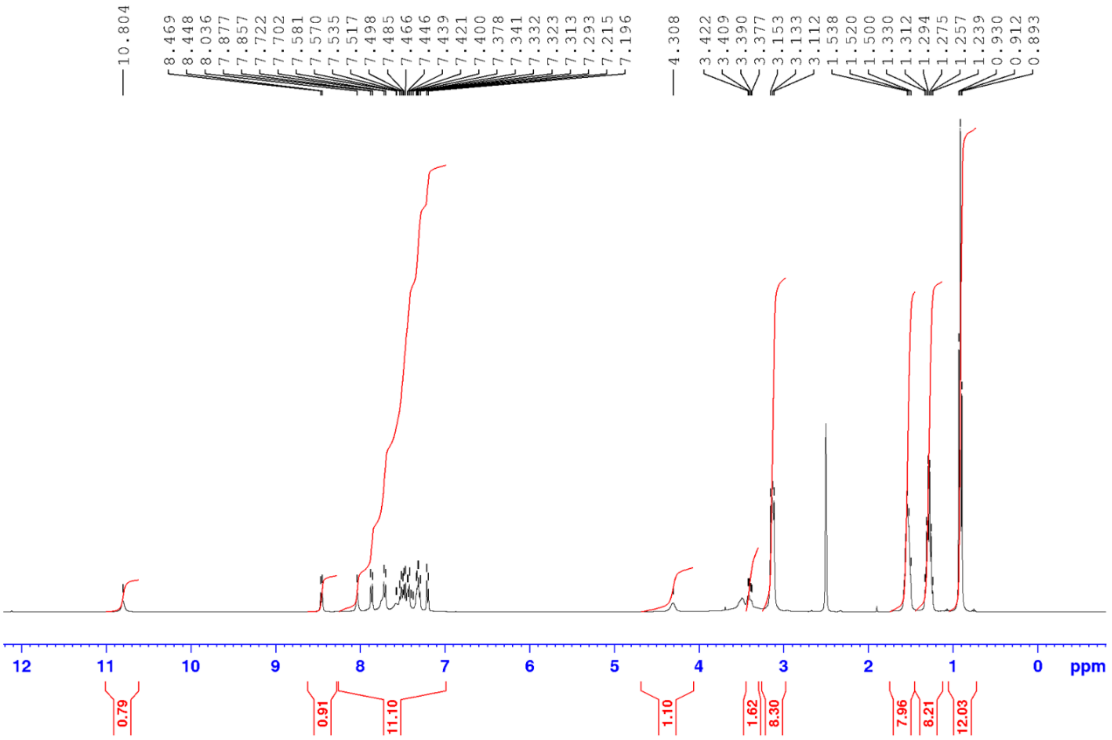

Figure S12 - ^{1}H NMR spectrum of **8** in DMSO-d_6 at 298 K.

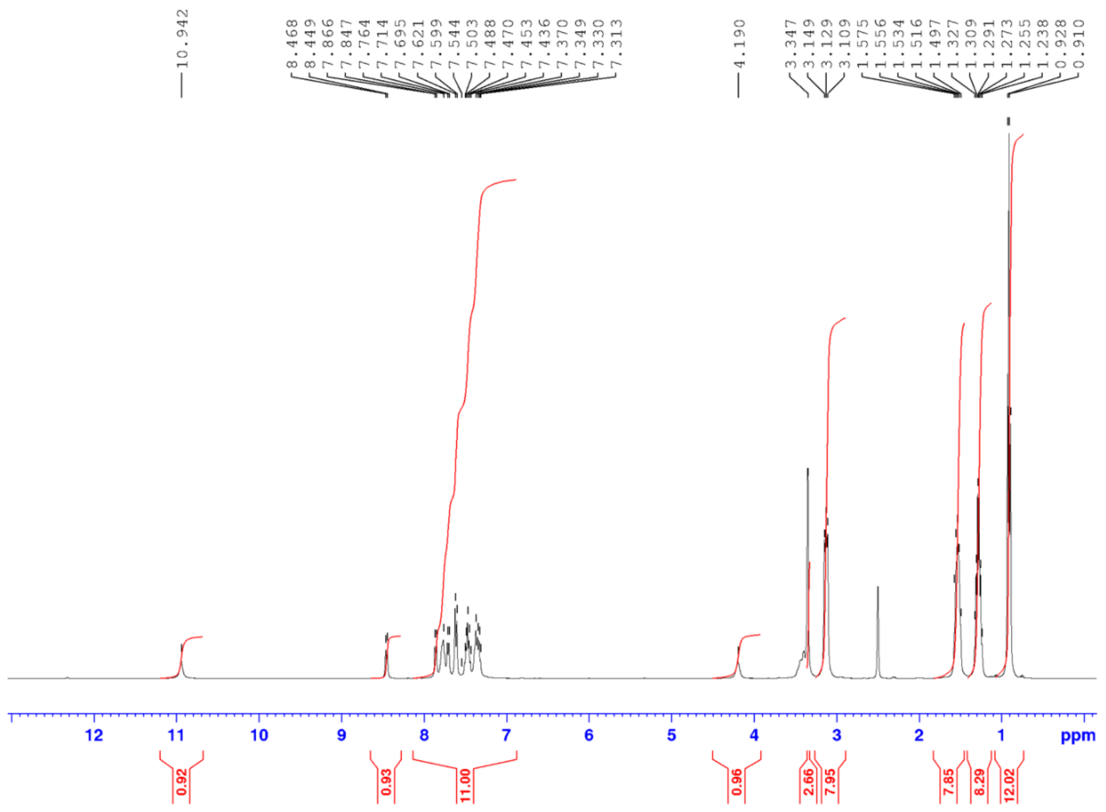

Figure S13 - ^{1}H NMR spectrum of **9** in DMSO-d_6 at 298 K.

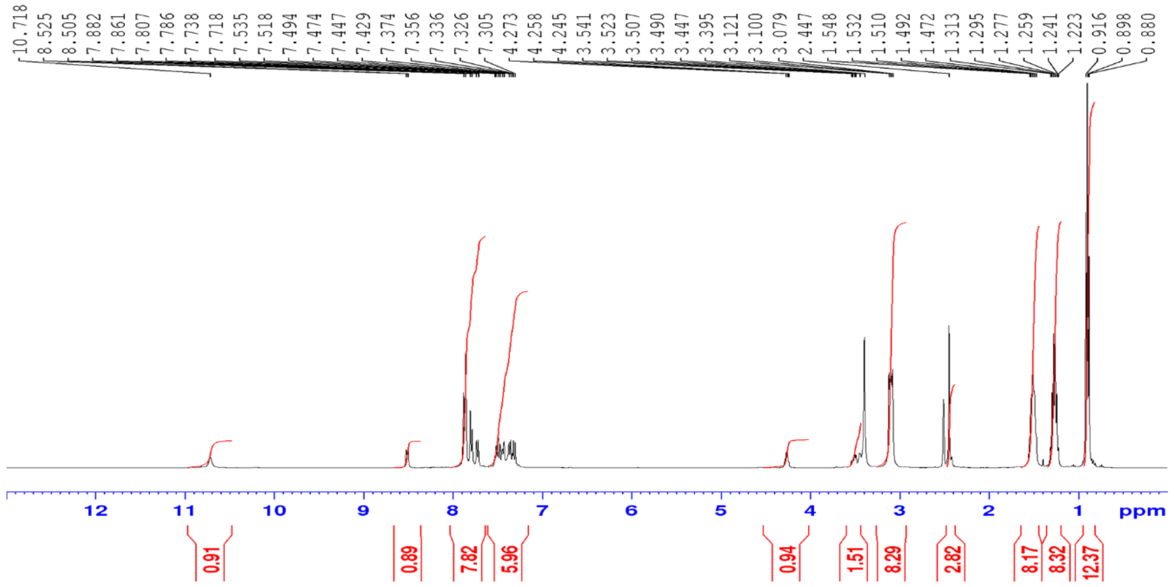

Figure S14 - ^{1}H NMR spectrum of **10** in DMSO-d_6 at 298 K.

Section S5.2: $^{13}C\{^{1}H\}$ NMR spectroscopy data

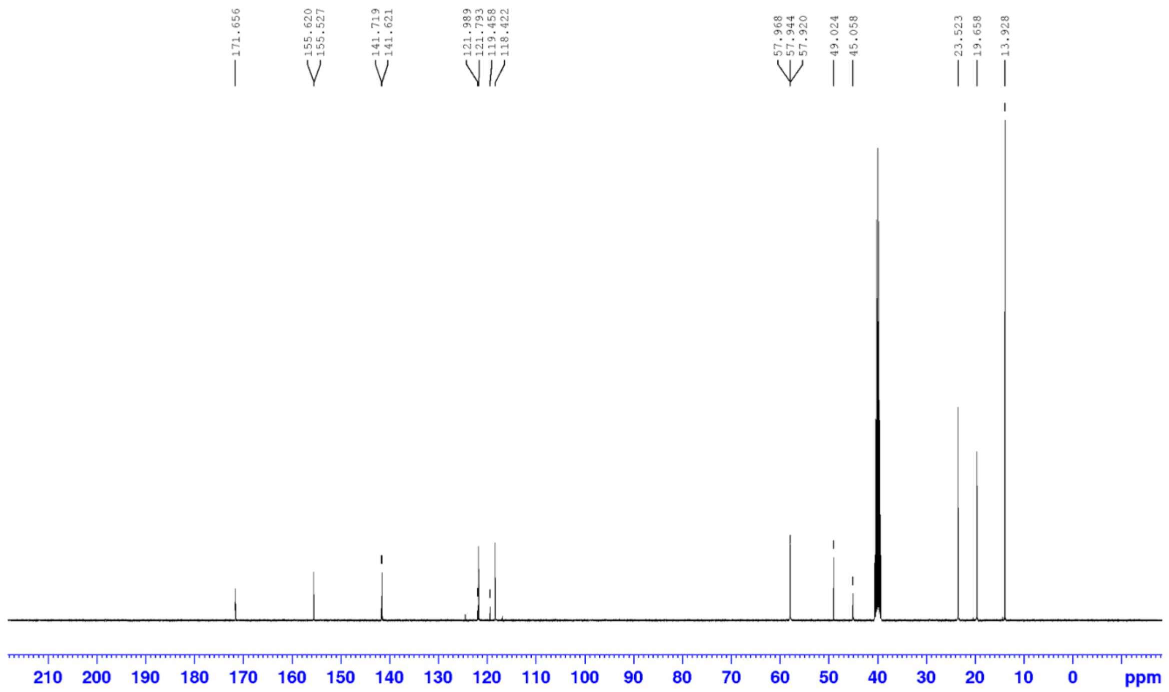

Figure S15 - $^{13}C\{^{1}H\}$ NMR spectra of **1** in DMSO-d_6 at 298 K.

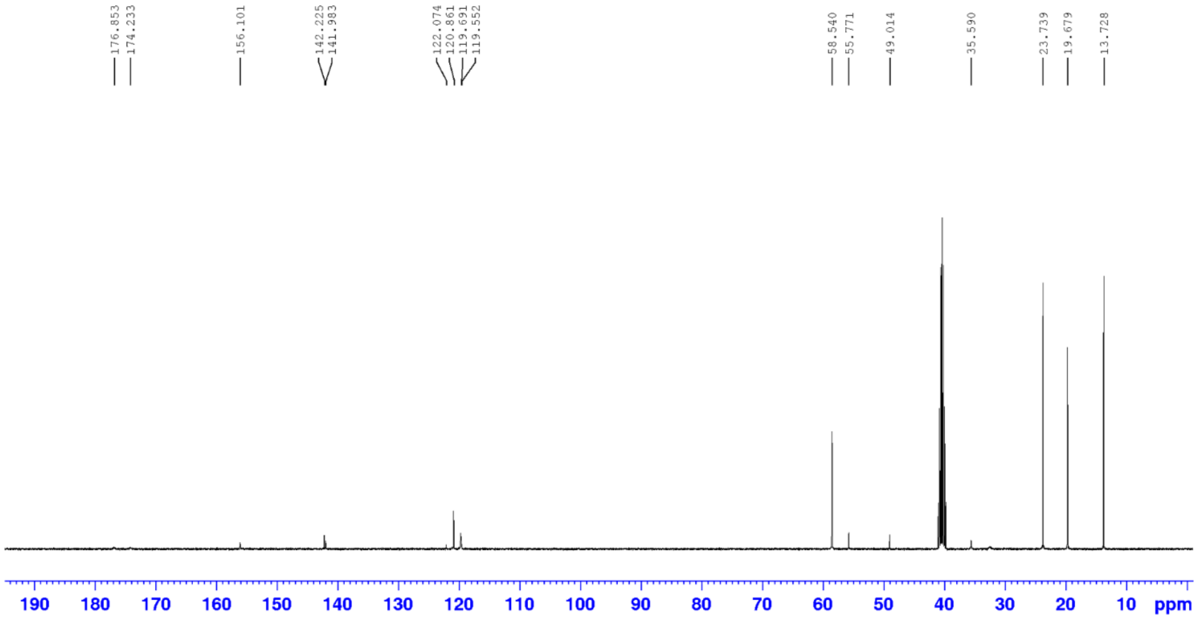

Figure S16 - $^{13}C\{^{1}H\}$ NMR spectra of **2** in DMSO-d_6 at 343 K.

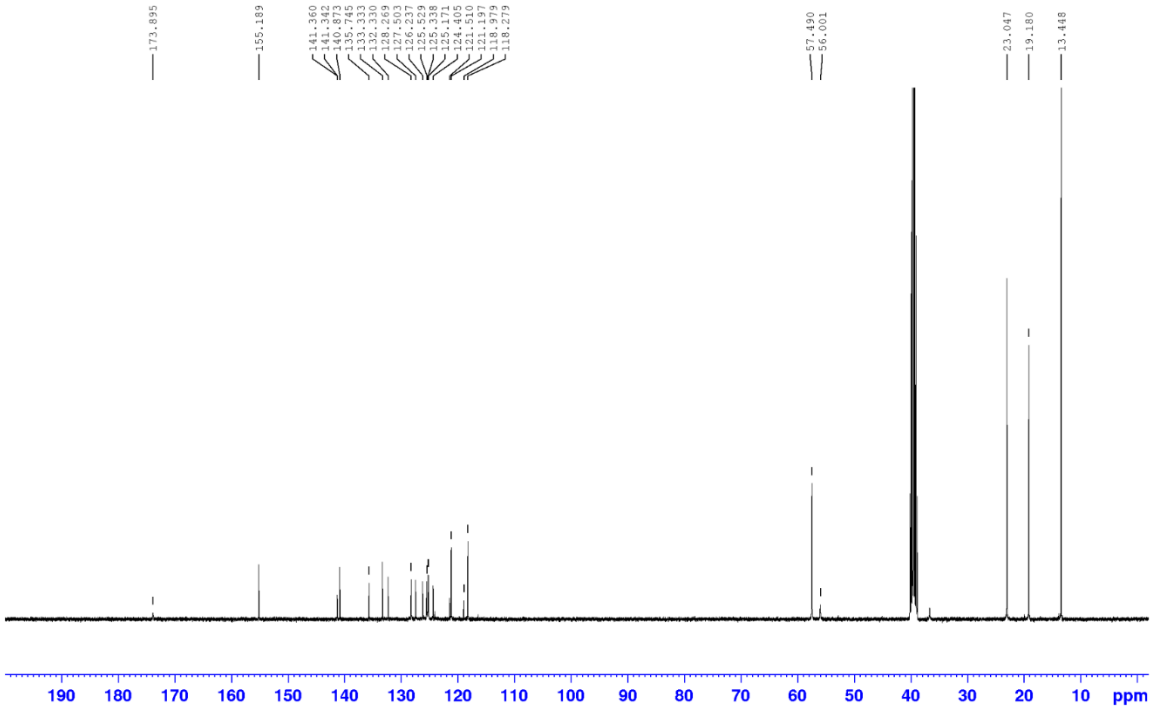

Figure S17 - $^{13}C\{^1H\}$ NMR spectra of **3** in DMSO-d_6 at 298 K.

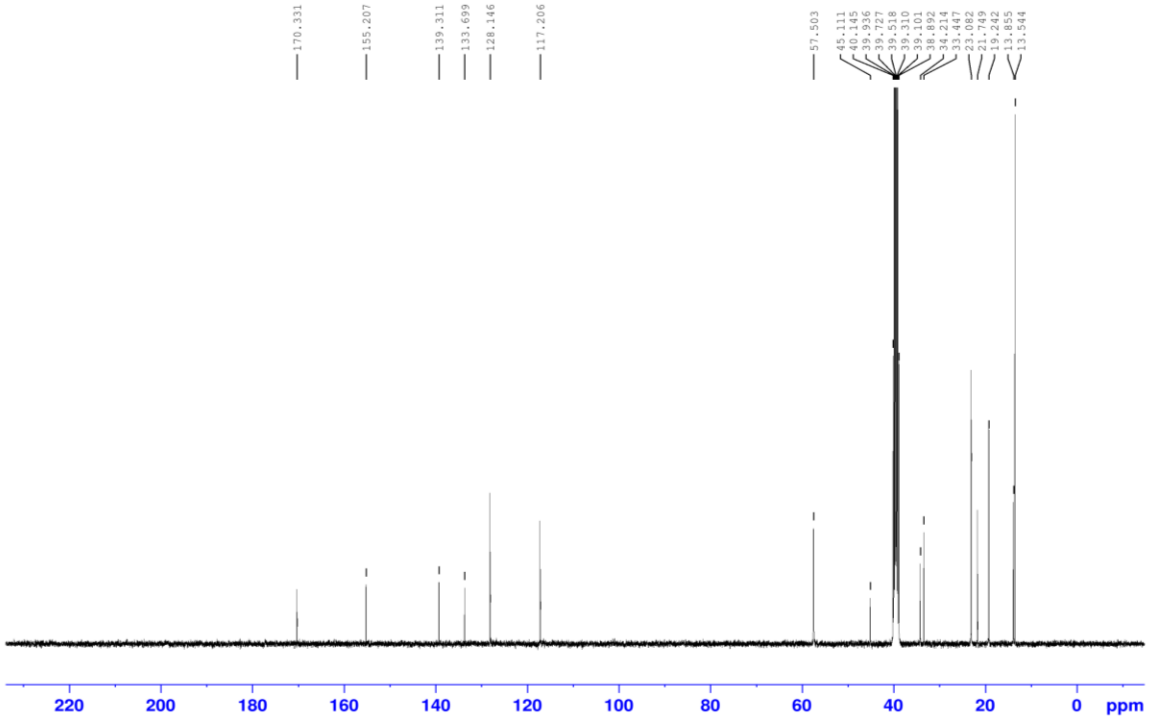

Figure S18 - $^{13}C\{^1H\}$ NMR spectra of **4** in DMSO-d_6 at 298 K.

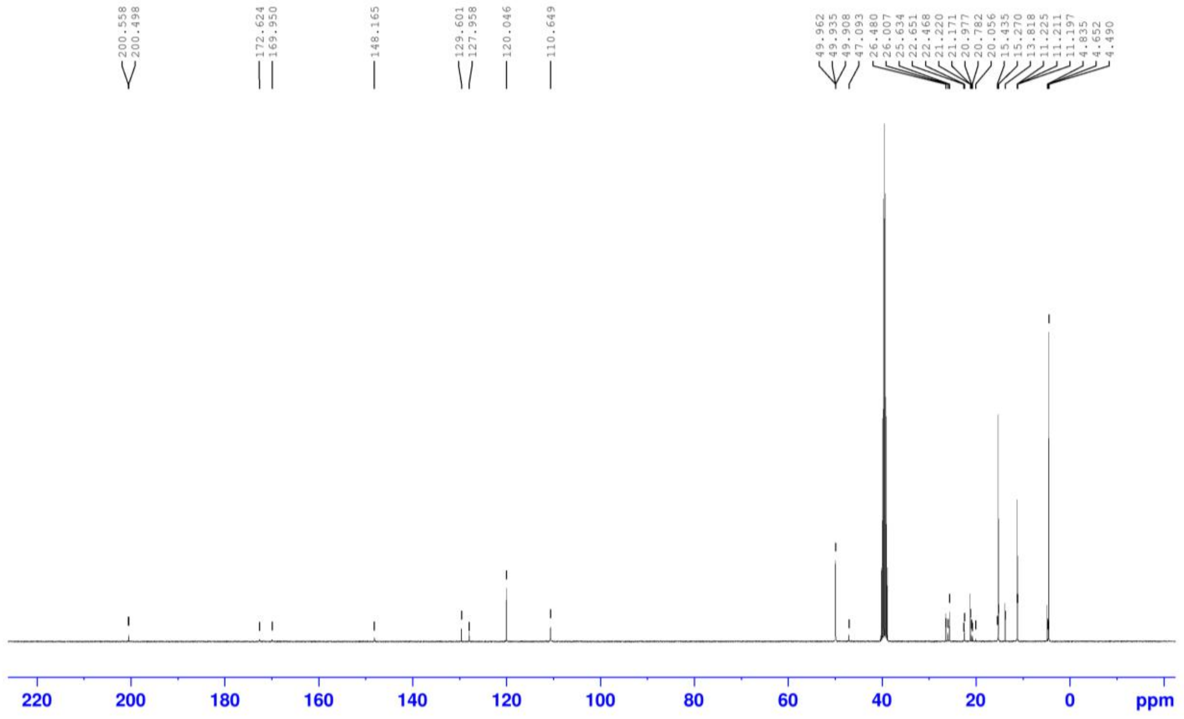

Figure S19 - $^{13}C\{^{1}H\}$ NMR spectra of **5** in DMSO-d_6 at 298 K.

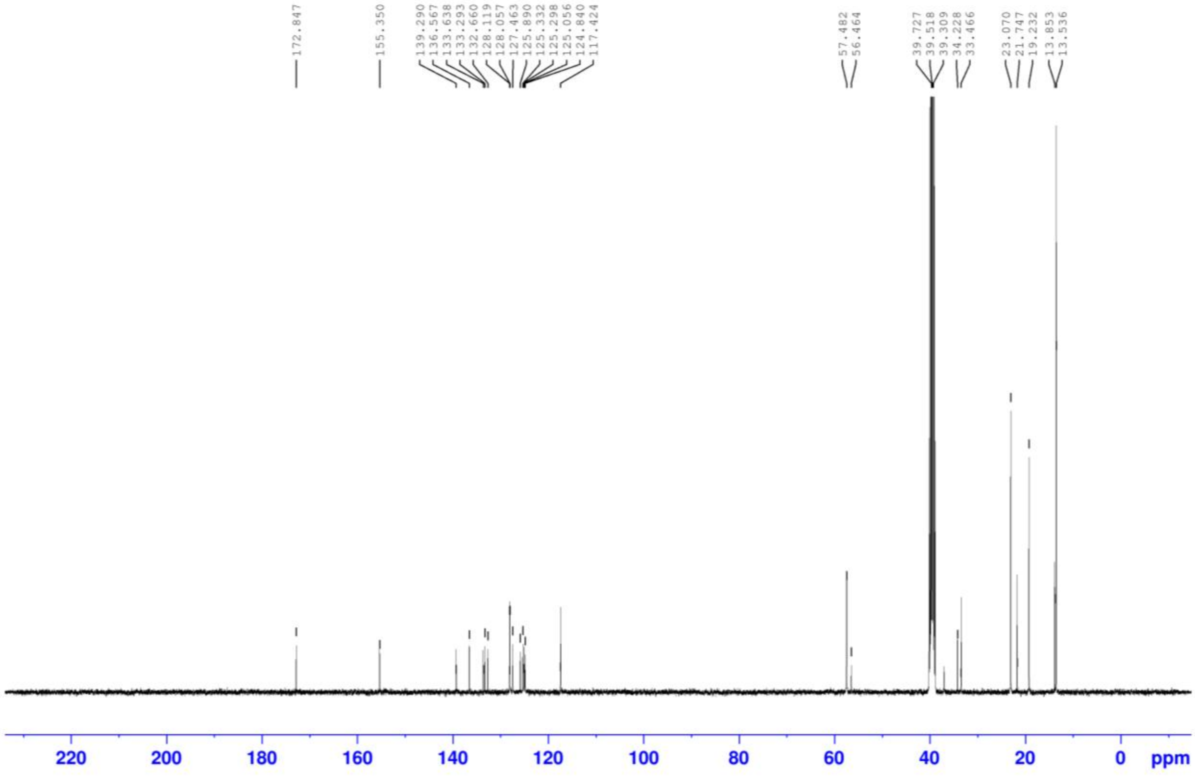

Figure S20 - $^{13}C\{^{1}H\}$ NMR spectra of **6** in DMSO-d_6 at 298 K.

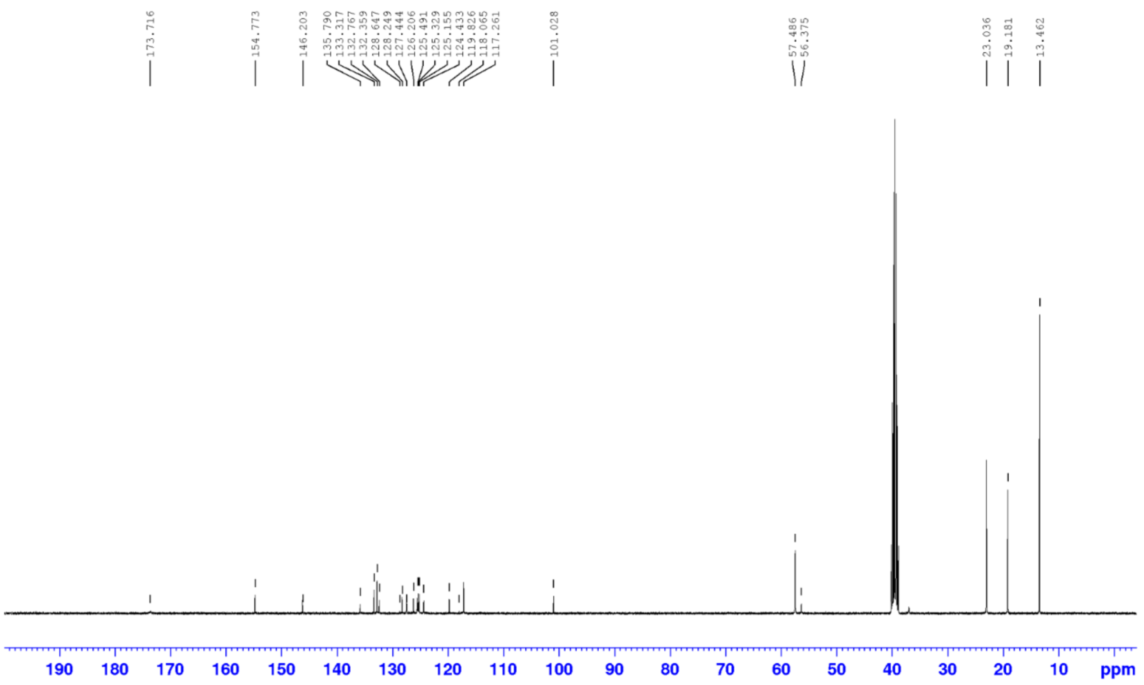

Figure S21 - $^{13}C\{^{1}H\}$ NMR spectra of **7** in DMSO-d_6 at 298 K.

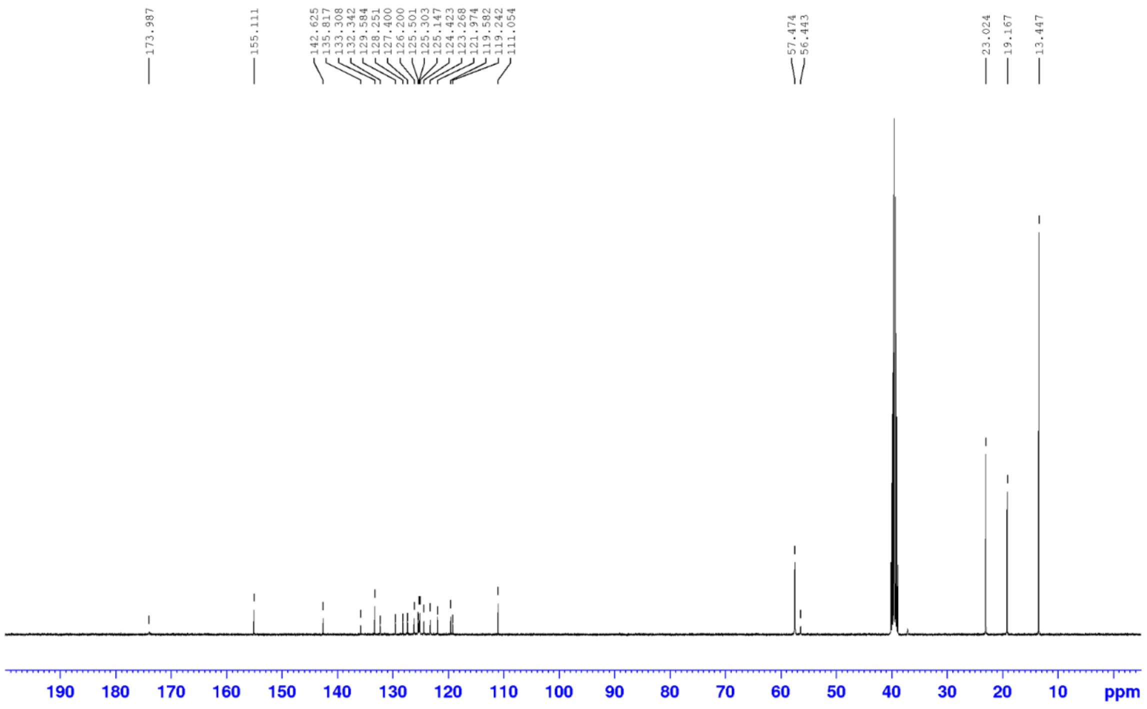

Figure S22 - $^{13}C\{^{1}H\}$ NMR spectra of **8** in DMSO-d_6 at 298 K.

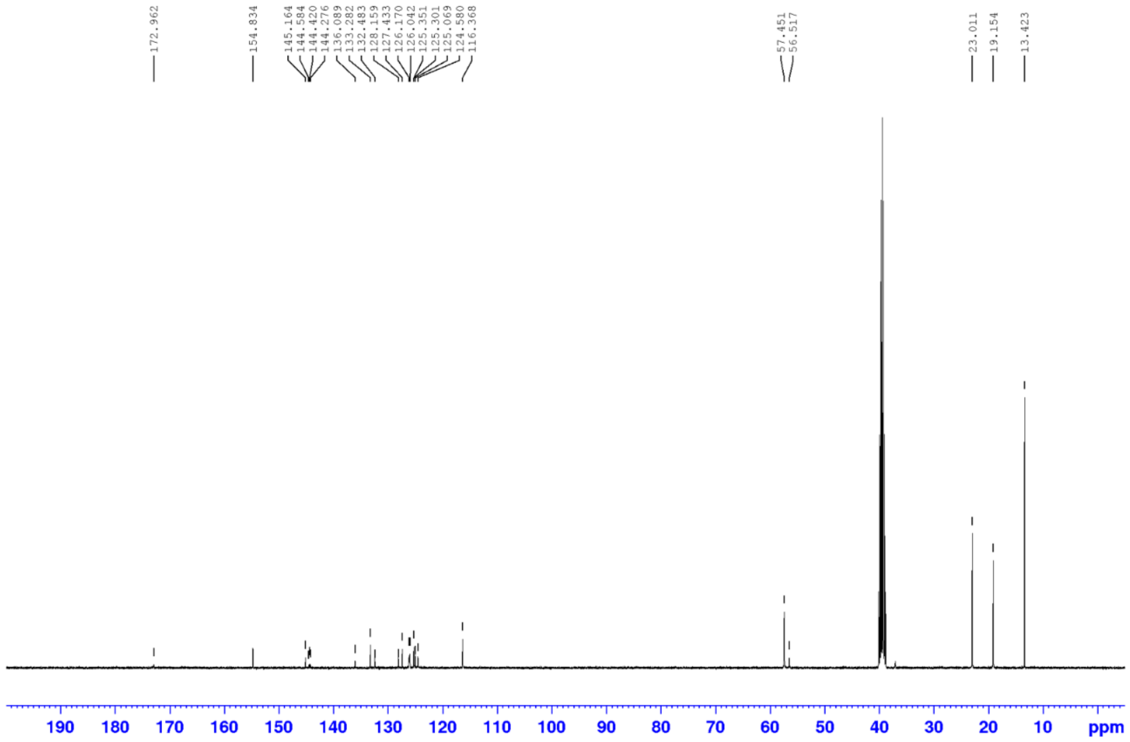

Figure S23 - $^{13}C\{^{1}H\}$ NMR spectra of **9** in DMSO-d_6 at 298 K.

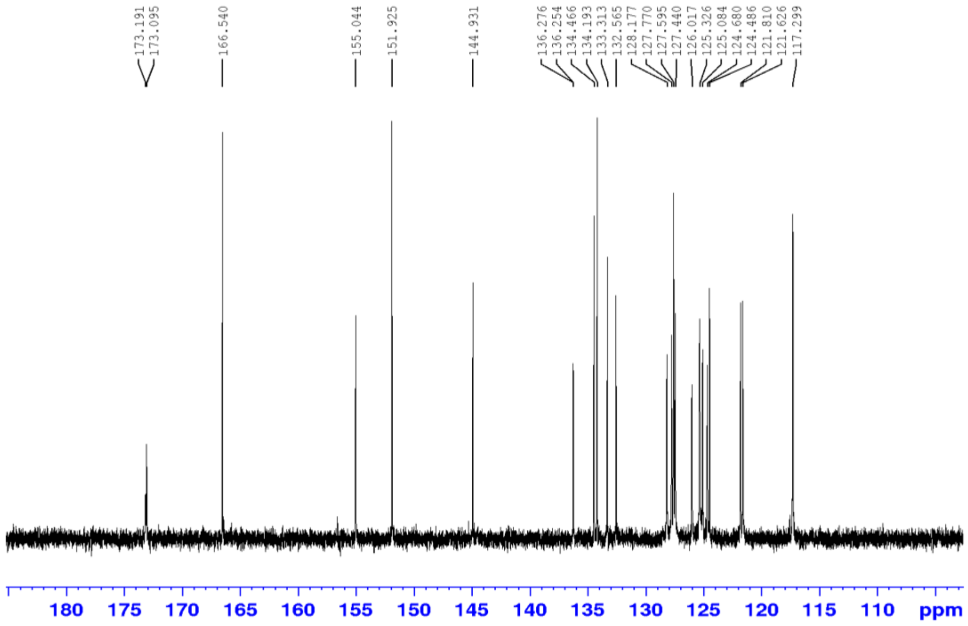

Figure S24 - $^{13}C\{^{1}H\}$ NMR spectra of **10** in DMSO-d_6 at 298 K.

Section S5.3: ¹H Quantitative NMR (qNMR) Spectroscopy Data

Section S5.3.1: ¹H qNMR spectra in DMSO-d_6/1% DCM

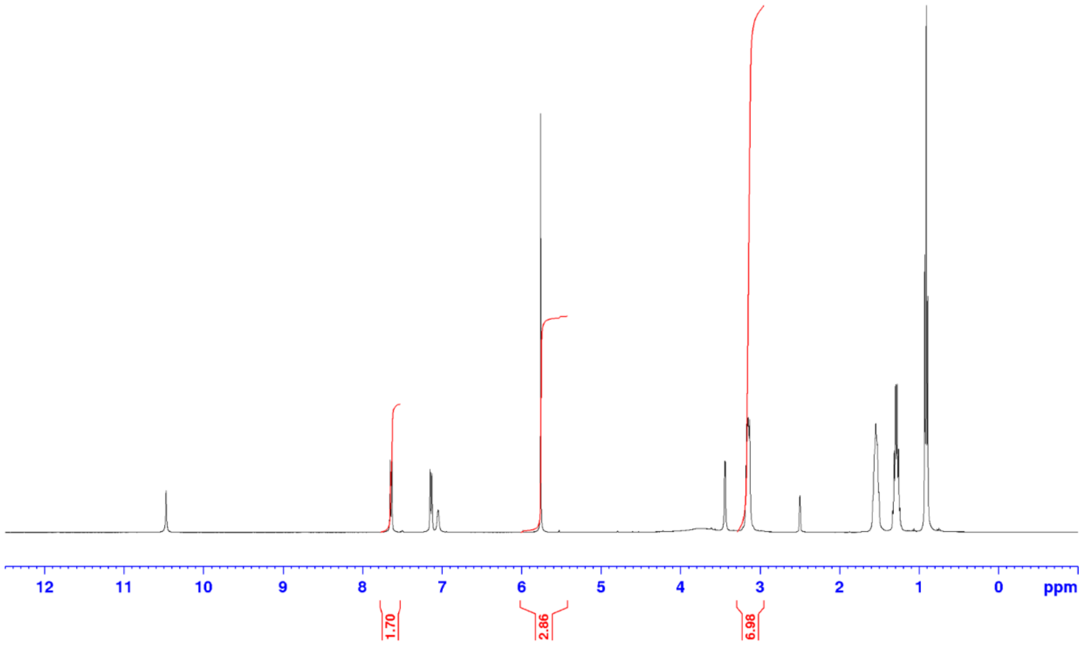

Figure S25 - ¹H NMR spectra with delay (d_1 = 60 s) of **1** (112 mM) in DMSO-d_6/1 % DCM at 298 K. Comparative integration indicates evidence of higher order aggregate formation with an apparent loss of 15 % of the anionic component and 14 % of the cationic component.

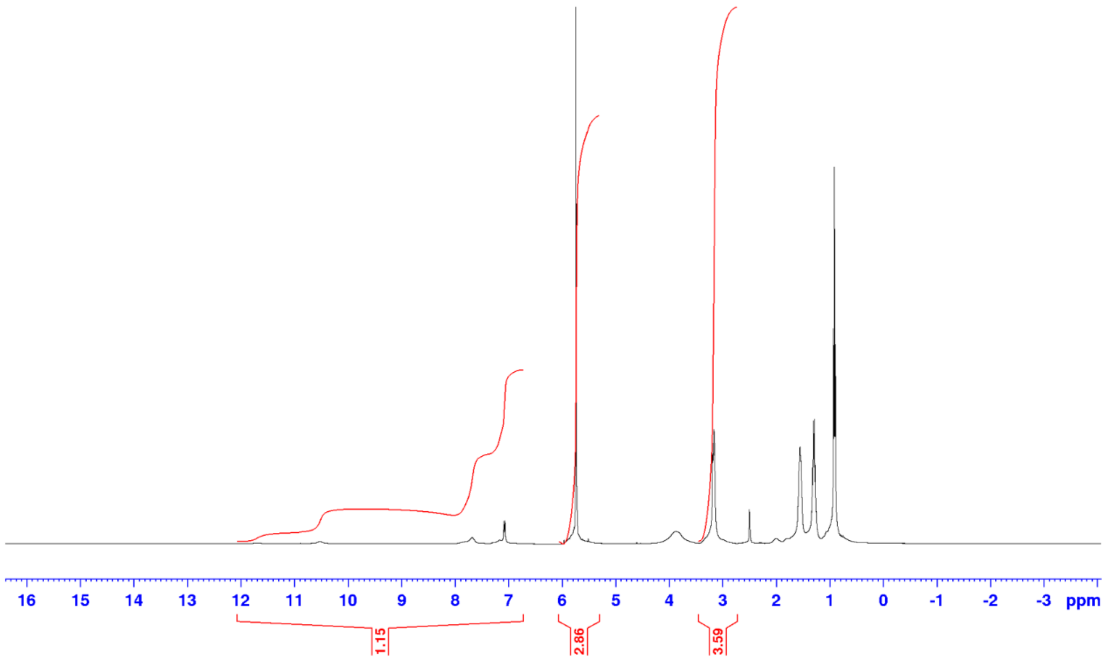

Figure S26 - ¹H NMR spectra with delay (d_1 = 60 s) of **2** (112 mM) in DMSO-d_6/1 % DCM Comparative integration indicates evidence of higher order aggregate formation with an apparent loss of 72 % of the anionic component and 78 % of the cationic component. Due to slow exchange in this solvent system at 298 K, integration is approximate.

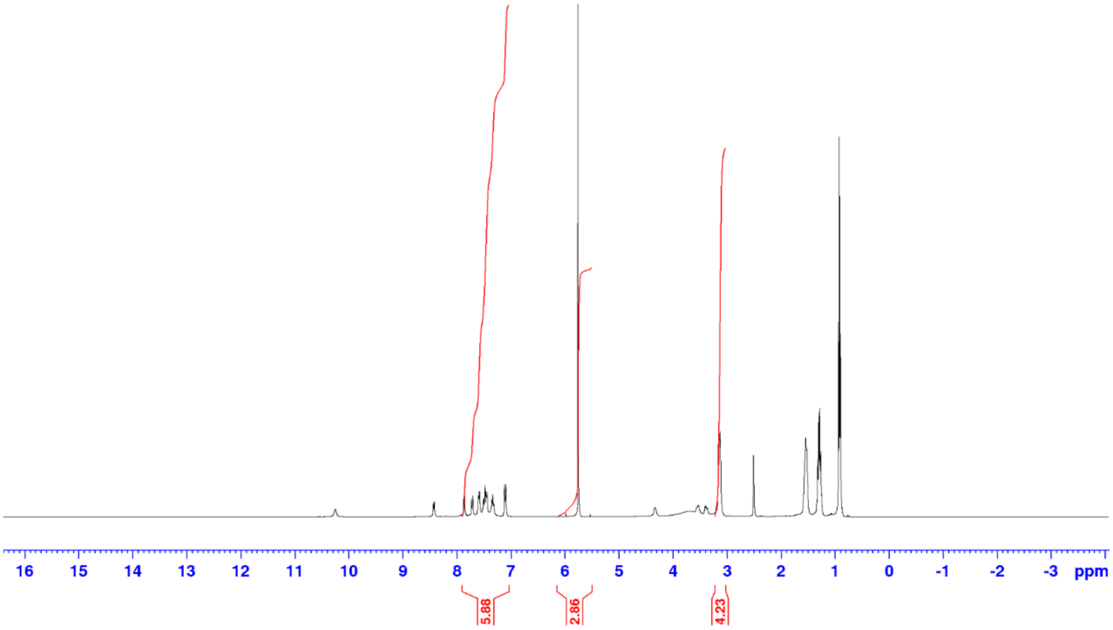

Figure S27 - ^{1}H NMR spectra with delay (d_1 = 60 s) of **3** (112 mM) in DMSO-d_6/1 % DCM at 298 K. Comparative integration indicates evidence of higher order aggregate formation with an apparent loss of 47 % of the anionic component and 47 % of the cationic component.

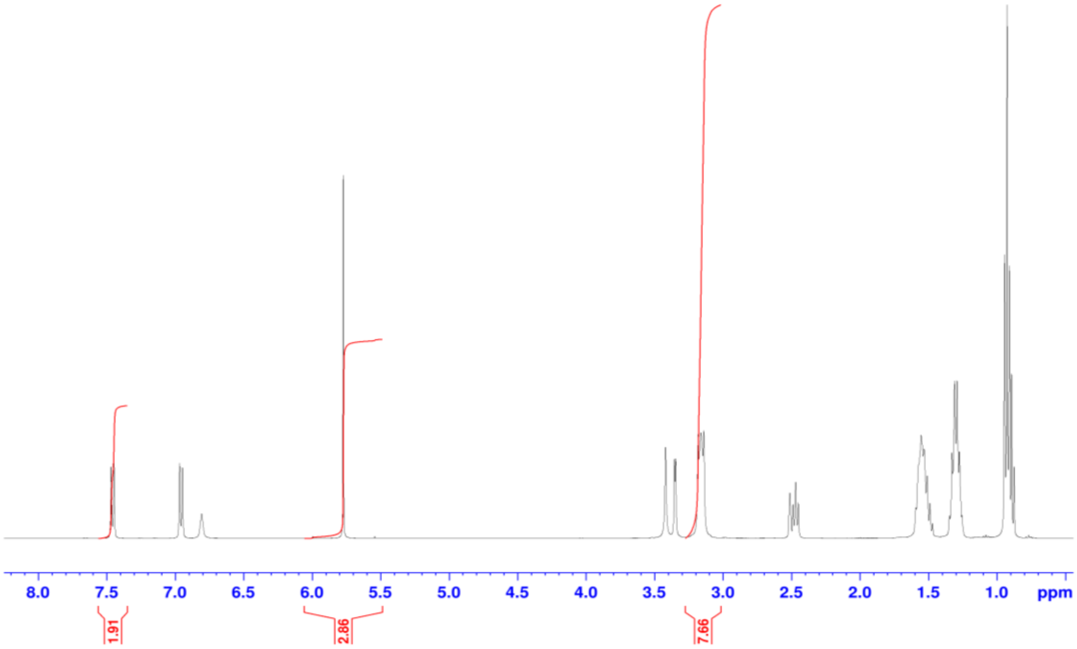

Figure S28 - ^{1}H NMR spectra with delay (d_1 = 60 s) of **4** (112 mM) in DMSO-d_6/1 % DCM at 298 K. Comparative integration indicates evidence of higher order aggregate formation with an apparent loss of 5 % of the anionic component and 5 % of the cationic component.

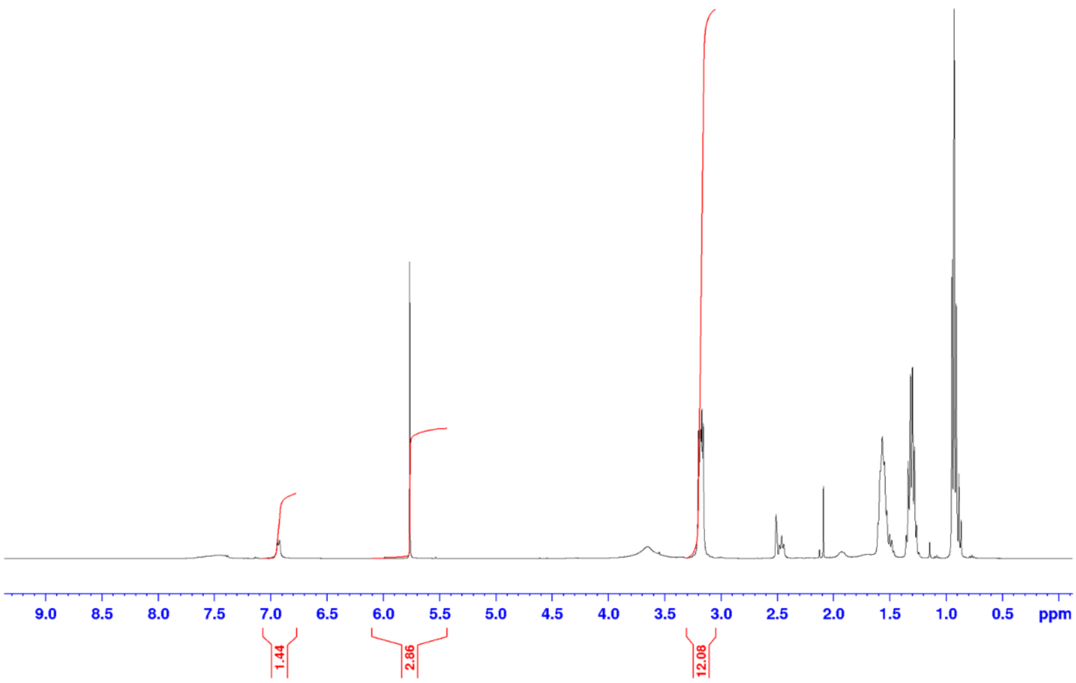

Figure S29 - ^{1}H NMR spectra with delay (d_1 = 60 s) of **5** (112 mM) in DMSO-d_6/1 % DCM. Comparative integration indicates evidence of higher order aggregate formation with an apparent loss of 25 % of the anionic component and 24 % of the cationic component. Due to slow exchange in this solvent system at 298 K, integration is approximate.

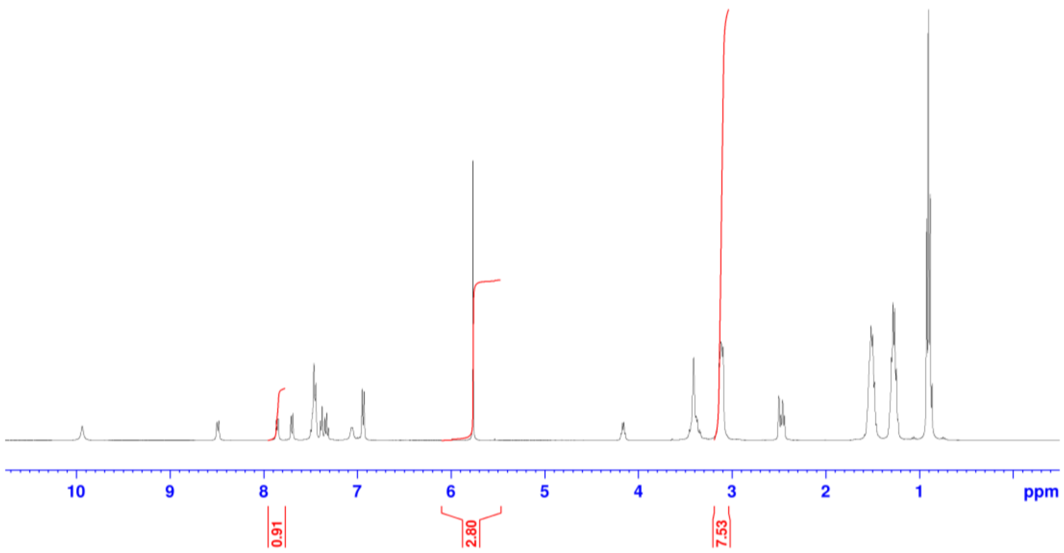

Figure S30 - ^{1}H NMR spectra with delay (d_1 = 60 s) of **6** (112 mM) in DMSO-d_6/1 % DCM at 298 K. Comparative integration indicates evidence of higher order aggregate formation with an apparent loss of 9 % of the anionic component and 6 % of the cationic component.

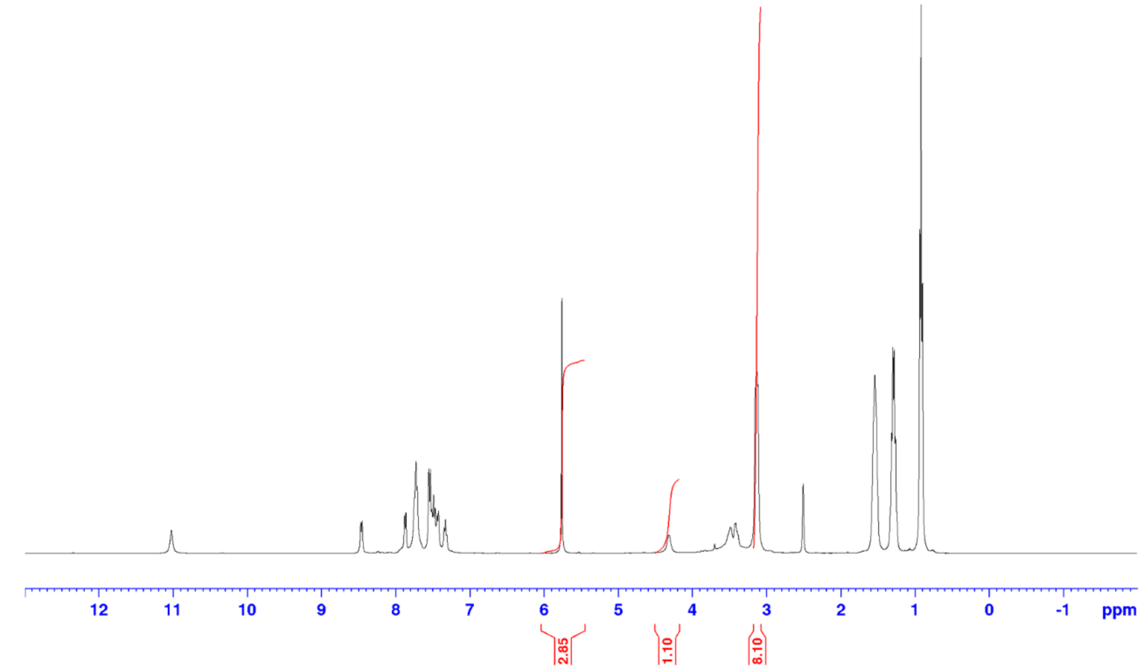

Figure S31 - ^{1}H NMR spectra with delay (d_1 = 60 s) of **7** (112 mM) in DMSO-d_6/1 % DCM at 298 K. Comparative integration indicates 0 % of the anionic and cationic component of **7** has become NMR silent.

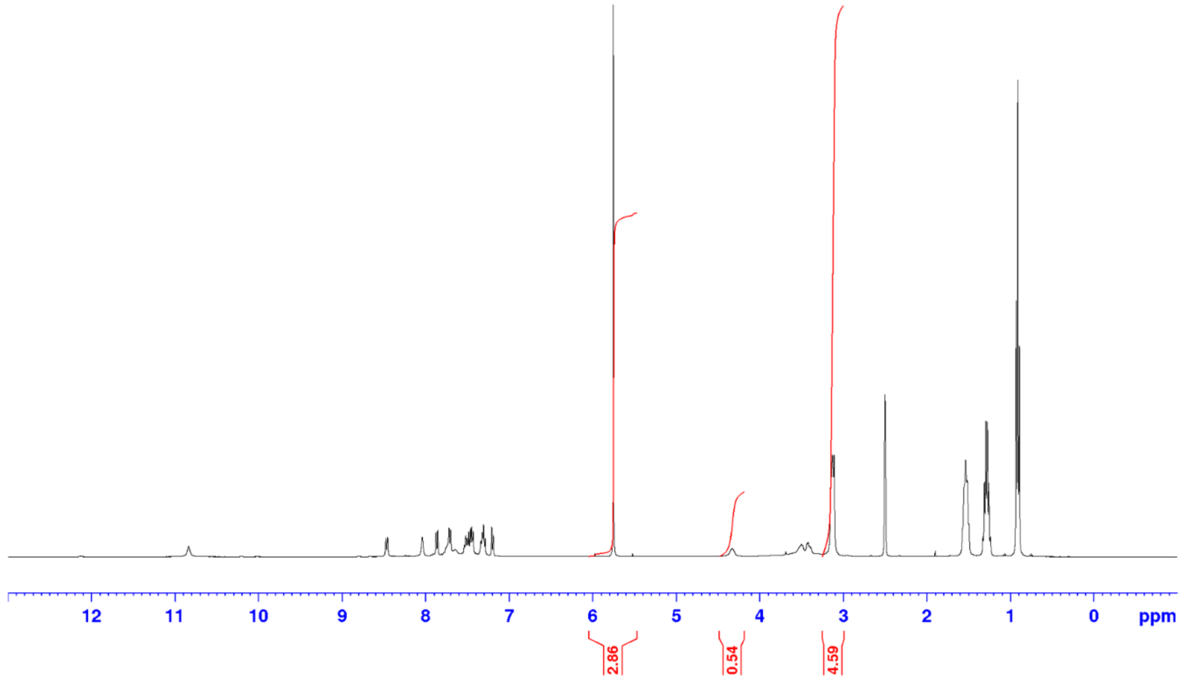

Figure S32 - ^{1}H NMR spectra with delay (d_1 = 60 s) of **8** (112 mM) in DMSO-d_6/1 % DCM at 298 K. Comparative integration indicates 46 % of the anionic and 43 % cationic component of **8** has become NMR silent.

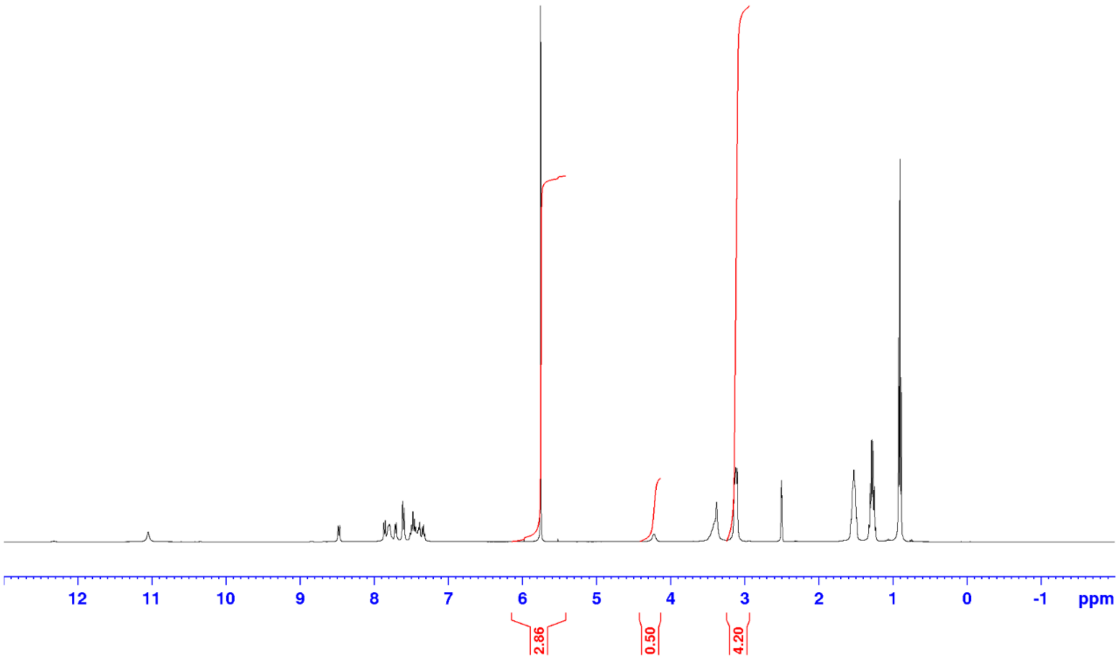

Figure S33 - ^{1}H NMR spectra with delay (d_1 = 60 s) of **9** (112 mM) in DMSO-d_6/1 % DCM at 298 K. Comparative integration indicates 50 % of the anionic and 48 % cationic component of **9** has become NMR silent.

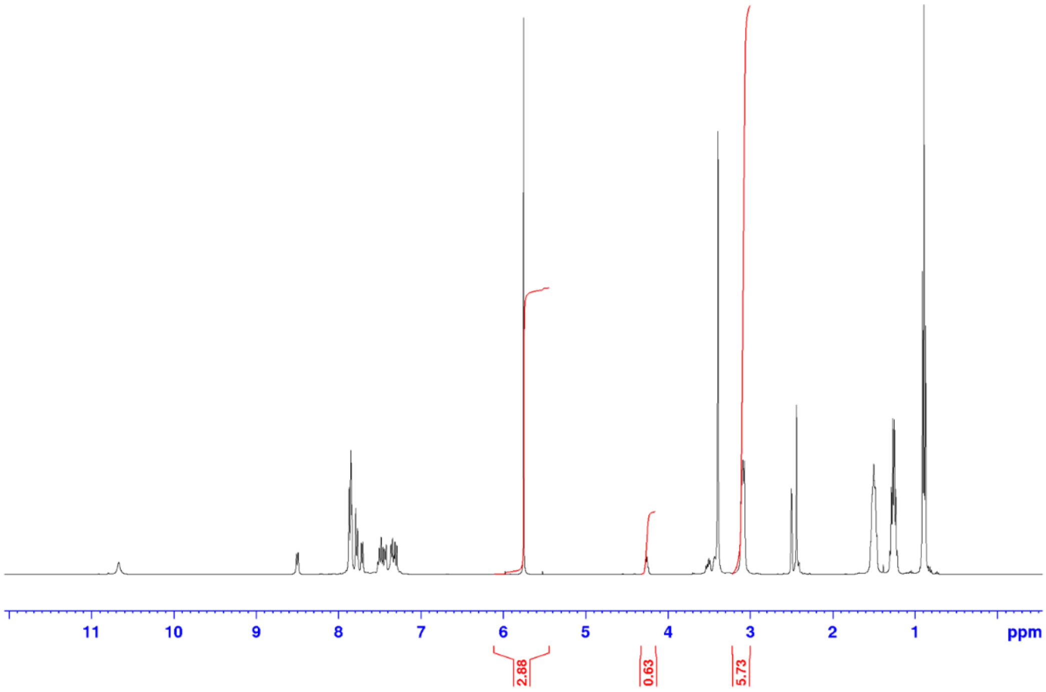

Figure S34 - ^{1}H NMR spectra with delay (d_1 = 60 s) of **10** (112 mM) in DMSO-d_6/1 % DCM at 298 K. Comparative integration indicates 29 % of the anionic and 28 % cationic component of **10** has become NMR silent.

Section S5.3.2: ^{1}H qNMR spectroscopy in D_2O/5 % EtOH

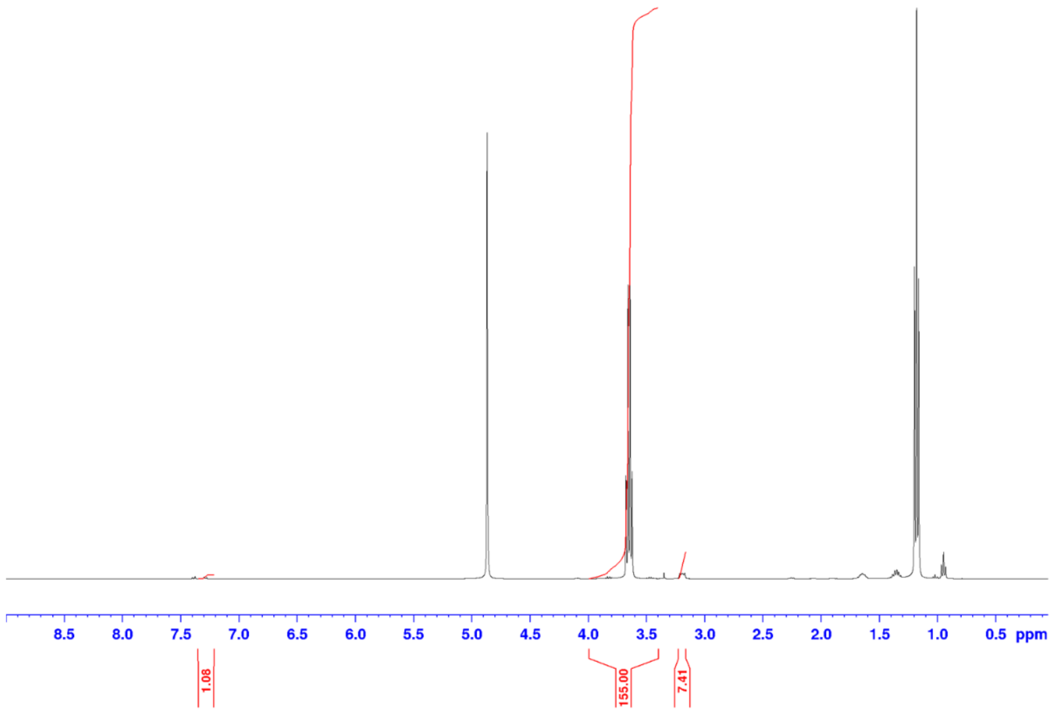

Figure S35 - ^{1}H NMR spectra with delay (d_1 = 60 s) of **1** (5.56 mM) in D_2O/5 % EtOH at 298 K. Comparative integration indicates evidence of higher order aggregate formation with an apparent loss of 54 % of the anionic component and 56 % of the cationic component.

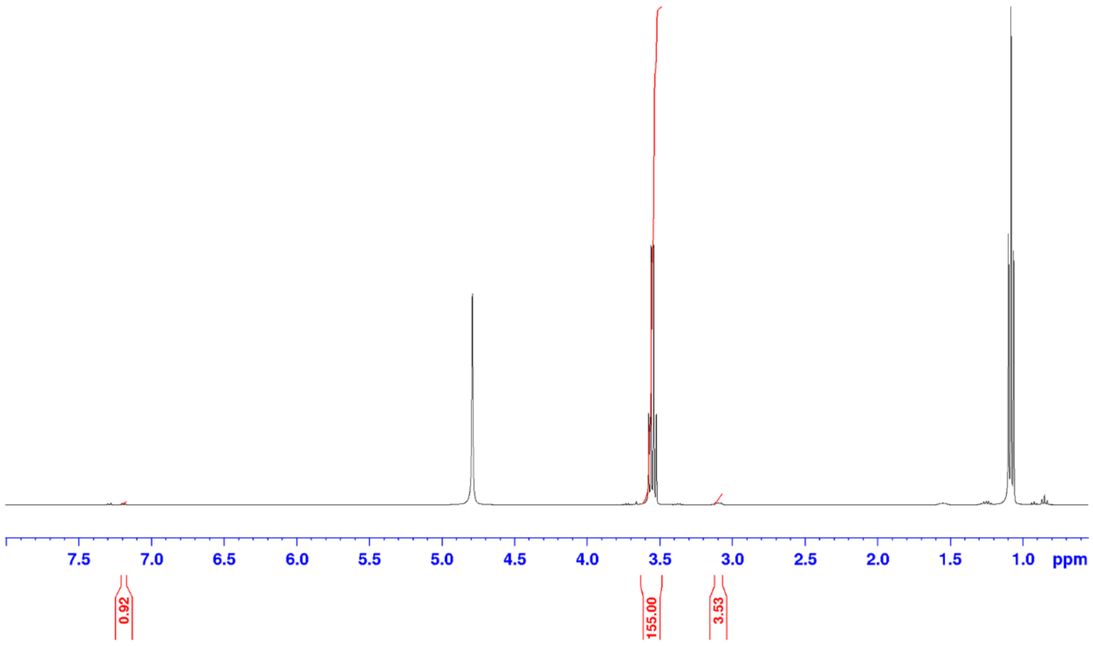

Figure S36 - ^{1}H NMR spectra with delay (d_1 = 60 s) of **2** (5.56 mM) in D_2O/5 % EtOH at 298 K. Comparative integration indicates evidence of higher order aggregate formation with an apparent loss of 53 % of the anionic component and 50 % of the cationic component.

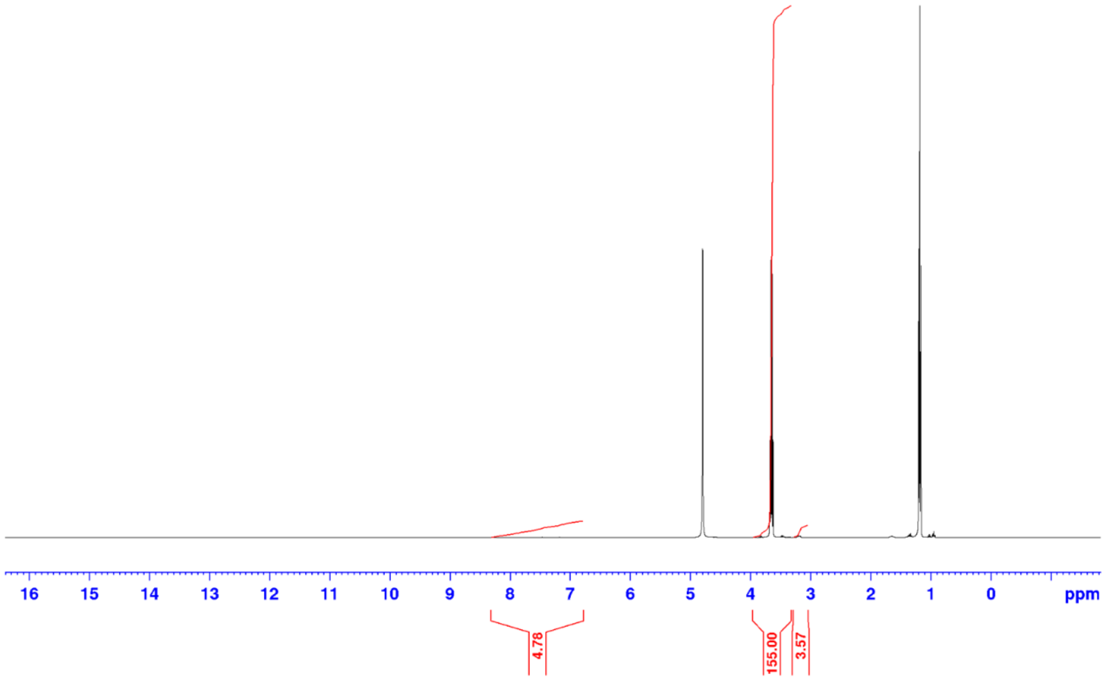

Figure S37 - ^{1}H NMR spectra with delay (d_1 = 60 s) of **3** (5.56 mM) in D_2O/5 % EtOH at 298 K. Comparative integration indicates evidence of higher order aggregate formation with an apparent loss of 56 % of the anionic component and 56 % of the cationic component.

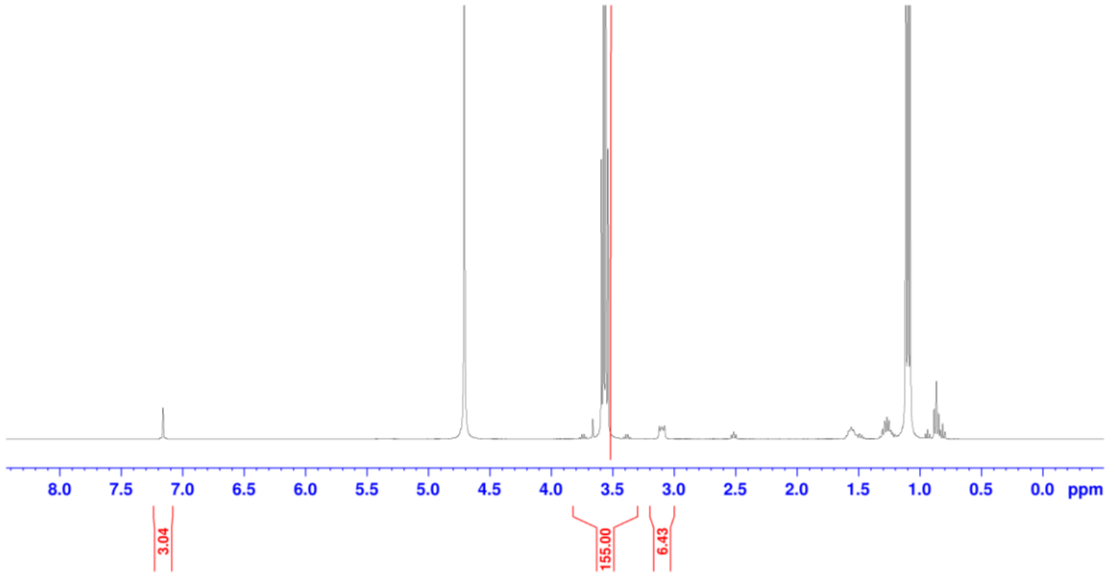

Figure S38 - ^{1}H NMR spectra with delay (d_1 = 60 s) of **4** (5.56 mM) in D_2O/5 % EtOH at 298 K. Comparative integration indicates evidence of higher order aggregate formation with an apparent loss of 25 % of the anionic component and 20 % of the cationic component.

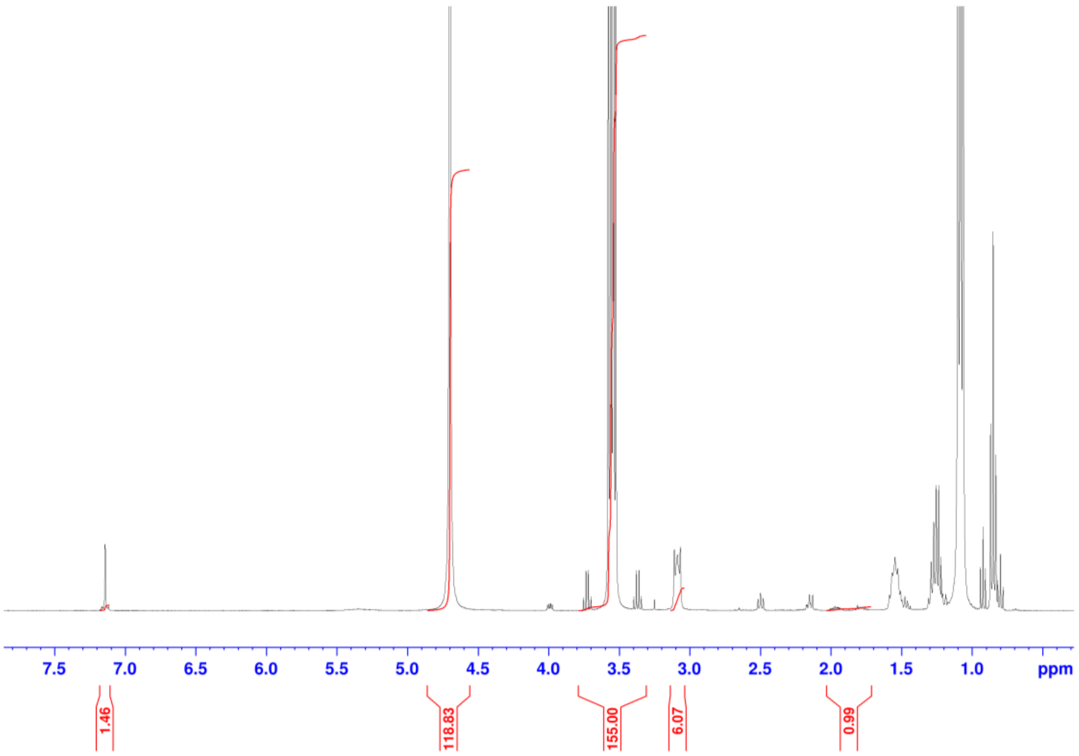

Figure S39 - ^{1}H NMR spectra with delay (d_1 = 60 s) of **5** (5.58 mM) in D_2O/5 % EtOH at 298 K. Comparative integration indicates evidence of higher order aggregate formation with an apparent loss of 67 % of the anionic component and 66 % of the cationic component.

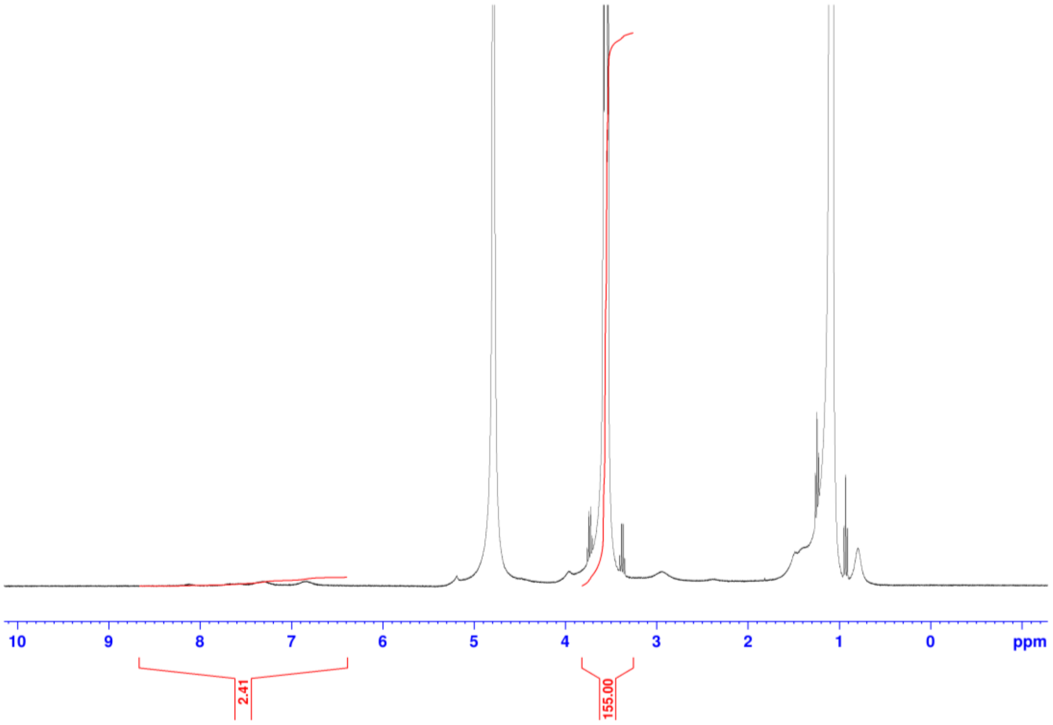

Figure S40 - ^{1}H NMR spectra with delay (d_1 = 60 s) of **6** (5.56 mM) in D_2O/5 % EtOH at 298 K. Comparative integration indicates evidence of higher order aggregate formation with an apparent loss of 88 % of the anionic component. Reliable integration of the cation is not possible due to overlap with solvent.

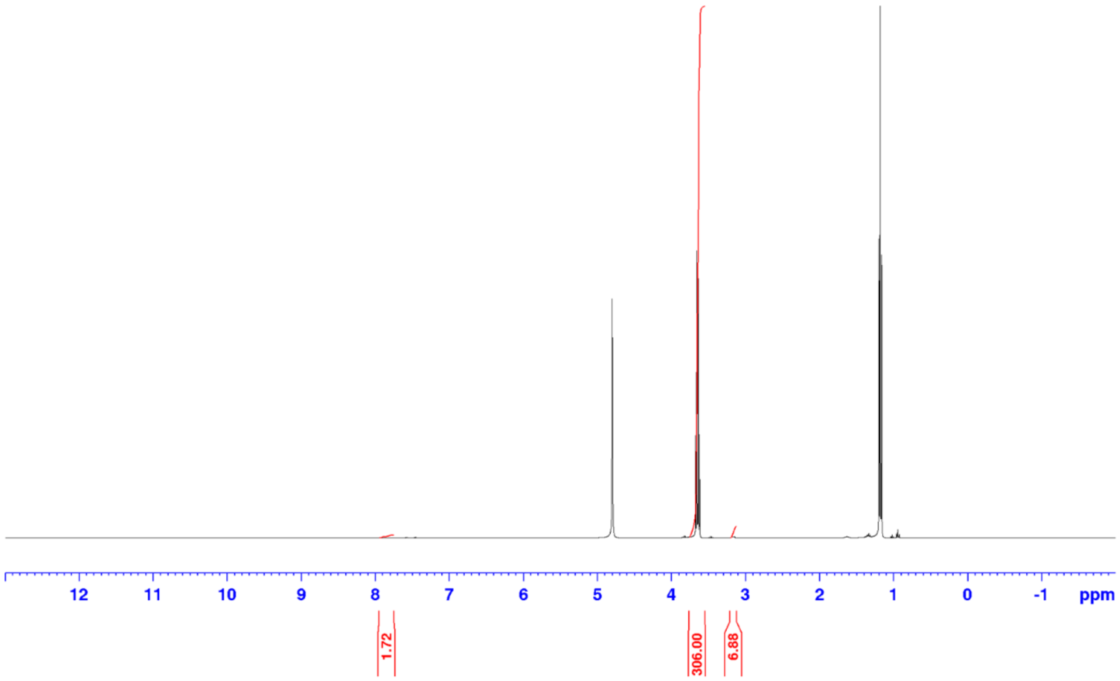

Figure S41 - ^{1}H NMR spectra with delay (d_1 = 60 s) of **7** (5.56 mM) in D_2O/5 % EtOH at 298 K. Comparative integration indicates 14 % of the anionic and 14 % of the cationic component of **7** has become NMR silent.

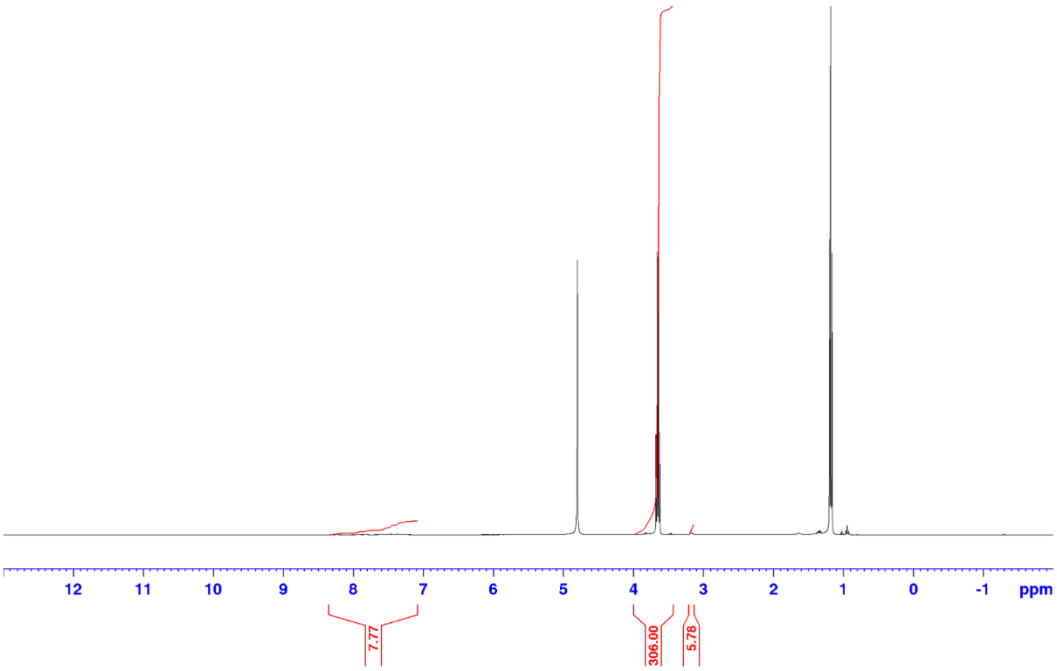

Figure S42 - ^{1}H NMR spectra with delay (d_1 = 60 s) of **8** (5.56 mM) in D_2O/5 % EtOH at 298 K. Comparative integration indicates 29 % of the anionic and 28 % of the cationic component of **8** has become NMR silent.

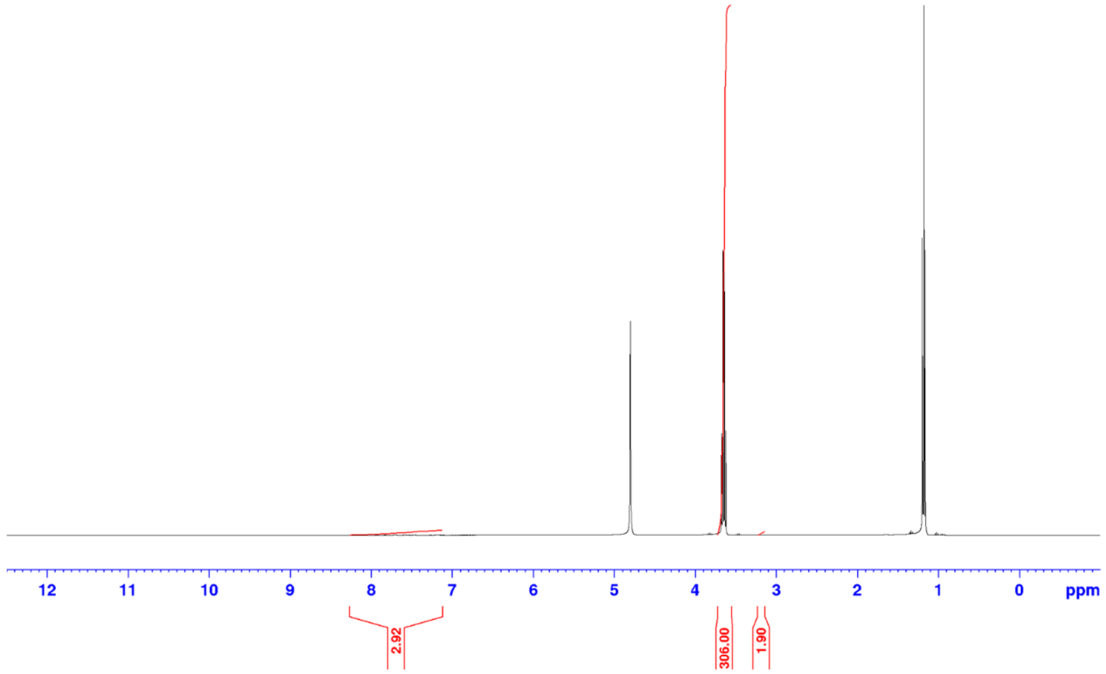

Figure S43 - ^{1}H NMR spectra with delay (d_1 = 60 s) of **9** (5.56 mM) in D_2O/5 % EtOH at 298 K. Comparative integration indicates 74 % of the anionic and 76 % of the cationic component of **9** has become NMR silent.

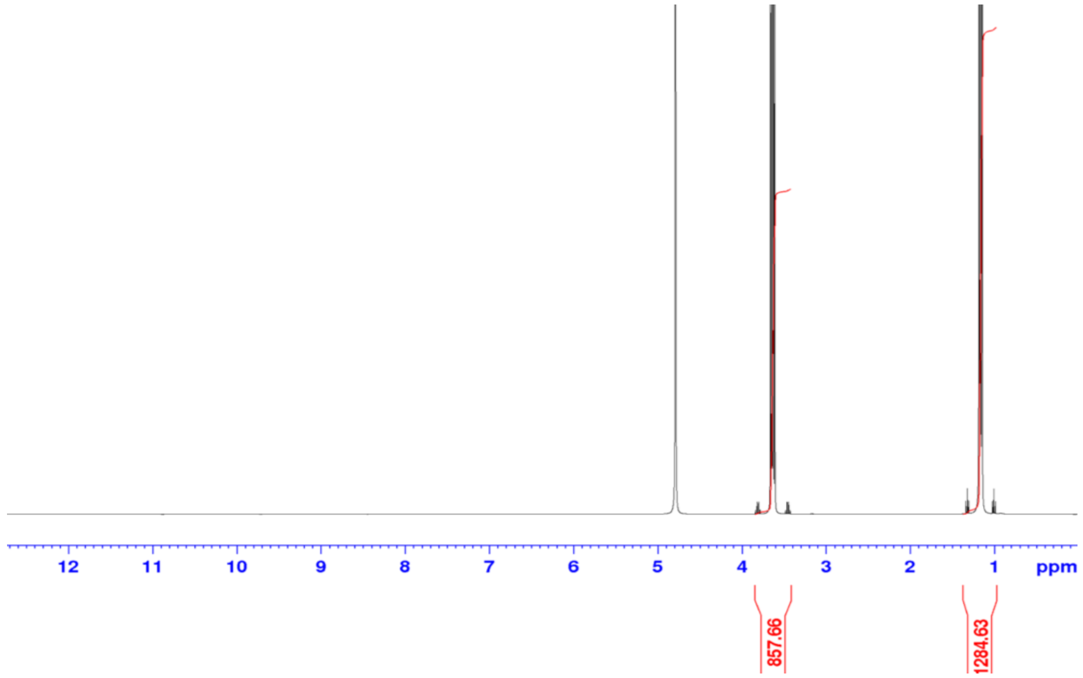

Figure S44 - ^{1}H NMR spectra with delay (d_1 = 60 s) of **10** (1.5 mM) in D_2O/5 % EtOH at 298 K. Accurate integration of SSA anion and cation signals could not be obtained due to low signal intensity and signal overlap.

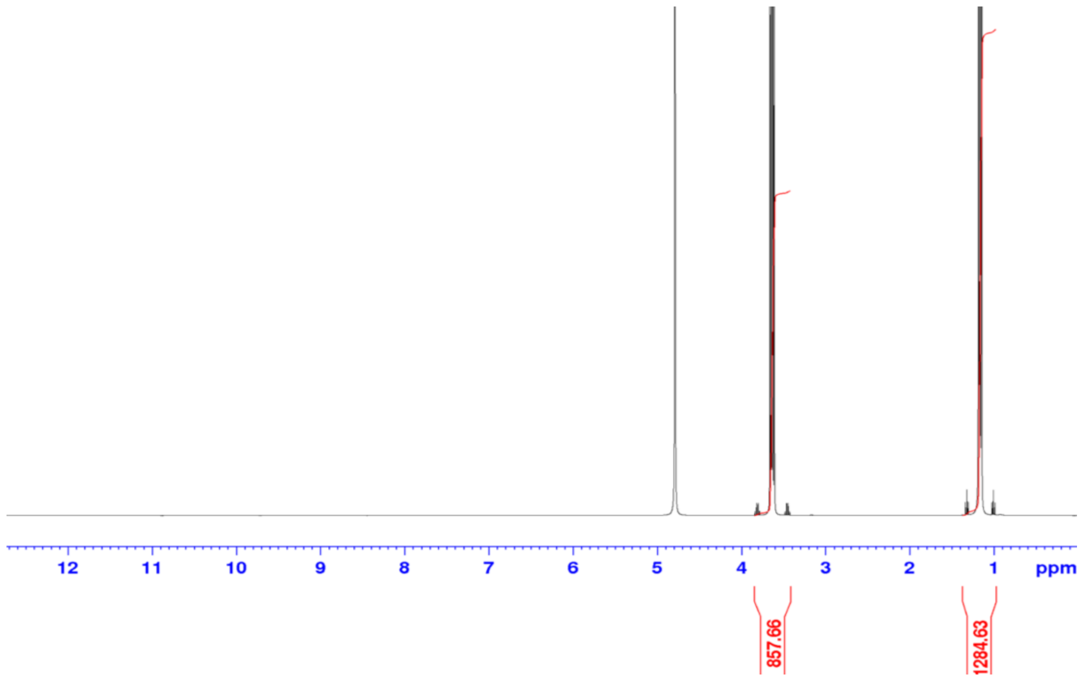

Figure S45 - ^{1}H NMR spectra with delay (d_1 = 60 s) of **10** (1.0 mM) in D_2O/5 % EtOH at 298 K. Accurate integration of SSA anion and cation signals could not be obtained due to low signal intensity and signal overlap.

Table S2 - Summary of results from qNMR spectroscopy studies in DMSO-d_6 /1 % DCM and D_2O /5 % EtOH. Values given in % represent the observed proportion of compound that has been apparently 'lost'. Proton NMR spectroscopy experiments carried out at 298 K, at a total molar concentration of 112 mM and 5.56 mM respectively (unless otherwise stated). d_1 increased to 60s.

**SSAs**	**DMSO-d_6 /1 % DCM**		**D_2O/5 % EtOH**	
	**Anion (%)**	**Cation (%)**	**Anion (%)**	**Cation (%)**
**1**	15	14	54	56
**2**	72 (a)	78 (a)	53	50
**3**	47	47	56	56
**4**	5	5	25	20
**5**	25 (a)	24 (a)	67	66
**6**	9	6	88	b
**7**	0	0	14	14
**8**	46	43	29	28
**9**	50	48	74	76
**10**	d^c	d^c	31	29

a - Integration of spectra in DMSO-d_6 is unreliable due to slow exchange processes in this solvent system.
b - Integration of spectra in D_2O/5 % EtOH *is unreliable due to overlap with solvent peaks.*
c – Experiment conducted at 1 mM.
d - Accurate integration of SSA anion and cation signals could not be obtained due to low signal intensity and signal overlap.

Section S6: Dynamic Light Scattering Studies

Section S6.1: Data in D_2O/5 % EtOH

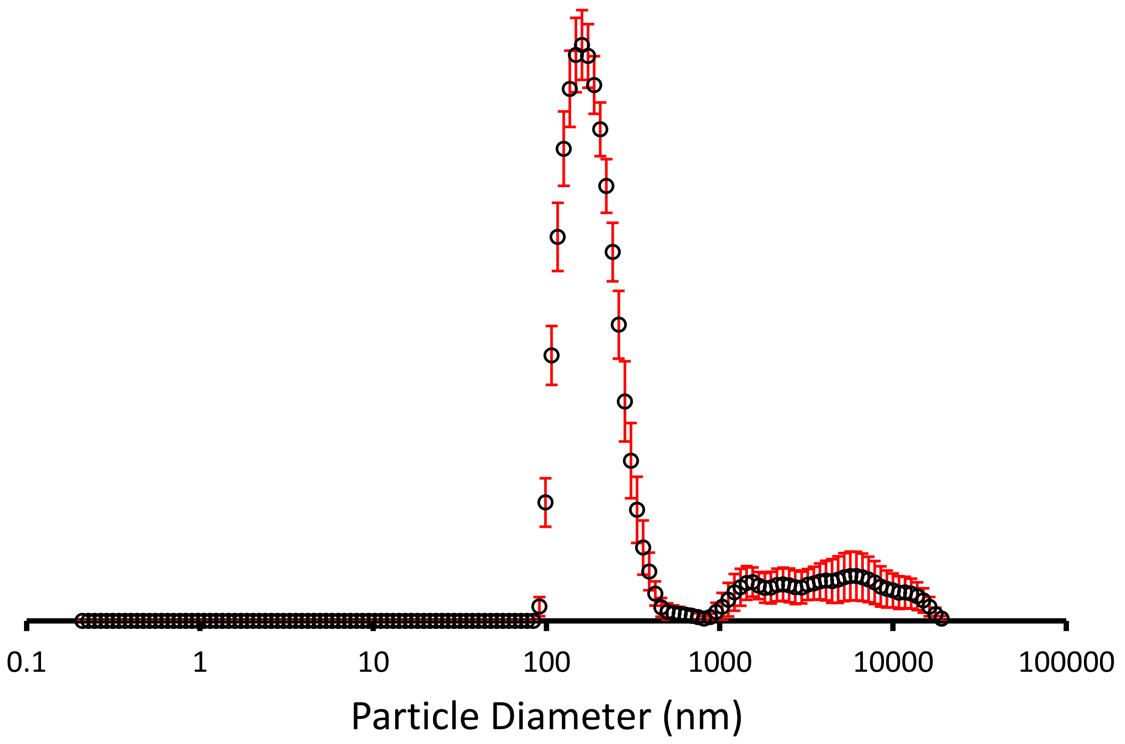

Figure S46 - The average intensity particle size distribution calculated (peak maxima = 188 and greater than 1000 nm) using 10 DLS runs for **1** (5.56 mM) in an H_2O/5 % EtOH solution at 298 K.

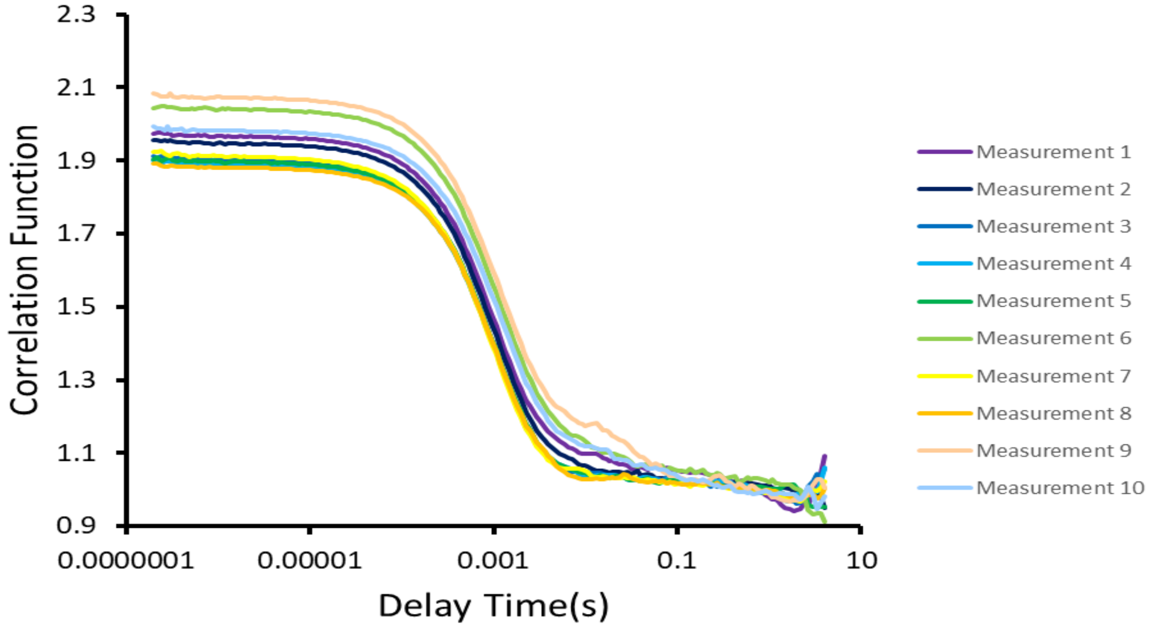

Figure S47 - Correlation function data for 10 DLS runs of **1** (5.56 mM) in an H_2O/5 % EtOH solution at 298 K.

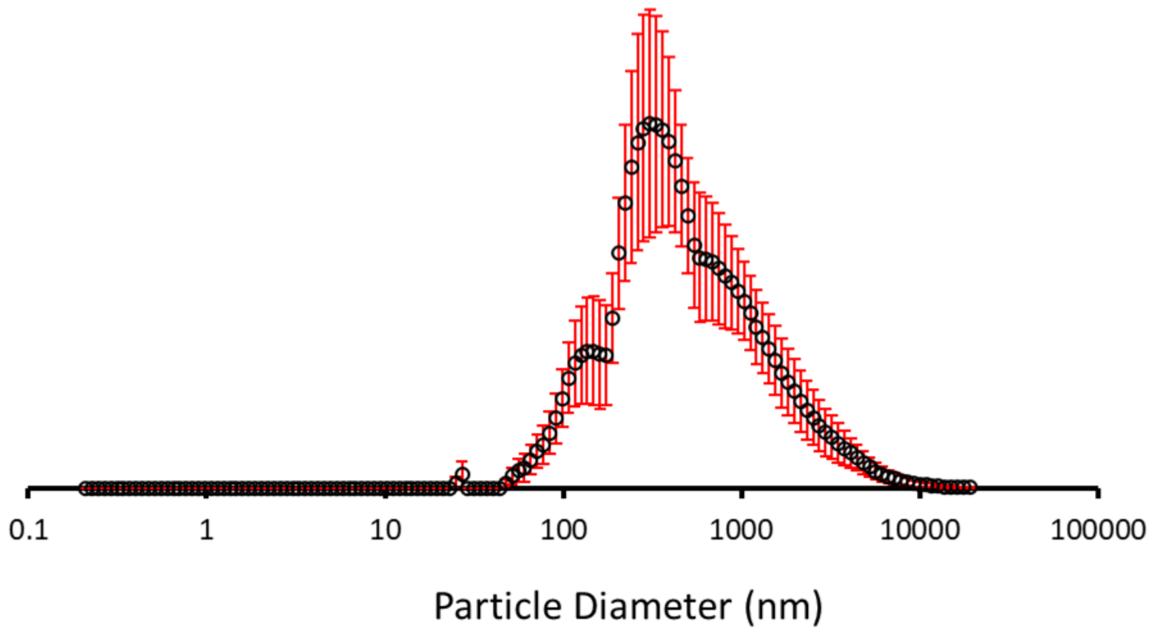

Figure S48 - The average intensity particle size distribution calculated (peak maxima = 409 nm) using 10 DLS runs for **2** (5.56 mM) in an H_2O/5 % EtOH solution at 298 K.

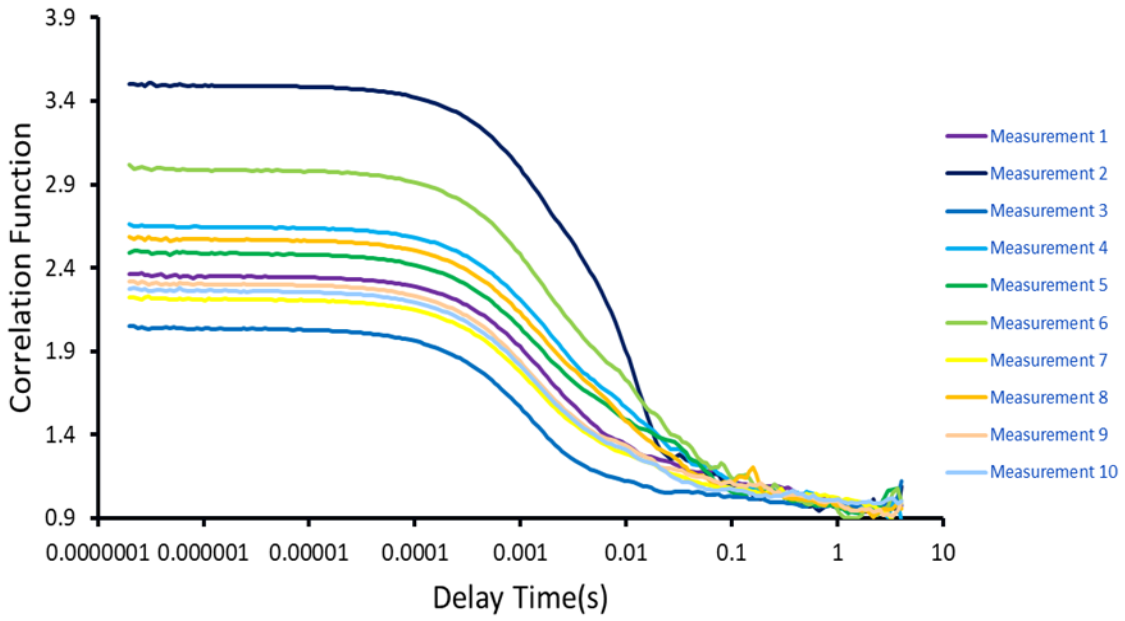

Figure S49 - Correlation function data for 10 DLS runs of **2** (5.56 mM) in an H_2O/5 % EtOH solution at 298 K.

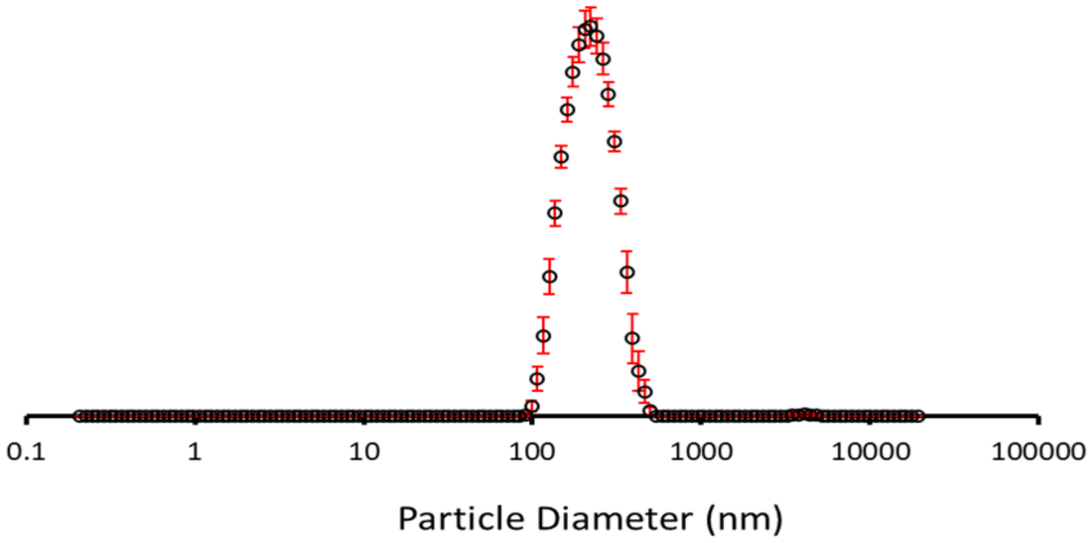

Figure S50 - The average intensity particle size distribution calculated (peak maxima = 422 nm) using 10 DLS runs for **3** (5.56 mM) in an H_2O/5 % EtOH solution at 298 K.

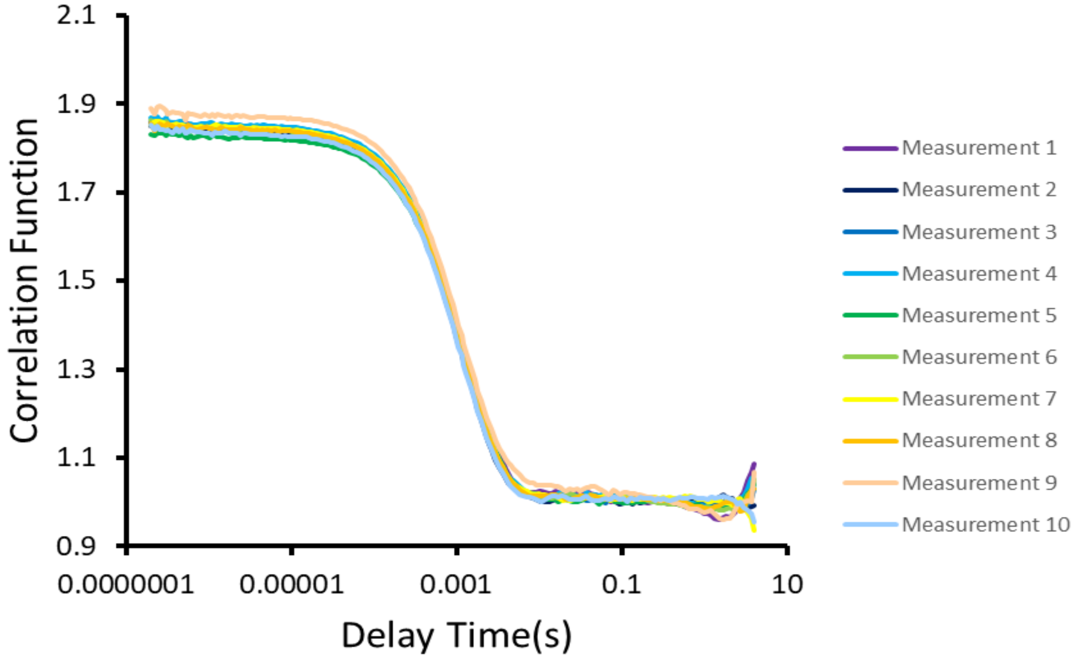

Figure S51 - Correlation function data for 10 DLS runs of **3** (5.56 mM) in an H_2O/5 % EtOH solution at 298 K.

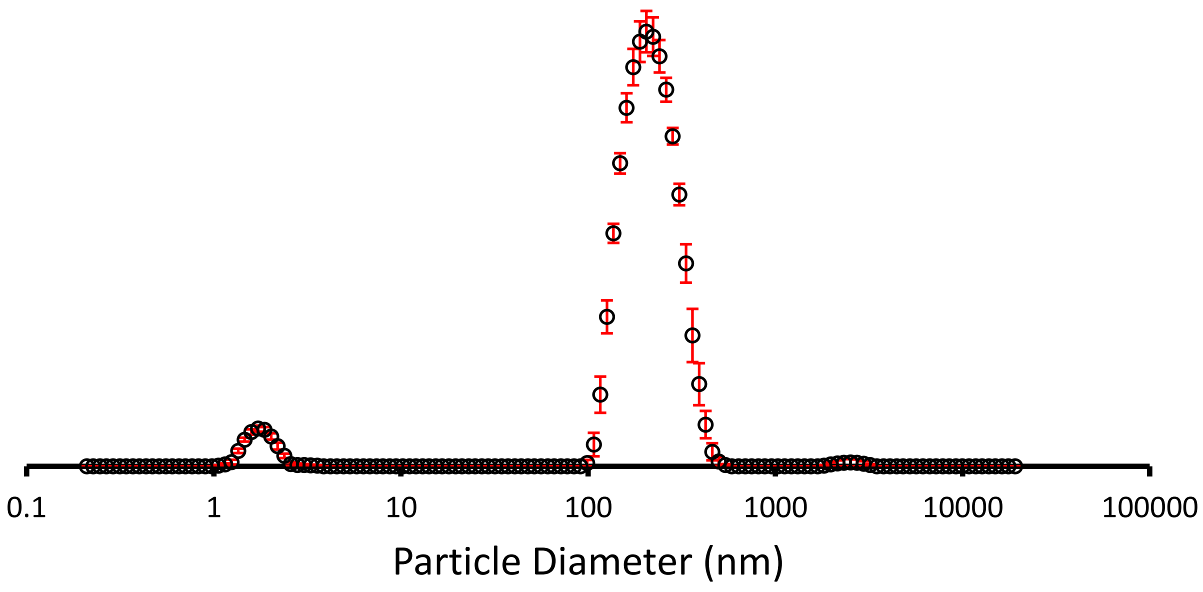

Figure S52 - The average intensity particle size distribution calculated (peak maxima = 3 and 222 nm) using 10 DLS runs for **4** (5.56 mM) in an H_2O/5 % EtOH solution at 298 K.

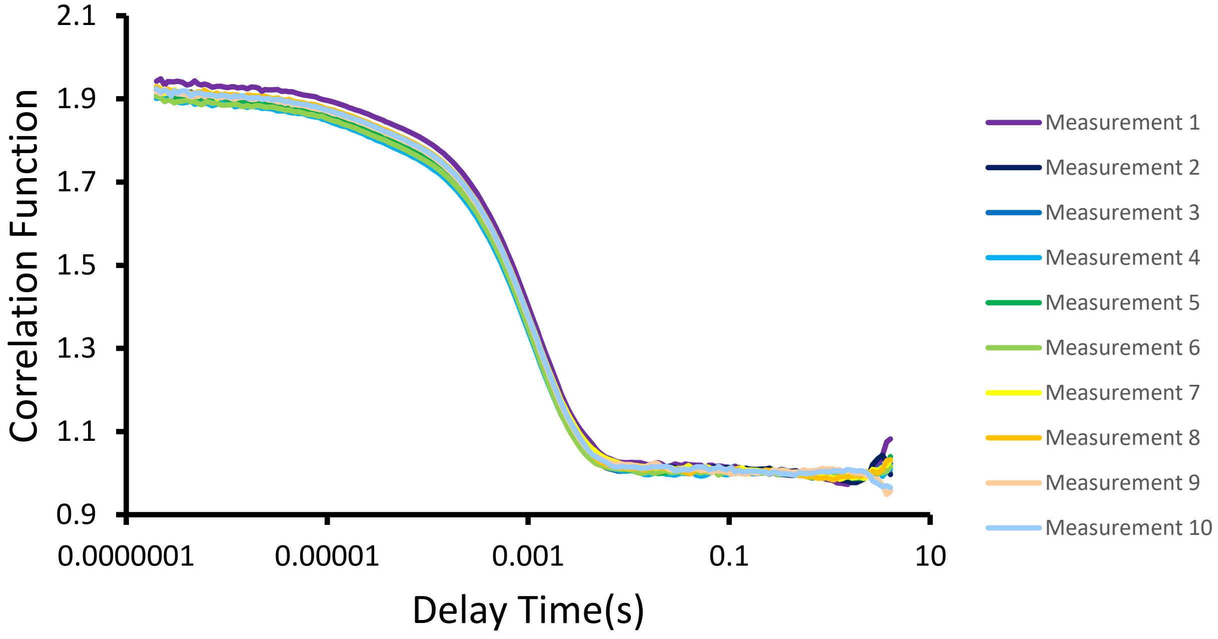

Figure S53 - Correlation function data for 10 DLS runs of **4** (5.56 mM) in an H_2O/5 % EtOH solution at 298 K.

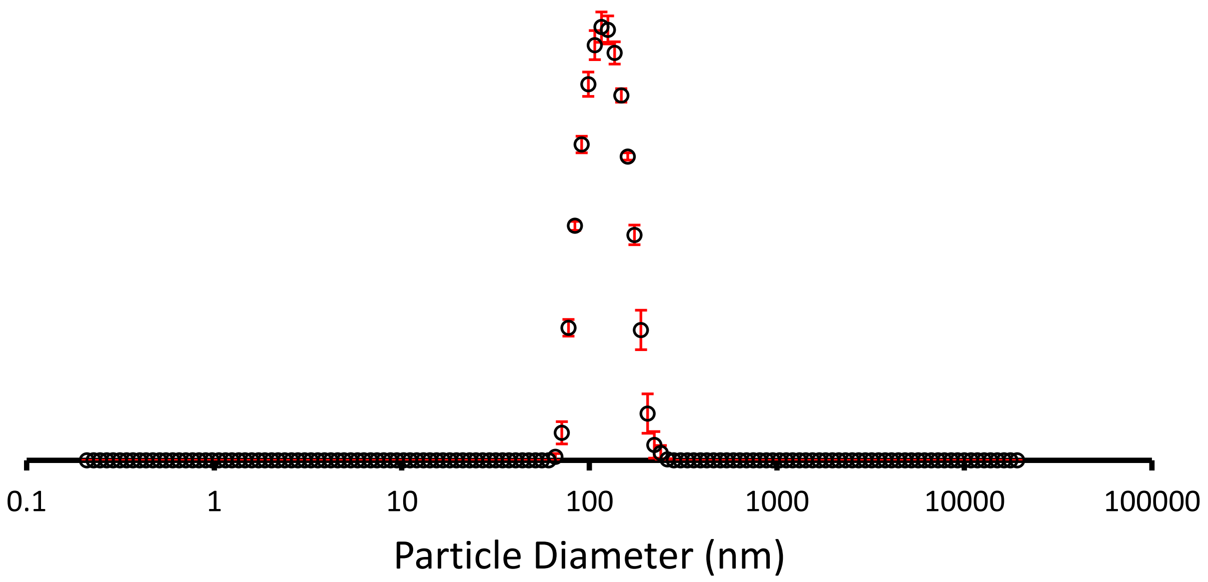

Figure S54 - The average intensity particle size distribution calculated (peak maxima = 125 nm) using 10 DLS runs for **5** (5.56 mM) in an H_2O/5 % EtOH solution at 298 K.

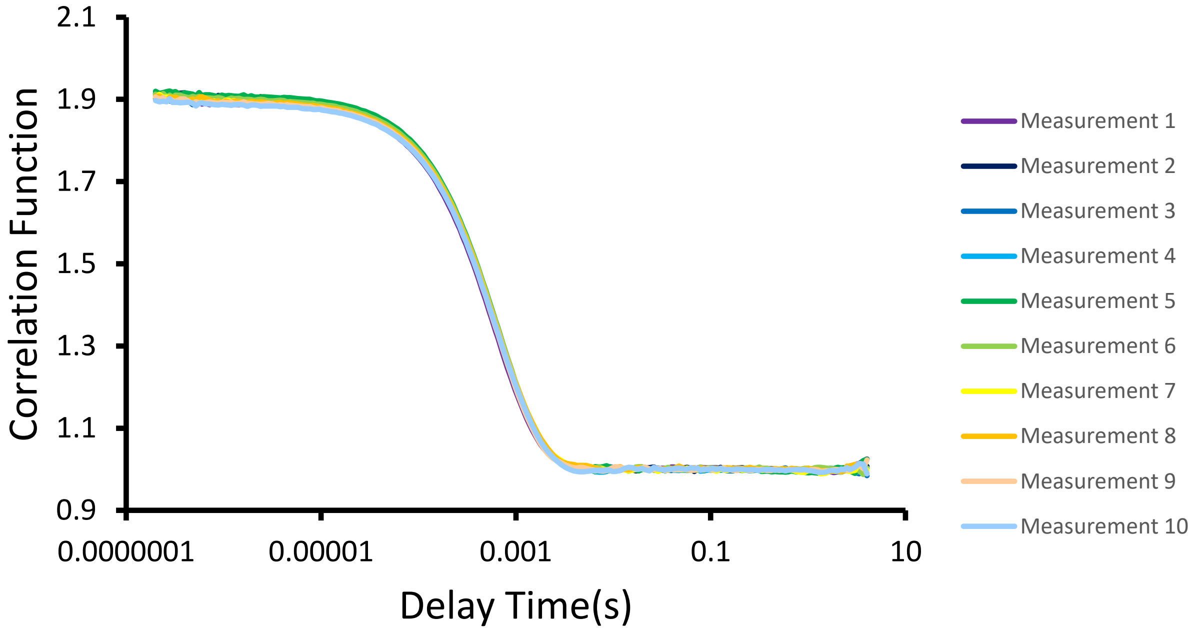

Figure S55 - Correlation function data for 10 DLS runs of **5** (5.56 mM) in an H_2O/5 % EtOH solution at 298 K.

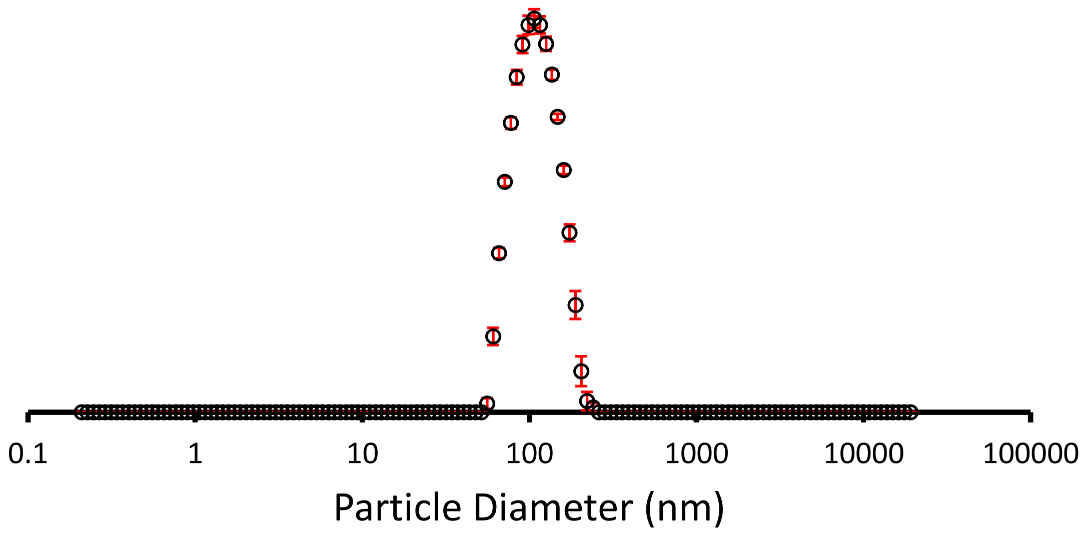

Figure S56 - The average intensity particle size distribution calculated (peak maxima = 116 nm) using 10 DLS runs for **6** (2.75 mM) in an H_2O/5 % EtOH solution at 298 K.

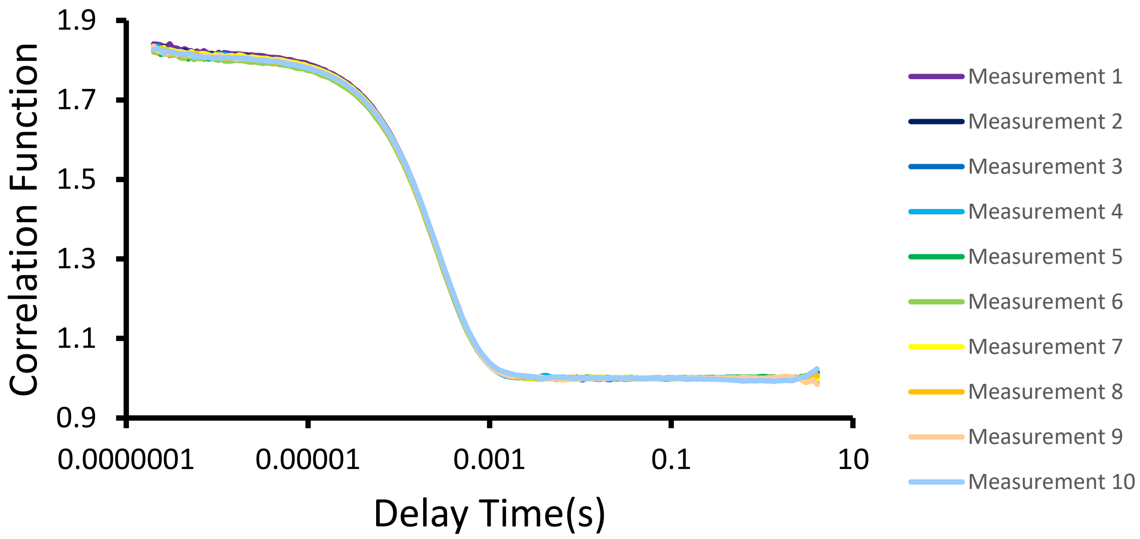

Figure S57 - Correlation function data for 10 DLS runs of **6** (2.75 mM) in an H_2O/5 % EtOH solution at 298 K.

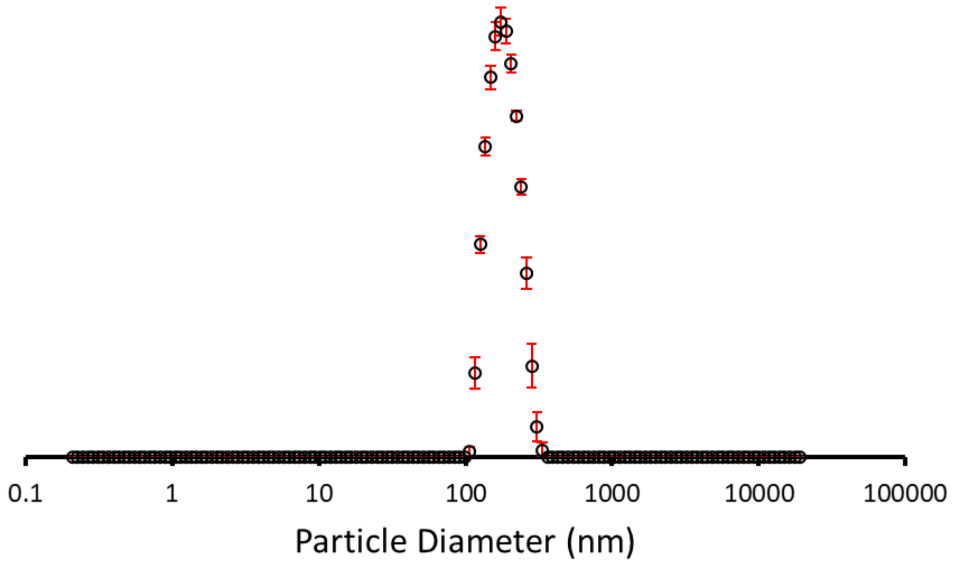

Figure S58 - The average intensity particle size distribution calculated (peak maxima = 186 nm) using 10 DLS runs for **7** (5.56 mM) in an H_2O/5 % EtOH solution at 298 K.

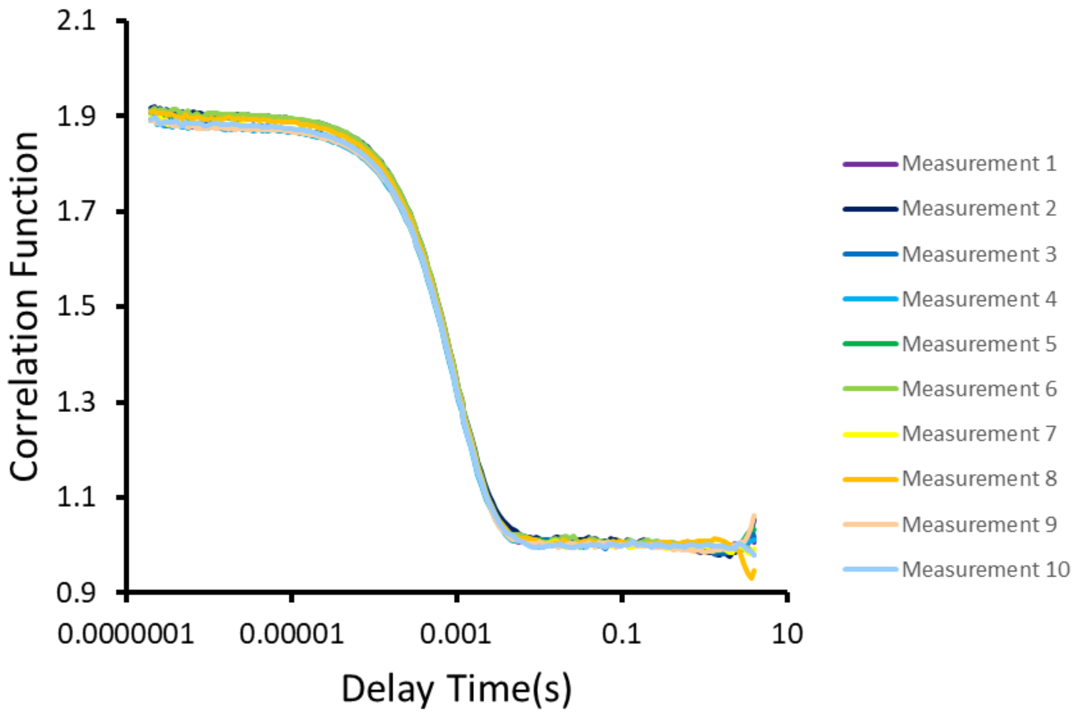

Figure S59 - Correlation function data for 10 DLS runs of **7** (5.56 mM) in an H_2O/5 % EtOH solution at 298 K.

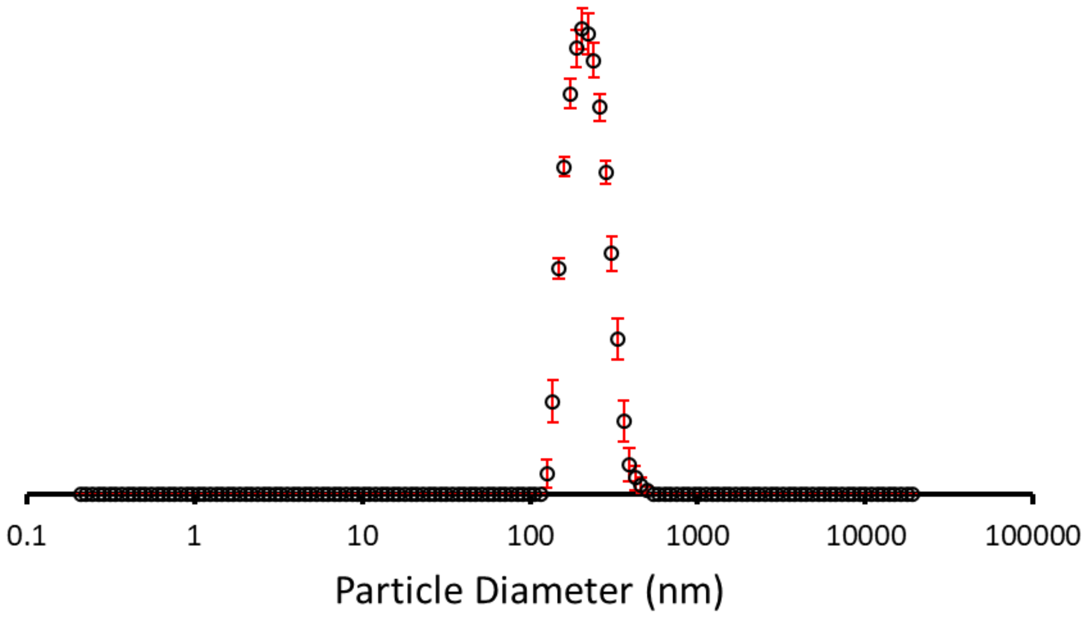

Figure S60 - The average intensity particle size distribution calculated (peak maxima = 225 nm) using 10 DLS runs for **8** (5.56 mM) in an H_2O/5 % EtOH solution at 298 K.

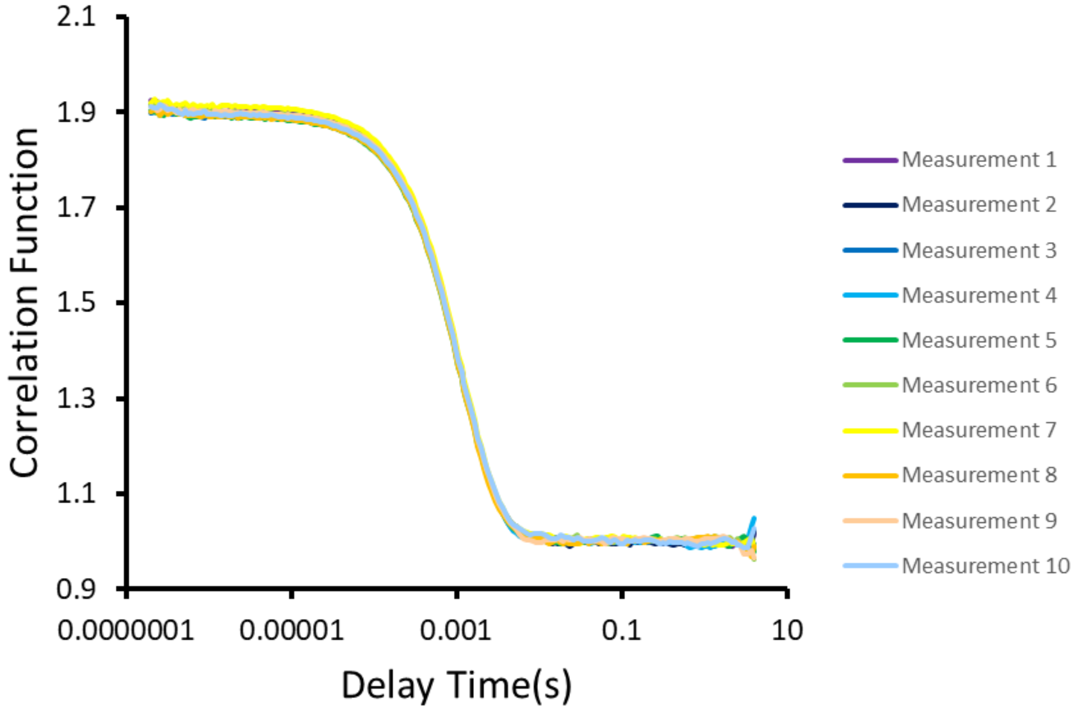

Figure S61 - Correlation function data for 10 DLS runs of **8** (5.56 mM) in an H_2O/5 % EtOH solution at 298 K.

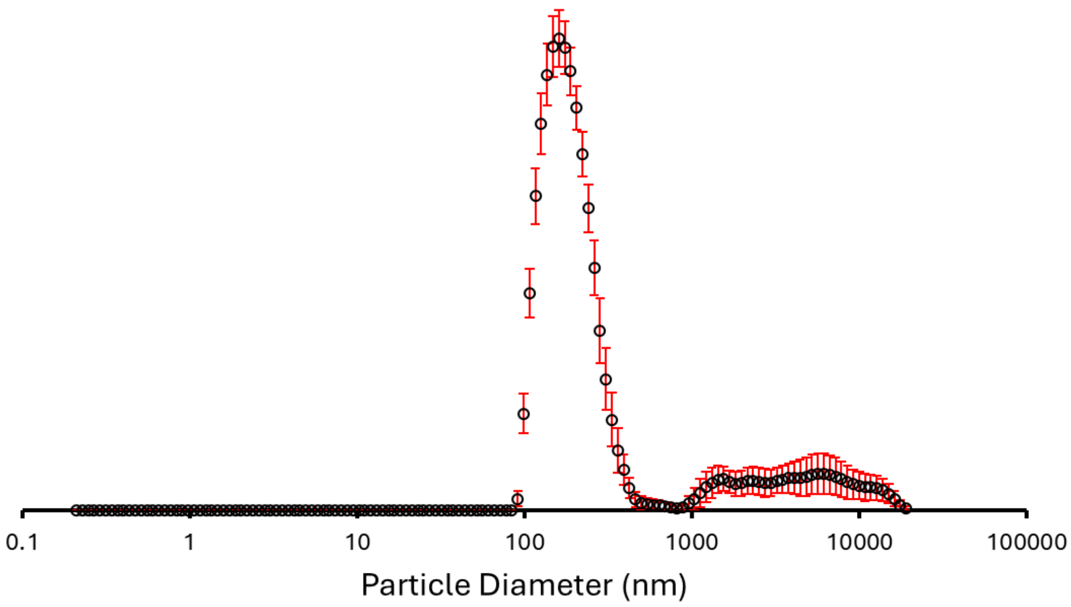

Figure S62 - The average intensity particle size distribution calculated (peak maxima = 364 and greater than 1000 nm) using 10 DLS runs for **9** (5.56 mM) in an H_2O/5 % EtOH solution at 298 K.

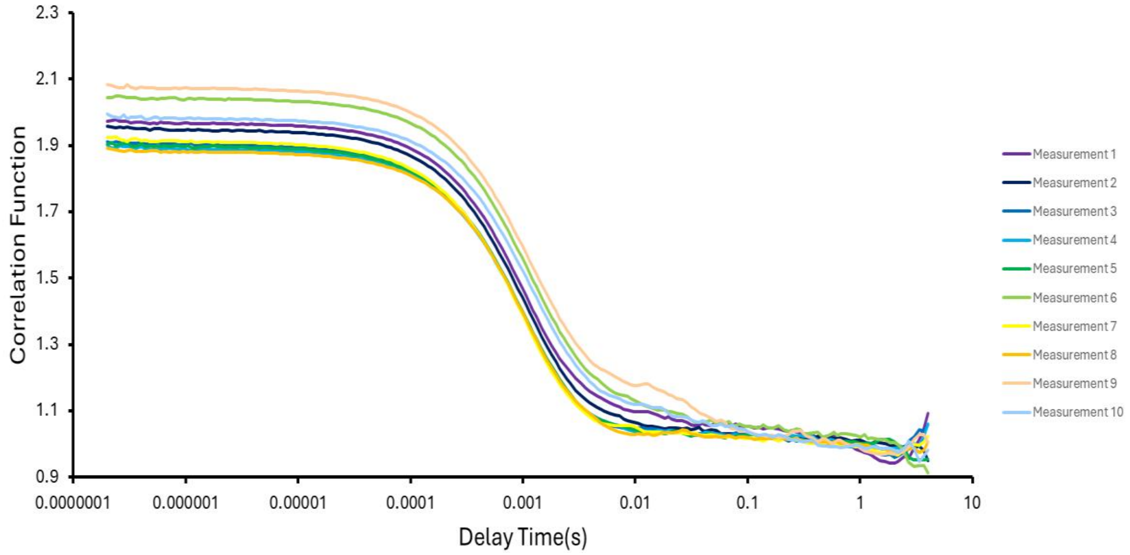

Figure S63 - Correlation function data for 10 DLS runs of **9** (5.56 mM) in an H_2O/5 % EtOH solution at 298 K.

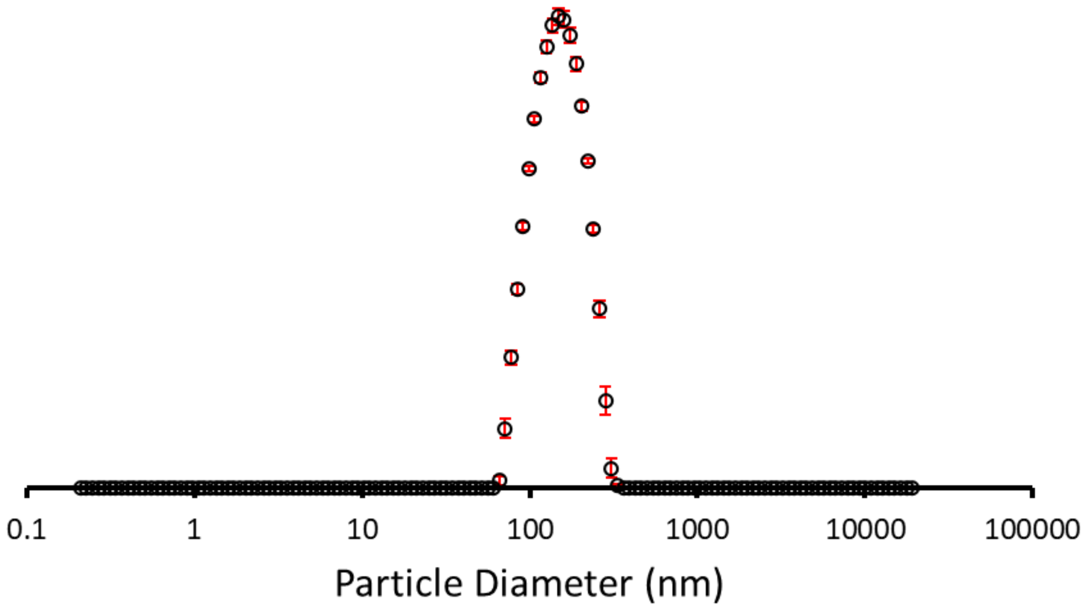

Figure S64 - The average intensity particle size distribution calculated (peak maxima = 155 nm) using 10 DLS runs for **10** (1.5 mM) in an H_2O/5 % EtOH solution at 298 K.

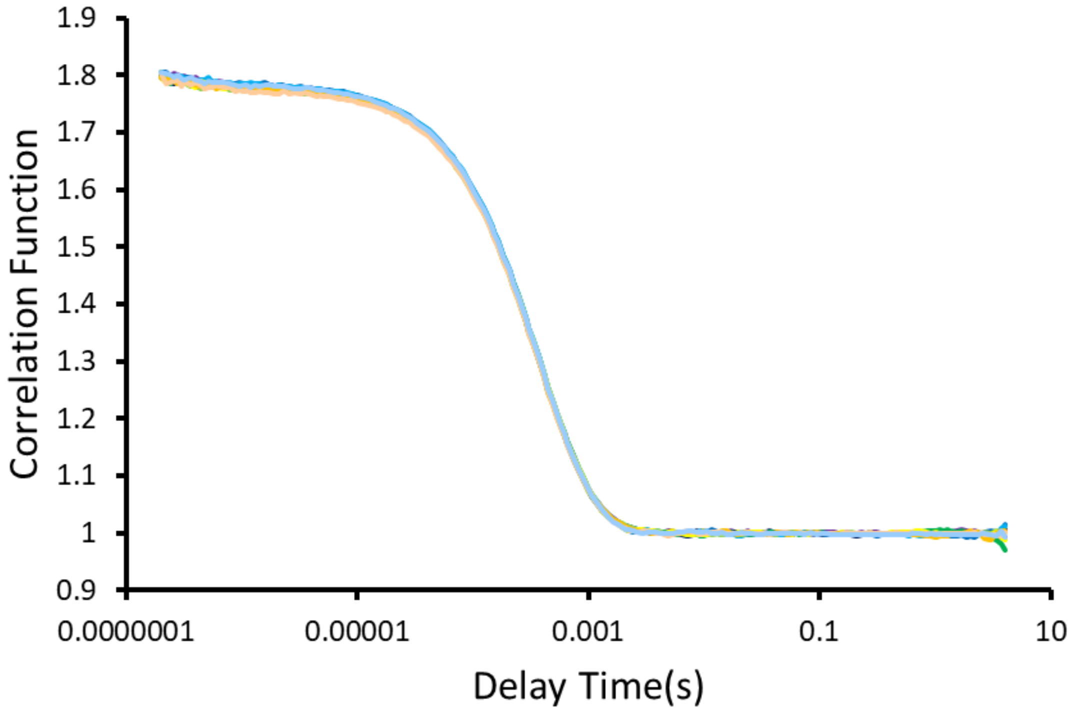

Figure S65 - Correlation function data for 10 DLS runs of **10** (1.5 mM) in an H_2O/5 % EtOH solution at 298 K.

Table S3 - Summary of average intensity particle size distribution data determined by DLS in H_2O/5 % EtOH at 298 K

SSA	Concentration (mM)	PDI	± Error	Peak 1 (nm)	Peak 2 (nm)
**1**	5.56	0.06	0.0003	188	> 1000
**2**	5.56	0.10	0.0032	409	0
**3**	5.56	0.04	0.0003	522	0
**4**	5.56	0.05	0.0004	3	222
**5**	5.56	0.02	0.0001	125	0
**6**	5.56	0.02	0.0003	116	0
**7**	5.56	0.01	0.0008	186	0
**8**	5.56	0.03	0.0005	225	0
**9**	5.56	0.05	0.0003	254	> 1000
**10**	1.50	0.03	0.0006	145	0

Section S6.2: DLS data in DMSO

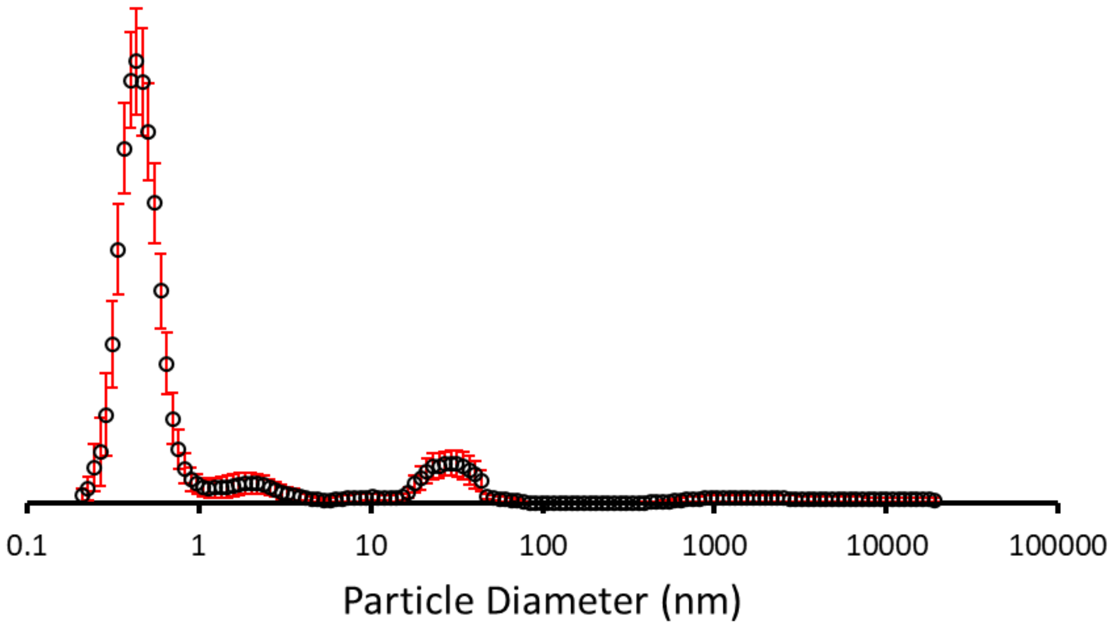

Figure S66 - The average intensity particle size distribution calculated (peak maxima = less than 1, 3 and 48 nm) using 10 DLS runs for **1** (112 mM) in a DMSO solution at 298 K.

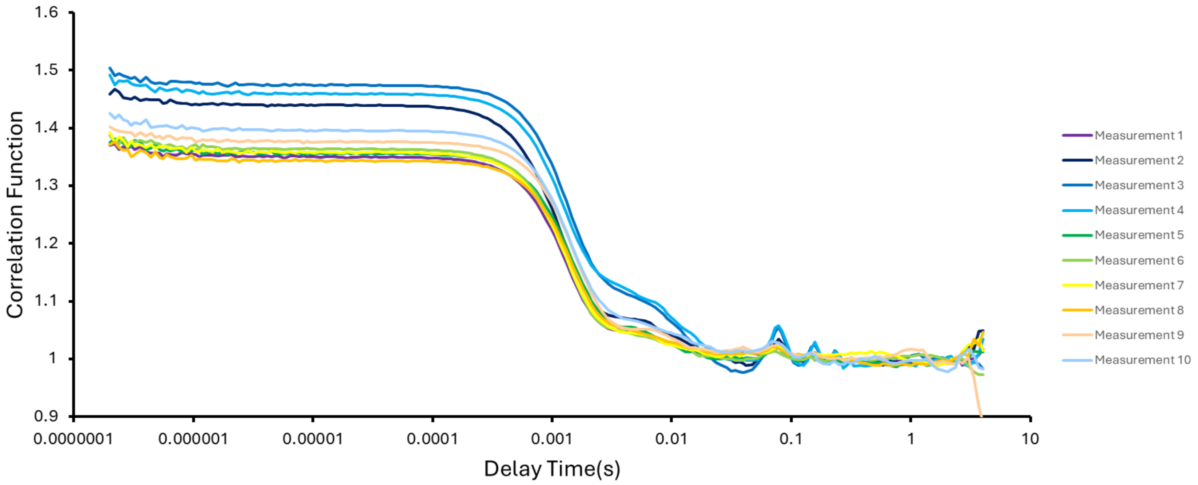

Figure S67 - Correlation function data for 10 DLS runs of **1** (112 mM) in a DMSO solution at 298 K.

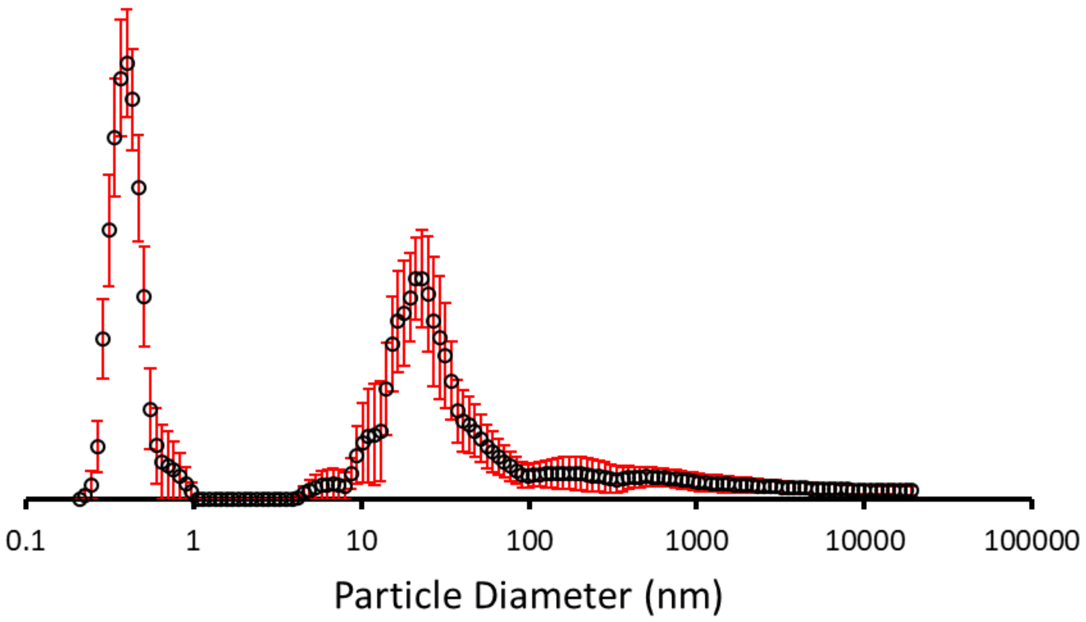

Figure S68 - The average intensity particle size distribution calculated (peak maxima = less than 1 and 38 nm) using 10 DLS runs for **2** (112 mM) in a DMSO solution at 298 K.

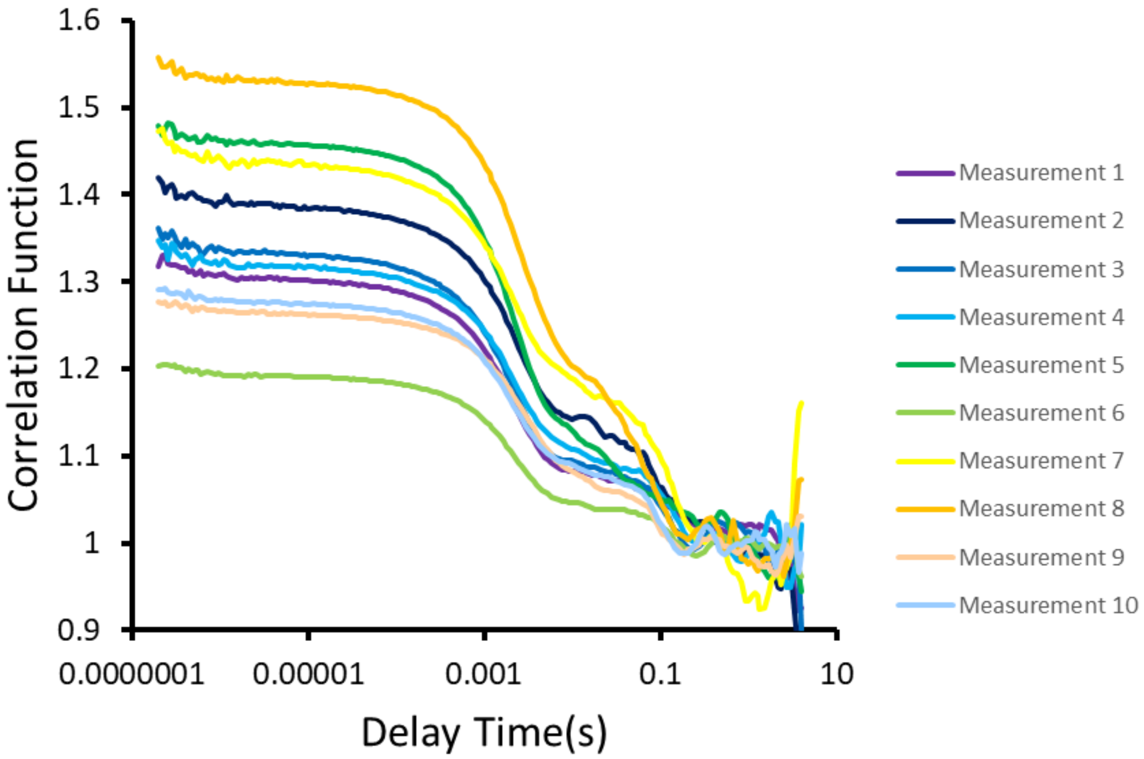

Figure S69 - Correlation function data for 10 DLS runs of **2** (112 mM) in a DMSO solution at 298 K.

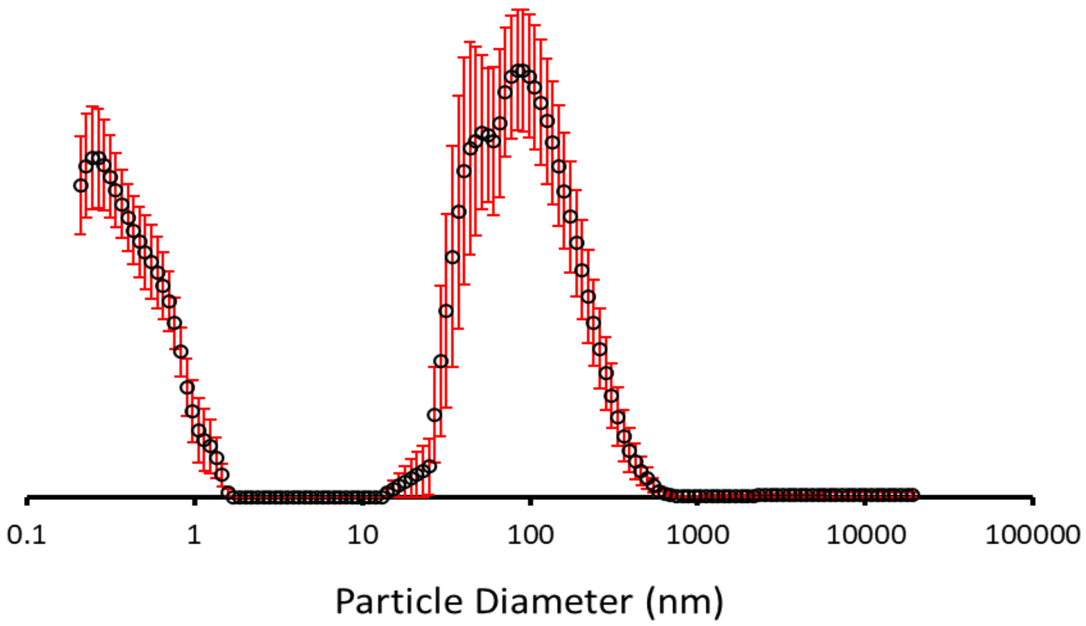

Figure S70 - The average intensity particle size distribution calculated (peak maxima = less than 1 and 100 nm) using 10 DLS runs for **3** (112 mM) in a DMSO solution at 298 K.

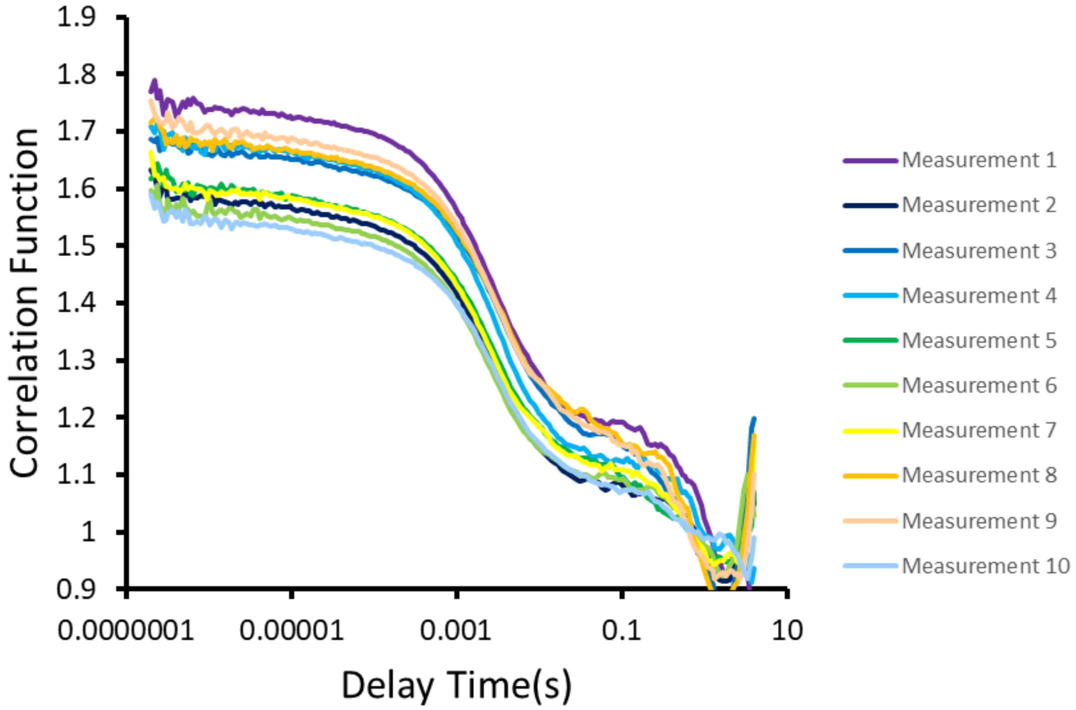

Figure S71 - Correlation function data for 10 DLS runs of **3** (112 mM) in a DMSO solution at 298 K.

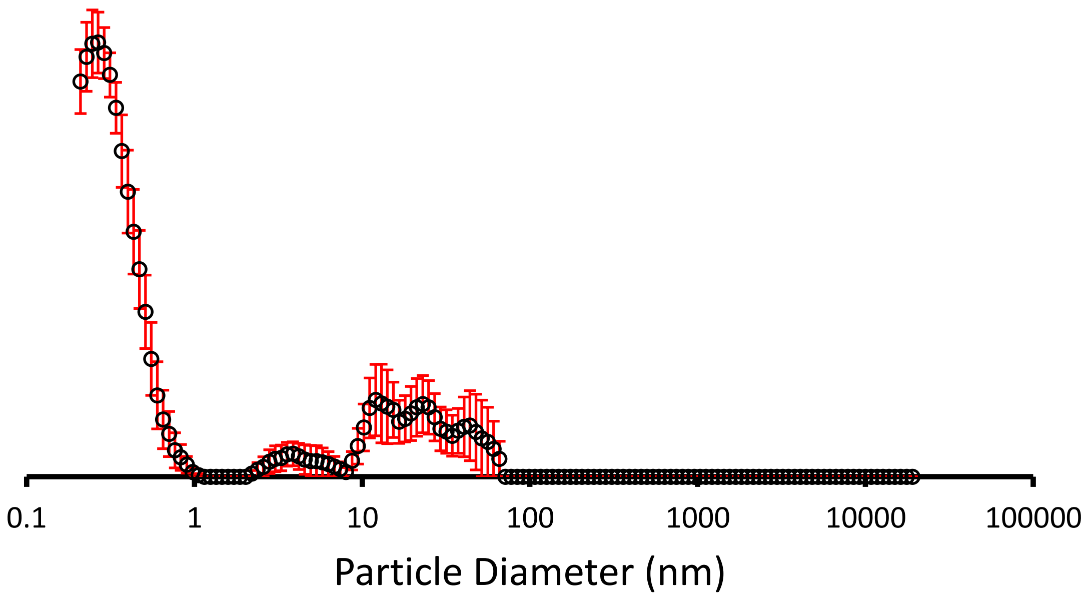

Figure S72 - The average intensity particle size distribution calculated (peak maxima = less than 1, 7 and between 10 - 100 nm) using 10 DLS runs for **4** (112 mM) in a DMSO solution at 298 K.

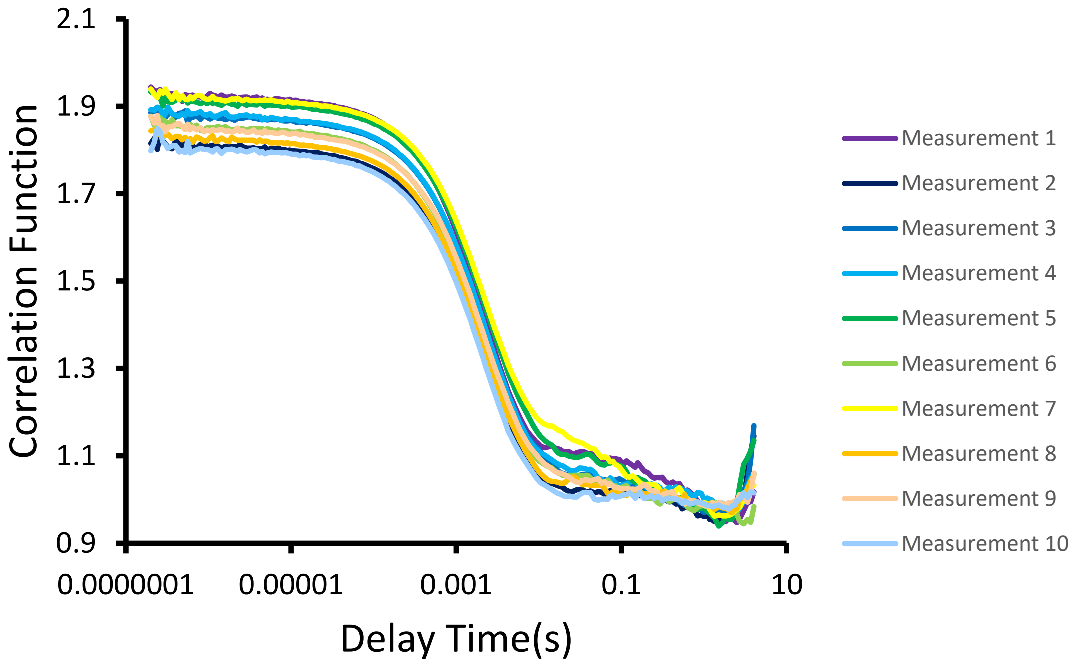

Figure S73 - Correlation function data for 10 DLS runs of **4** (112 mM) in a DMSO solution at 298 K.

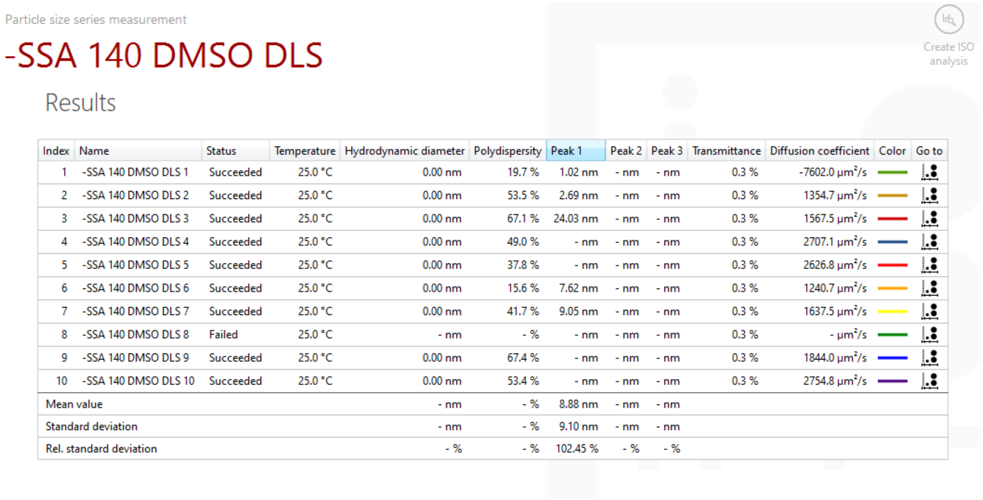

Particle size series measurement

-SSA 140 DMSO DLS

Results

Index	Name	Status	Temperature	Hydrodynamic diameter	Polydispersity	Peak 1	Peak 2	Peak 3	Transmittance	Diffusion coefficient	Color	Go to
1	-SSA 140 DMSO DLS 1	Succeeded	25.0 °C	0.00 nm	19.7 %	1.02 nm	- nm	- nm	0.3 %	-7602.0 μm²/s		
2	-SSA 140 DMSO DLS 2	Succeeded	25.0 °C	0.00 nm	53.5 %	2.69 nm	- nm	- nm	0.3 %	1354.7 μm²/s		
3	-SSA 140 DMSO DLS 3	Succeeded	25.0 °C	0.00 nm	67.1 %	24.03 nm	- nm	- nm	0.3 %	1567.5 μm²/s		
4	-SSA 140 DMSO DLS 4	Succeeded	25.0 °C	0.00 nm	49.0 %	- nm	- nm	- nm	0.3 %	2707.1 μm²/s		
5	-SSA 140 DMSO DLS 5	Succeeded	25.0 °C	0.00 nm	37.8 %	- nm	- nm	- nm	0.3 %	2626.8 μm²/s		
6	-SSA 140 DMSO DLS 6	Succeeded	25.0 °C	0.00 nm	15.6 %	7.62 nm	- nm	- nm	0.3 %	1240.7 μm²/s		
7	-SSA 140 DMSO DLS 7	Succeeded	25.0 °C	0.00 nm	41.7 %	9.05 nm	- nm	- nm	0.3 %	1637.5 μm²/s		
8	-SSA 140 DMSO DLS 8	Failed	25.0 °C	- nm	- %	- nm	- nm	- nm	0.3 %	- μm²/s		
9	-SSA 140 DMSO DLS 9	Succeeded	25.0 °C	0.00 nm	67.4 %	- nm	- nm	- nm	0.3 %	1844.0 μm²/s		
10	-SSA 140 DMSO DLS 10	Succeeded	25.0 °C	0.00 nm	53.4 %	- nm	- nm	- nm	0.3 %	2754.8 μm²/s		
Mean value				- nm	- %	8.88 nm	- nm	- nm				
Standard deviation				- nm	- %	9.10 nm	- nm	- nm				
Rel. standard deviation				- %	- %	102.45 %	- %	- %				

Figure S74 - The average intensity particle size distribution using 10 DLS runs for **5** (112 mM) in a DMSO solution at 298 K could not be determined due to the instability of the aggregate sizes in this solvent system.

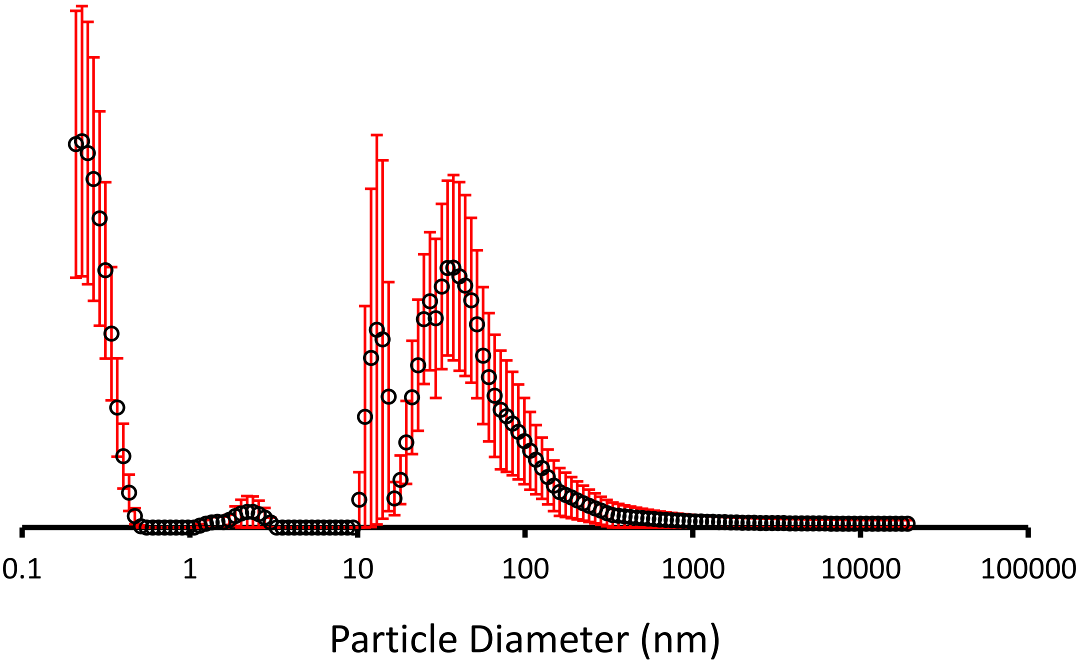

Figure S75 - The average intensity particle size distribution calculated (peak maxima = less than 1 and 67 nm) using 10 DLS runs for **6** (112 mM) in a DMSO solution at 298 K.

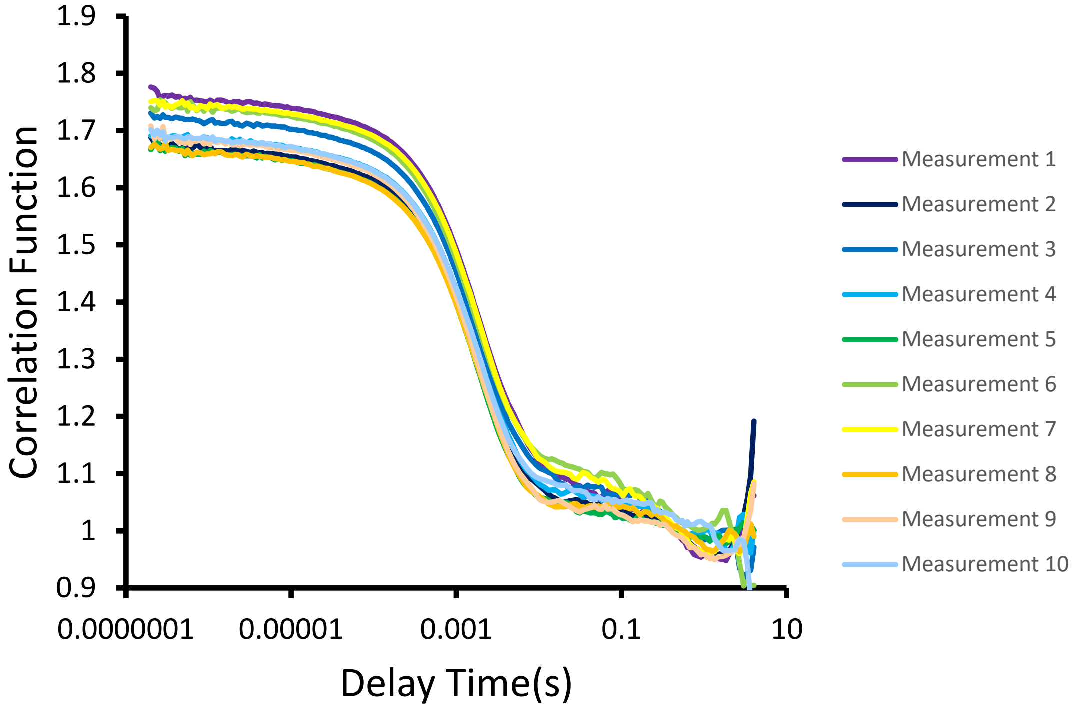

Figure S76 - Correlation function data for 10 DLS runs of **6** (112 mM) in a DMSO solution at 298 K.

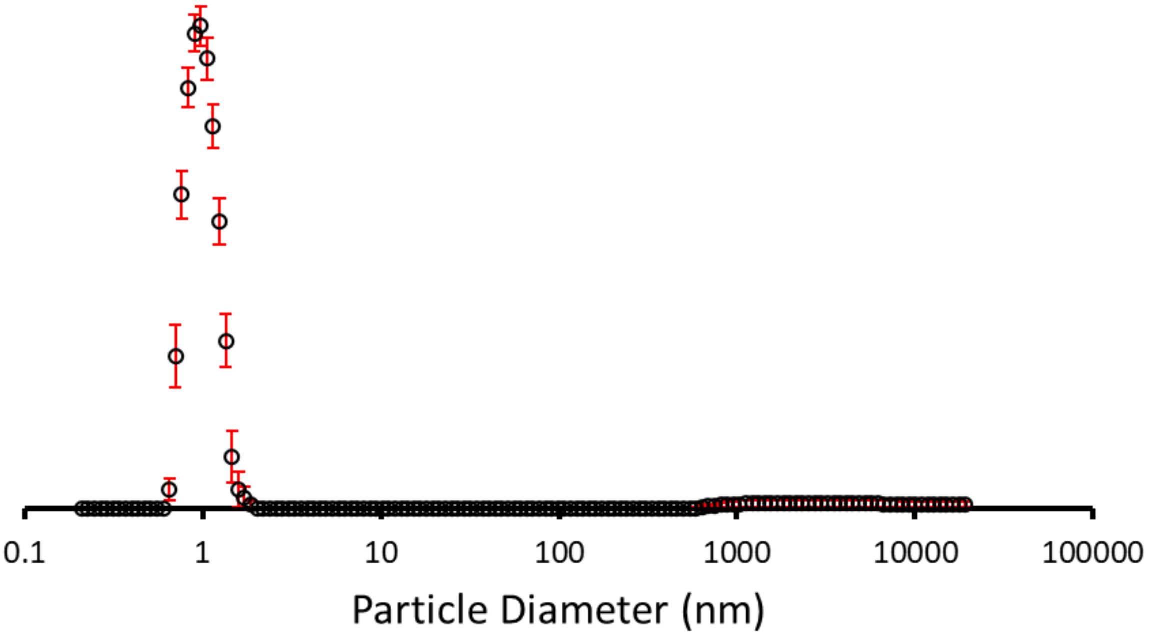

Figure S77 - The average intensity particle size distribution calculated (peak maxima = 1 nm) using 10 DLS runs for **8** (112 mM) in a DMSO solution at 298 K.

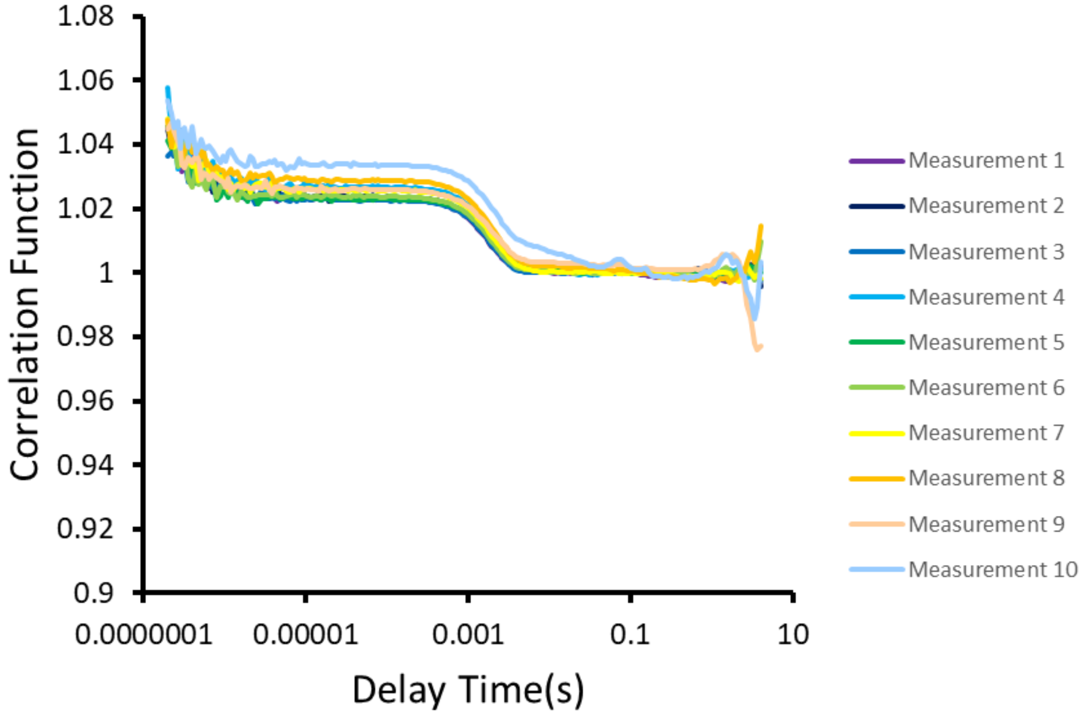

Figure S78 - Correlation function data for 10 DLS runs of **8** (112 mM) in a DMSO solution at 298 K.

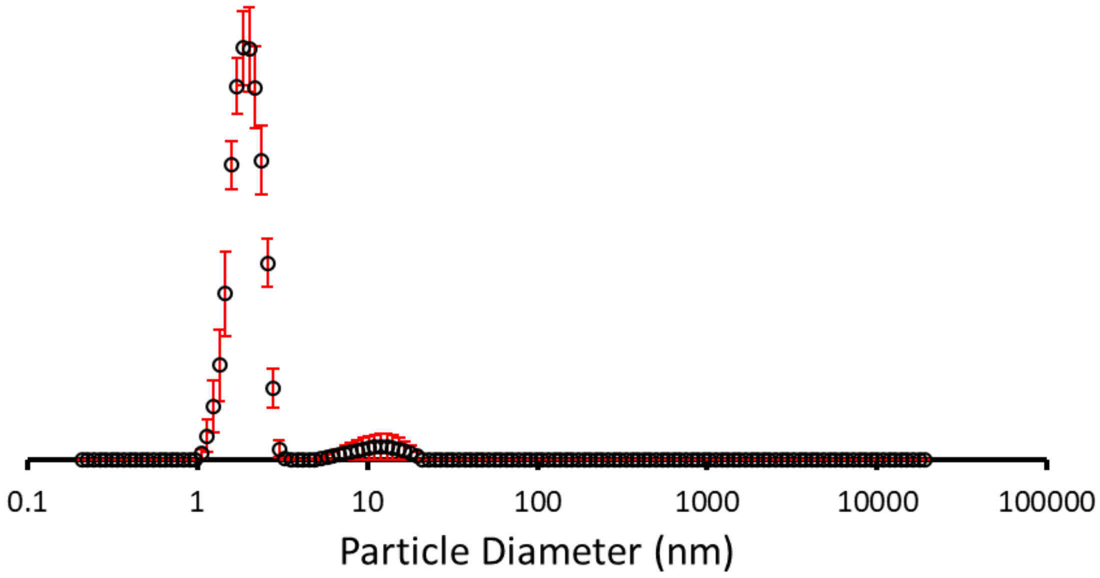

Figure S79 - The average intensity particle size distribution calculated (peak maxima = 2 nm) using 10 DLS runs for **9** (112 mM) in a DMSO solution at 298 K.

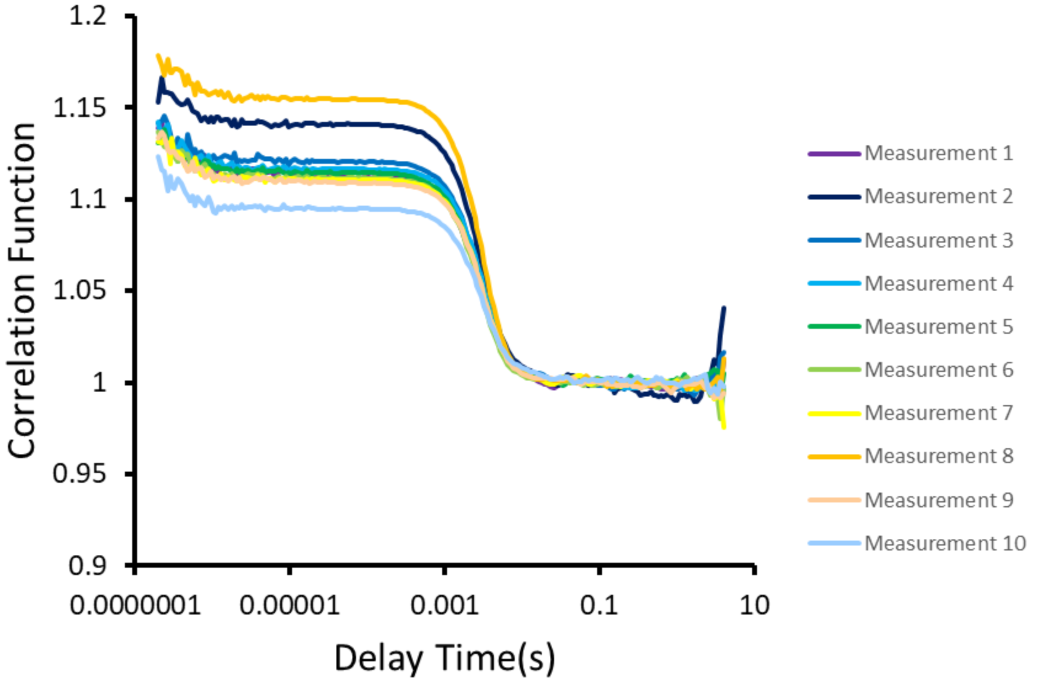

Figure S80 - Correlation function data for 10 DLS runs of **9** (112 mM) in a DMSO solution at 298 K.

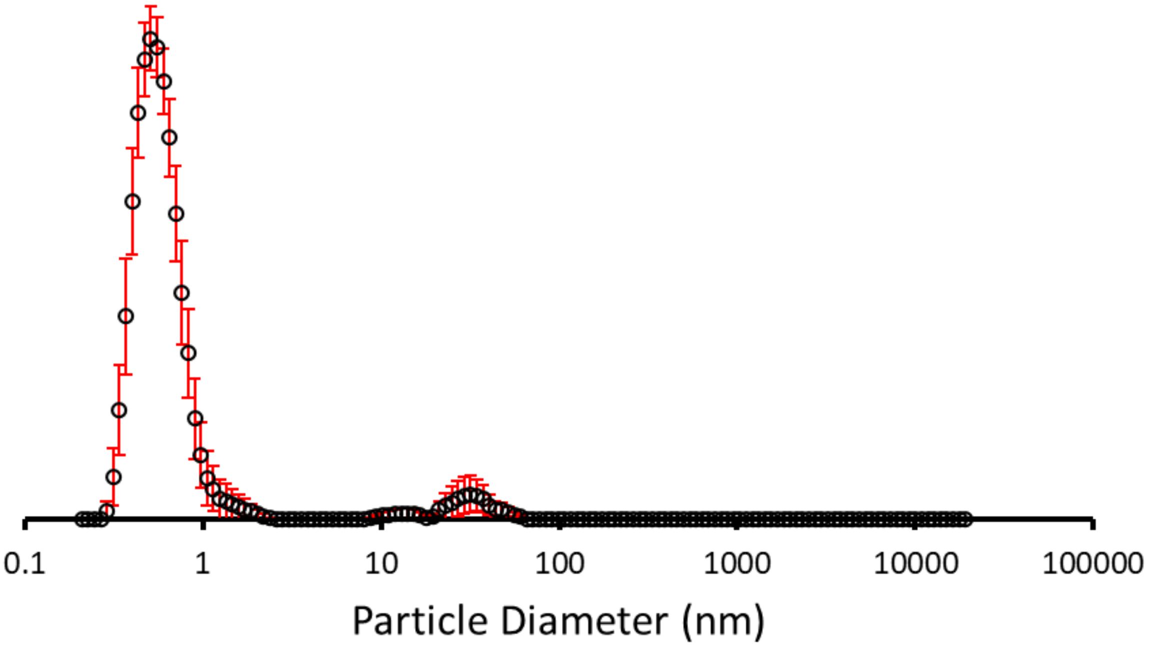

Figure S81 - The average intensity particle size distribution calculated (peak maxima = less than 1 nm) using 10 DLS runs for **10** (112 mM) in a DMSO solution at 298 K.

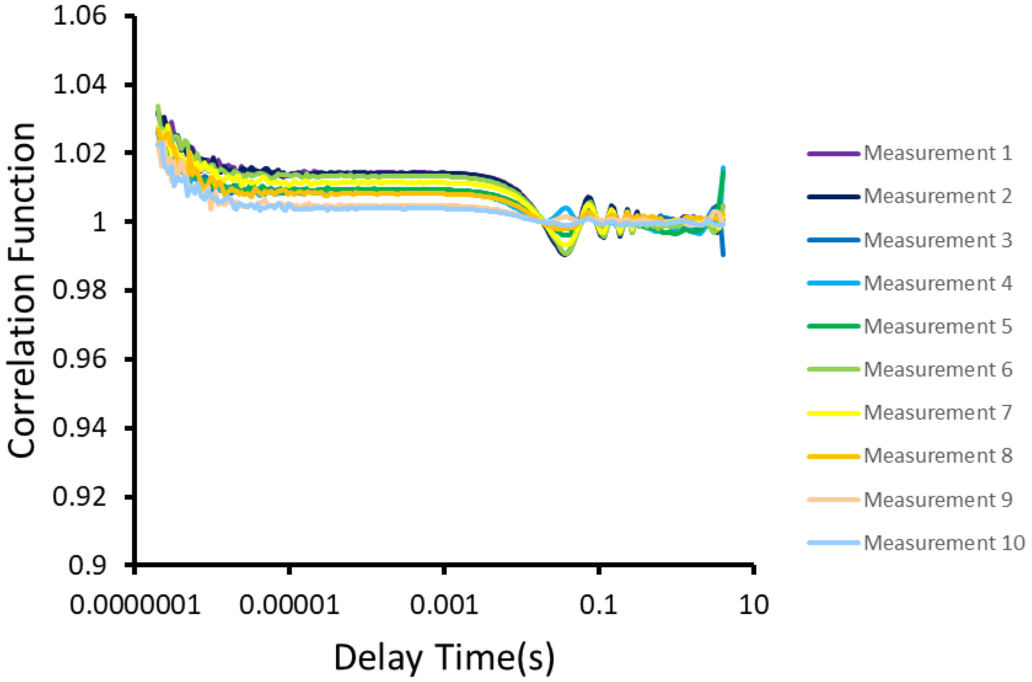

Figure S82 - Correlation function data for 10 DLS runs of **10** (112 mM) in a DMSO solution at 298 K.

Table S4 - Summary of average intensity particle size distribution data determined by DLS in DMSO at 298 K.

SSA	Concentration (mM)	PDI	± Error	Peak 1 (nm)	Peak 2 (nm)	Peak 3 (nm)
**1**	112	542.15	169.5039	< 1	3	48
**2**	112	0.10	0.0008	< 1	38	
**3**	112	0.10	0.0001	< 1	100	
**4**	112	0.07	0.00022	< 1	7	10 - 100
**5**	112	*a*	*a*	*a*	*a*	*a*
**6**	112	0.07	0.000061	< 1	67	
**7**	*a*	*a*	*a*	*a*	*a*	*a*
**8**	112	0.03	0.0016	1		
**9**	112	205.70	26.0555	2		
**10**	112	3.62	0.85605	< 1		

a - DLS data not collected for this compound in DMSO due to insufficient evidence of higher order aggregation in this solvent system or size instability that cannot be analysed.

Section S7: Stability and Surface Tension Data

Section S7.1: Zeta potential data

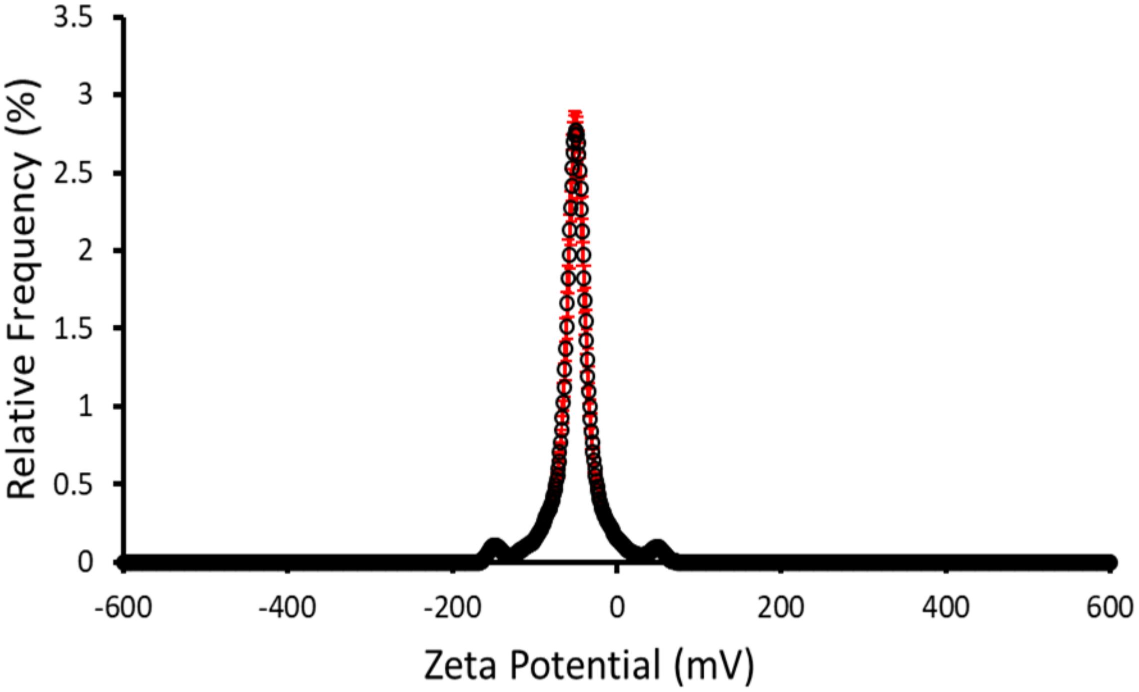

Figure S83 - The average zeta potential distribution calculated using 10 runs for **1** (5.56 mM) in an H_2O/5 % EtOH solution at 298 K. Average measurement value -48 mV.

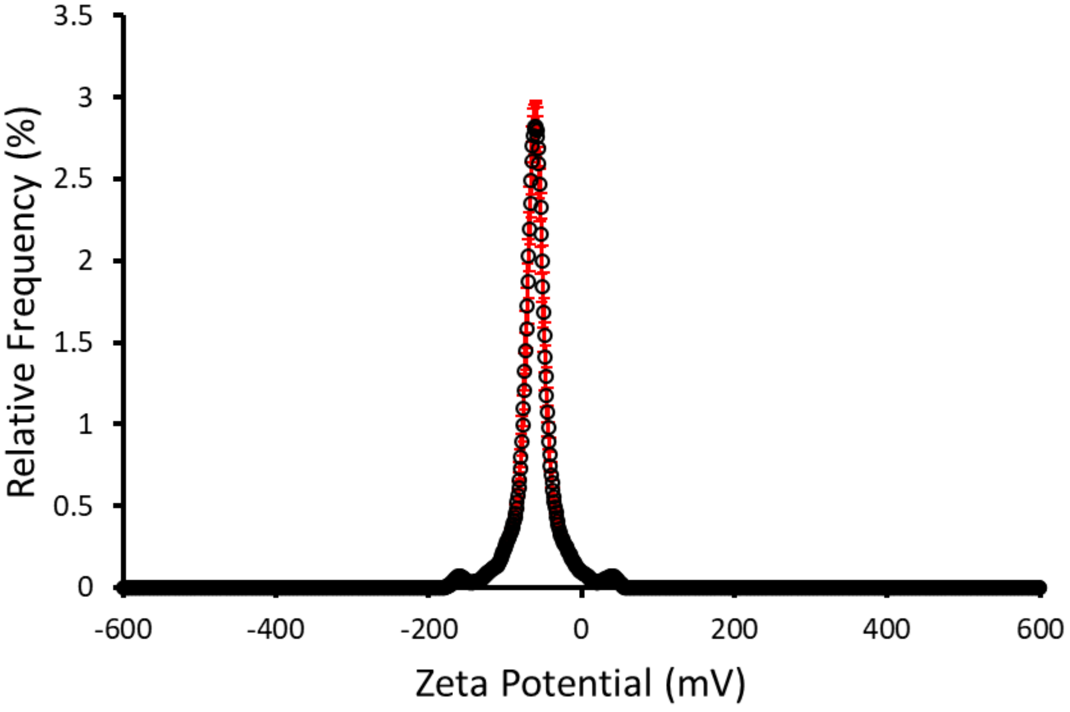

Figure S84 - The average zeta potential distribution calculated using 10 runs for **2** (5.56 mM) in an H_2O/5 % EtOH solution at 298 K. Average measurement value -64 mV.

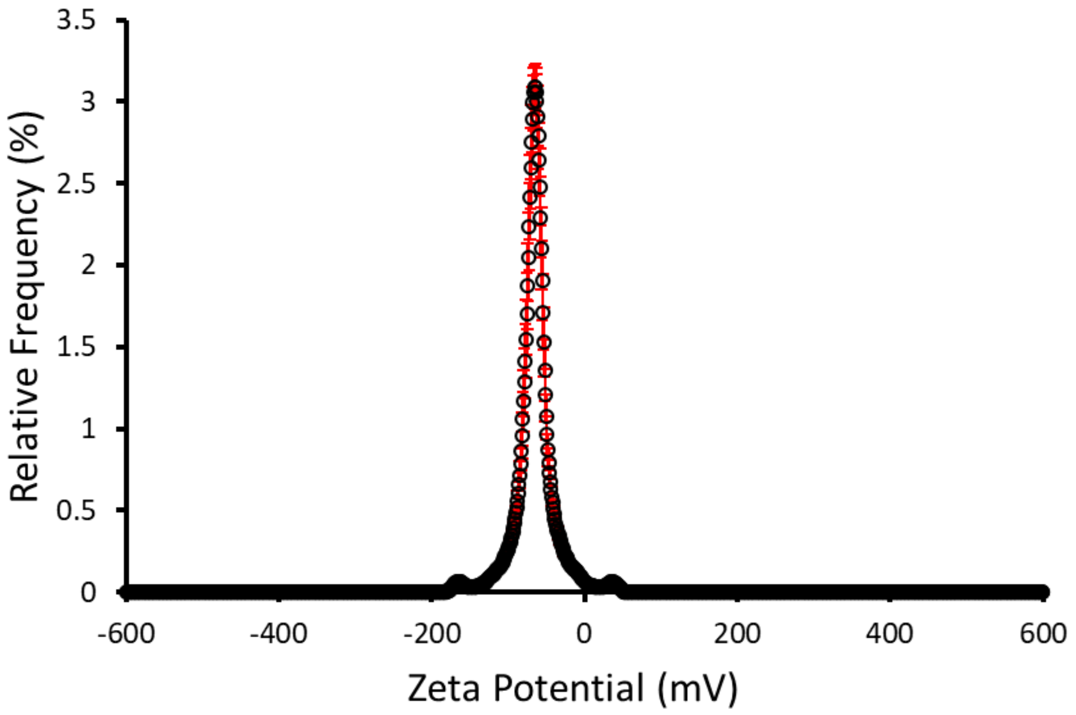

Figure S85 - The average zeta potential distribution calculated using 10 runs for **3** (5.56 mM) in an H_2O/5 % EtOH solution at 298 K. Average measurement value -66 mV.

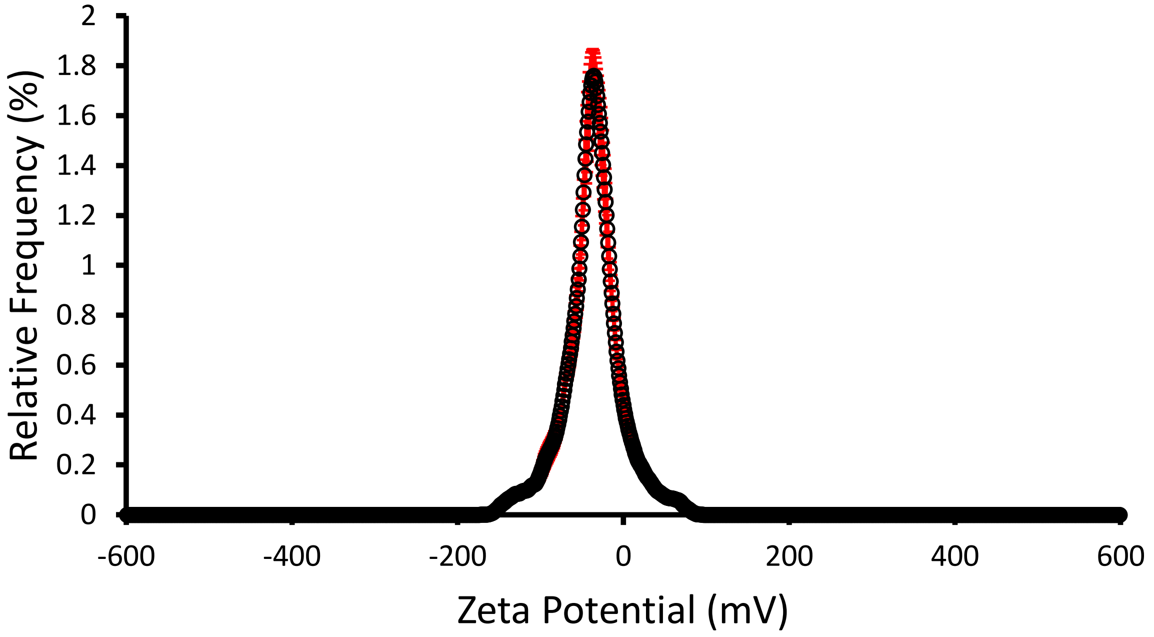

Figure S86 - The average zeta potential distribution calculated using 10 runs for **4** (5.56 mM) in an H_2O/5 % EtOH solution at 298 K. Average measurement value -47 mV.

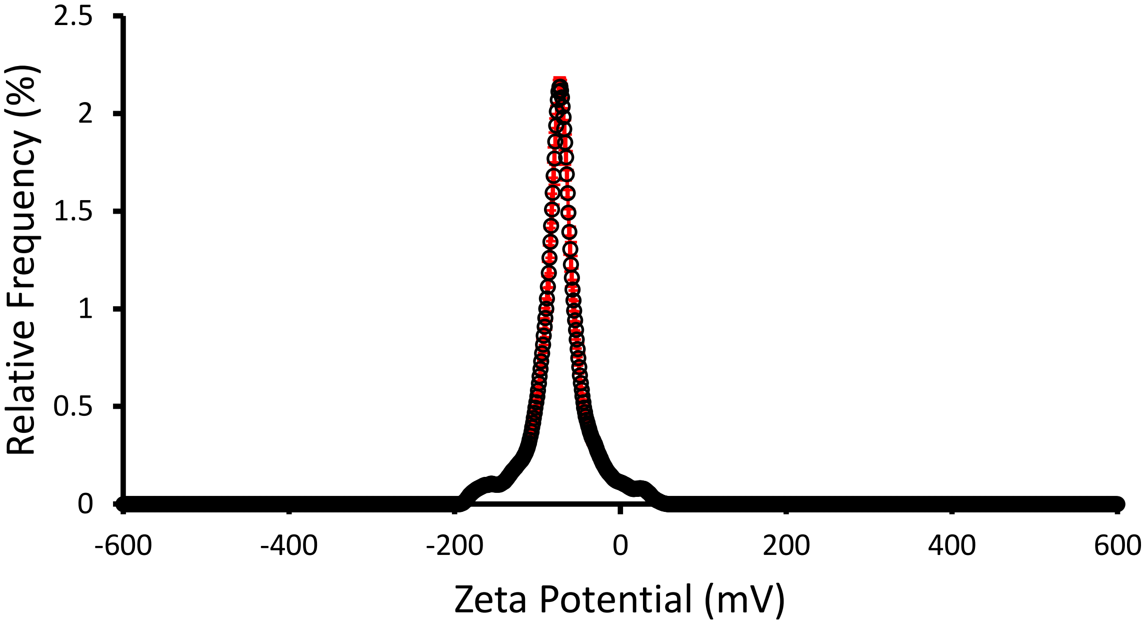

Figure S87 - The average zeta potential distribution calculated using 10 runs for **5** (5.56 mM) in an H_2O/5 % EtOH solution at 298 K. Average measurement value -71 mV.

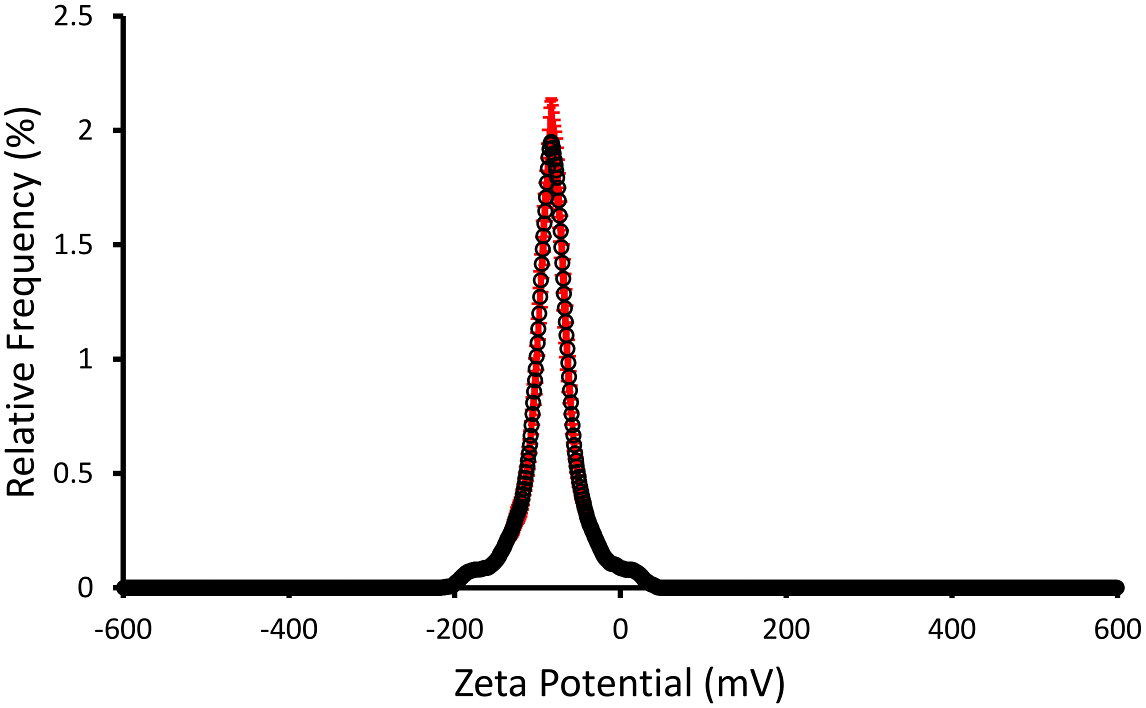

Figure S88 - The average zeta potential distribution calculated using 10 runs for **6** (2.75 mM) in an H_2O/5 % EtOH solution at 298 K. Average measurement value -71 mV.

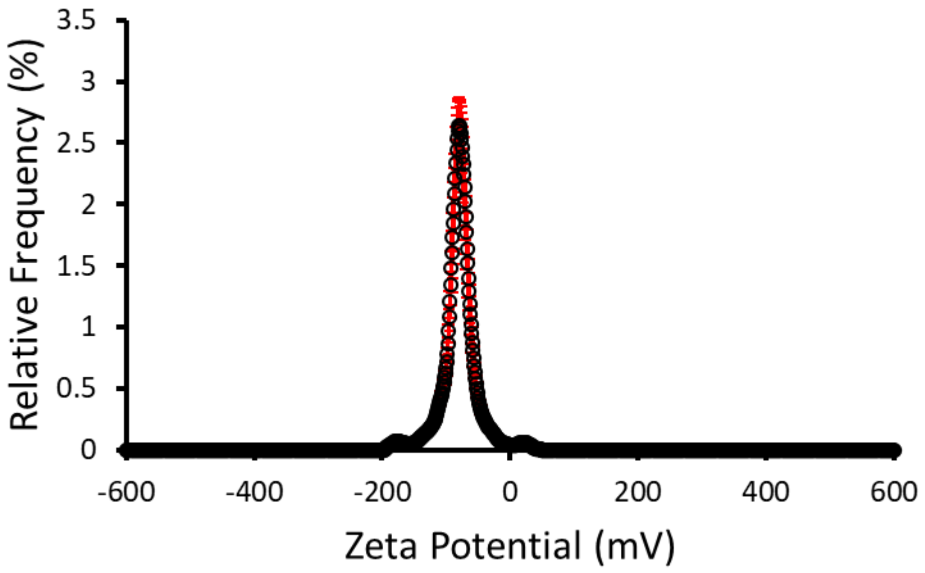

Figure S89 - The average zeta potential distribution calculated using 10 runs for **7** (5.56 mM) in an H_2O/5 % EtOH solution at 298 K. Average measurement value -82 mV.

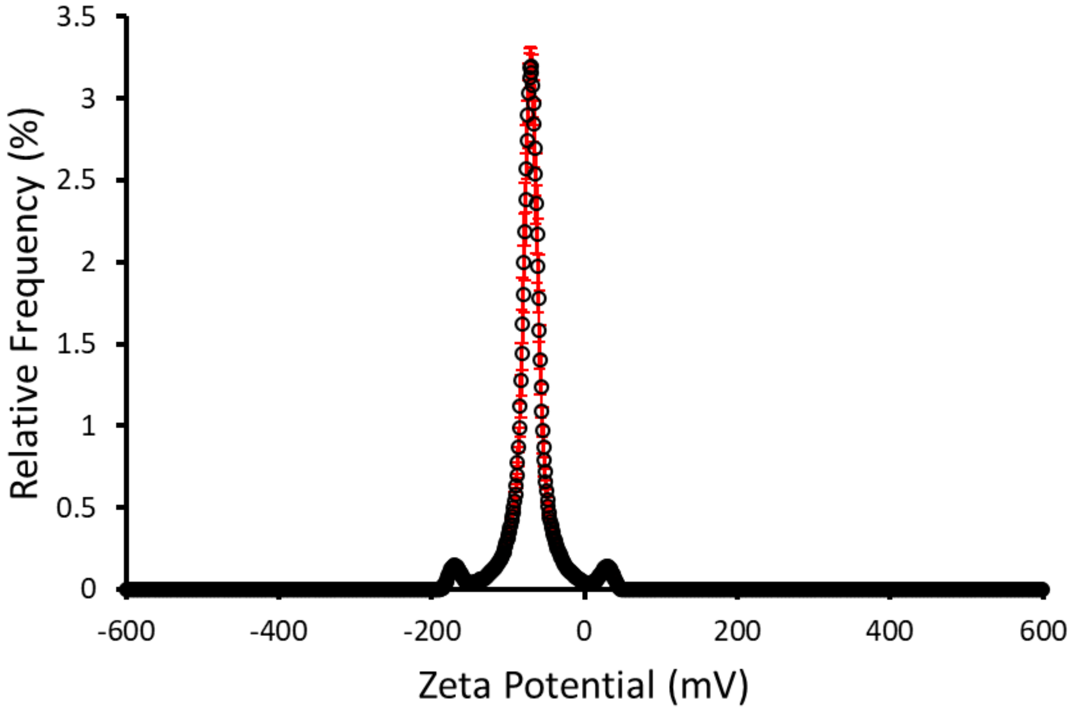

Figure S90 - The average zeta potential distribution calculated using 10 runs for **8** (5.56 mM) in an H_2O/5 % EtOH solution at 298 K. Average measurement value -72 mV.

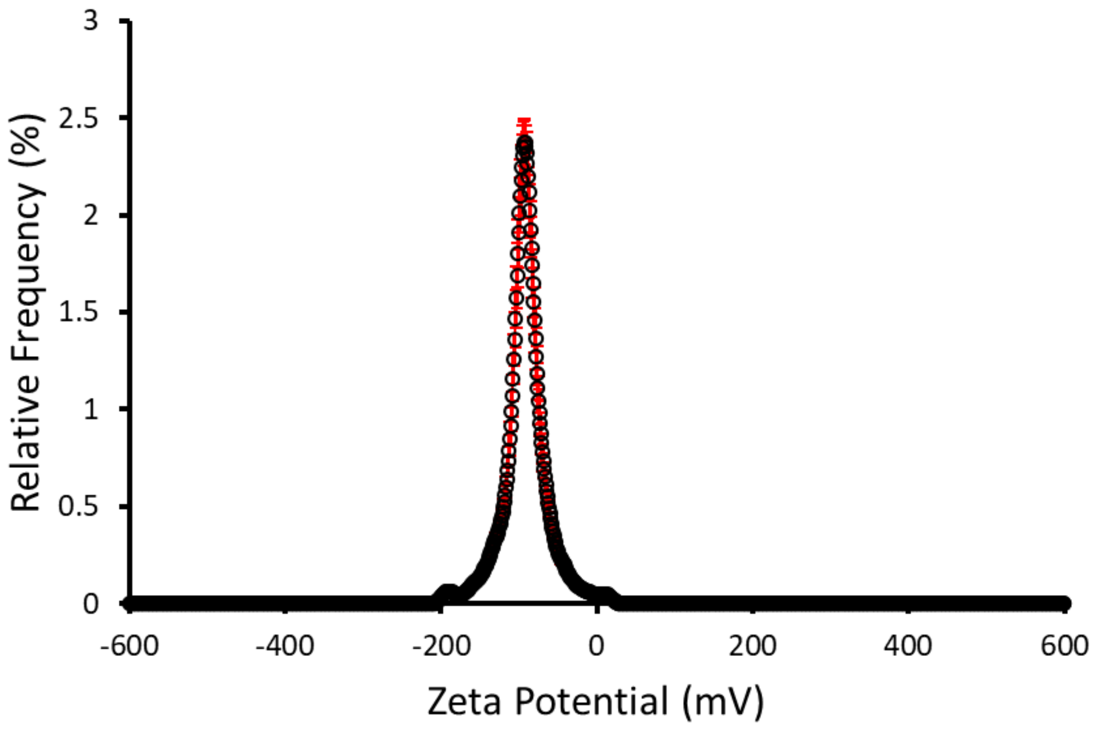

Figure S91 - The average zeta potential distribution calculated using 10 runs for **9** (5.56 mM) in an H_2O/5 % EtOH solution at 298 K. Average measurement value -92 mV.

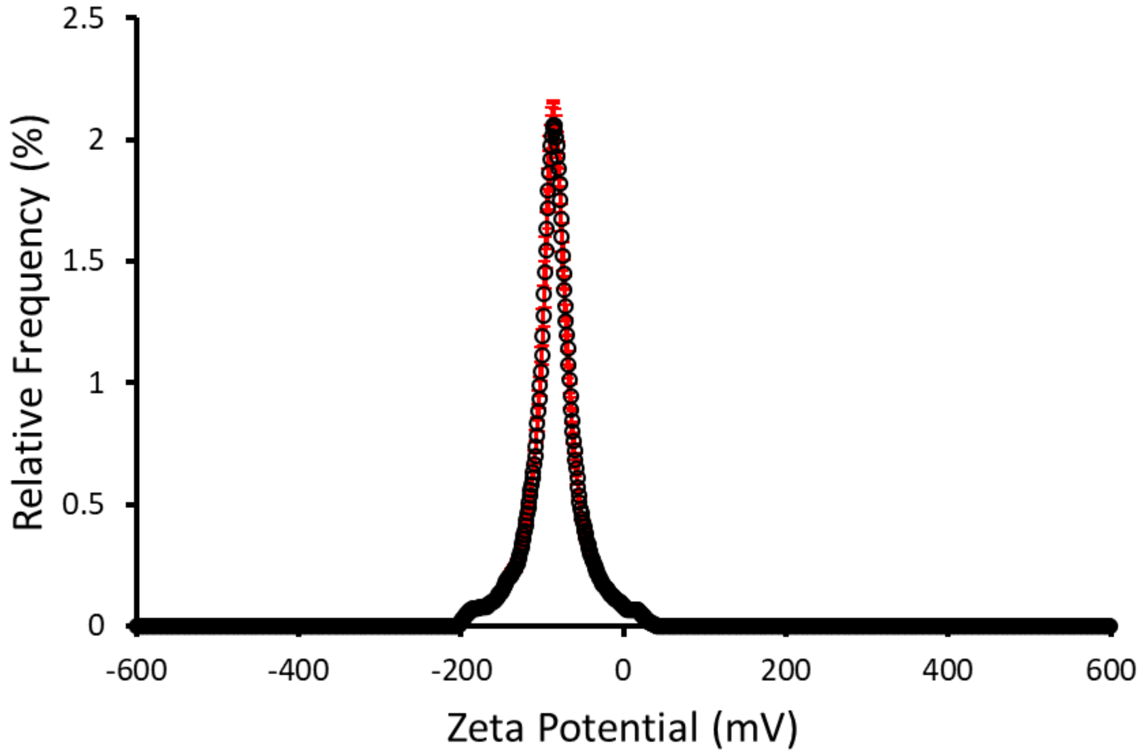

Figure S92 - The average zeta potential distribution calculated using 10 runs for **10** (1.5 mM) in an H_2O/5 % EtOH solution at 298 K. Average measurement value -94 mV.

Table S5 - Summary of the average zeta potential (mV) distribution calculated using 10 runs at 298 K. Error = standard deviation (SDV) of the mean.

**SSA**	**H_2O/5 % EtOH**		
	**Concentration (mM)**	**ZP (mV)**	**Error (±mV)**
**1**	5.56	-48.3	1.02
**2**	5.56	-63.7	1.10
**3**	5.56	-65.9	1.10
**4**	5.56	-47.0	1.55
**5**	5.56	-70.8	1.56
**6**	2.75	-71.0	1.30
**7**	5.56	-81.9	0.86
**8**	5.56	-71.7	1.10
**9**	5.56	-92.4	1.84
**10**	1.50	-94.0	2.10

Section 7.2: Surface tension and determination of critical aggregation concentration (CAC)

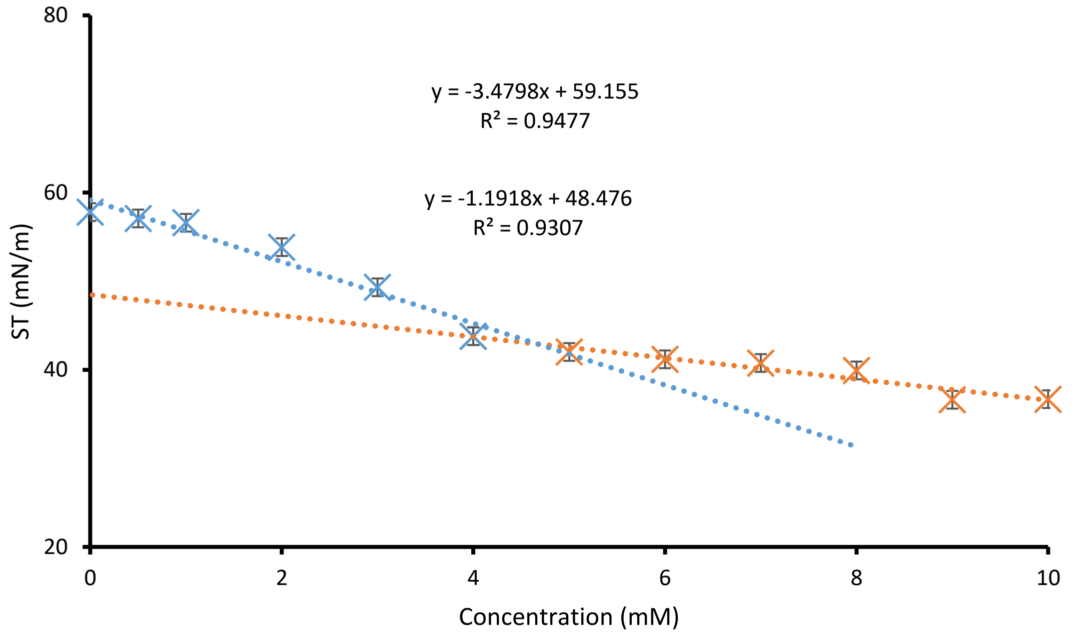

Figure S93 - Calculation of CAC (4.50 mM) for **1** in an H_2O/5 % EtOH mixture using surface tension measurements.

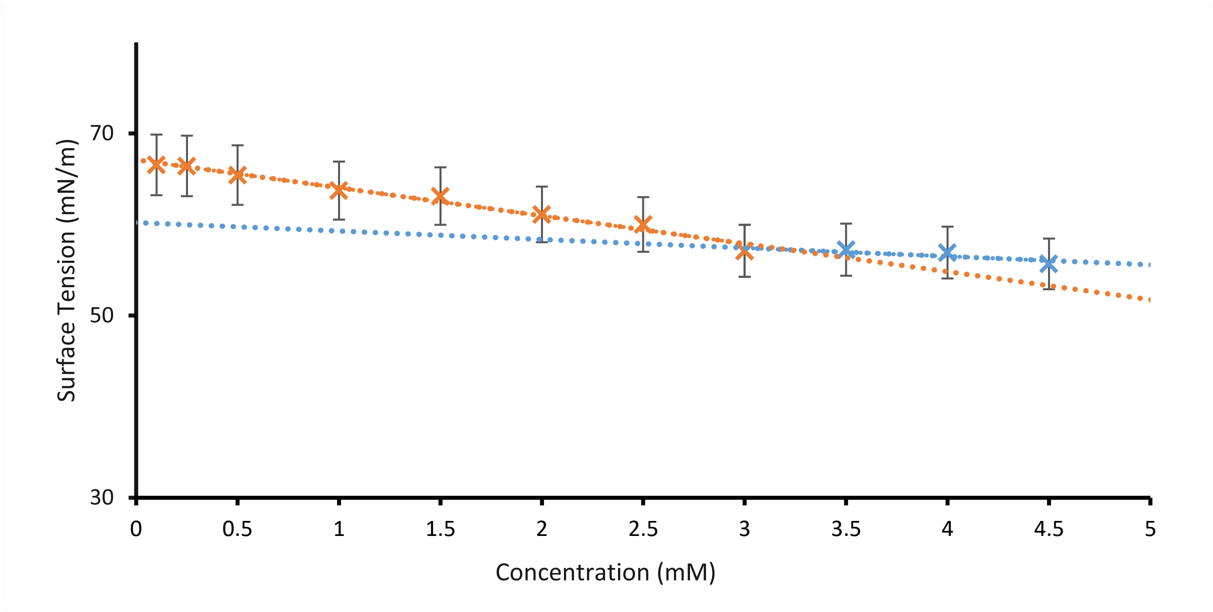

Figure S94 – A graph showing the CAC of **2** in an H_2O/5 % EtOH mixture using surface tension measurements. Due to solubility limits, this study could not be conducted above 5.56 mM.

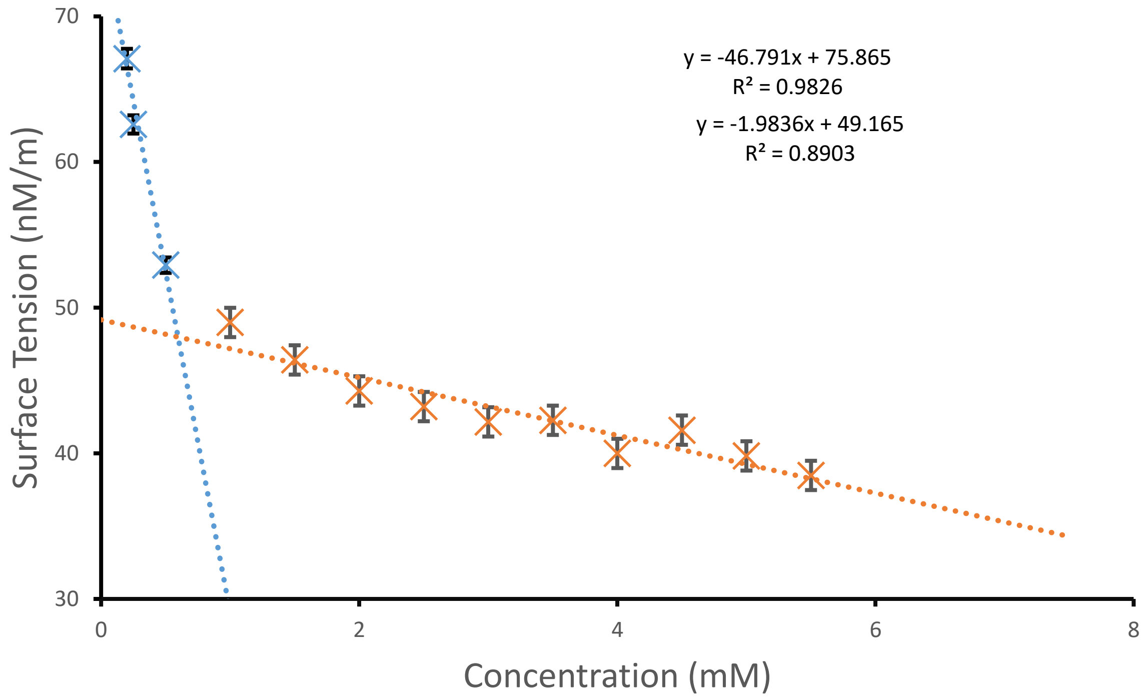

Figure S95 - Calculation of CAC (0.52 mM) for **3** in an H_2O/5 % EtOH mixture using surface tension measurements.

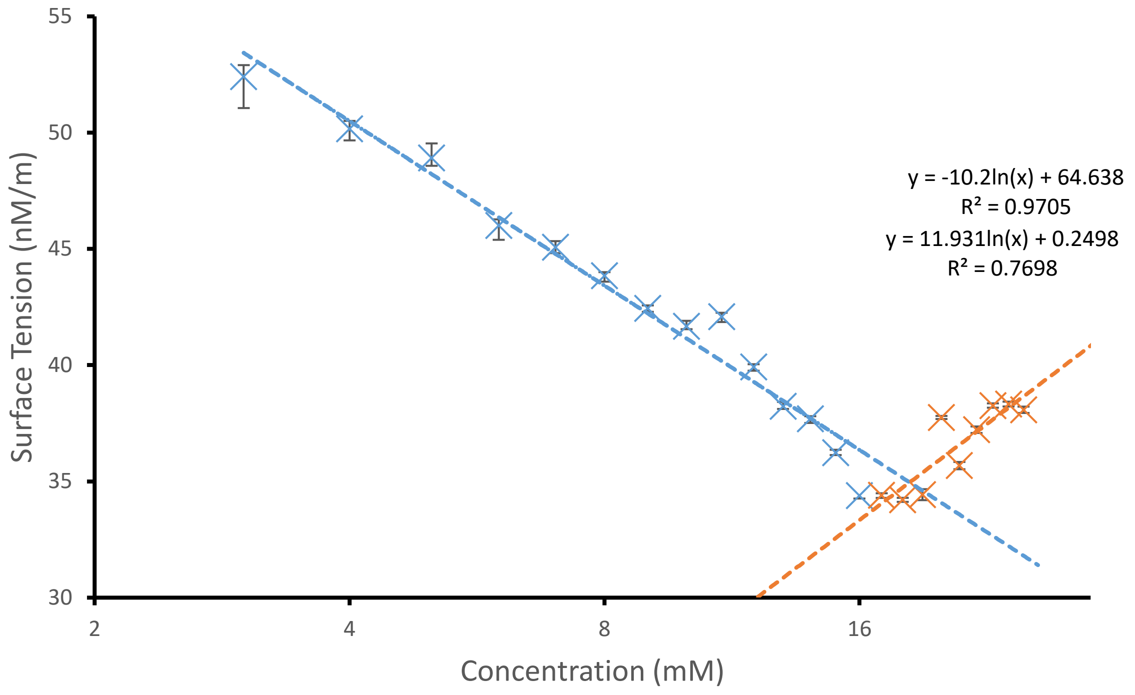

Figure S96 - Calculation of CAC (18.5 mM) for **4** in an H_2O/5 % EtOH mixture using surface tension measurements.

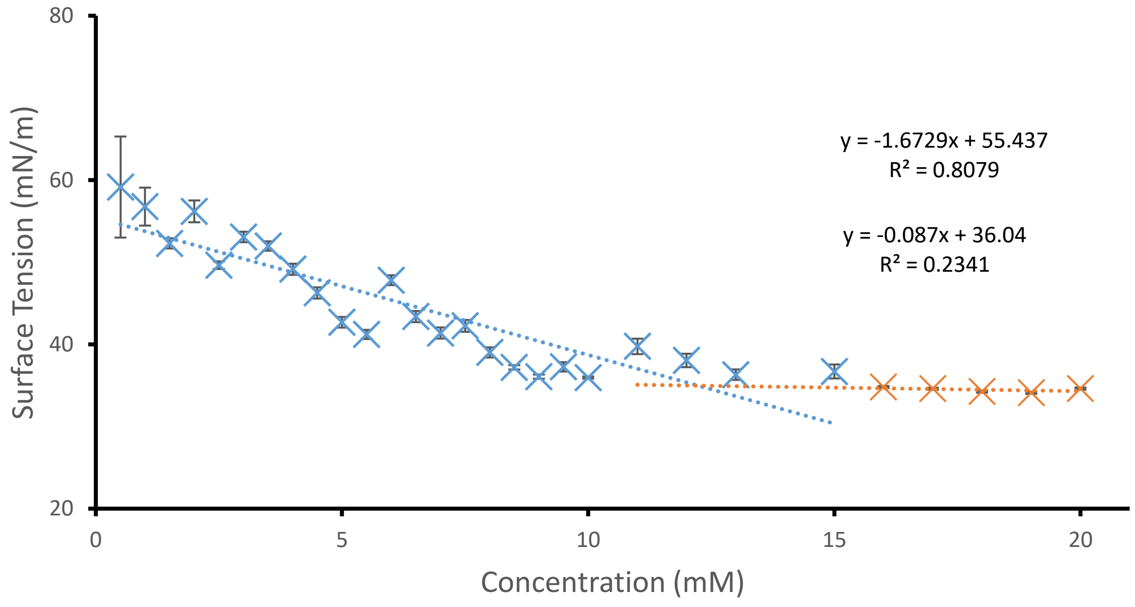

Figure S97 - Calculation of CAC (12.5 mM) for **5** in an H_2O/5 % EtOH mixture using surface tension measurements.

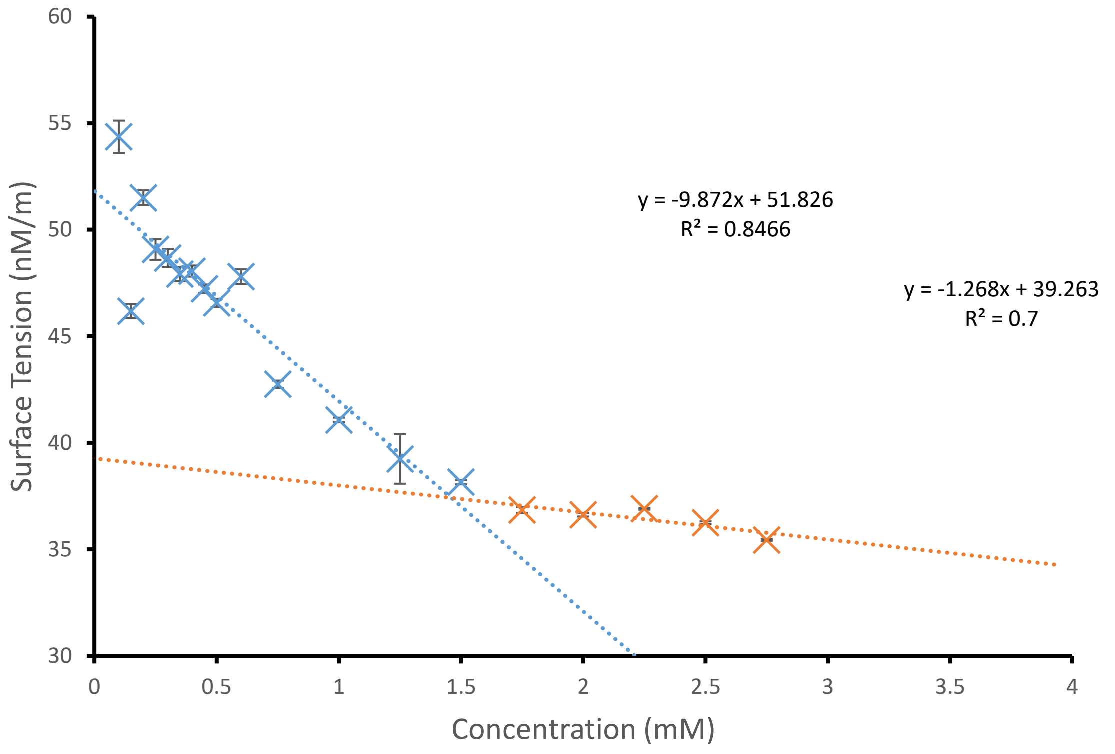

Figure S98 - Calculation of CAC (1.45 mM) for **6** in an H_2O/5 % EtOH mixture using surface tension measurements.

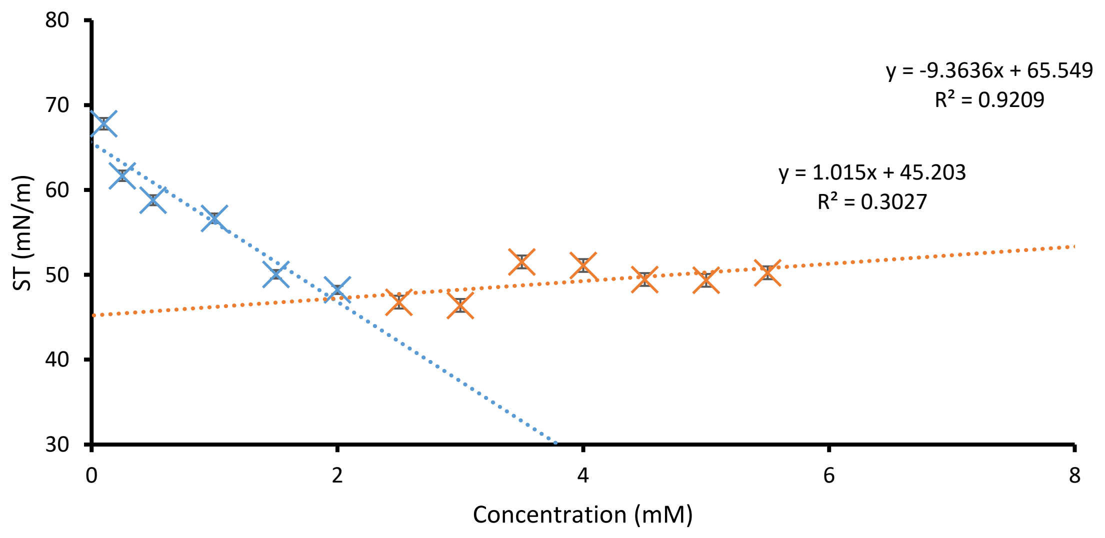

Figure S99 - Calculation of CAC (2.00 mM) for **7** in an H_2O/5 % EtOH mixture using surface tension measurements.

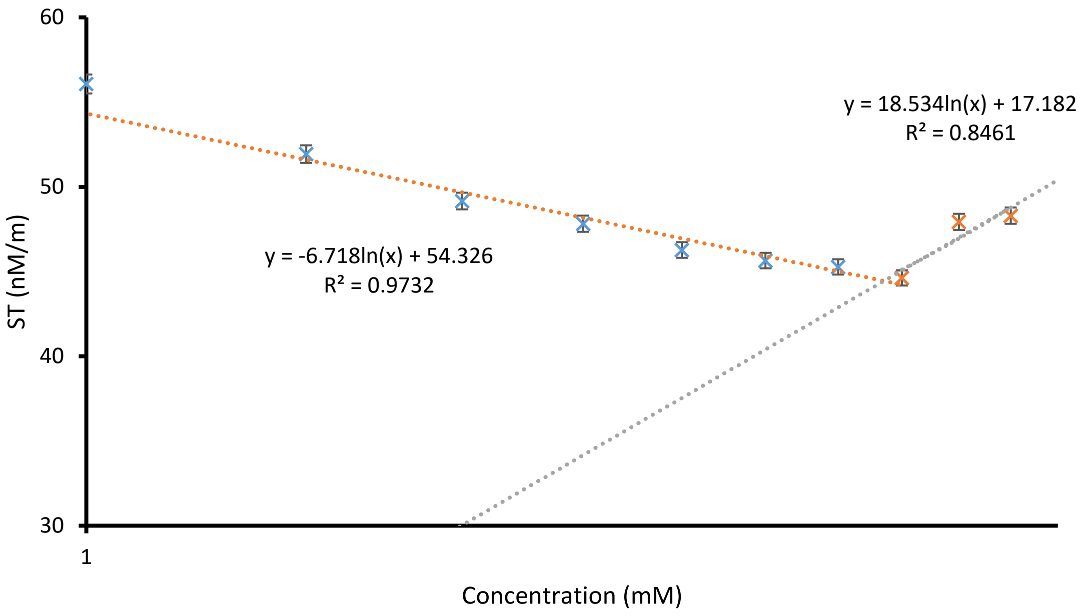

Figure S100 - Calculation of CAC (4.35 mM) for **8** in an H_2O/5 % EtOH mixture using surface tension measurements.

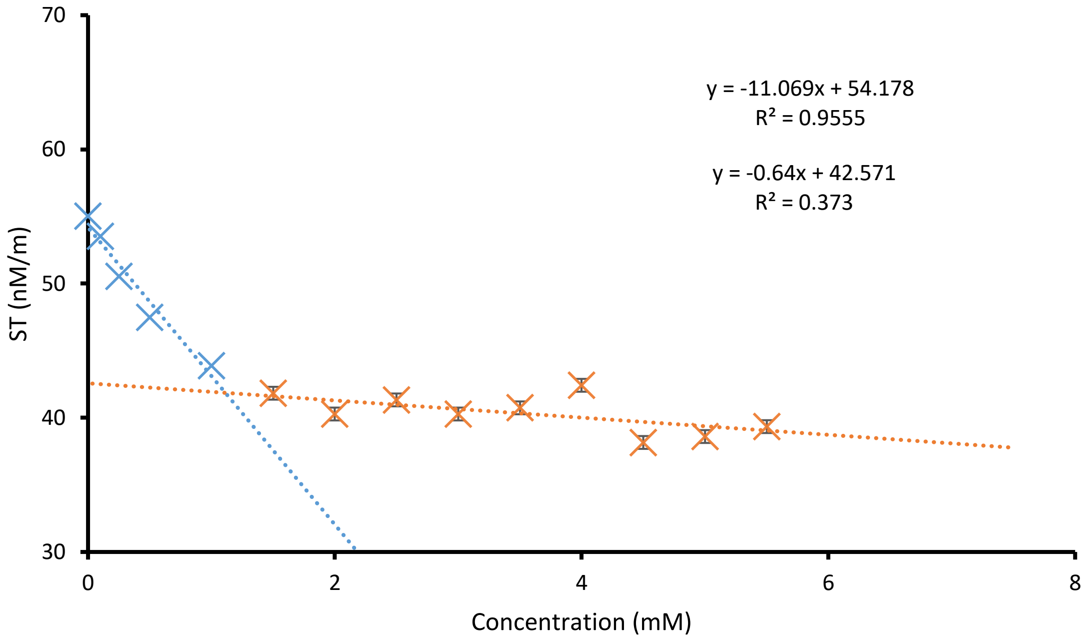

Figure S101 - Calculation of CAC (1.10 mM) for **9** in an H_2O/5 % EtOH mixture using surface tension measurements.

Table S6 - Summary table of critical aggregation studies for **1-10**. CAC was derived at approximately 298 K from surface tension measurements.

**SSA**	**H_2O/5 % ETOH**	
	**CAC (mM)**	**ST at CAC (nN/m)**
**1**	4.50	43
**2**	*a*	*a*
**3**	0.52	48
**4**	18.5	35
**5**	12.5	35
**6**	1.45	38
**7**	2.00	47
**8**	4.35	44
**9**	1.10	42
**10**	*a*	a

a - CAC above the limit of solubility or does not have surfactant properties.

Section S8: Low level in-silico modelling

Section S8.1: Electrostatic potential maps

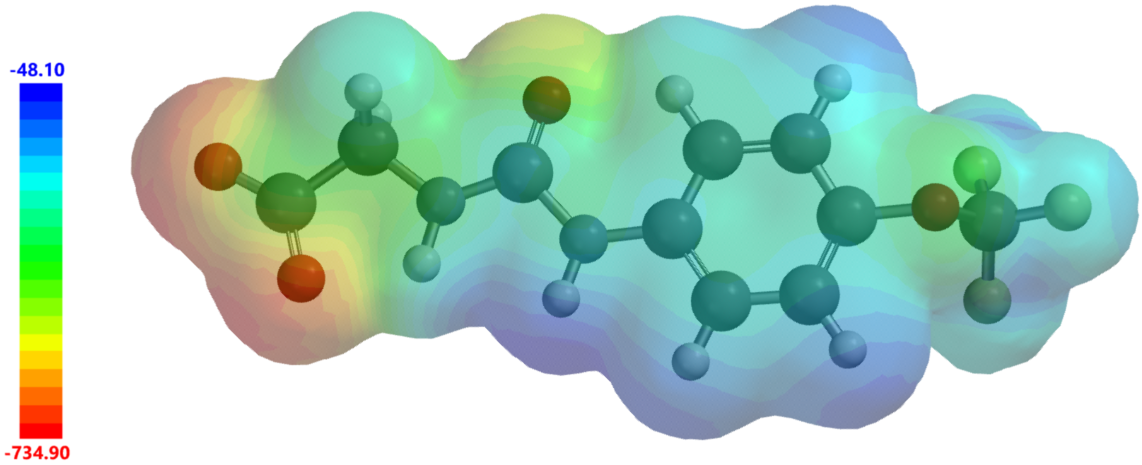

Figure S102 - Electrostatic potential map calculated for the anionic component of **1**. E_{max} and E_{min} values depicted in the figure legends are given in KJ/mol.

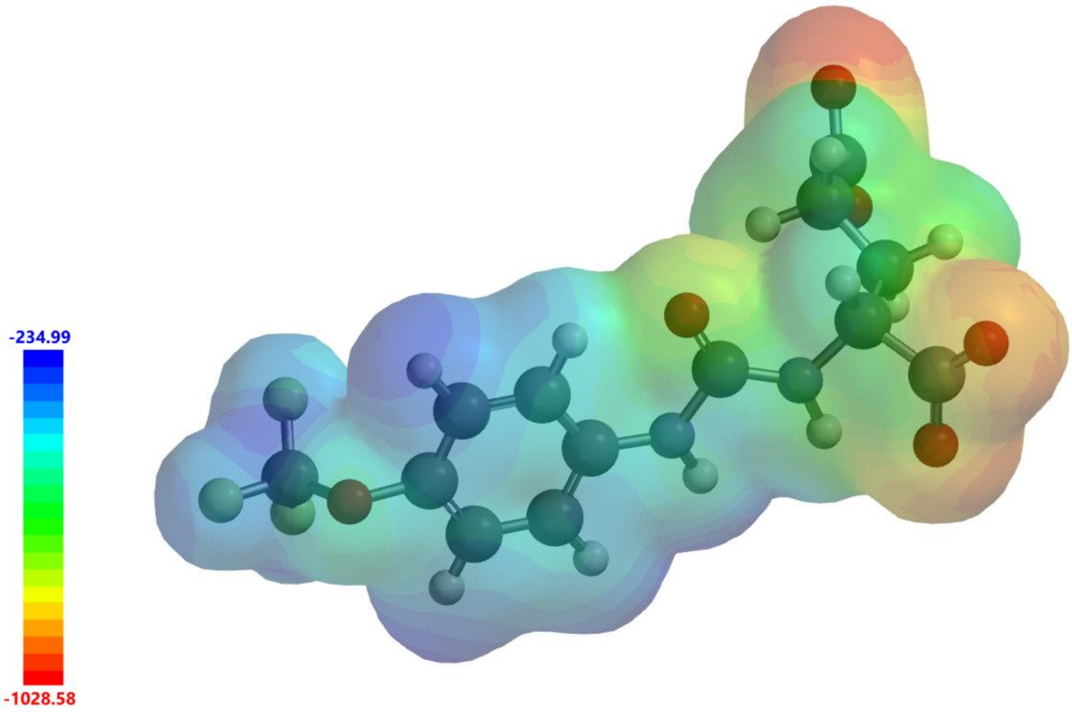

Figure S103 - Electrostatic potential map calculated for the anionic component of **2**. E_{max} and E_{min} values depicted in the figure legends are given in KJ/mol.

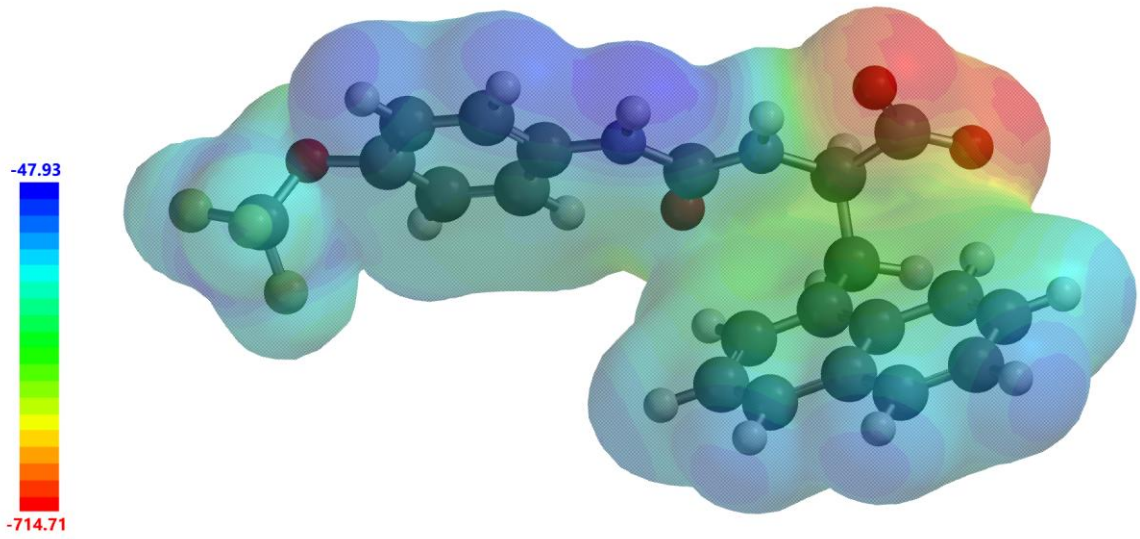

Figure S104 - Electrostatic potential map calculated for the anionic component of **3**. E_{max} and E_{min} values depicted in the figure legends are given in KJ/mol.

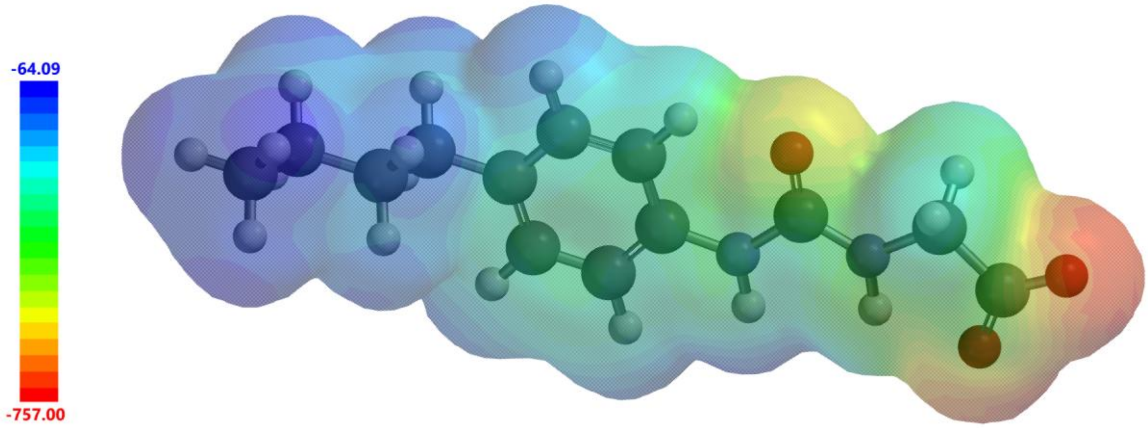

Figure S105 - Electrostatic potential map calculated for the anionic component of **4**. E_{max} and E_{min} values depicted in the figure legends are given in KJ/mol.

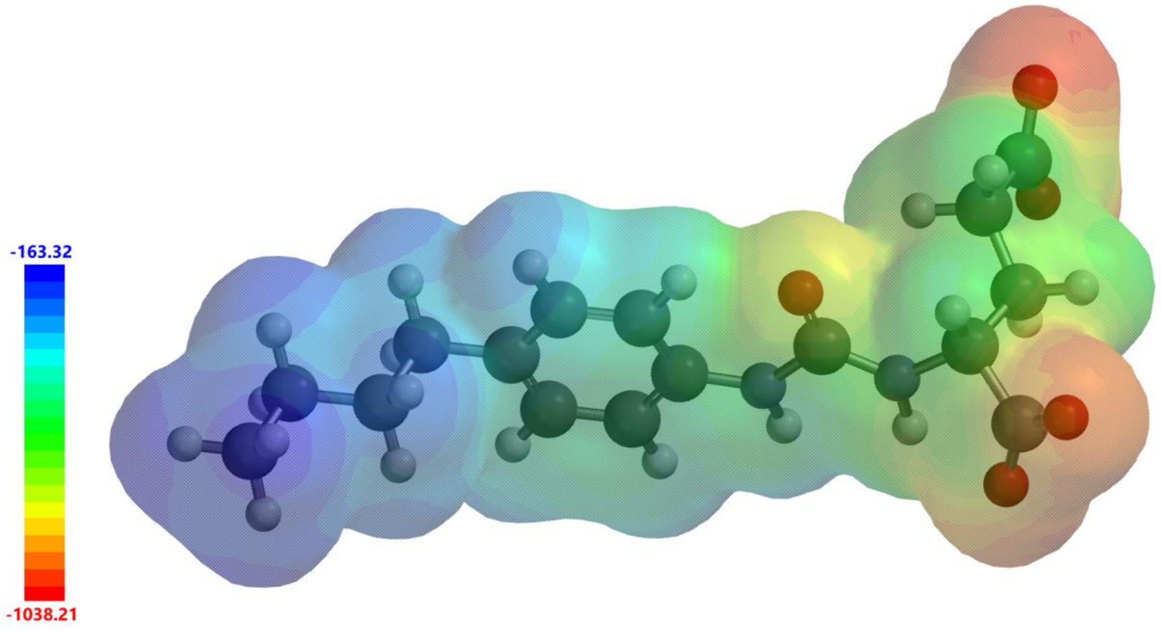

Figure S106 - Electrostatic potential map calculated for the anionic component of **5**. E_{max} and E_{min} values depicted in the figure legends are given in KJ/mol.

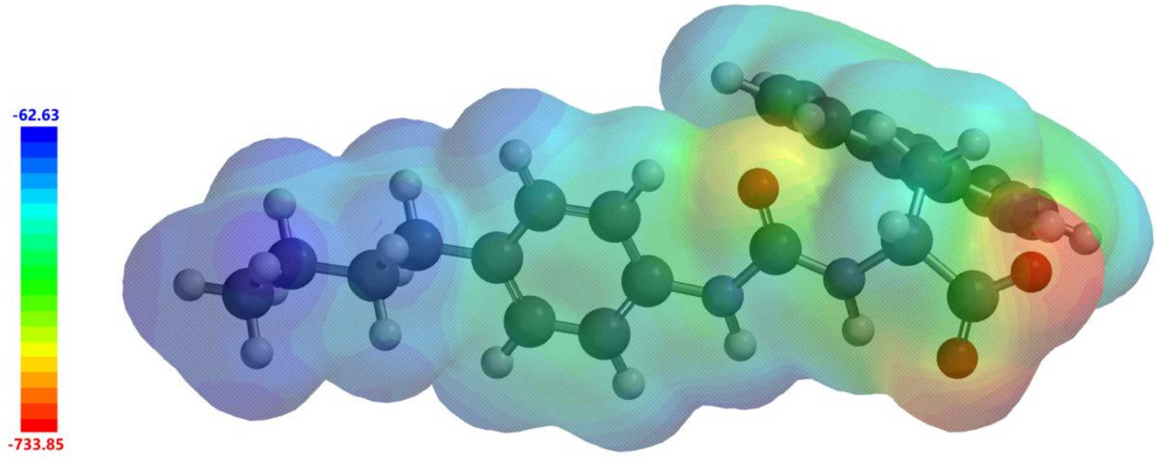

Figure S107 - Electrostatic potential map calculated for the anionic component of **6**. E_{max} and E_{min} values depicted in the figure legends are given in KJ/mol.

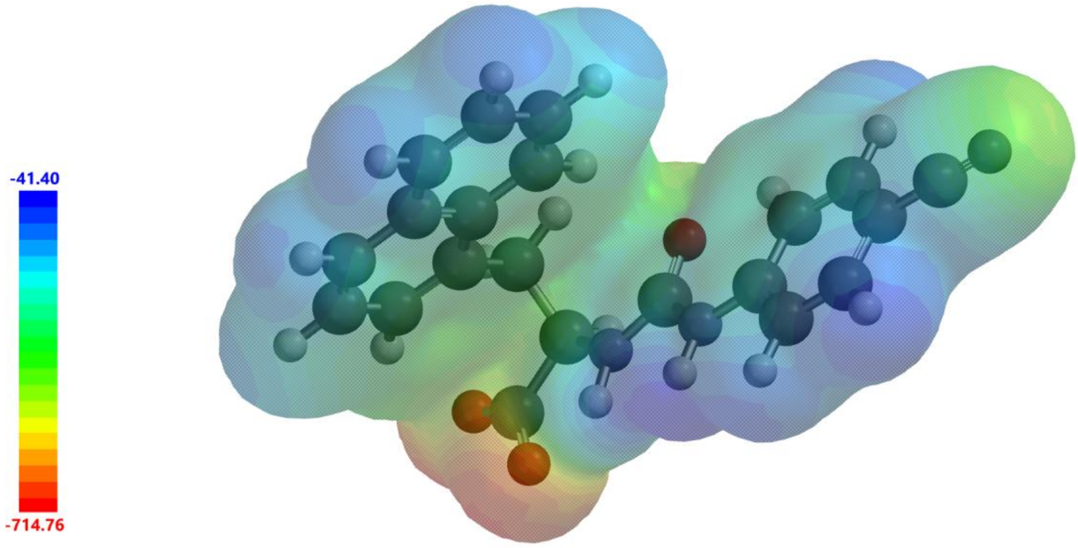

Figure S108 - Electrostatic potential map calculated for the anionic component of **7**. E_{max} and E_{min} values depicted in the figure legends are given in KJ/mol.

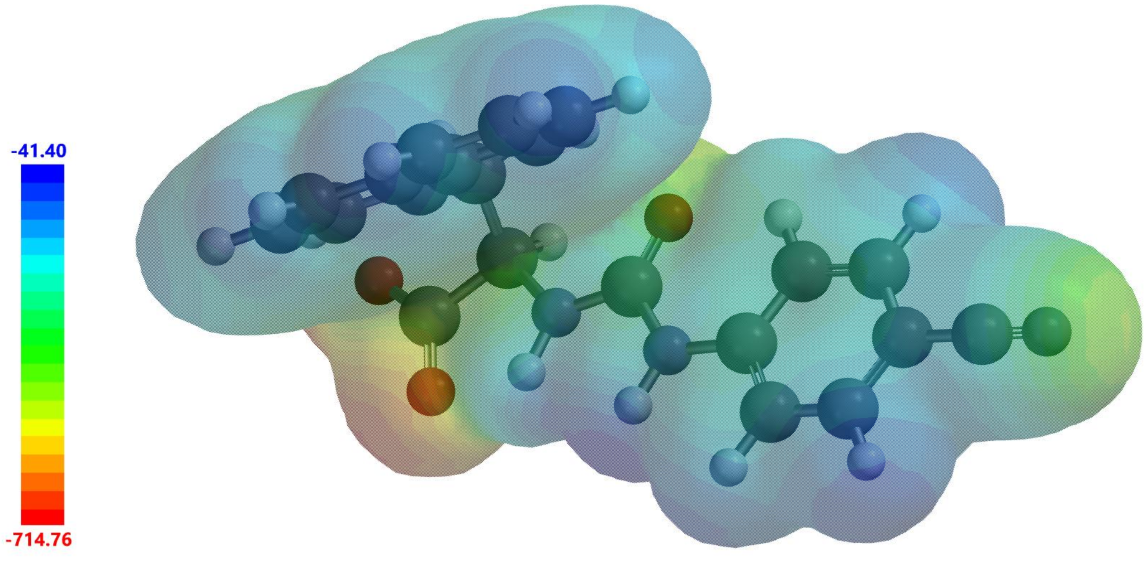

Figure S109 - Electrostatic potential map calculated for the anionic component of **8**. E_{max} and E_{min} values depicted in the figure legends are given in KJ/mol.

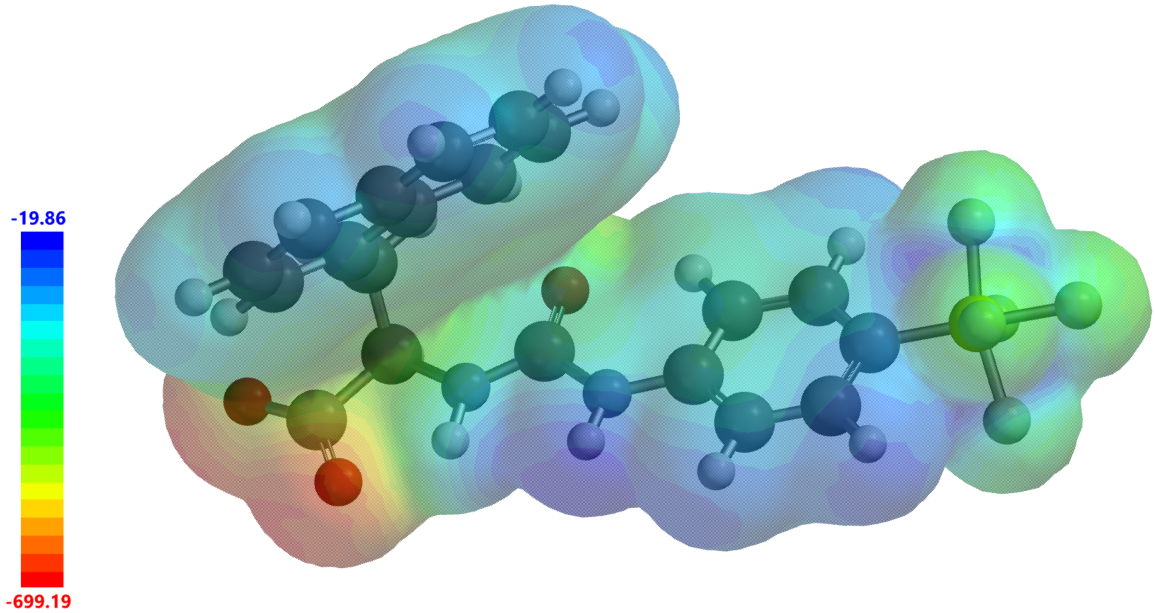

Figure S110 - Electrostatic potential map calculated for the anionic component of **9**. E_{max} and E_{min} values depicted in the figure legends are given in KJ/mol.

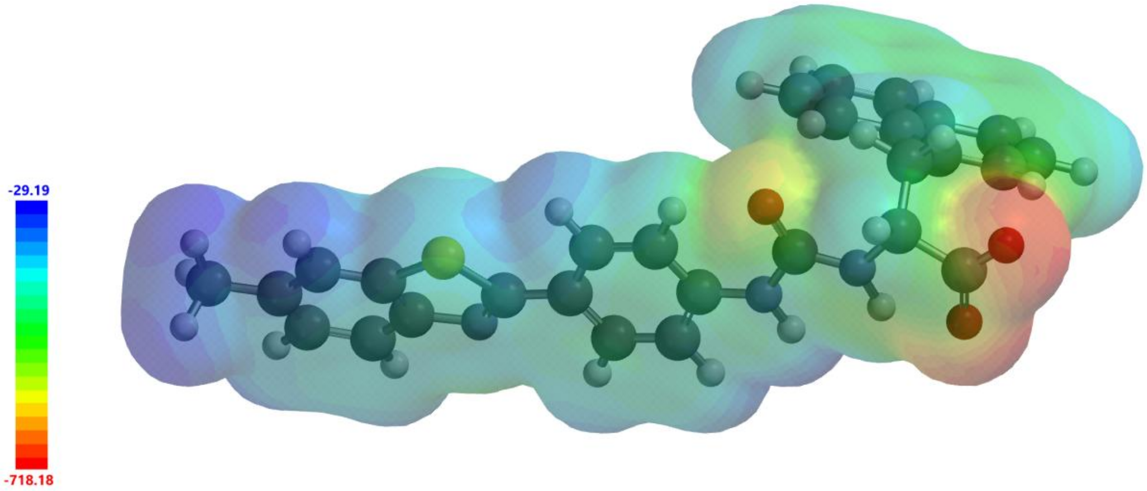

Figure S111 - Electrostatic potential map calculated for the anionic component of **10**. E_{max} and E_{min} values depicted in the figure legends are given in KJ/mol.

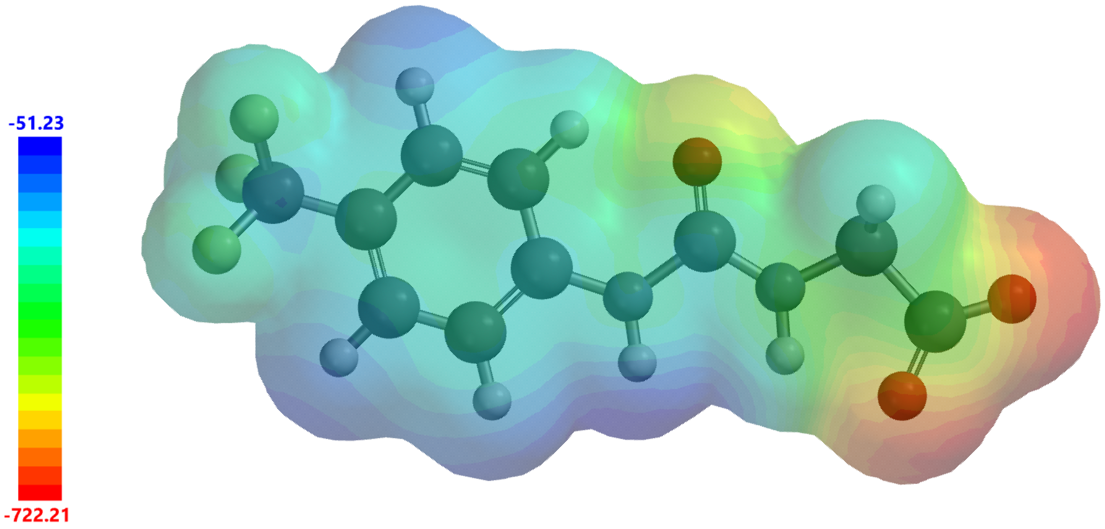

Figure S112 - Electrostatic potential map calculated for the anionic component of **the control compound** (see main manuscript). E_{max} and E_{min} values depicted in the figure legends are given in KJ/mol.

Section S8.2: Modelling *in vitro* ADME properties

To estimate the *in vitro* ADME properties for the anionic components of **1-10** and the control compound (see main manuscript), the anionic component of these structures was converted from a ChemDraw image to the following set of smiles:

1 = [O-]C(CNC(NC1=CC=C(OC(F)(F)F)C=C1)=O)=O

2 = [O-]C(CC[C@H](C([O-])=O)NC(NC1=CC=C(OC(F)(F)F)C=C1)=O)=O

3 = O=C([O-])[C@H](NC(NC1=CC=C(OC(F)(F)F)C=C1)=O)CC2=C3C(C=CC=C3)=CC=C2

4 = CCCCC1=CC=C(NC(NCC([O-])=O)=O)C=C1

5 = [O-]C(CC[C@H](C([O-])=O)NC(NC1=CC=C(CCCC)C=C1)=O)=O

6 = CCCCC1=CC=C(NC(N[C@H](CC2=C3C(C=CC=C3)=CC=C2)C([O-])=O)=O)C=C1

7 = [O-]C([C@@H](CC1=CC=CC2=C1C=CC=C2)NC(NC3=CC=C(C#N)C=C3)=O)=O

8 = [O-]C([C@@H](CC1=CC=CC2=C1C=CC=C2)NC(NC3=CC(C#N)=CC=C3)=O)=O

9 = [O-]C([C@@H](CC1=CC=CC2=C1C=CC=C2)NC(NC3=CC=C(S(F)(F)(F)(F)F)C=C3)=O)=O

10 = O=C(N[C@H](CC1=CC=CC2=C1C=CC=C2)C([O])=O)NC3=CC=C(C4=NC5=C(C=C(C)C=C5)S4)C=C3

Control = [O-]C(CNC(NC1=CC=C(C(F)(F)F)C=C1)=O)=O

These smiles were then placed into Swiss ADME online portal[3] and the *in vitro* ADME properties for this group of compounds generated. From these data we report the consensus (c)LogP value.[4-6]

Section S8.3: Modelling data references

1. C. A. Hunter, *Angew. Chemie Int. Ed.*, 2004, **43**, 5310–5324.
2. J. J. P. Stewart, *J. Mol. Model.*, 2007, **13**, 1173–1213.
3. https://swissadme.ch/ (accessed 19/11/2025)
4. A. Daina, O. Michielin, V. Zoete, *Sci. Rep.*, 2017, **7**, 42717.
5. A. Daina, O. Michielin, V. Zoete, *J. Chem. Inf. Model.*, 2014, **54**, 3284-3301.
6. A. Daina, V. Zoete, *ChemMedChem*, 2016, **11**, 1117-1121.

Section S9: UV-Vis and fluorescence dilution study data

Section S9.1 UV-Vis dilution data

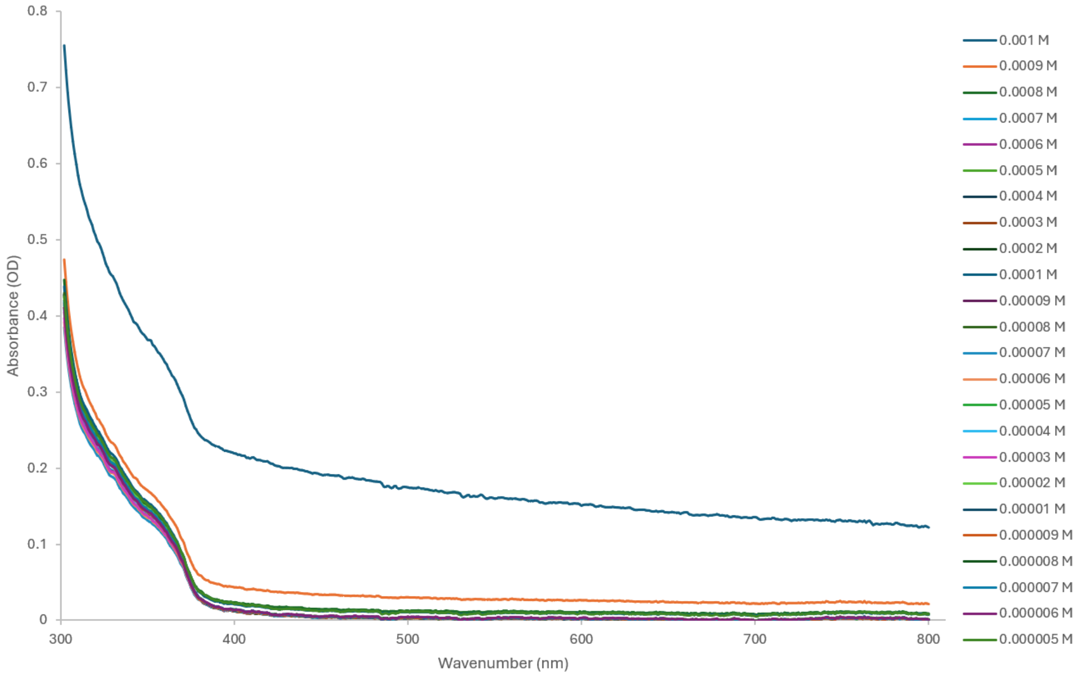

Figure S113 - UV-Vis spectra of control compound at 1.0 mM for 24 dilutions in an H_2O/5 % EtOH mixture at 298 K. All spectra were collected n = 3 and subsequently averaged. The averaged spectra were then normalized by subtracting the absorbance of the solvent (n = 3).

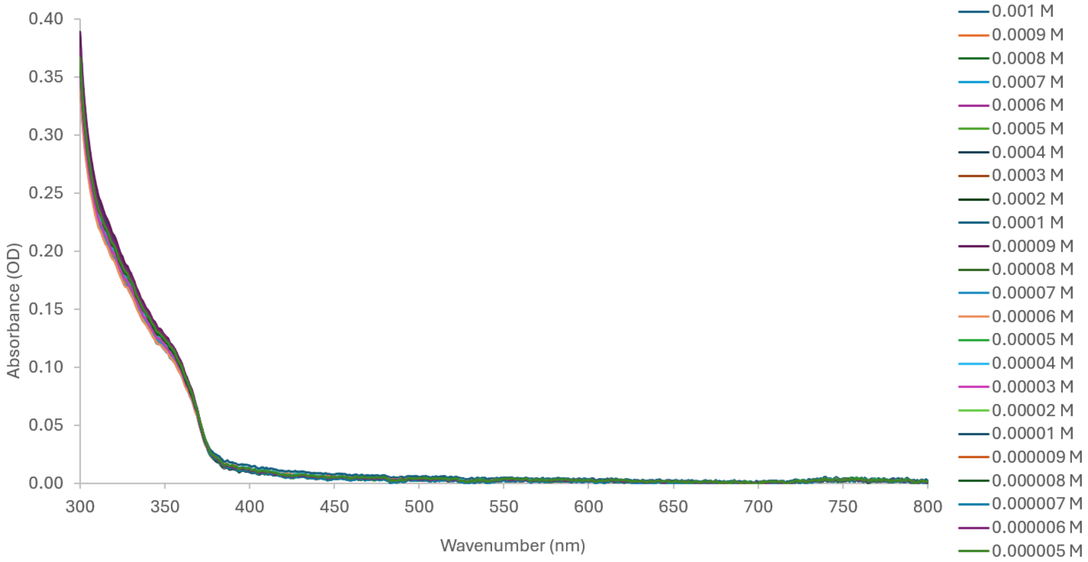

Figure S114 - UV-Vis spectra of **1** at 1.0 mM for 24 dilutions in an H_2O/5 % EtOH mixture at 298 K. All spectra were collected n = 3 and subsequently averaged. The averaged spectra were then normalized by subtracting the absorbance of the solvent (n = 3).

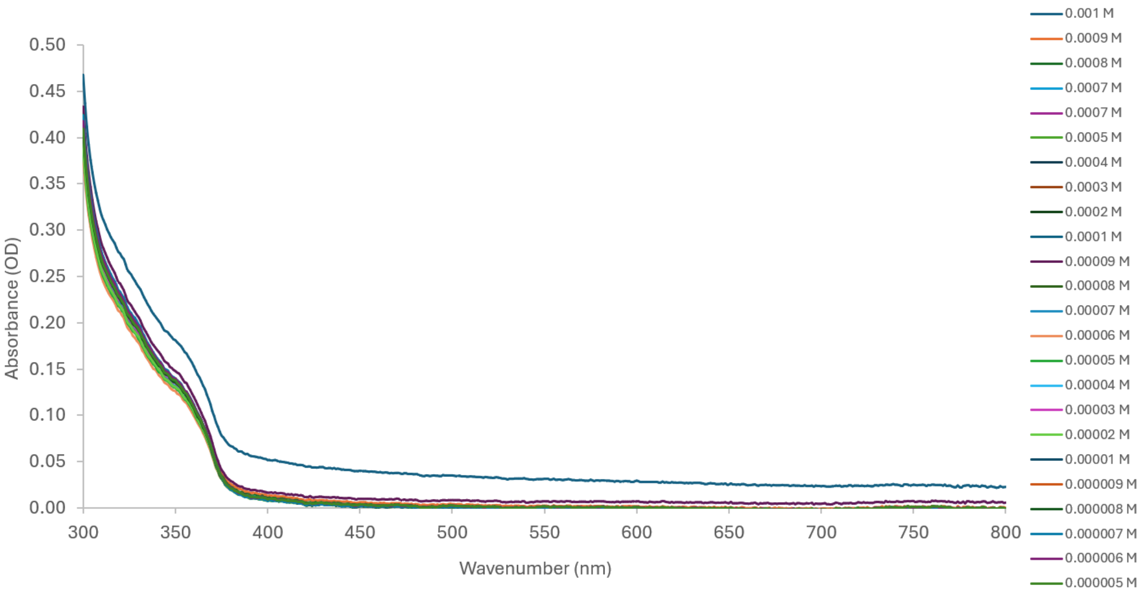

Figure S115 - UV-Vis spectra of **2** at 1.0 mM for 24 dilutions in an H_2O/5 % EtOH mixture at 298 K. All spectra were collected n = 3 and subsequently averaged. The averaged spectra were then normalized by subtracting the absorbance of the solvent (n = 3).

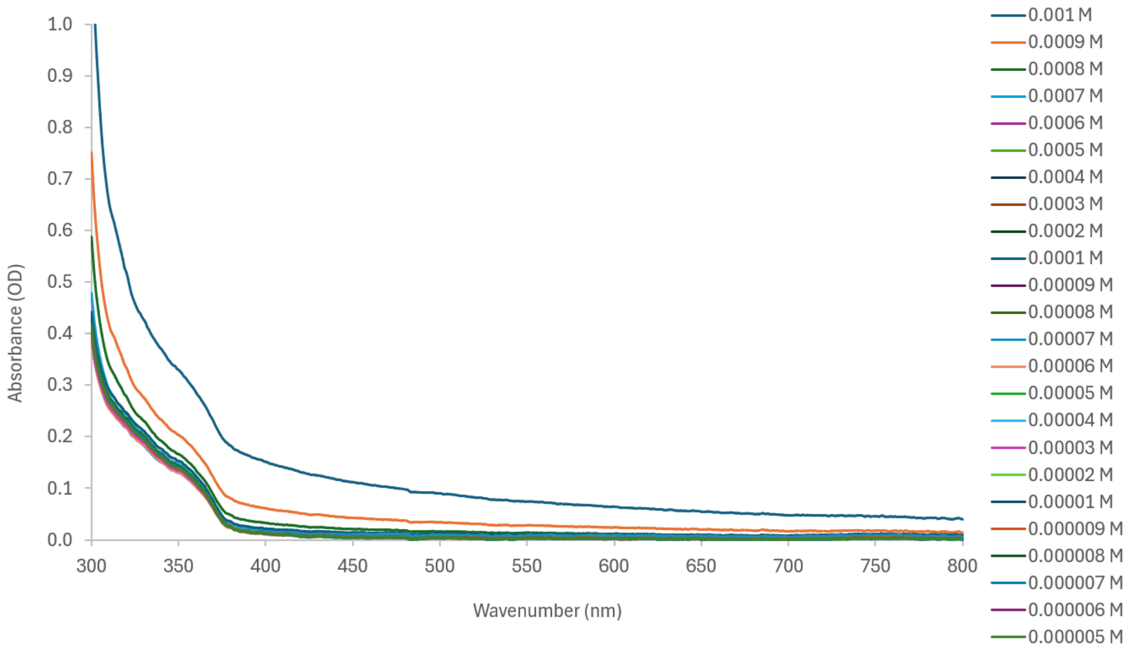

Figure S116 - UV-Vis spectra of **3** at 1.0 mM for 24 dilutions in an H_2O/5 % EtOH mixture at 298 K. All spectra were collected n = 3 and subsequently averaged. The averaged spectra were then normalized by subtracting the absorbance of the solvent (n = 3).

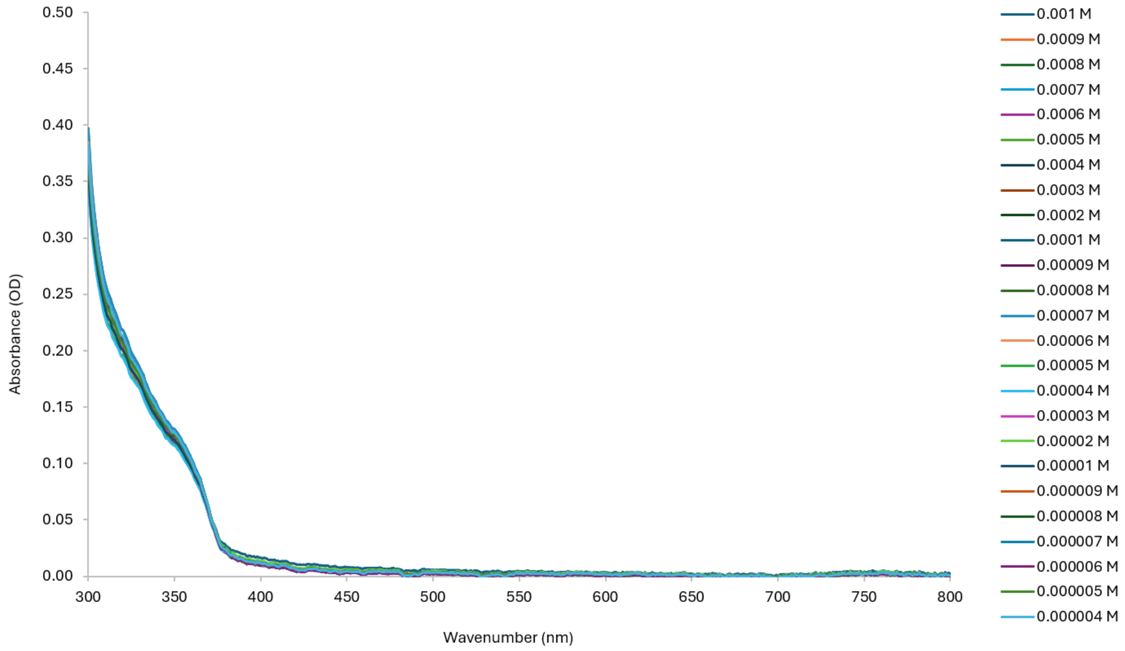

Figure S117 - UV-Vis spectra of **4** at 1.0 mM for 24 dilutions in an H_2O/5 % EtOH mixture at 298 K. All spectra were collected n = 3 and subsequently averaged. The averaged spectra were then normalized by subtracting the absorbance of the solvent (n = 3).

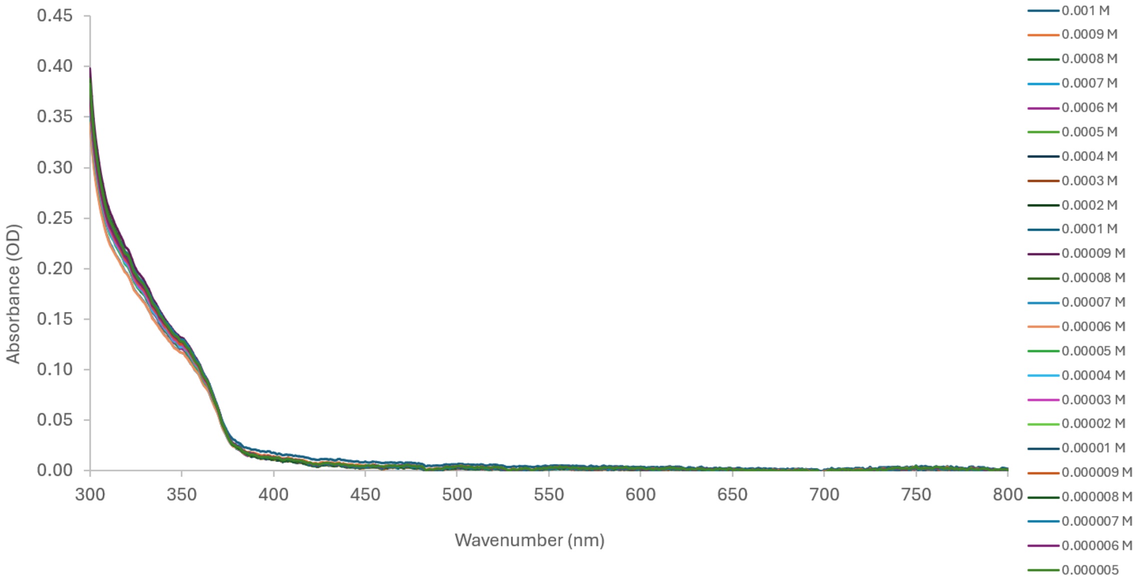

Figure S118 - UV-Vis spectra of **5** at 1.0 mM for 24 dilutions in an H_2O/5 % EtOH mixture at 298 K. All spectra were collected n = 3 and subsequently averaged. The averaged spectra were then normalized by subtracting the absorbance of the solvent (n = 3).

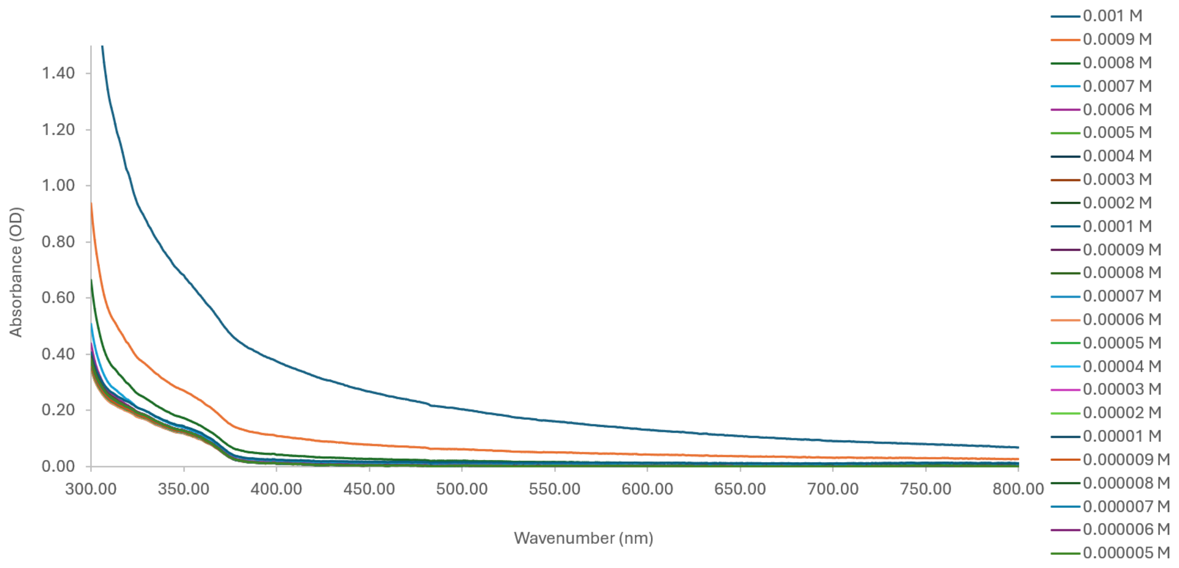

Figure S119 - UV-Vis spectra of **6** at 1.0 mM for 24 dilutions in an H_2O/5 % EtOH mixture at 298 K. All spectra were collected n = 3 and subsequently averaged. The averaged spectra were then normalized by subtracting the absorbance of the solvent (n = 3).

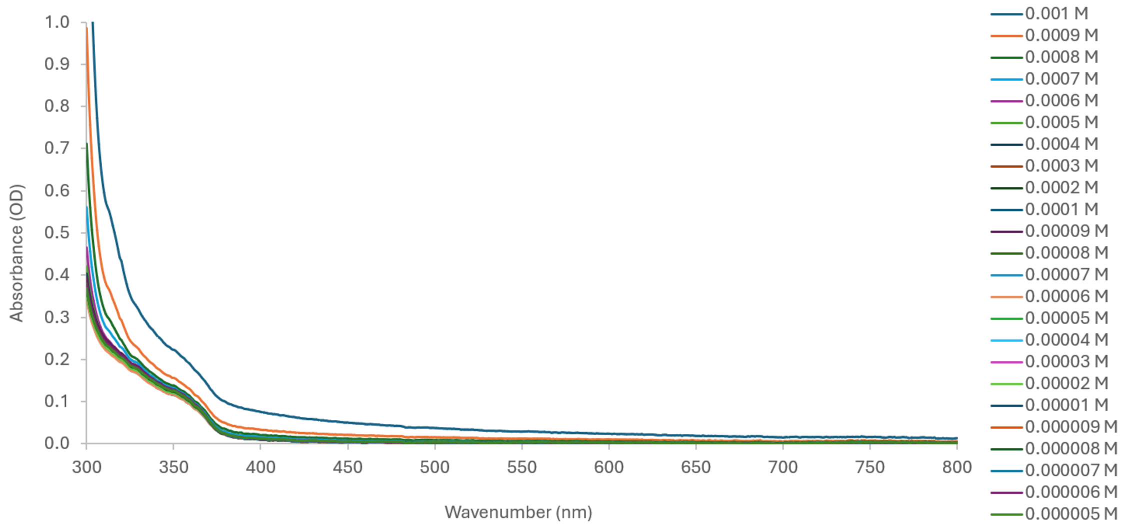

Figure S120 - UV-Vis spectra of **7** at 1.0 mM for 24 dilutions in an H_2O/5 % EtOH mixture at 298 K. All spectra were collected n = 3 and subsequently averaged. The averaged spectra were then normalized by subtracting the absorbance of the solvent (n = 3).

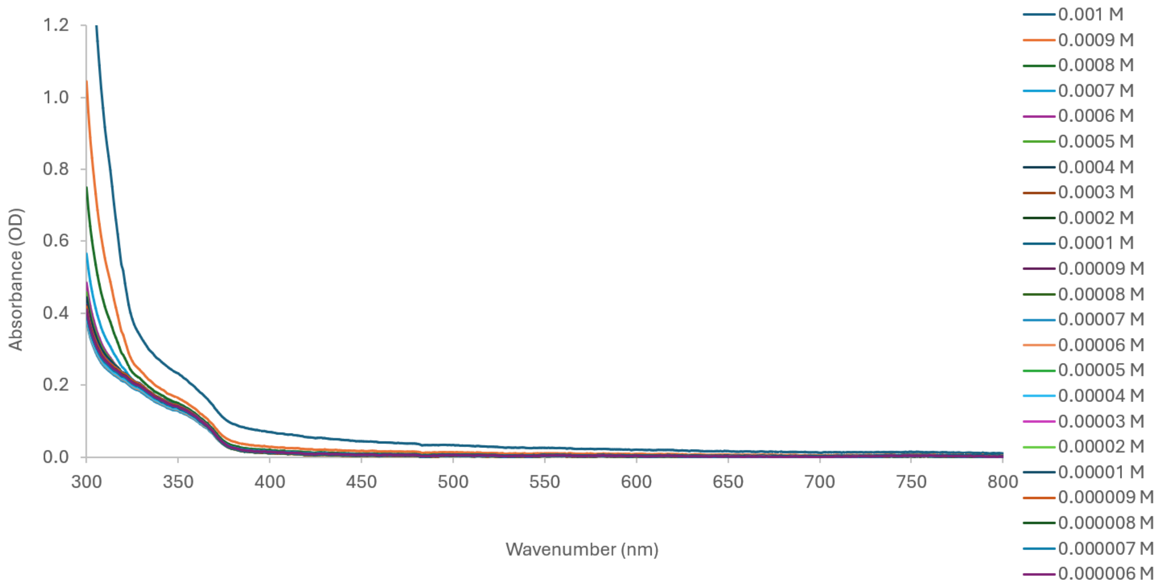

Figure S121 - UV-Vis spectra of **8** at 1.0 mM for 24 dilutions in an H_2O/5 % EtOH mixture at 298 K. All spectra were collected n = 3 and subsequently averaged. The averaged spectra were then normalized by subtracting the absorbance of the solvent (n = 3).

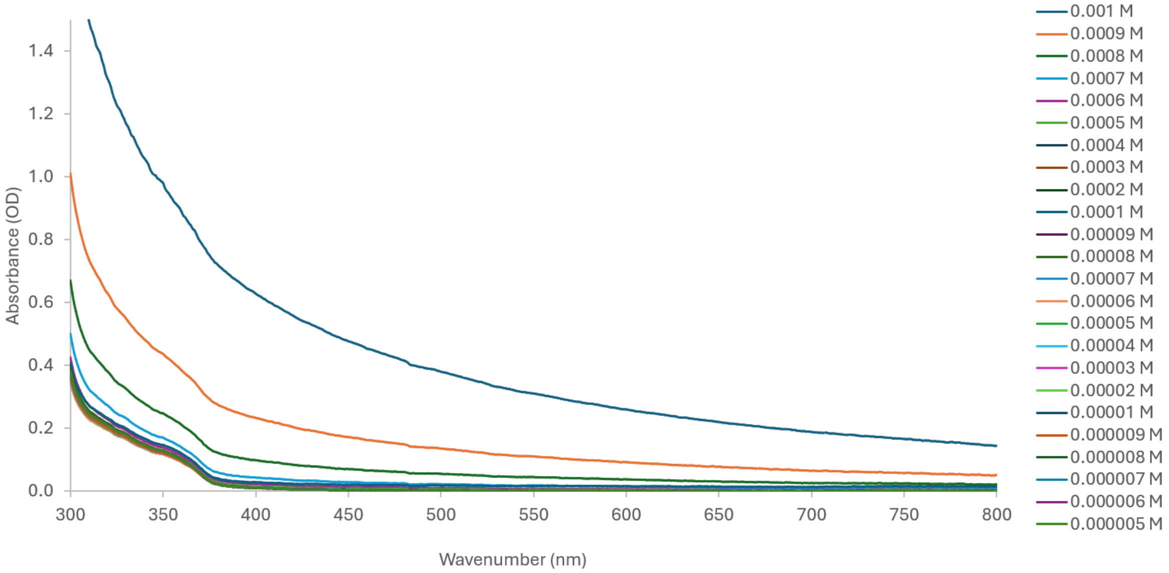

Figure S122 - UV-Vis spectra of **9** at 1.0 mM for 24 dilutions in an H_2O/5 % EtOH mixture at 298 K. All spectra were collected n = 3 and subsequently averaged. The averaged spectra were then normalized by subtracting the absorbance of the solvent (n = 3).

Section S9.2: Fluorescence data

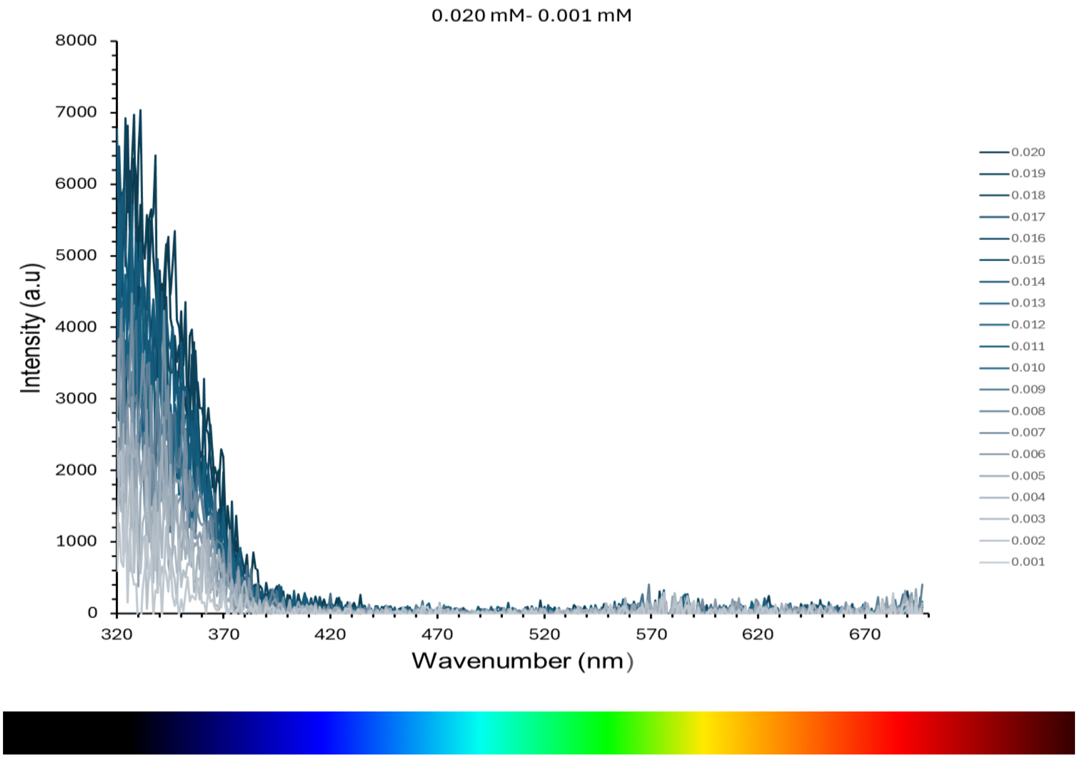

Figure S123 - Fluorescence excitation dilution study data of **10** at 0.02 - 0.001 mM (solutiom).

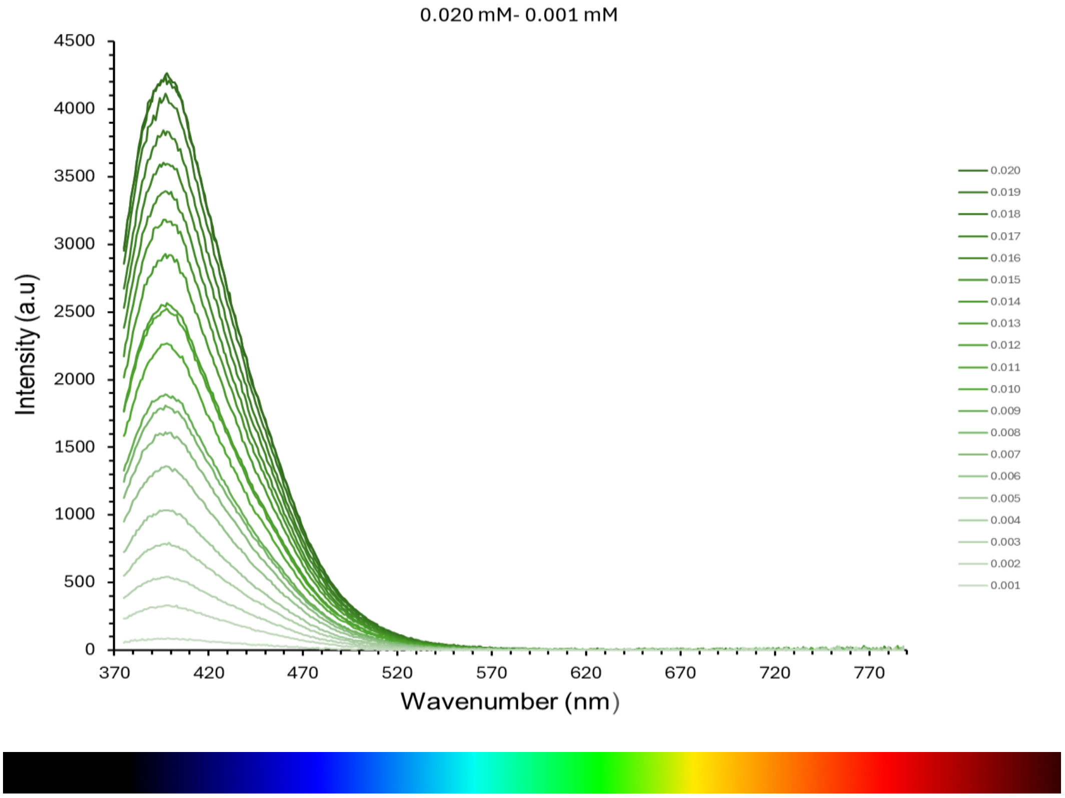

Figure S124 - Fluorescence emission dilution study data of **10** at 0.02 - 0.001 mM (add excitation wavelength and solvent).

Section S9.3: Bindfit data

Section S9.3.1: EK and CoEK binding isotherm models

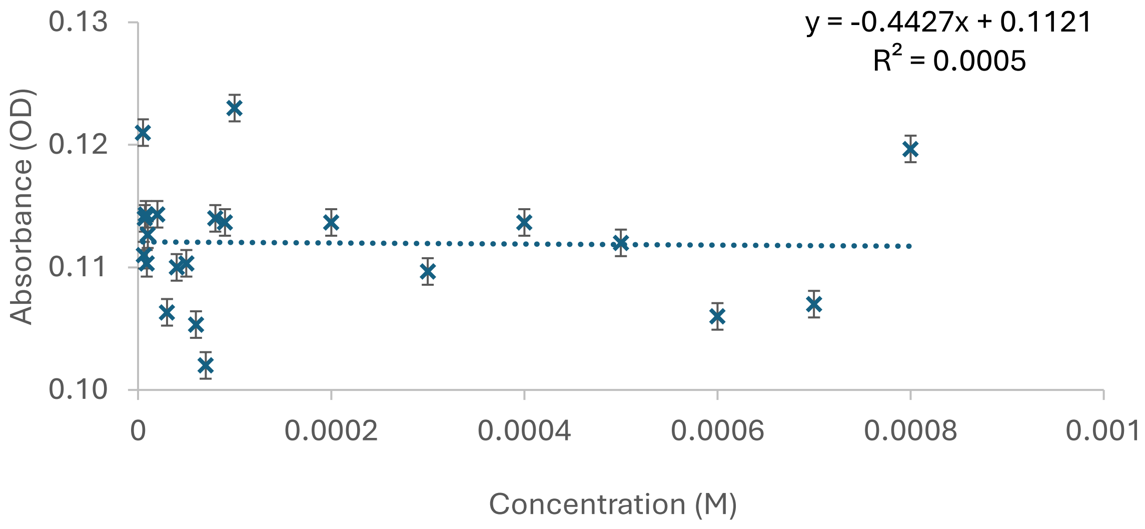

Figure S125 - UV-Vis dilution study data of control compound could not be fitted to either the EK or CoEK binding isotherm models. Spectral signals are hypothesised to be affected by compound aggregation typical to self-associating amphiphiles of this nature, at concentrations > 0.0008 M.

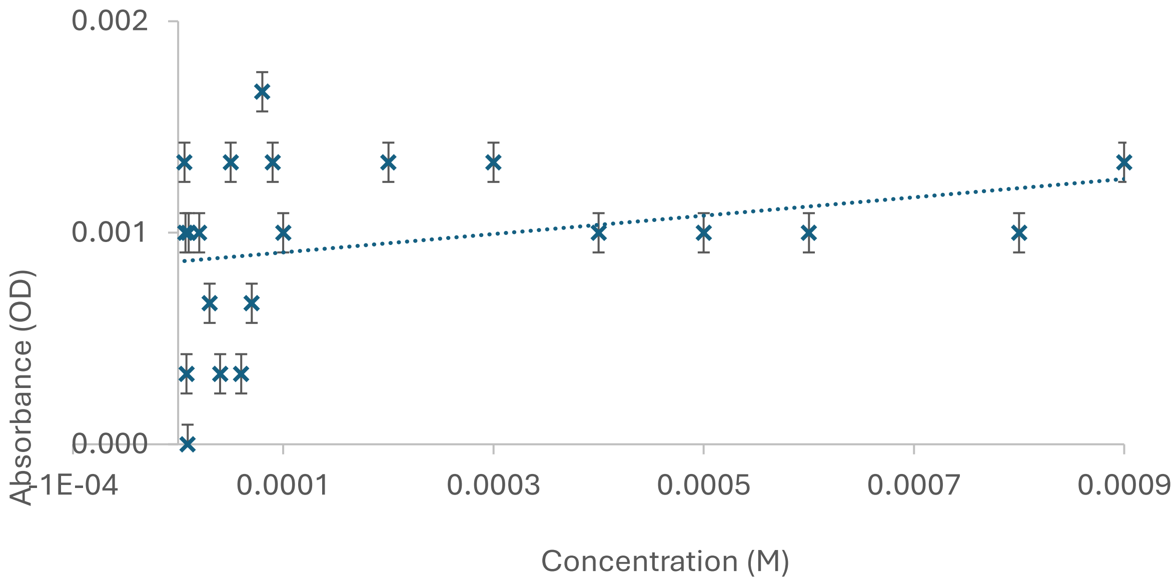

Figure S126 - UV-Vis dilution study data of **1** could not be fitted to either the EK or CoEK binding isotherm models.

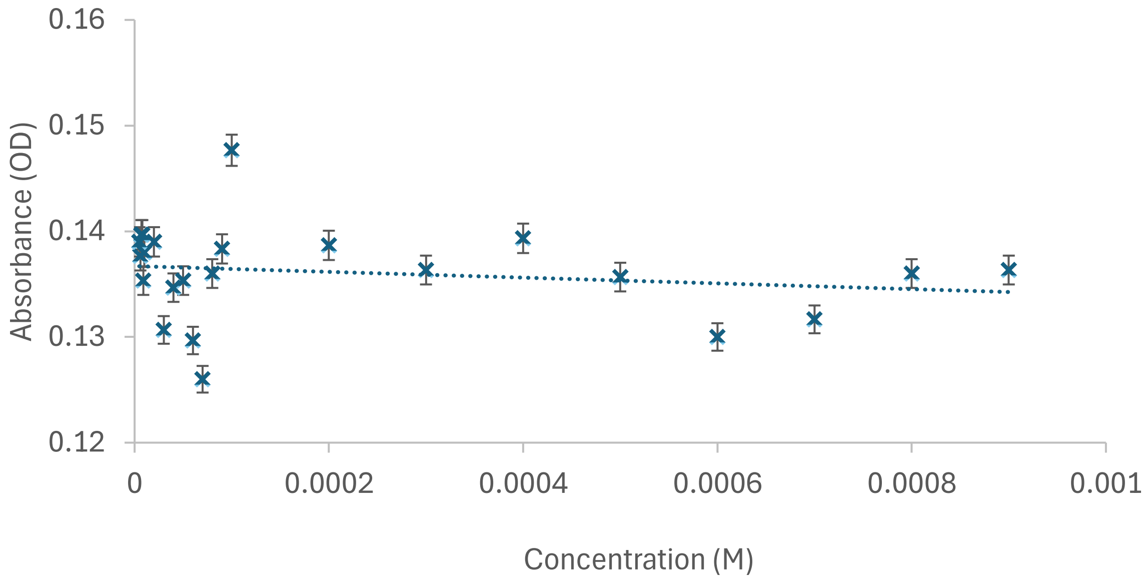

Figure S127 - UV-Vis dilution study data of **2** could not be fitted to either the EK or CoEK binding isotherm models. Spectral signals are hypothesised to be affected by compound aggregation, typical to self-associating amphiphiles of this nature, at concentrations > 0.0009 M.

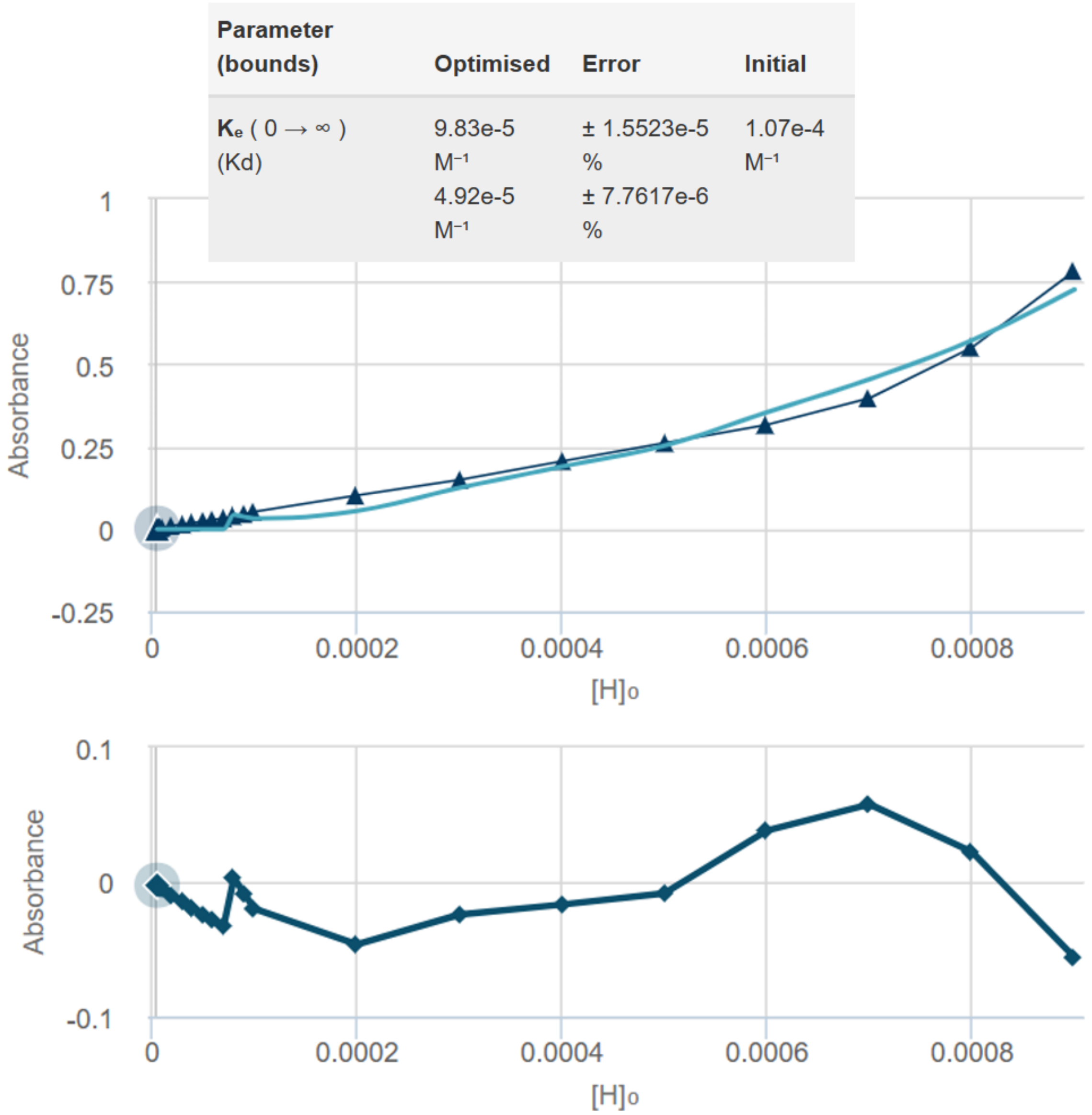

Parameter (bounds)	Optimised	Error	Initial
K_e (0 → ∞) (Kd)	9.83e-5 M^{-1}<br>4.92e-5 M^{-1}	± 1.5523e-5 %<br>± 7.7617e-6 %	1.07e-4 M^{-1}

Figure S128 - UV-Vis dilution study data of **3** fitted to the EK binding isotherm model using Bindfit v0.5/ (http://app.supramolecular.org/bindfit/view/2599d87f-9b75-4c00-85b5-a1cdb0e294eb). Fittings conducted at 0.0009 M – 0.000005 M with a dilution correction.

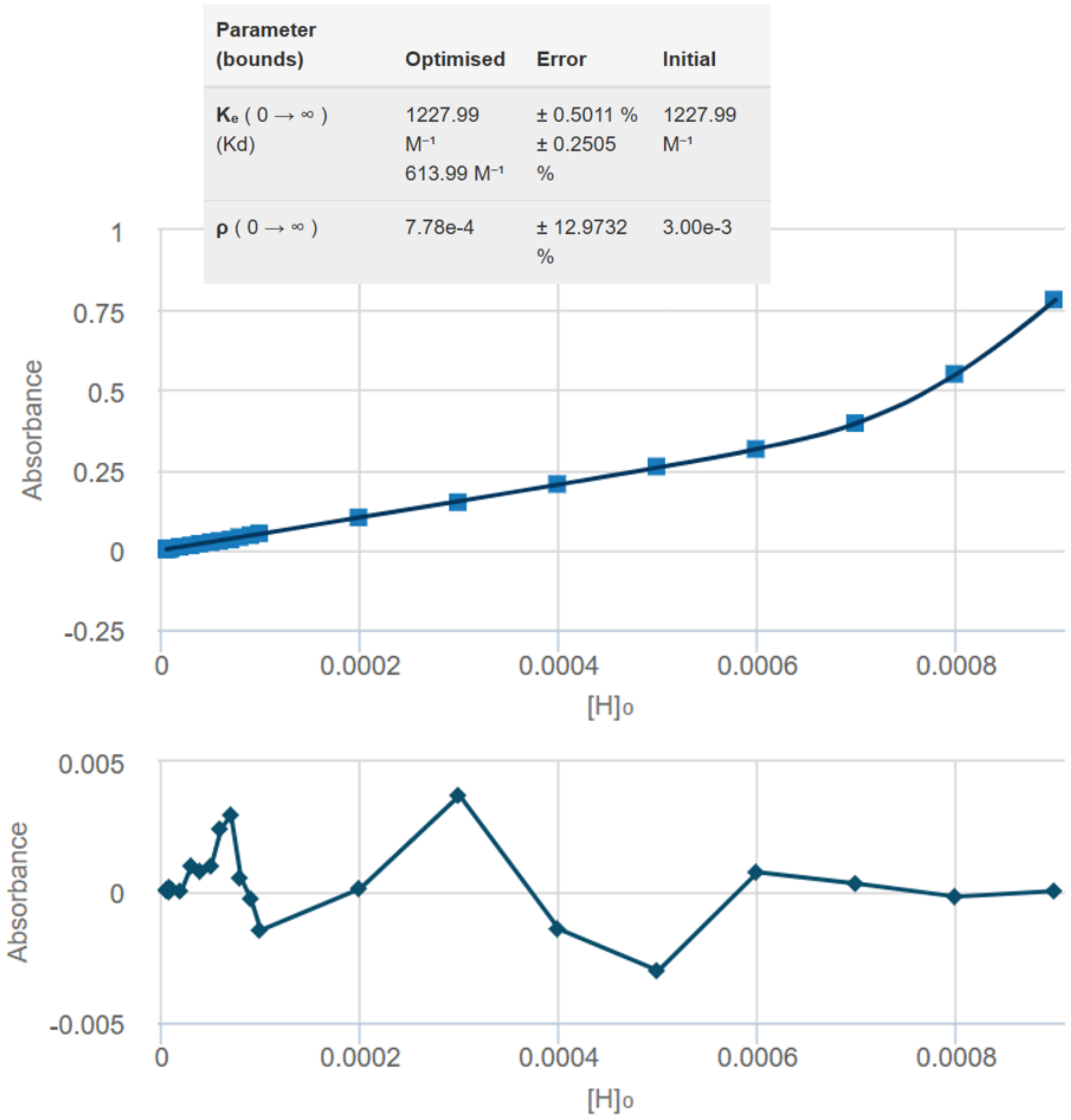

Parameter (bounds)	Optimised	Error	Initial
K_e (0 → ∞) (Kd)	1227.99 M^{-1} 613.99 M^{-1}	± 0.5011 % ± 0.2505 %	1227.99 M^{-1}
ρ (0 → ∞)	7.78e-4	± 12.9732 %	3.00e-3

Figure S129 - UV-Vis dilution study data of **3** fitted to the CoEK binding isotherm model using Bindfit v0.5/ (http://app.supramolecular.org/bindfit/view/853d876e-0d90-4ee3-a640-54a6c330a07a). Fittings conducted at 0.0009 M – 0.000005 M with a dilution correction.

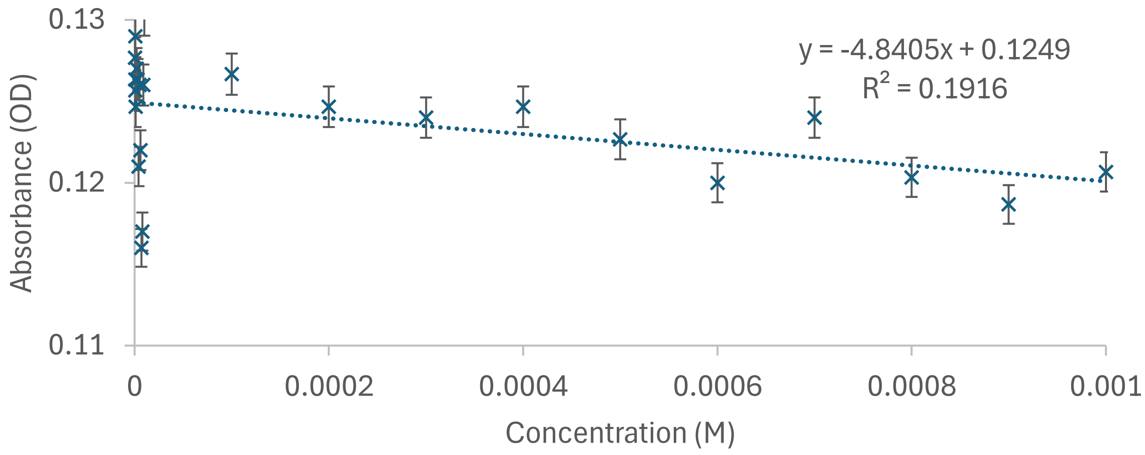

Figure S130 - UV-Vis dilution study data of **4** could not be fitted to either the EK or CoEK binding isotherm models.

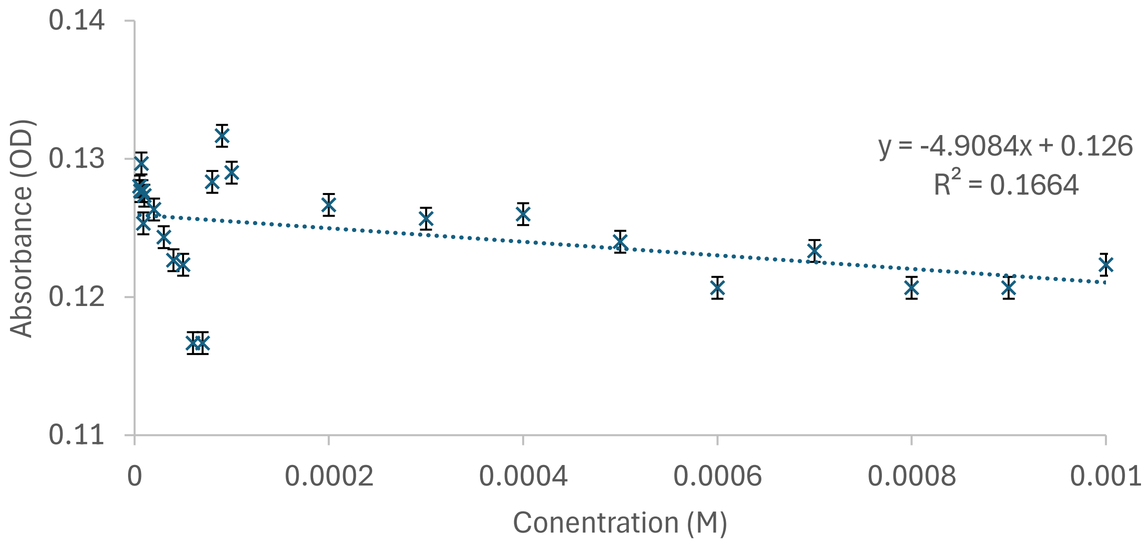

Figure S131 - UV-Vis dilution study data of **5** could not be fitted to either the EK or CoEK binding isotherm models.

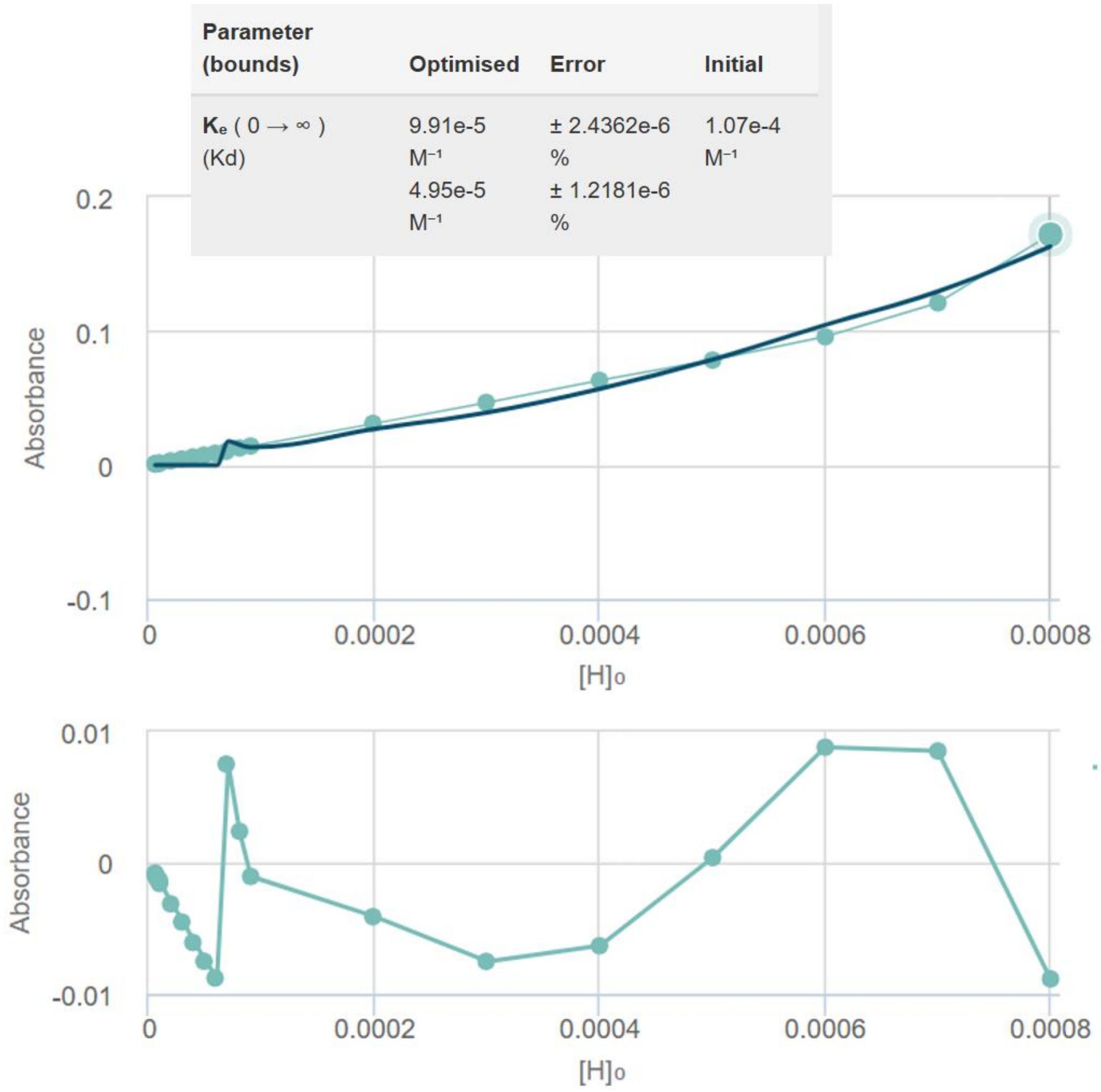

Figure S132 - UV-Vis dilution study data of **6** fitted to the EK binding isotherm model using Bindfit v0.5/ (http://app.supramolecular.org/bindfit/view/2035787a-cec2-45f0-86ef-f435c31e51aa). Fittings conducted at 0.0008 M – 0.000005 M with a dilution correction.

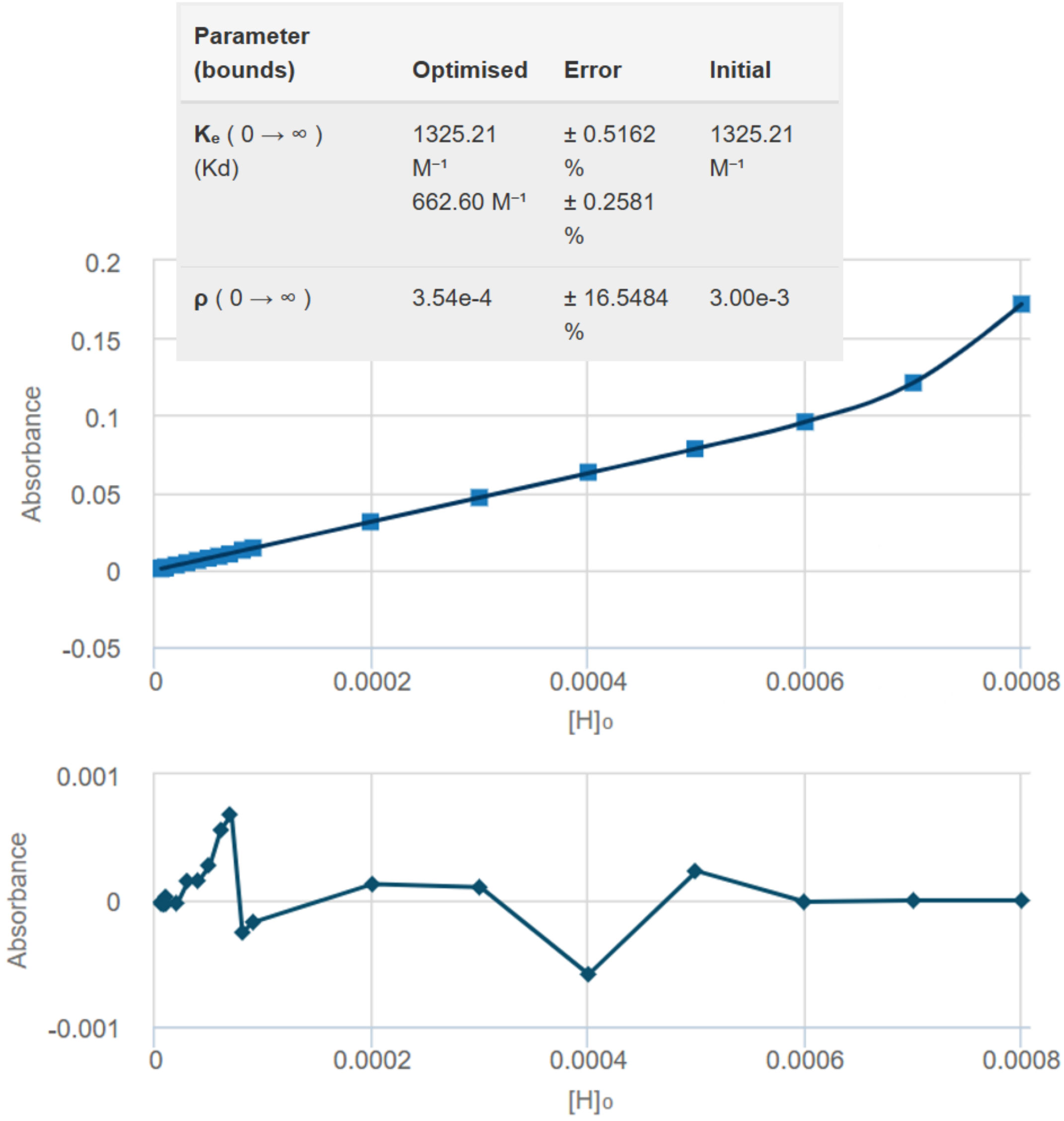

Figure S133 - UV-Vis dilution study data of **6** fitted to the CoEK binding isotherm model using Bindfit v0.5/ (http://app.supramolecular.org/bindfit/view/3e2d616d-ac23-4e89-8bfa-c0c651729e8b). Fittings conducted at 0.0008 M – 0.000005 M with a dilution correction.

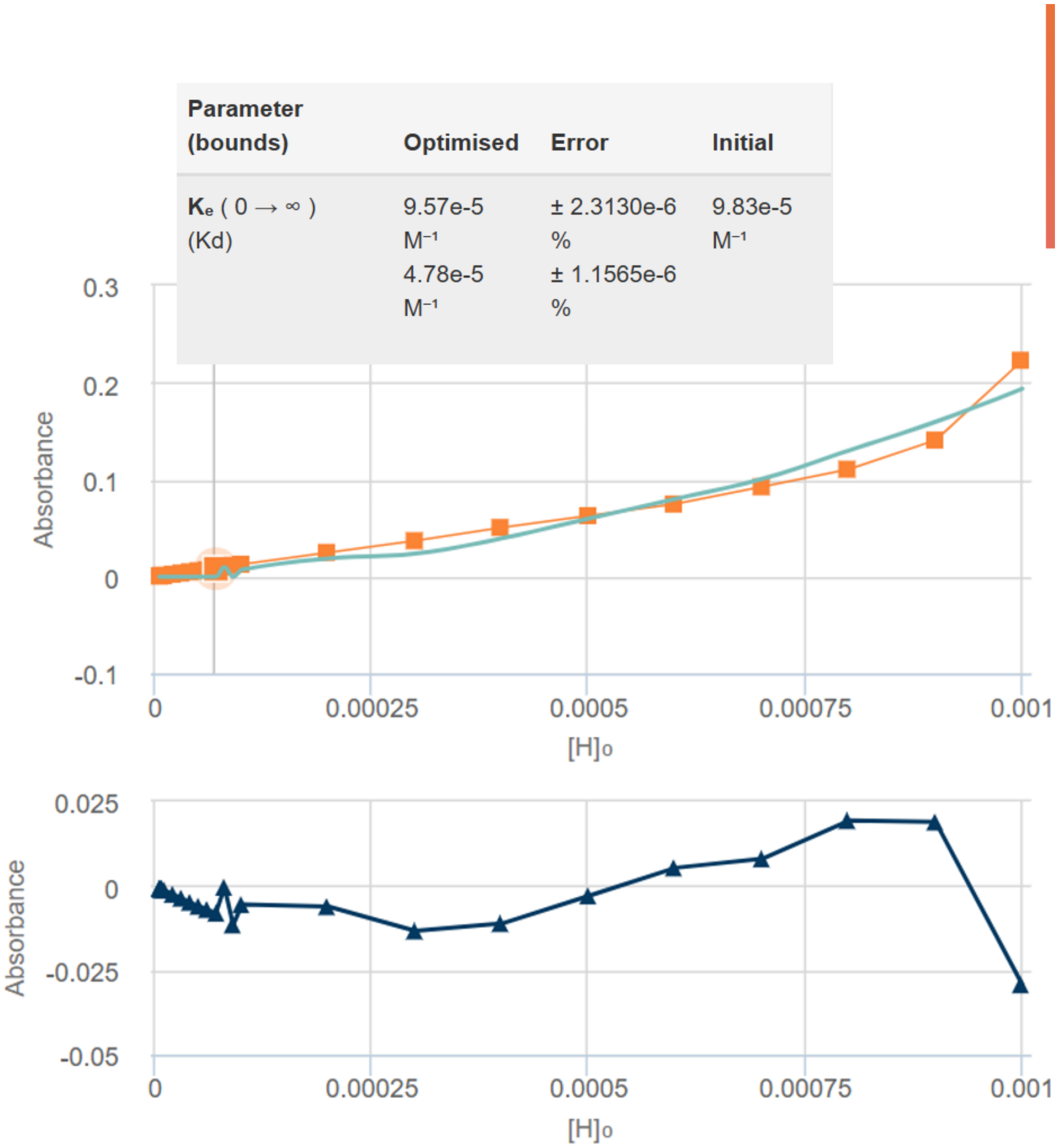

Parameter (bounds)	Optimised	Error	Initial
**K**$_e$ (0 → ∞) (Kd)	9.57e-5 M^{-1} 4.78e-5 M^{-1}	± 2.3130e-6 % ± 1.1565e-6 %	9.83e-5 M^{-1}

Figure S134 - UV-Vis dilution study data of **7** fitted to the EK binding isotherm model using Bindfit v0.5/ (http://app.supramolecular.org/bindfit/view/bf99c206-6bcb-4b2b-b711-84aa20c2c6d9). Fittings conducted at 0.001 M – 0.000005 M with a dilution correction.

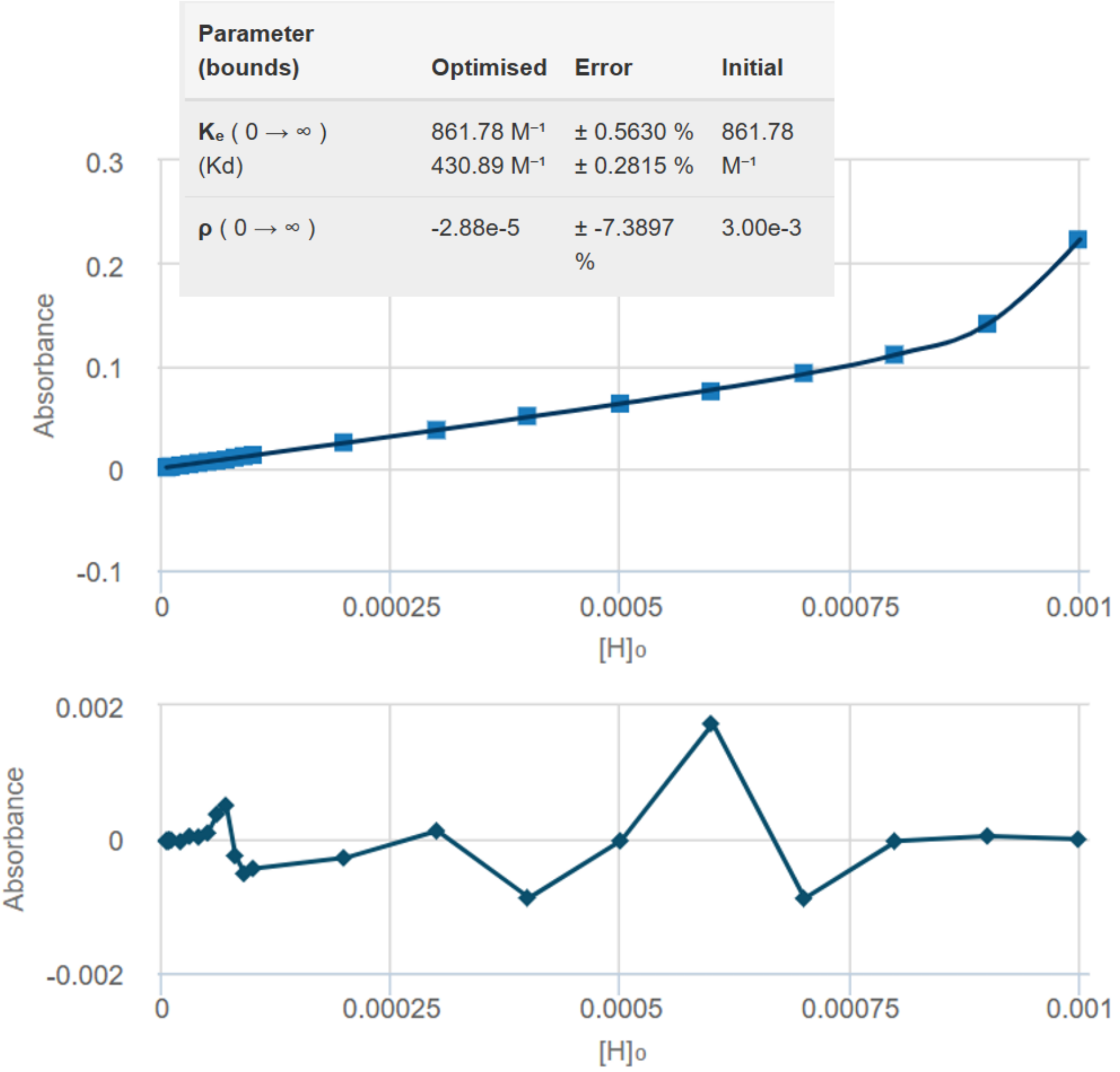

Parameter (bounds)	Optimised	Error	Initial
K_e (0 → ∞) (Kd)	861.78 M^{-1} 430.89 M^{-1}	± 0.5630 % ± 0.2815 %	861.78 M^{-1}
ρ (0 → ∞)	-2.88e-5	± -7.3897 %	3.00e-3

Figure S135 - UV-Vis dilution study data of **7** fitted to the CoEK binding isotherm model using Bindfit v0.5/ (http://app.supramolecular.org/bindfit/view/5c17cb21-99c5-467e-87e9-1c8f7e69f580). Fittings conducted at 0.001 M – 0.000005 M with a dilution correction.

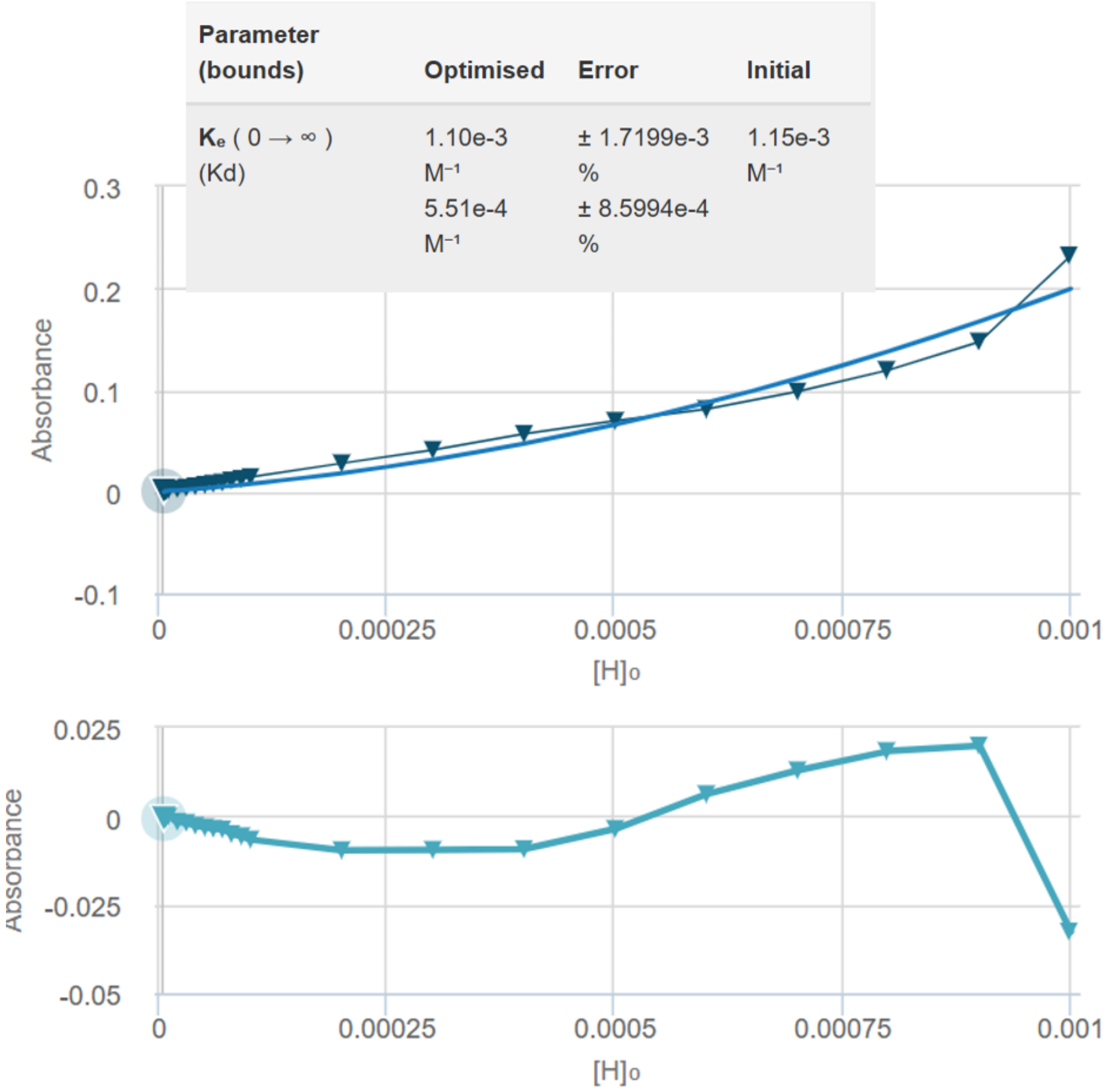

Figure S136 - UV-Vis dilution study data of **8** fitted to the EK binding isotherm model using Bindfit v0.5/ (http://app.supramolecular.org/bindfit/view/3f2a61f1-c6f6-4307-b7bd-11766bfa99db). Fittings conducted at 0.001 M – 0.000005 M with a dilution correction.

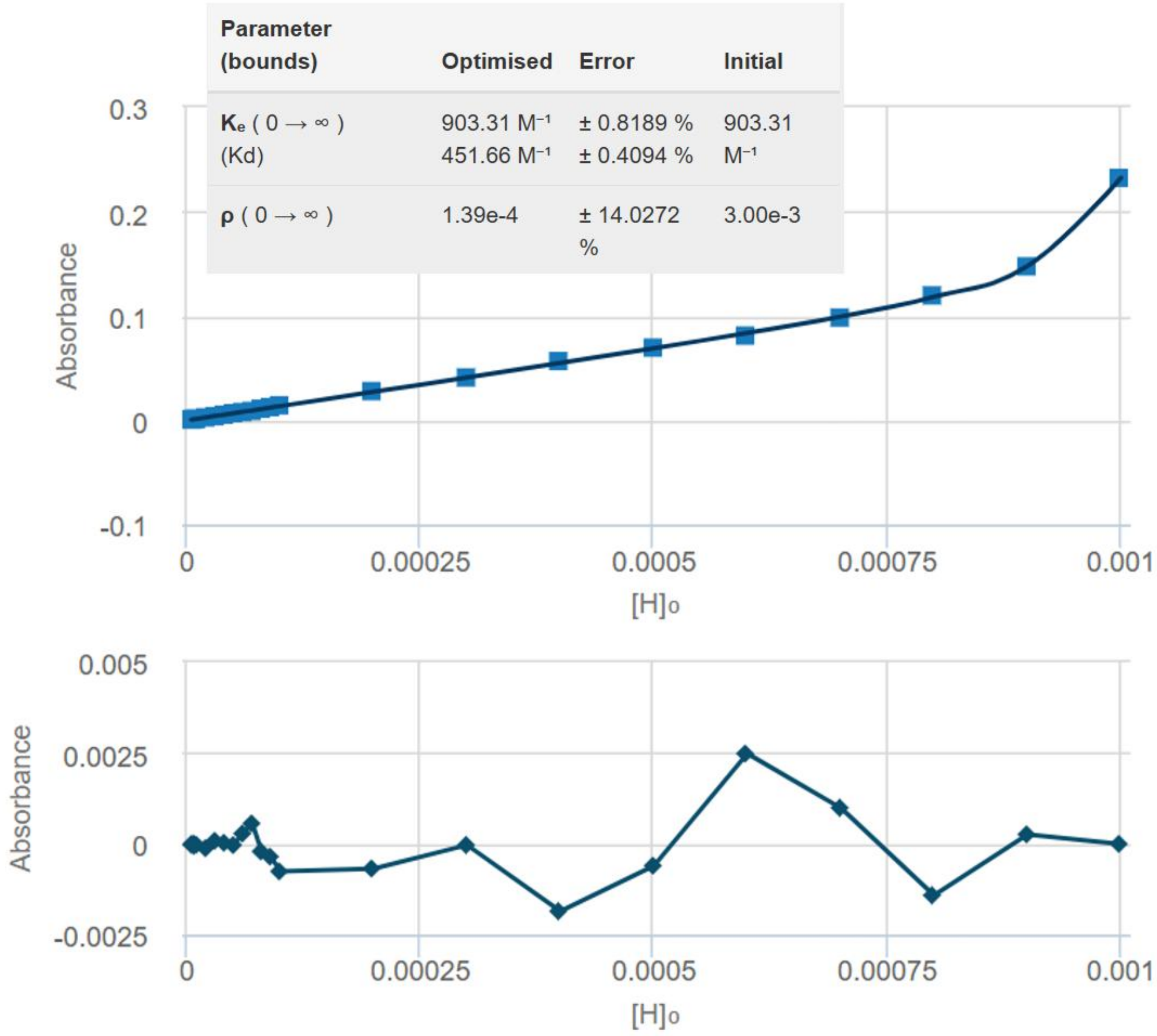

Figure S137 - UV-Vis dilution study data of **8** fitted to the CoEK binding isotherm model using Bindfit v0.5/ (http://app.supramolecular.org/bindfit/view/9f2b4dbb-c974-4641-9954-93375022f67a). Fittings conducted at 0.001 M – 0.000005 M with a dilution correction.

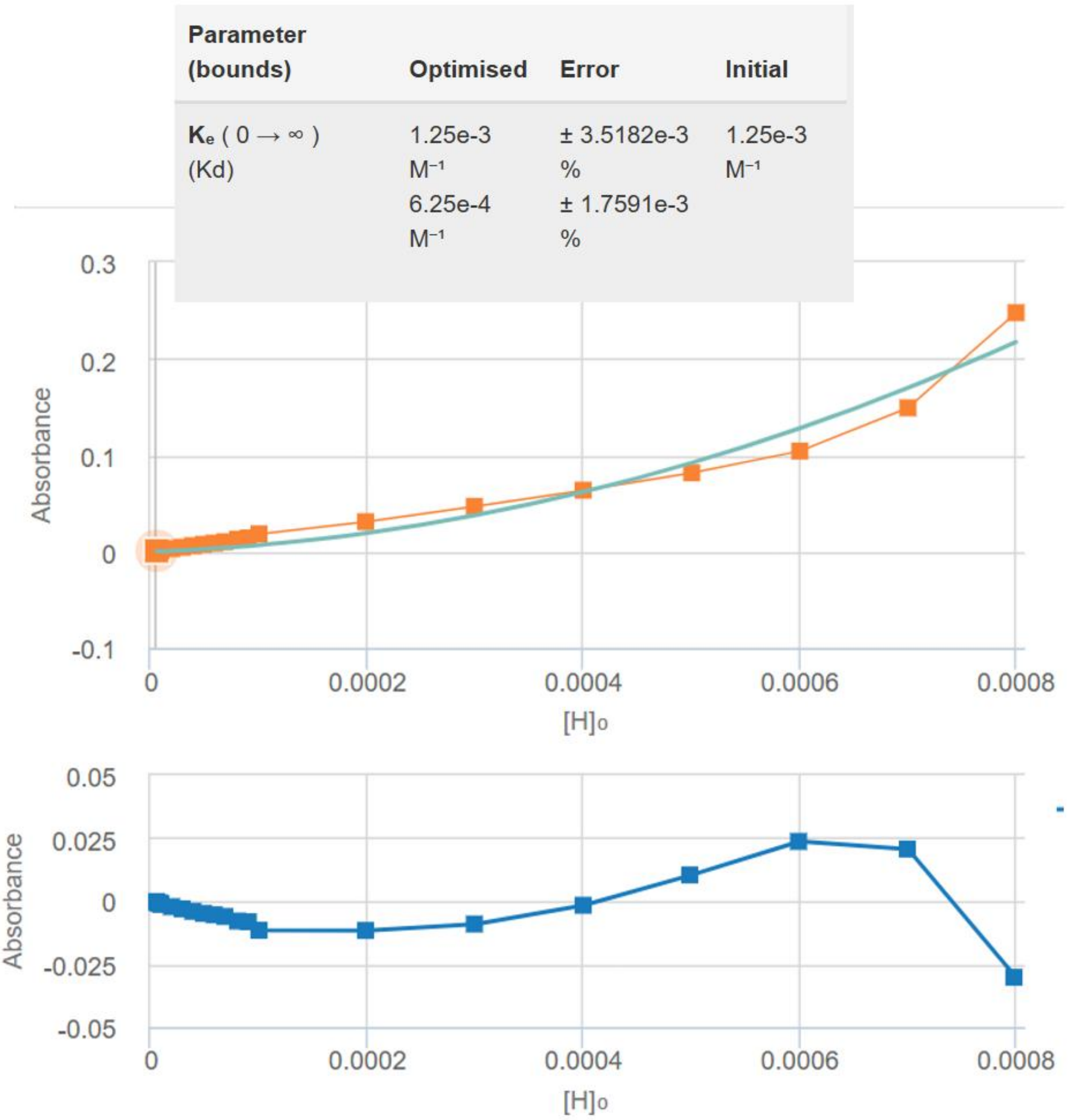

Figure S138 - UV-Vis dilution study data of **9** fitted to the EK binding isotherm model using Bindfit v0.5/ (http://app.supramolecular.org/bindfit/view/e4550ed9-fc7d-43d2-95bb-be342391d66b). Fittings conducted at 0.0008 M – 0.000005 M with a dilution correction.

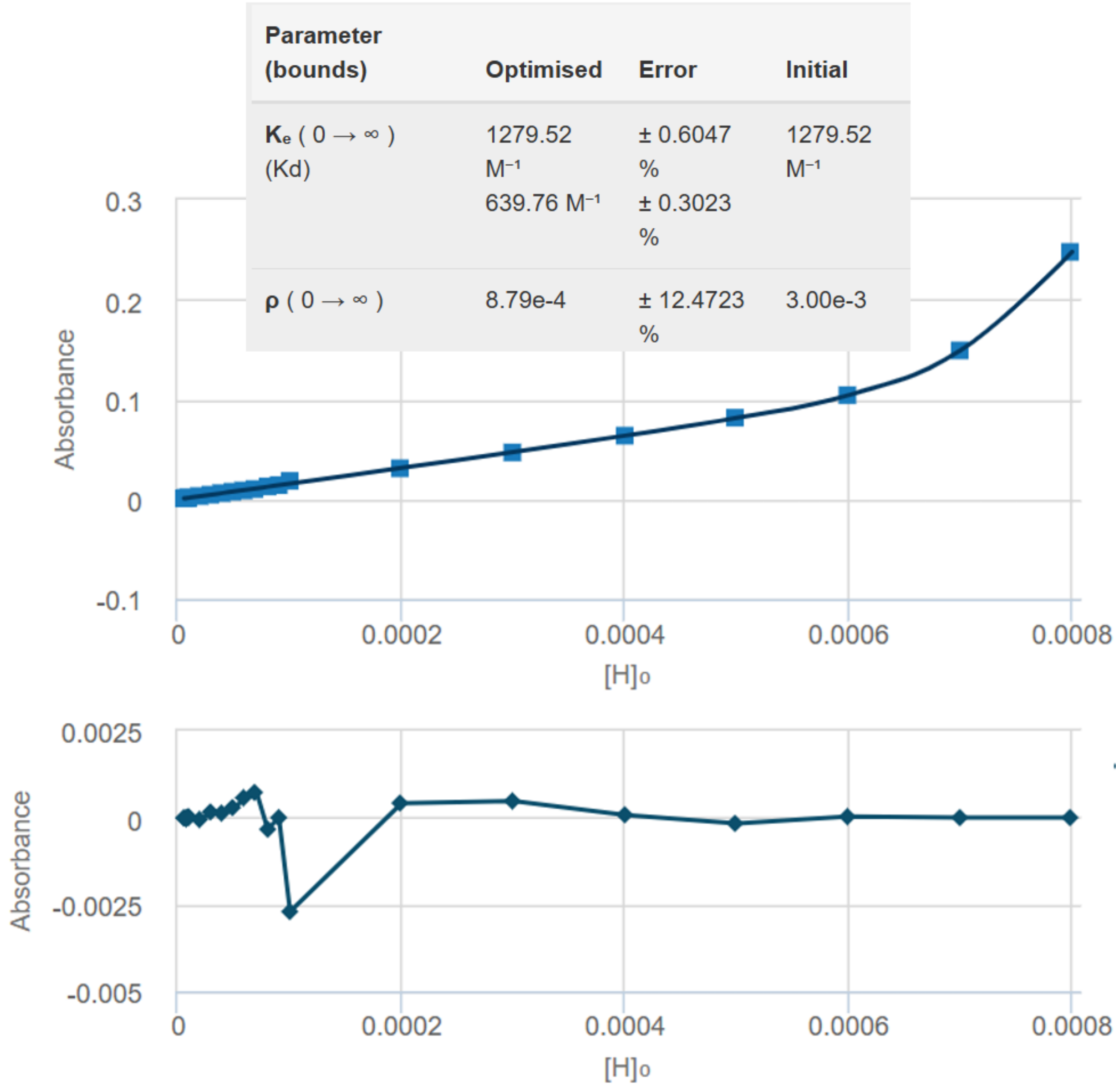

Parameter (bounds)	Optimised	Error	Initial
K_e (0 → ∞) (Kd)	1279.52 M^{-1} 639.76 M^{-1}	± 0.6047 % ± 0.3023 %	1279.52 M^{-1}
ρ (0 → ∞)	8.79e-4	± 12.4723 %	3.00e-3

Figure S139 - UV-Vis dilution study data of **9** fitted to the CoEK binding isotherm model using Bindfit v0.5/ (http://app.supramolecular.org/bindfit/view/9c1bd809-c339-406e-bd20-8fa656160b5e). Fittings conducted at 0.0008 M – 0.000005 M with a dilution correction.

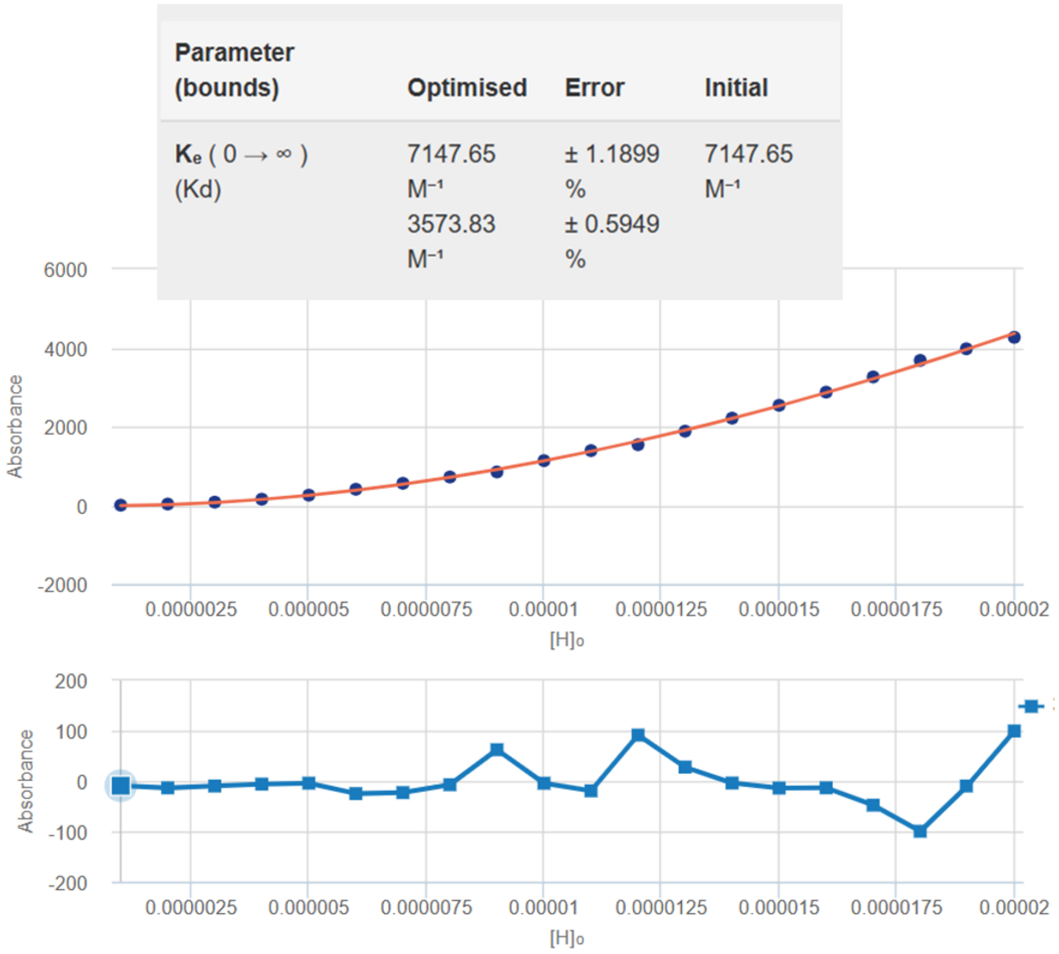

Parameter (bounds)	Optimised	Error	Initial
K_e (0 → ∞) (Kd)	7147.65 M^{-1}<br>3573.83 M^{-1}	± 1.1899 %<br>± 0.5949 %	7147.65 M^{-1}

Figure S140 - UV-Vis dilution study data of **10** fitted to the EK binding isotherm model using Bindfit v0.5/ (http://app.supramolecular.org/bindfit/view/3b175474-79a8-4134-abdf-ce2e8c7bf467). Fittings conducted at 0.000002 M - 0.00002 M with a dilution correction.

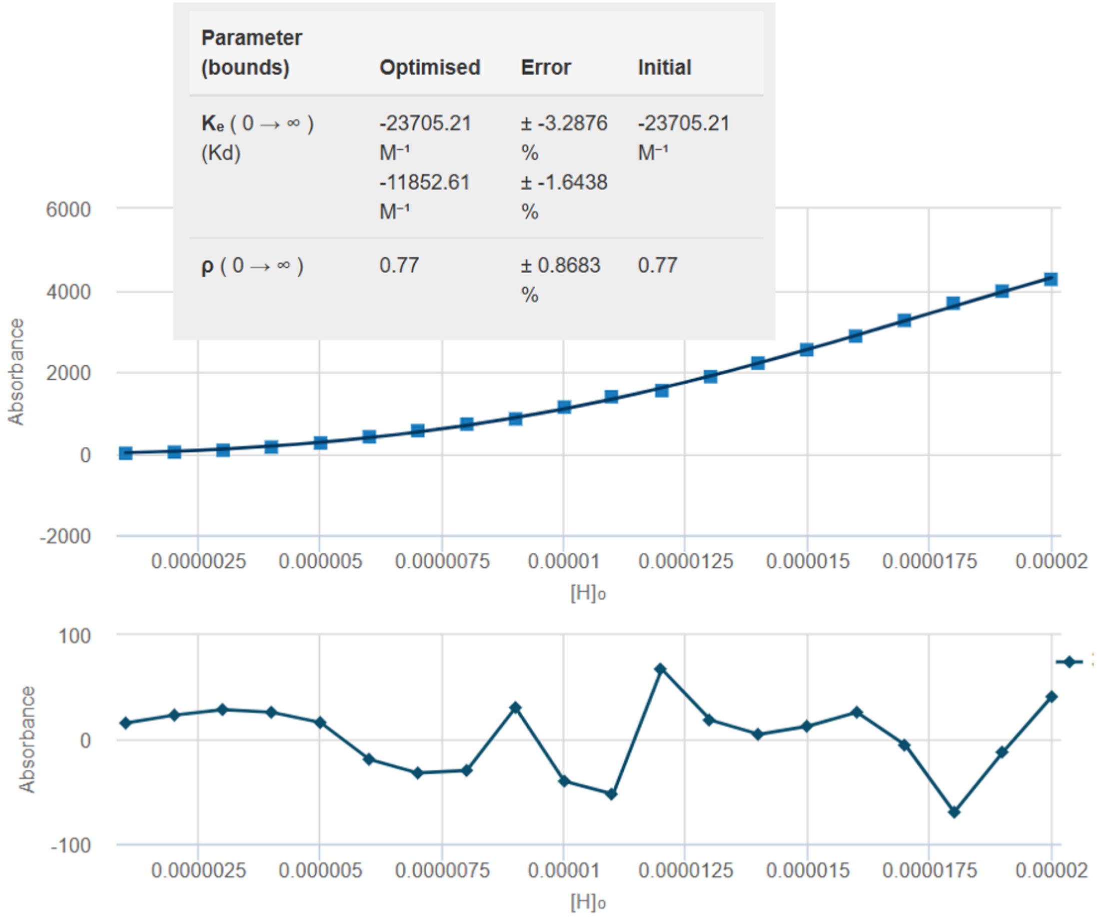

Figure S141 - UV-Vis dilution study data of **10** fitted to the CoEK binding isotherm model using Bindfit v0.5/ (http://app.supramolecular.org/bindfit/view/9d746826-f869-4a47-ba12-e8f8af221f94). Fittings conducted at 0.000002 M - 0.00002 M with a dilution correction.

Section S10: Mass Spectrometry data

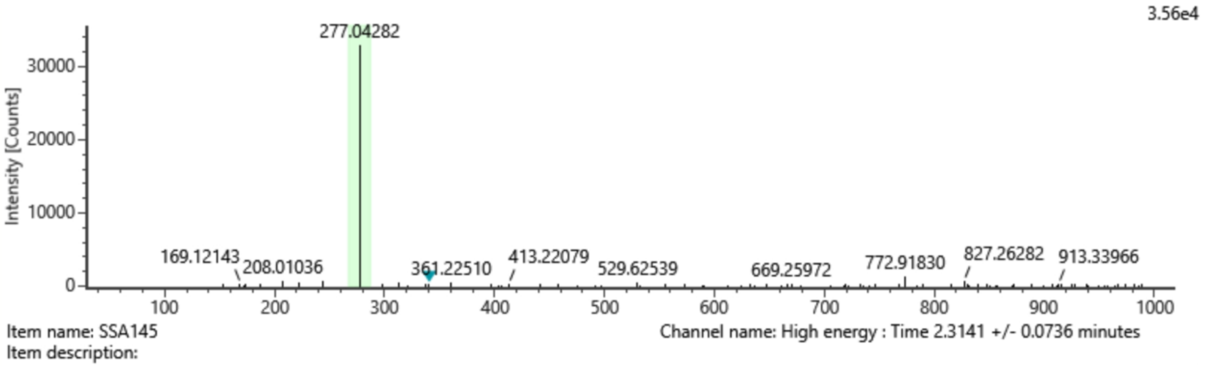

Figure S142 - A high-resolution mass spectrum (ESI$^-$) obtained for **1**, m/z [M]$^-$. Data obtained by Dr P. Hailey.

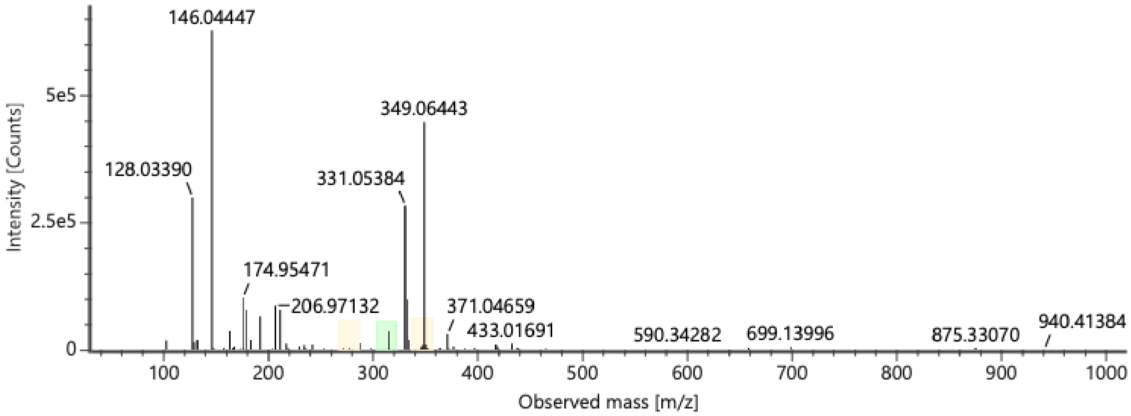

Figure S143 - A high-resolution mass spectrum (ESI$^-$) obtained for **2**, m/z [M]$^-$. Data obtained by Dr P. Hailey.

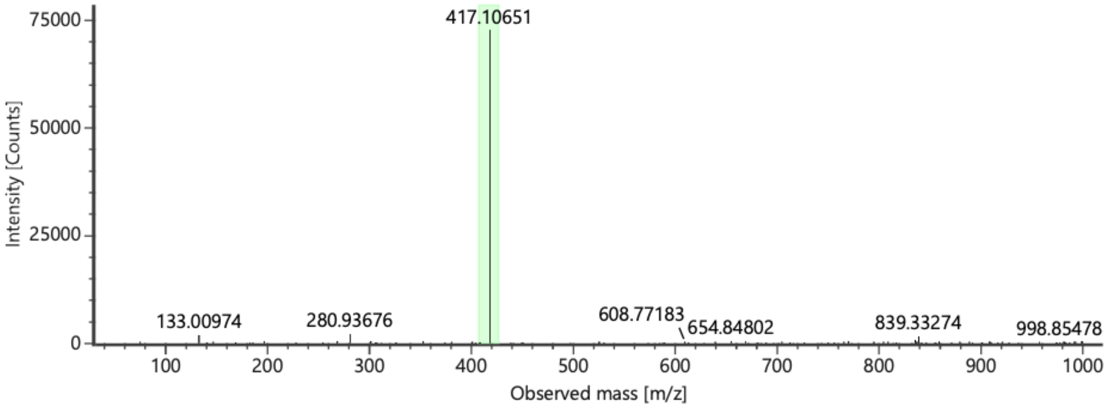

Figure S144 - A high-resolution mass spectrum (ESI$^-$) obtained for **3**, m/z [M]$^-$. Data obtained by Dr P. Hailey.

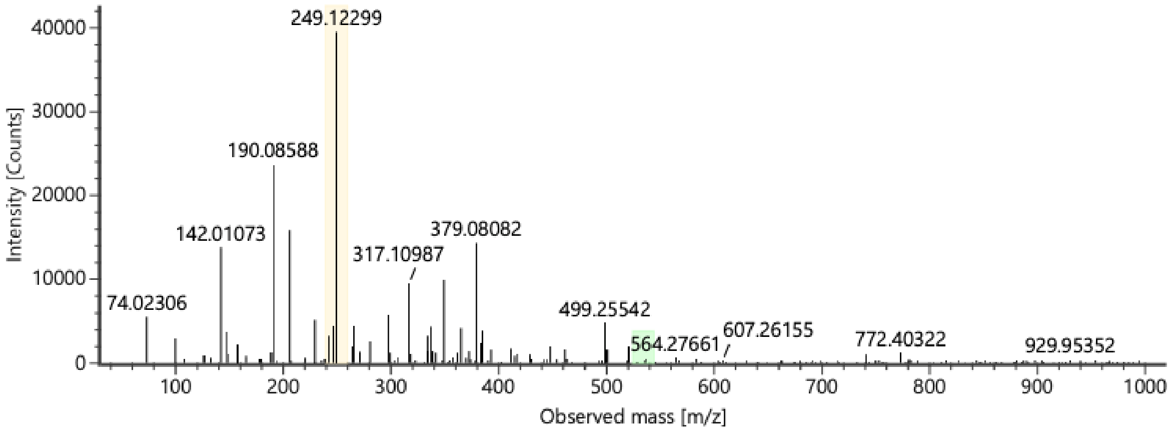

Figure S145 - A high-resolution mass spectrum (ESI$^-$) obtained for **4**, m/z [M]$^-$. Data obtained by Dr P. Hailey.

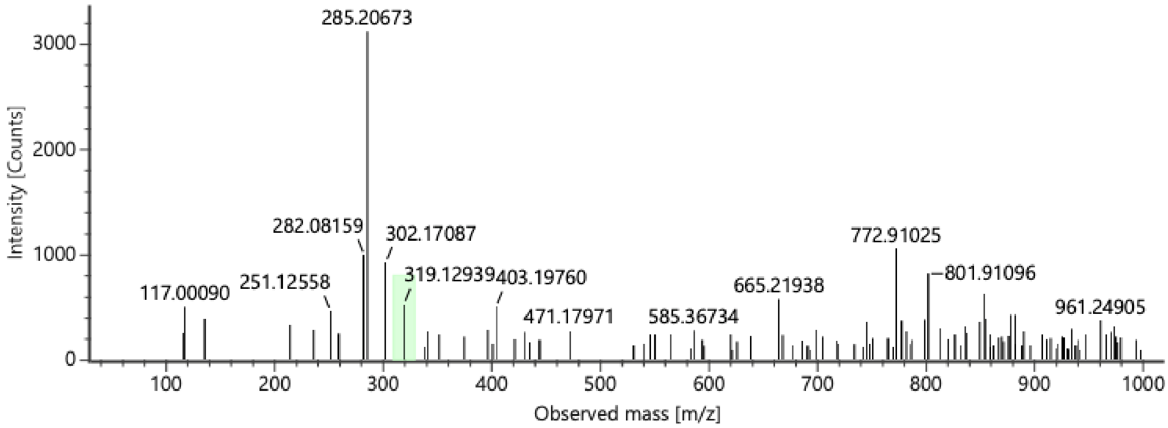

Figure S146 - A high-resolution mass spectrum (ESI$^-$) obtained for **5**, m/z [M]$^-$. Data obtained by Dr P. Hailey.

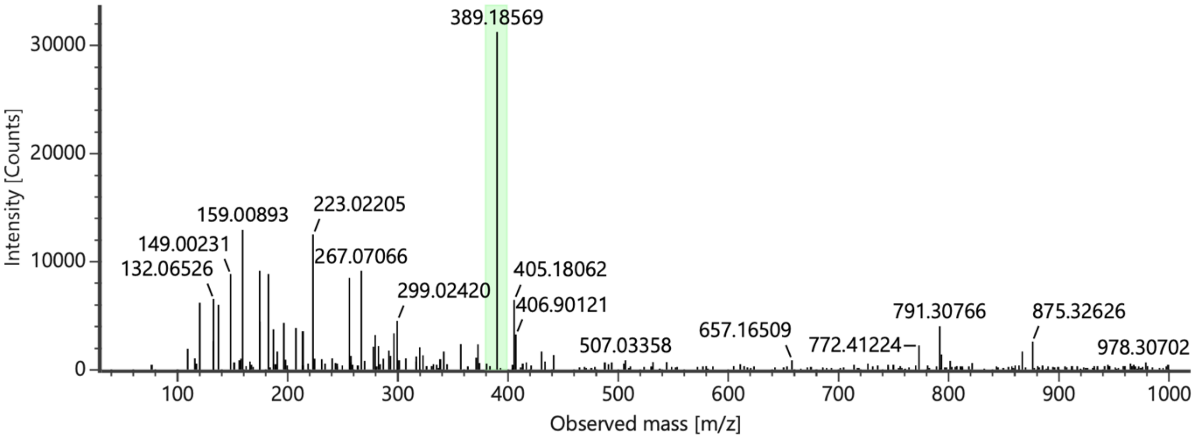

Figure S147 - A high-resolution mass spectrum (ESI$^-$) obtained for **6**, m/z [M]$^-$. Data obtained by Dr P. Hailey.

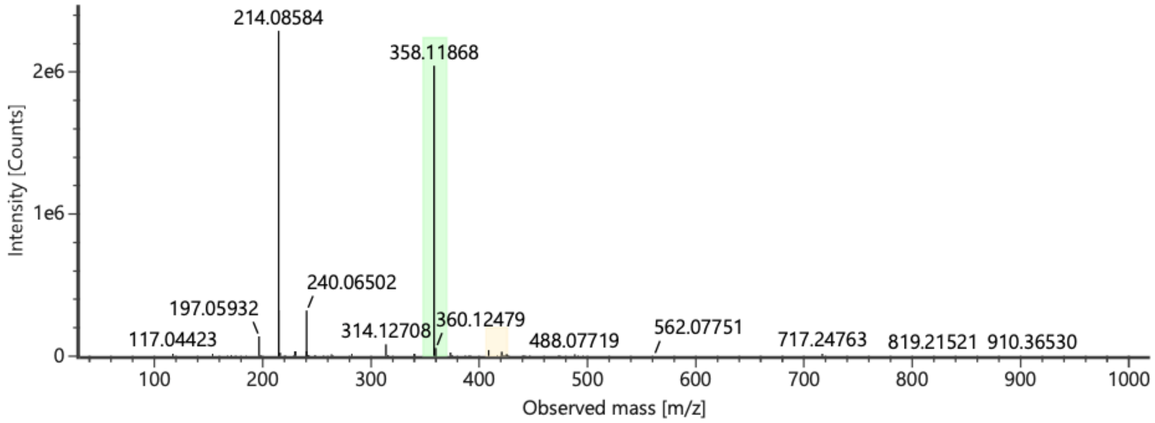

Figure S148 - A high-resolution mass spectrum (ESI$^-$) obtained for **7**, m/z [M]$^-$. Data obtained by Dr P. Hailey.

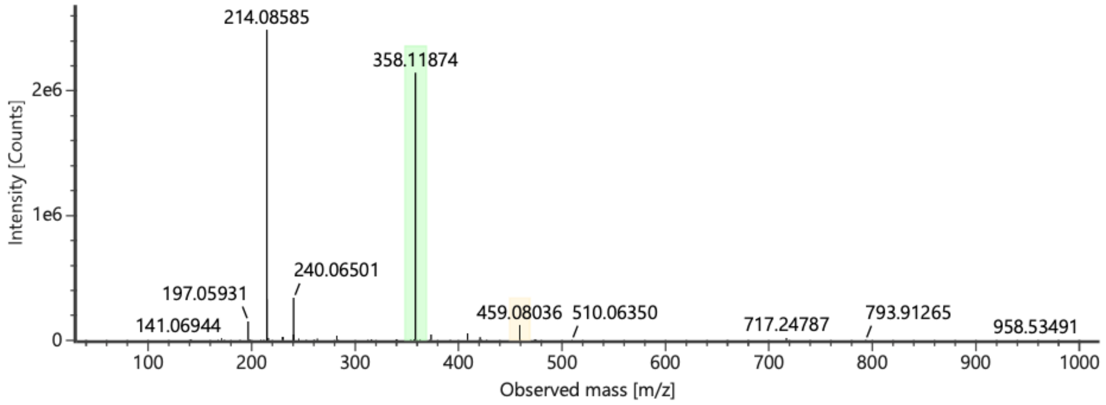

Figure S149 - A high-resolution mass spectrum (ESI$^-$) obtained for **8**, m/z [M]$^-$. Data obtained by Dr P. Hailey.

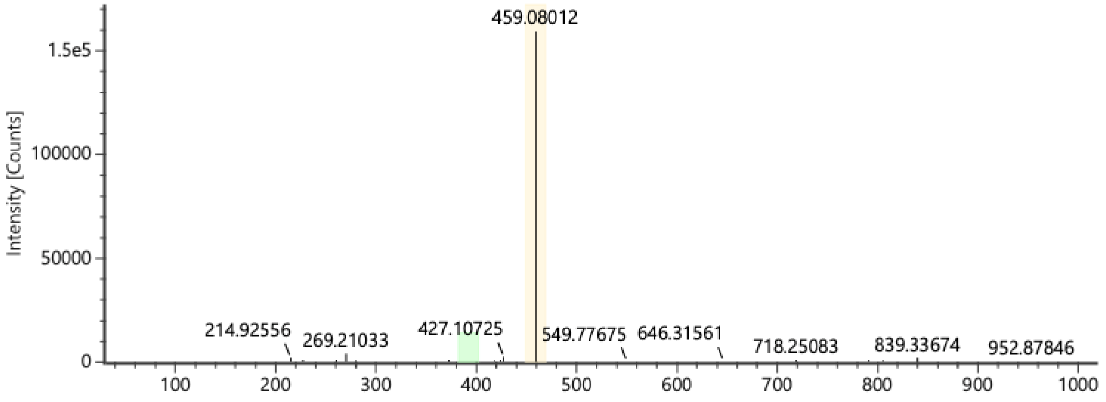

Figure S150 - A high-resolution mass spectrum (ESI$^-$) obtained for **9**, m/z [M]$^-$. Data obtained by Dr P. Hailey.

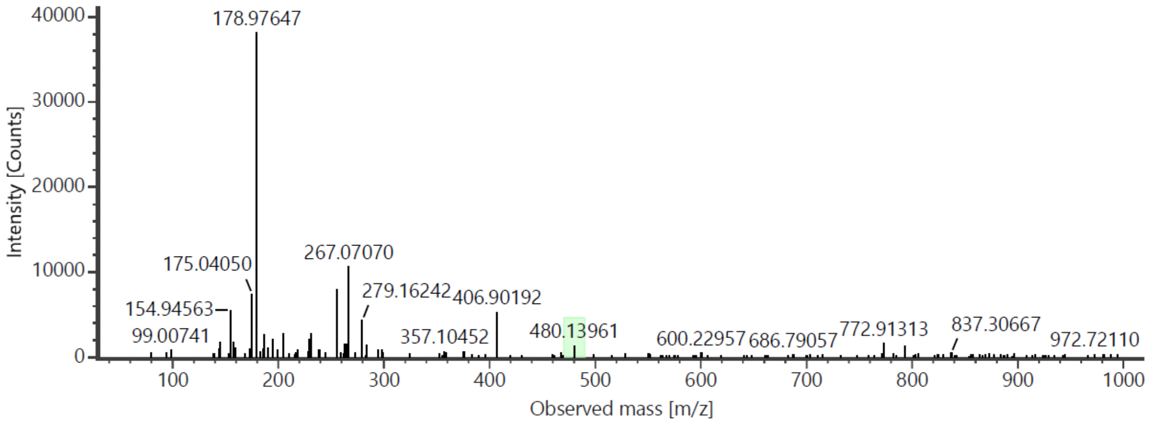

Figure S151 - A high-resolution mass spectrum (ESI$^-$) obtained for **10**, m/z [M]$^-$. Data obtained by Dr P. Hailey.
